# Cu-Promoted *ipso*-Hydroxylation of sp^2^ Bonds with Concomitant Aromatic 1,2-Rearrangement Involving a Cu-oxyl-hydroxo Species

**DOI:** 10.1021/acs.inorgchem.4c03304

**Published:** 2024-10-18

**Authors:** Sunipa Goswami, Karan Gill, Xinyi Yin, Marcel Swart, Isaac Garcia-Bosch

**Affiliations:** † Department of Chemistry, Carnegie Mellon University, Pittsburgh, Pennsylvania 15213, United States; ψ University of Girona, Campus Montilivi (Ciències), IQCC, Girona, Spain, ICREA, Pg. Lluís Companys 23, 08010 Barcelona, Spain

## Abstract

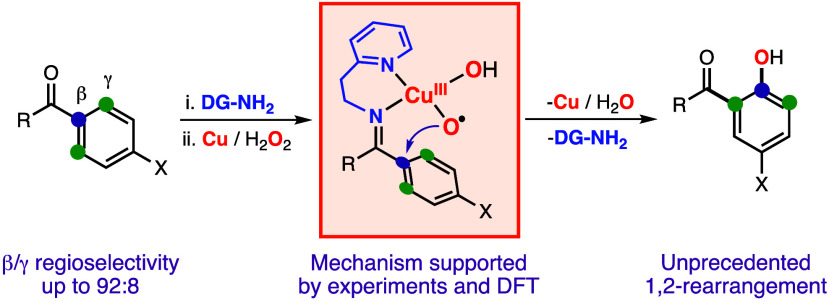

Herein, we report the first example of Cu-promoted β *ipso*-hydroxylation of substituted benzophenones using a bidentate directing group (DG) and H_2_O_2_ as an oxidant. In addition to the new C–O bond formed, the *ipso*-oxidation induces a very unusual 1,2-rearrangement of the iminyl group to the vicinal γ position. This transformation is highly dependent on the substrate utilized (favored for 4-methoxy-substituted benzophenones) and on the DG used (2-picolylamine leads to selective γ-C–H functionalization, while β *ipso*-oxidation requires 2-(2-aminoethyl)pyridine). An analysis of the oxidation of substrate–ligands derived from 2-(2-aminoethyl)pyridine and unsymmetrical 4-MeO-substituted benzophenones indicates high regioselectivity (up to 89:11 for the MeO-substituted arene ring and up to 92:8 for β *ipso*- vs γ-C–H hydroxylation). Mechanistic studies (which include spectroscopic characterization of reaction intermediates, kinetics, and calculations) suggest the formation of a mononuclear Cu^II^OOH species before the rate-determining step (rds) of the reaction. DFT calculations suggest that the γ-C–H hydroxylation pathway involves a one-step concerted O–O cleavage and electrophilic aromatic attack. Conversely, β *ipso*-hydroxylation occurs in a stepwise fashion, in which O–O bond cleavage produces a Cu^III^(O·)(OH) before electrophilic aromatic attack. Calculations also shed light on the mechanism of the 1,2-rearrangement step, which involves strain release from a spiro 5-membered to a 6-membered Cu chelate.

## Abstract 2

In this research article, we report the Cu-directed *ipso*-hydroxylation of benzophenones using a bidentate directing group (DG) and H_2_O_2_ as an oxidant. The *ipso*-oxidation induces an unusual 1,2-rearrangement of the iminyl group to the vicinal γ position. Analysis of the oxidation of substrate−ligands derived from unsymmetrical 4-MeO-substituted benzophenones indicates a high preference for the ipso-functionalization of the electron-rich ring. Experiment and computational studies reveal that the oxidation occurs via the formation of a Cu^III^-oxyl-hydroxo intermediate.

## Introduction

Cu-dependent metalloenzymes catalyze a wide array of oxidative transformations using O_2_ under mild conditions (i.e., room temperature, atmospheric pressure).^1−3^ Lytic polysaccharide monooxygenase enzymes (LPMOs) promote the C–H hydroxylation and subsequent cleavage of the polysaccharide chains found in natural materials such as cellulose and chitin.^4−6^ Recent reports on the reactivity of LPMOs suggest that, instead of O_2_, these Cu-dependent metalloenzymes might utilize H_2_O_2_ as an oxidant.^7,8^ In 2015, our research laboratory reported that the catalytic hydroxylation of strong C–H bonds (e.g., cyclohexane) using Cu and H_2_O_2_ proceeded via the formation of nonselective Fenton-like oxidants (hydroxyl and hydroperoxyl radicals).^9^ To achieve regioselectivity, LPMOs bind the organic substrate before exposing the Cu center to the oxidant, a reaction that leads to the formation of a highly organized ternary complex prior to substrate hydroxylation (i.e., metal–substrate–oxidant).^10,11^

**Figure 1:**
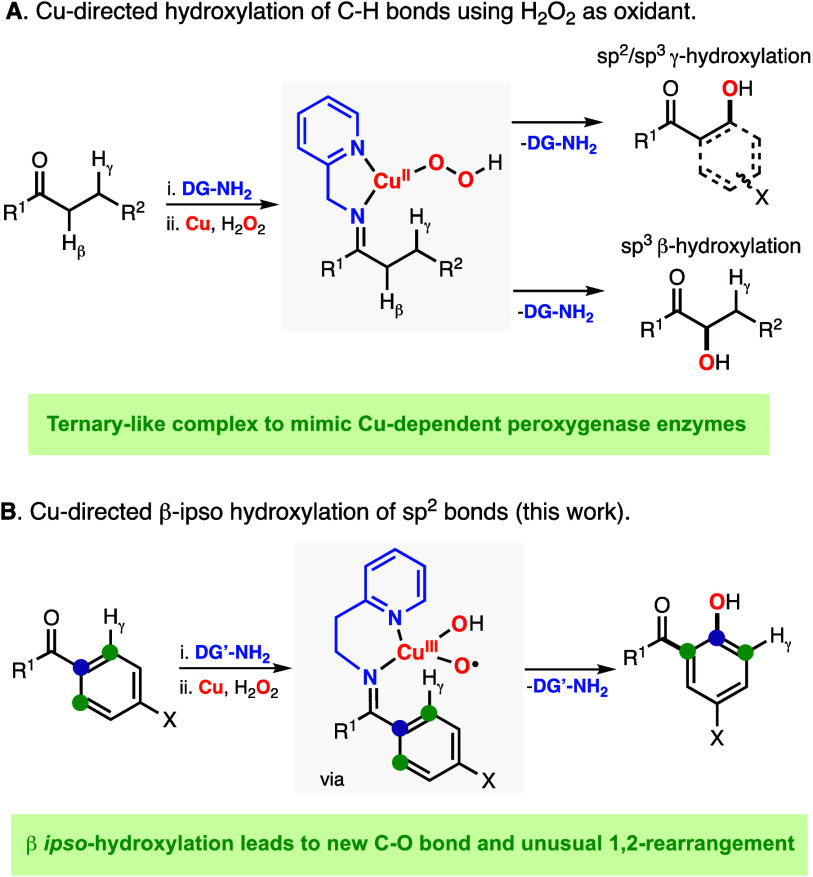
(A) Cu-directed hydroxylation of sp^2^ and sp^3^ C–H bonds using Cu, H_2_O_2_, and 2-picolylamine as the DG. (B) β-*ipso*-Hydroxylation of sp^2^ substrates using Cu, H_2_O_2_, and 2-(2-aminoethyl)pyridine as the DG.

Inspired by the reactivity of LPMOs, our research laboratory has published a series of research articles in which we utilize Cu, H_2_O_2_, and directing groups to promote regioselective C–H hydroxylation of ketones and aldehydes (Figure 1A).^12−15^ Most of our research has focused on the utilization of 2-picolylamine as DG, which allows the γ-hydroxylation of sp^3^ C–H bonds (e.g., steroids), the γ-hydroxylation of sp^2^ C–H bonds (e.g., benzophenones), and the β-hydroxylation of sp^3^ C–H bonds (e.g., dicyclohexyl ketone). Herein, we report that the utilization of Cu, H_2_O_2_, and 2-(2-aminoethyl)pyridine as DG leads to β *ipso*-hydroxylation, an oxidation that also entails a very unusual iminyl 1,2-rearrangement (breaking the C_α_–C_β_ bond and forming a C_α_–C_γ_ bond), an unprecedented transformation in metal-directed functionalization reactions.

## Results and Discussion

### Hydroxylation of 4,4′-Dimethoxybenzophenone

**Figure 2:**
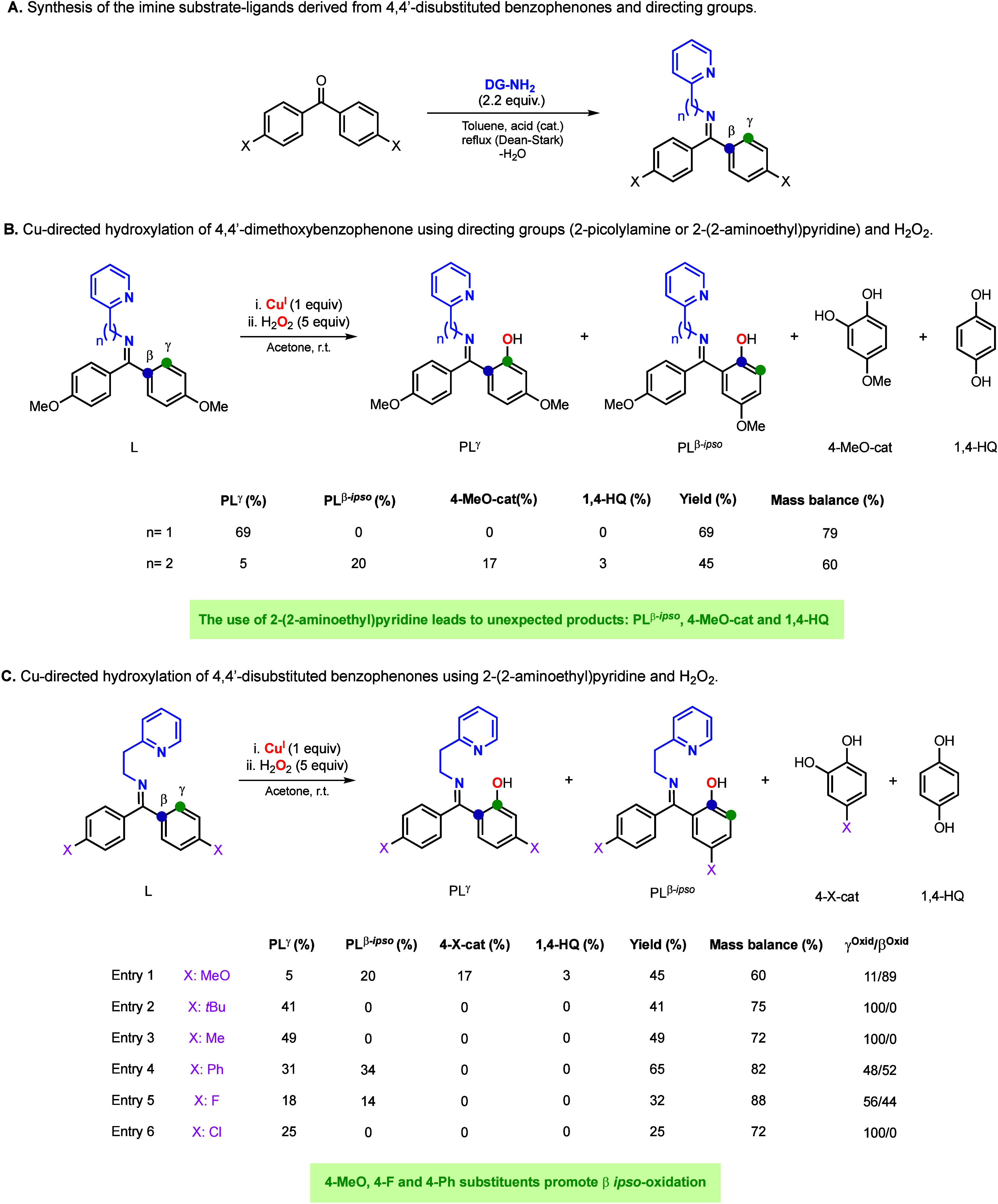
(A) Synthesis of imine substrate–ligands derived from 4,4′-dimethoxybenzophenone and bidentate DGs. (B) Hydroxylation substrate–ligands derived from 4,4′-dimethoxybenzophenone using Cu and H_2_O_2_. (C) Hydroxylation of symmetrical benzophenones using Cu, H_2_O_2_, and 2-(2-aminoethyl)pyridine as the DG.

Our research laboratory has recently reported the hydroxylation of substituted benzophenones using Cu^I^, H_2_O_2_, and 2-picolylamine.^13,15^ Our synthetic protocol entails the installation of the directing group, which leads to the formation of substrate–ligands that are isolated and characterized by ^1^H NMR (Figure 2A). The oxidation of the resulting imine substrate–ligands is carried out in acetone using 1 equiv of [Cu^I^(CH_3_CN)_4_](PF_6_) and 5 equiv of aqueous H_2_O_2_ (Figure 2B). For 2-picolyamine, we observed the formation of the hydroxylation product derived from γ sp^2^ C–H hydroxylation (PL^γ^) with good yields and good mass balances (69 and 79%, respectively).

A similar protocol was followed for the hydroxylation of 4,4′-dimethoxybenzophenone with 2-(2-aminoethyl)pyridine. After synthesizing the corresponding imine substrate–ligand (see the S.I.), we performed its oxidation under our standard conditions (1 equiv of Cu^I^ and 5 equiv of H_2_O_2_ in acetone). Unexpectedly, an array of oxidation products were formed (Figure 2B). In addition to the product derived from γ-C–H hydroxylation (PL^γ^: 5%), we also identified the hydroxylation product derived from β *ipso*-hydroxylation (PL^β*-ipso*^, 20%), 4-methoxycathechol (4-MeO-cat, 17%), and 1,4-dihydroquinone (1,4-H_2_Q, 3%). Note: see the S.I. for details on the NMR quantification of the oxidation products, including the characterization of the hydroxylation/1,2-rearragement product PL^β*-ipso*^. As we show in the sections below, the catechol and 1,4-dihydroquinone byproducts are derived from β *ipso*-oxidation.

### Hydroxylation of Symmetrical 4,4′-Disubstitued Benzophenones

Based on the results obtained in the hydroxylation of 4,4′-(MeO)_2_-benzophenone using 2-(2-aminoethyl)pyridine, we analyzed if other symmetrical 4,4′-disubstitued-benzophenones could also undergo β *ipso*-hydroxylation (Figure 2C). On one hand, we observed the formation of products derived from β *ipso*-oxidation for 4,4′-dimethoxybenzophenone, 4,4′-diphenylbenzophenone, and 4,4′-difluorobenzophenone (entries 1, 4, and 5 in Figure 2C). On the other hand, the oxidation of the imine substrate–ligands derived from 4,4′-di-*tert-*butylbenzophenone, 4,4′-dimethylbenzophenone, and 4,4′-dichlorobenzophenone produced only PL^γ^ (entries 2, 3, and 6 in Figure 2C). Interestingly, the regioselectivity of the hydroxylation depended on the substituent, with 4-MeO favoring β *ipso*-oxidation (γ^oxid^/β^oxid^: 11/89) and with 4-Ph and 4-F forming equimolar mixtures of both hydroxylation products (γ^oxid^/β^oxid^: 48/52 and 56/44, respectively). Moreover, the formation of catechol and hydroquinone was observed only in the oxidation of the 4,4′-dimethoxybenzophenone system.

### Hydroxylation of Unsymmetrical Benzophenones

**Figure 3:**
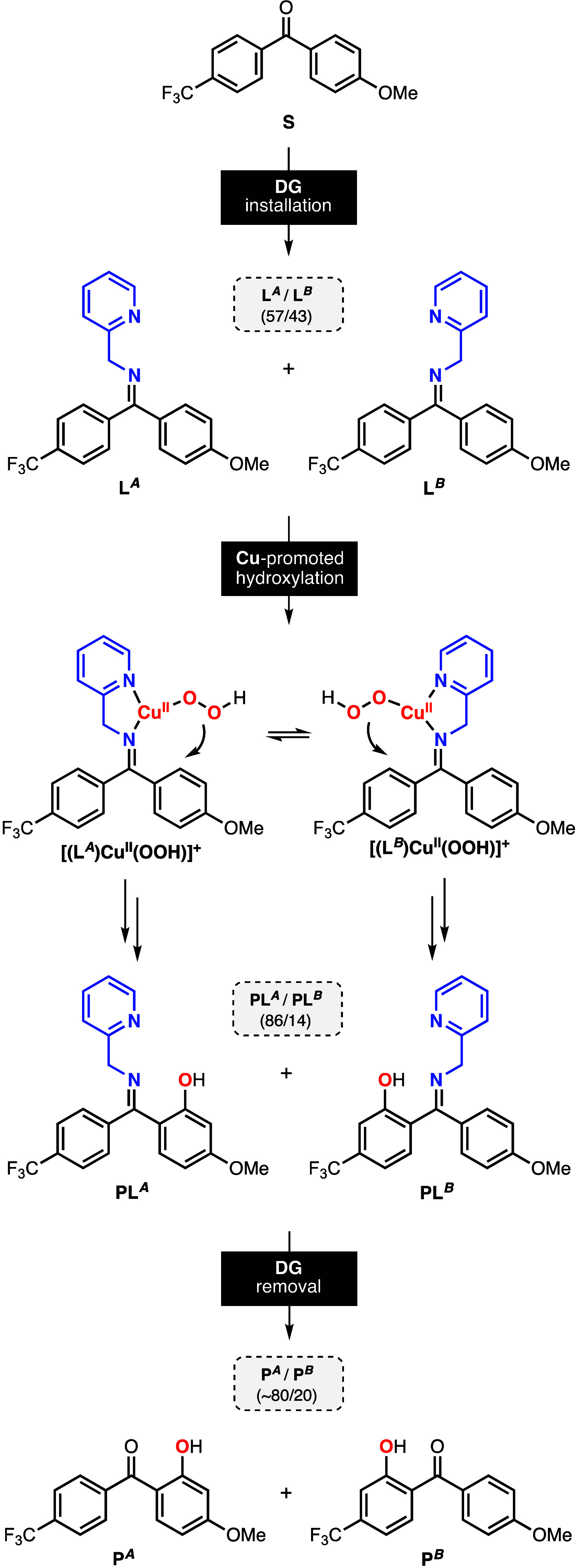
Cu-directed hydroxylation of 4-methoxy-4′-trifluoromethanebenzophenone using Cu, H_2_O_2_, and 2-picolylamine as directing groups.

The regioselectivity of Cu-promoted hydroxylation reactions of unsymmetrical ketones using imines as directing groups usually relies on the formation of a sole imine isomer, leading to the formation of only one hydroxylation product.^12,16,17^ We have recently reported that the installation of 2-picolyamine in unsymmetrical benzophenones produces two imine isomers that, upon being exposed to Cu and H_2_O_2_, produce two hydroxylation products.^15^ Interestingly, the ratio of the two hydroxylation products did not match the ratio of the imine isomers of the substrate–ligands. For example, 4-methoxy-4′-trifluoromethylbenzophenone and 2-picolylamine produced two imine isomers (L^A^ and L^B^ in Figure 3) with one of the isomers slightly favored (L^A^/L^B^: 57/43). However, the reaction with Cu and H_2_O_2_ led to a high selectivity for the oxidation of the arene ring with the electron-donating substituent (PL^A^/PL^B^: 86/14). Our mechanistic studies suggested the formation of an electrophilic Cu^II^OOH right before the rate-determining step of the reaction, favoring the oxidation of the electron-rich arene ring (e.g., MeO-substituted).

**Figure 4:**
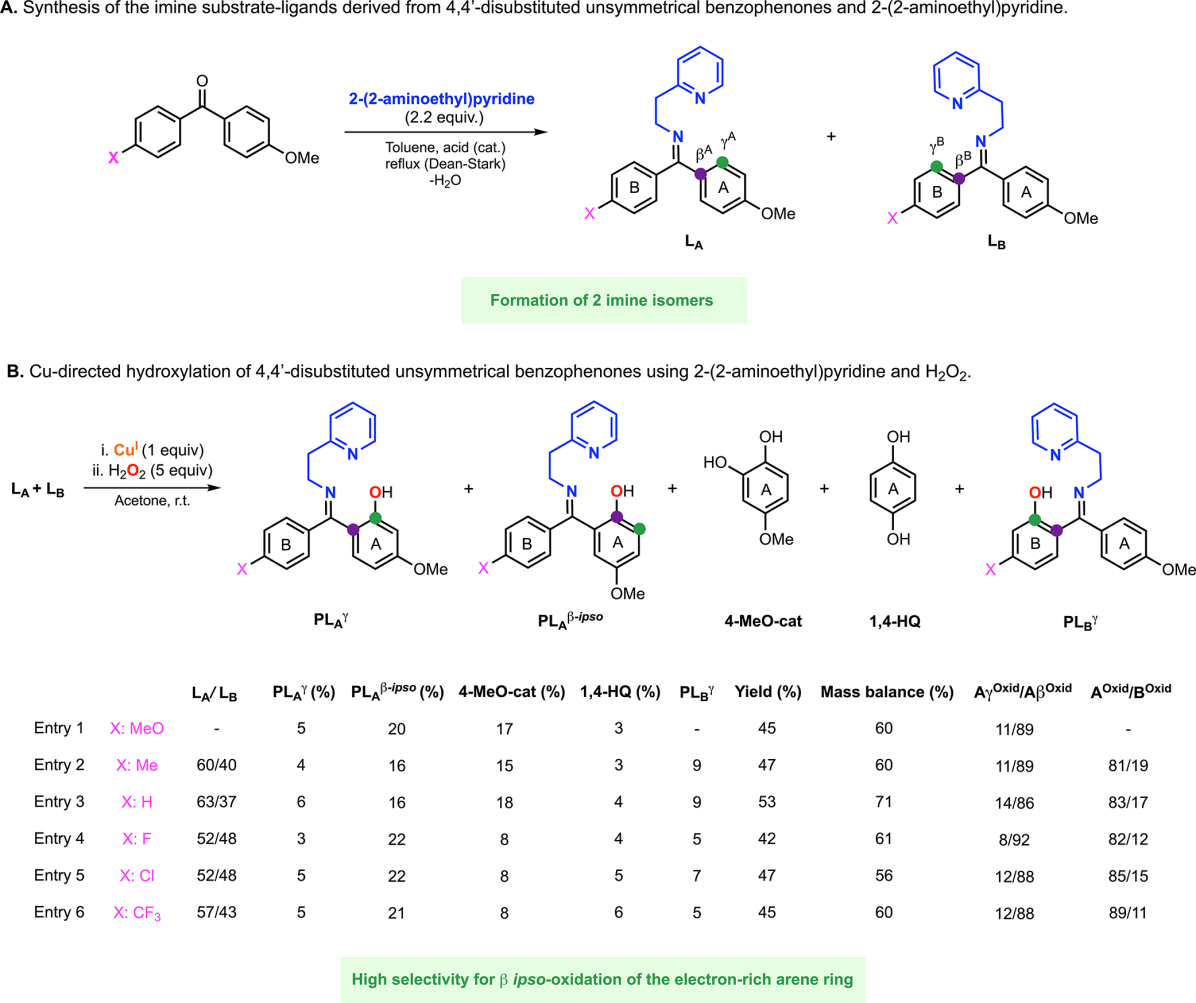
(A) Synthesis of the imine substrate–ligands derived from unsymmetrical benzophenones and 2-(2-aminoethyl)pyridine. (B) Hydroxylation of unsymmetrical benzophenones using Cu, H_2_O_2_, and 2-(2-aminoethyl)pyridine as the directing groups.

A similar behavior was observed in the Cu-directed hydroxylation of unsymmetrical 4-methoxy-4′-substituted benzophenones with 2-(2-aminoethyl)pyridine (Figure 4). The installation of the DG produced two imine isomers, in which one of the isomers was slightly favored (L^A^/L^B^ ≈ 55/45). As expected, the Cu-promoted hydroxylation of these systems led to the formation of an array of oxidation products, including the products derived from the γ-C–H hydroxylation of both arene rings (PL_A_^γ^ and PL_B_^γ^) and the products derived from β *ipso*-oxidation (PL_A_^β-ipso^, 4-MeO-cat, and 1,4-HQ; see Figure 4B). For all of the substrates analyzed, we observed high selectivity for the oxidation of the arene ring containing the MeO substituent (A^Oxid^/B^Oxid^ ratio), which increased when the other substituent varied from electron-donating (e.g., entry 2 in Figure 4B, A^Oxid^/B^Oxid^: 81/19) to electron-withdrawing (e.g., entry 6 in Figure 4B, A^Oxid^/B^Oxid^: 89/11). As in the hydroxylation of 4,4′-dimethoxybenzophenone, we observed high regioselectivity for β *ipso*-oxidation for all of the unsymmetrical benzophenones analyzed (Aγ^Oxid^/Aβ^Oxid^′ ≈ 10/90), with slight variations on the formation of catechol and hydroquinone.

### Proposed Mechanism

**Figure 5:**
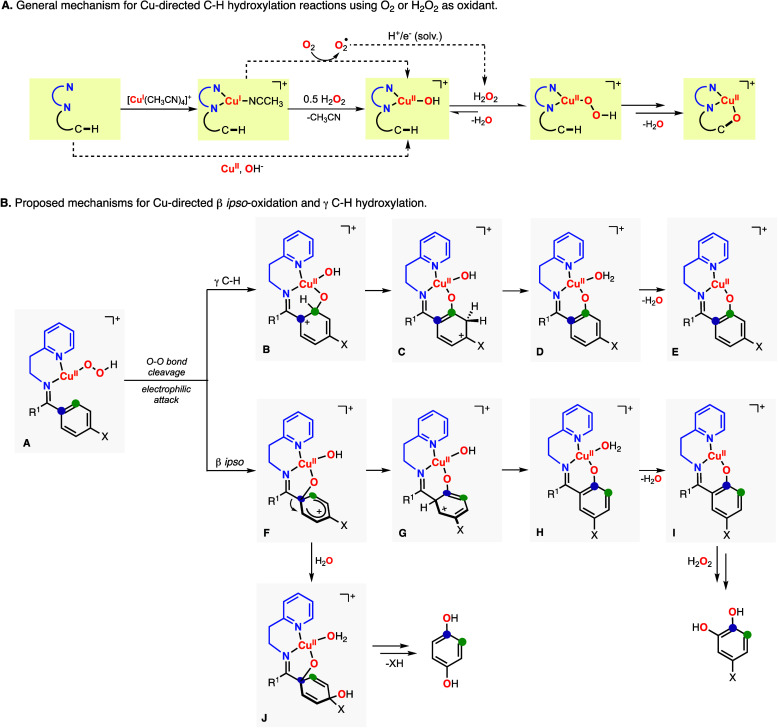
(A) General mechanism for the formation of mononuclear [LCuOOH]^+^ species using Cu^I^/O_2_, Cu^I^/H_2_O_2_, or Cu^II^/^–^OH/H_2_O_2_ before intramolecular substrate oxidation. (B) Proposed mechanisms for the Cu-promoted β *ipso*-oxidation and γ C–H hydroxylation for the substrate–ligands derived from substituted benzophenones and 2-(2-aminoethyl)pyridine.

In our previous publications, we reported the mechanism by which Cu and natural oxidants (O_2_ and H_2_O_2_) promote the hydroxylation of imine substrate–ligands (see Figure 5A).^12−15^ Our mechanistic evidence suggested the formation of a mononuclear Cu^II^OOH species before the rate-determining step of the reaction. This putative cupric hydroperoxide species can be formed under various reaction conditions by combining (i) Cu^I^ and O_2_ (solvent provides the protons and electrons to reduce O_2_ to H_2_O_2_); (ii) Cu^I^ and H_2_O_2_; and (iii) Cu^II^, hydroxide, and H_2_O_2_.

For the Cu-promoted oxidations reported herein, we propose the mechanism shown in Figure 5B, which is supported by the mechanistic evidence provided in the next sections. Our data support the formation of a mononuclear Cu^II^OOH species before the rate-determining step of the reaction (species A in Figure 5B). Depending on the arene substitution, the Cu^II^OOH intermediate can trigger γ-C–H hydroxylation and/or β *ipso*-oxidation. A concerted O–O bond cleavage electrophilic attack of the arene ring is proposed to occur during the rate-determining step of the oxidation, which can lead to γ-C–H hydroxylation (species B–D in Figure 5B). Alternatively, the O-O bond cleavage event can also lead to β *ipso*-attack of the arene ring (species F in Figure 5B). Rearrangement of the resulting carbocation intermediate (i.e., C–C bond cleavage and formation of a new C–C bond to produce species G in Figure 5B) followed by a hydride shift leads to the final β *ipso*-hydroxylation cupric product (species H and I in Figure 5B).

The formation of catechol is proposed to occur via overoxidation of the cupric β *ipso*-hydroxylation product with excess H_2_O_2_ present in solution (see more details in sections below). The formation of hydroquinone is believed to occur via nucleophilic attack of water (present in solution) on intermediate F to produce a metastable species that releases methanol before undergoing C–C cleavage to form hydroquinone (see species J in Figure 5B).

### Mechanistic Studies: Evidence of the Formation of Catechol and Hydroquinone

**Figure 6:**
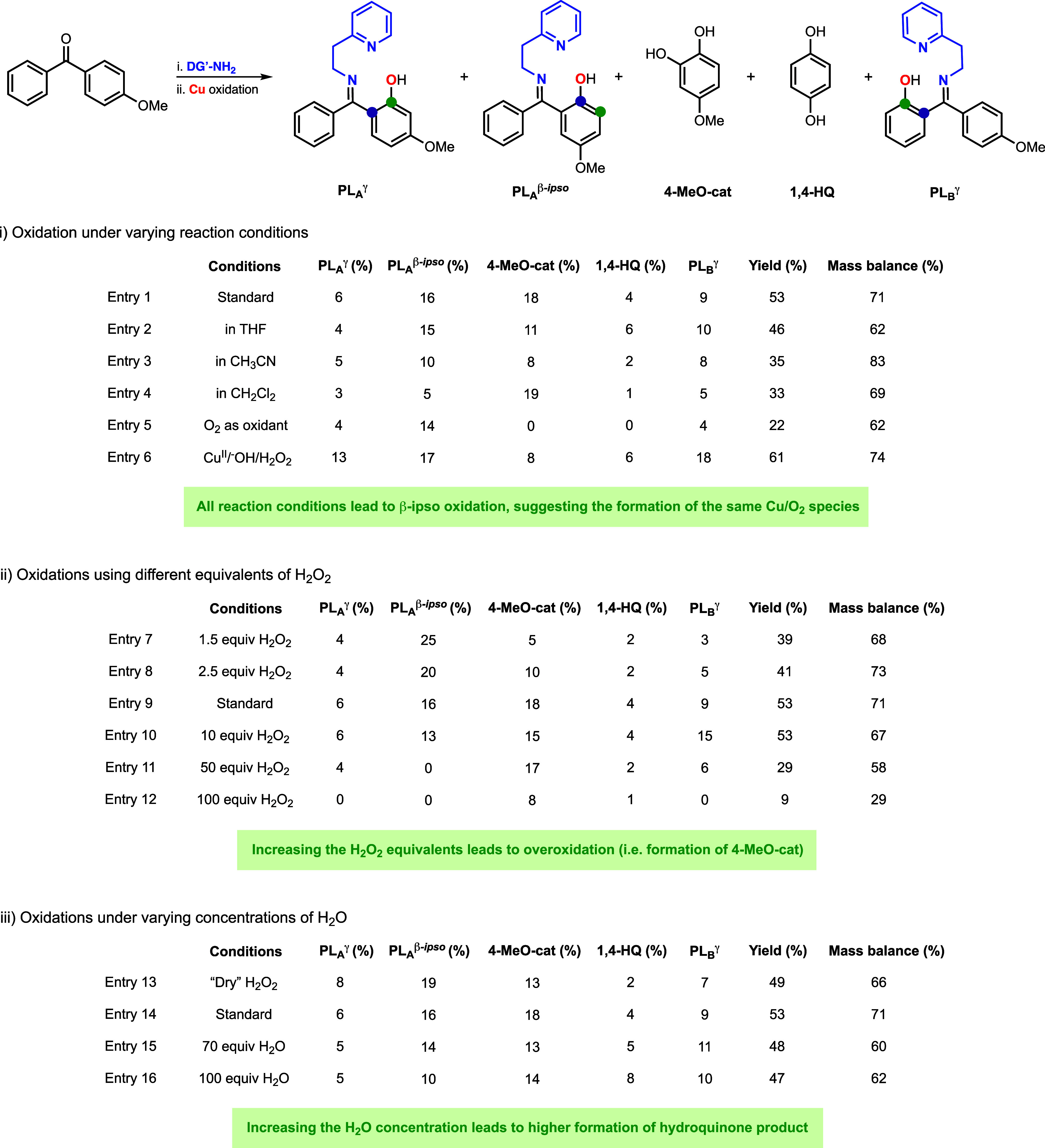
Cu-directed oxidation of the substrate–ligand derived from 4-methoxy-benzophenone and 2-(2-aminoethyl)pyridine under varying reaction conditions (See the S.I. for further details.)

The Cu-directed hydroxylation of the substrate–ligand derived from 4-methoxy-benzophenone and 2-(2-aminoethyl)pyridine was carried out under different reaction conditions (Figure 6). In the first set of experiments, we varied the solvent (THF, CH_3_CN, CH_2_Cl_2_) and the oxidant (Cu^I^/O_2_ and Cu^II^/^–^OH/H_2_O_2_; see Figure 6(i)). When compared to our standard conditions (1 equiv of Cu^I^ and 5 equiv of H_2_O_2_ in acetone), we observed the formation of the products derived from γ-C–H hydroxylation (PL_A_^γ^ and PL_B_^γ^) and β *ipso*-oxidation (PL_A_^β-*ipso*^) in all cases, suggesting the generation of a common reaction intermediate under all tested reaction conditions (mononuclear [LCu^II^OOH]^+^). In the oxidations performed with Cu^I^ and O_2_, we observed lower hydroxylation yields. Interestingly, these reaction conditions did not lead to the formation of catechol and hydroquinone. We believe that the use of Cu^I^/O_2_ allowed for generating substoichiometric amounts of H_2_O_2_ (leading to lower hydroxylation yields and preventing overoxidation to catechol) and minimal formation of water (precluding hydroquinone formation).

In the second set of experiments, we varied the equivalents of H_2_O_2_ utilized (Figure 6(ii)). When compared to our standard conditions (1 equiv of Cu^I^ and 5 equiv of H_2_O_2_ in acetone), the reactions with lower amounts of H_2_O_2_ (1.5 equiv and 2.5 equiv of H_2_O_2_) led to higher PL_A_^β-*ipso*^ formation (20–25% vs 16%) and a lower generation of the catechol product (5–10% vs 18%), suggesting that 4-MeO-cat is formed via overoxidation of PL_A_^β-*ipso*^. In fact, increasing the H_2_O_2_ equivalents (from 1.5 to 5 equiv) led to a higher formation of PL_A_^γ^ and PL_B_^γ^ and a decrease in PL_A_^β-*ipso*^, indicating that the catechol is not derived from PL_A_^γ^ and PL_B_^γ^ but from the overoxidation of PL_A_^β-*ipso*^. Further increases in the H_2_O_2_ equivalents (10, 50, and 100 equiv) led to a substantial decrease of the mass balance of the reactions, in which the catechol was the main reaction product, reinforcing the idea that excess amounts of H_2_O_2_ led to overoxidation.

In the third set of experiments, we analyzed the effect of water on Cu-promoted hydroxylation (Figure 6, (iii). In order to carry out the hydroxylation reactions in the absence of water, we utilized a dry source of H_2_O_2_ (*o*-Tol_3_P=O·H_2_O_2_)_2_^18^), which lowered the hydroquinone produced (from 4 to 2%). We also performed the hydroxylation reactions using aqueous H_2_O_2_ (5 equiv) in the presence of additional equivalents of H_2_O (70 and 100 equiv), which led to a substantial increase in the hydroquinone produced (from 4 to 8%). Higher concentrations of water also led to a decrease in the PL_A_^β-*ipso*^ and catechol formed, suggesting that water might prevent to some extent the formation of PL_A_^β-*ipso*^ and catechol, as shown in the proposed mechanism (see Figure 5).

**Figure 7:**
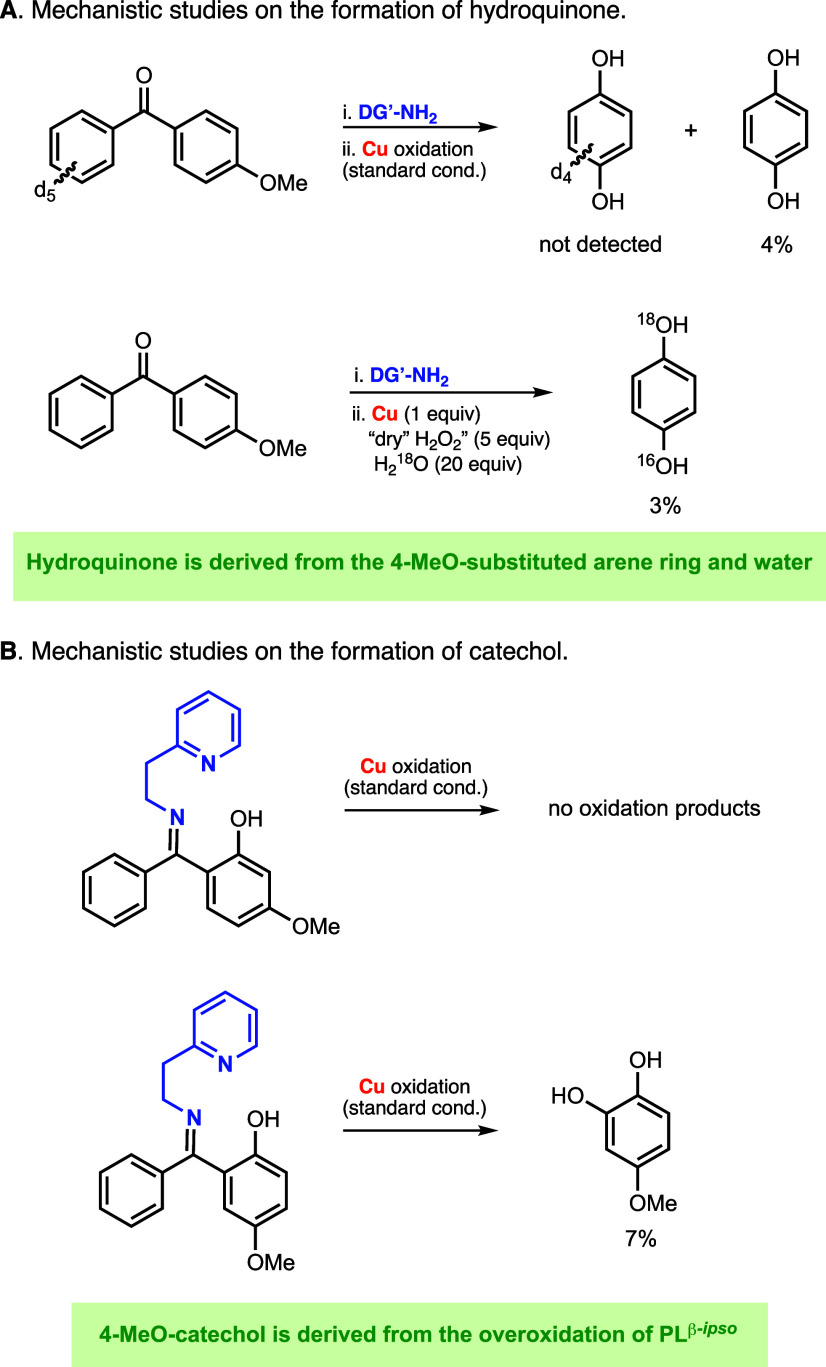
Mechanistic experiments on the formation of hydroquinone (A) and catechol (B). See the S.I. for further details.

Additional evidence of the mechanism by which the hydroquinone byproduct is formed was obtained in the Cu-directed hydroxylation of the substrate–ligand derived from 4-MeO-*d*_5_-benzophenone and 2-(2-aminoethyl)pyridine (Figure 7A). ^1^H NMR analysis of the hydroxylation reaction under our standard reaction conditions (1 equiv of Cu^I^ and 5 equiv of H_2_O_2_ in acetone) confirmed the formation of unlabeled hydroquinone with yields analogous to the ones observed in the hydroxylation of the parent nondeuterated substrate–ligand (∼4%). (Note that the major product of this oxidation was the *d*_5_-PL_A_^β-*ipso*^. See the S.I. for further details.) GC-MS analysis of the reaction indicated that only unlabeled hydroquinone was formed (i.e., no *d*_4_-hydroquinone was detected), which suggests that the hydroquinone product is derived from the oxidation of the phenyl ring containing the MeO substituent (see the S.I. for further details on the analysis). Additionally, no deuterated 4-MeO-catechol was observed, confirming that the catechol product is also derived from the MeO-substituted aryl ring.

To provide further evidence that the presence of water is causing the formation of the hydroquinone product, we carried out the oxidation of the substrate–ligand derived from 4-methoxybenzophenone and 2-(2-aminoethyl)pyridine using 1 equiv of Cu^I^ and 5 equiv of “dry” H_2_O_2_ in the presence of 20 equiv of labeled H_2_^18^O (Figure 7A). GC-MS analysis of the reaction indicated the formation of ^16^O,^18^O-hydroquinone, confirming the incorporation of water, in agreement with the proposed mechanism. GC-MS analysis also indicated that the 4-MeO-catechol, *ipso*-hydroxylation, and γ-hydroxylation products did not contain labeled ^18^O, suggesting that the O atoms incorporated into these compounds are derived from H_2_O_2_.

To corroborate that the catechol product was formed via overoxidation of PL_A_^β-*ipso*^ and not PL_A_^γ^, we independently synthesized the PL_A_^β-*ipso*^ and PL_A_^γ^ systems depicted in Figure 7B (see the S.I. for details), and we analyzed the products derived from their oxidation under standard conditions. Oxidation of PL_A_^γ^ led to the recovery of the starting material without the formation of any oxidation products. Conversely, the oxidation of the independently synthesized PL_A_^β-*ipso*^ system led to the formation of catechol (see Figure 7), suggesting that this byproduct is derived from the overoxidation of PL_A_^β-*ipso*^. In the oxidation of PL_A_^β-*ipso*^ and PL_A_^γ^, we did not observe the formation of the hydroquinone product, which indicated that its generation did not derive from the overoxidation of PL_A_^γ^ or PL_A_^β-*ipso*^.

### Mechanistic Studies: Spectroscopic Characterization of the Species Formed in Cu-Promoted Hydroxylation Reactions and Kinetic Analysis

**Figure 8:**
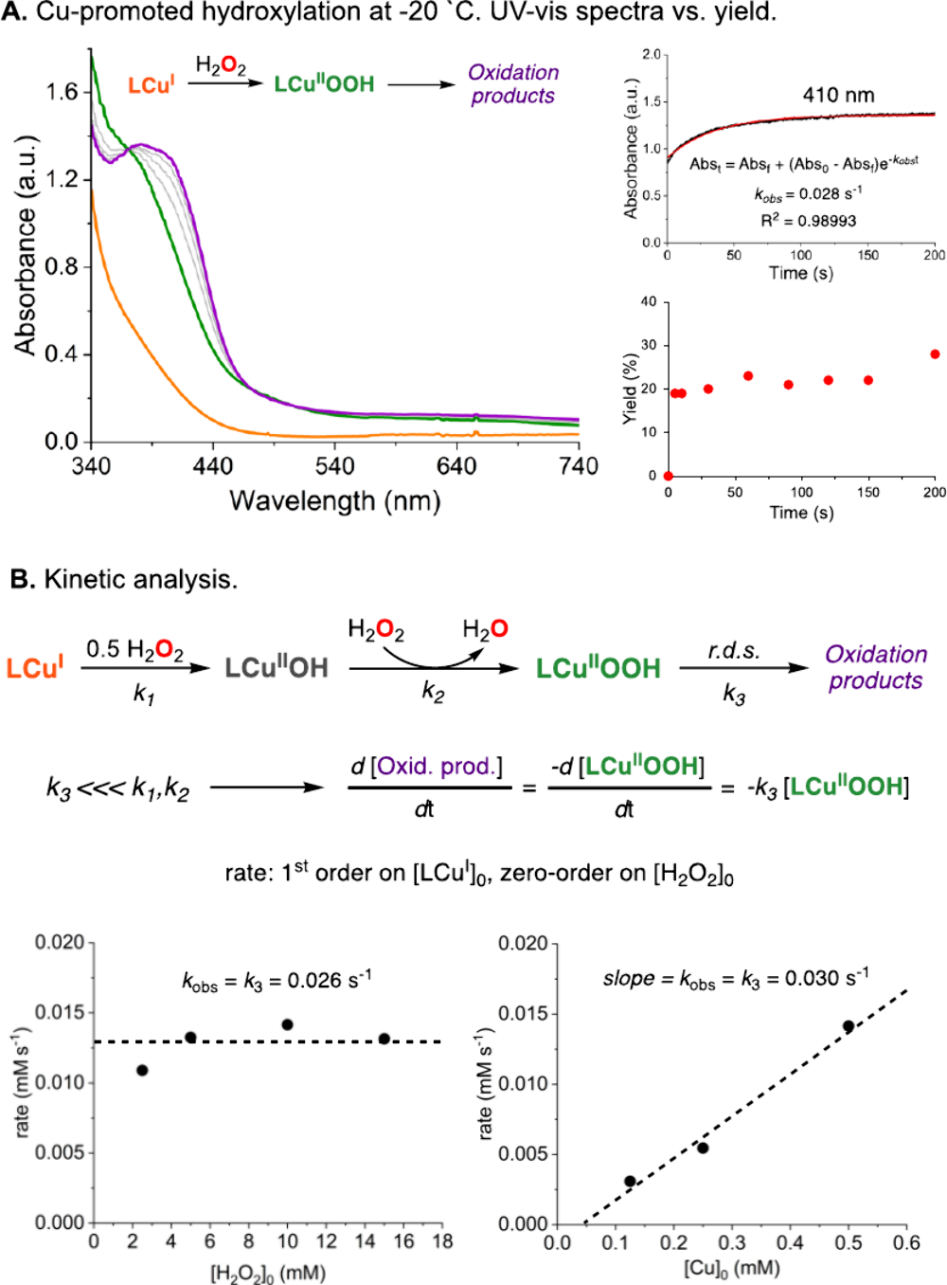
(A) UV–vis characterization of the species formed in the reaction between the substrate–ligand derived from 4,4′-methoxy-benzophenone and 2-(2-aminoethyl)pyridine, Cu^I^, and H_2_O_2_ ([Cu] = 0.5 mM, in acetone at −20 °C). The changes in the absorbance at 410 nm were compared to the oxidation yields (B). Kinetic analysis of the reaction among the same substrate–ligand, Cu^I^, and H_2_O_2_ under varying [Cu]_0_ and [H_2_O_2_]_0_. See the S.I. for further details.

The reaction between the substrate–ligand derived from 4,4′-dimethoxy-benzophenone and 2-(2-aminoethyl)pyridine, Cu^I^, and H_2_O_2_ in acetone was followed by UV–vis spectroscopy at cryogenic temperatures (Figure 8). As in our prior publications, the reaction between the [LCu^I^]^+^ complex and H_2_O_2_ produced an intermediate with UV–vis features centered at 370 nm (ε = 2400 M^–1^ cm^–1^), a peroxide-to-Cu(II) ligand-to-metal charge transfer (LMCT) transition characteristic of mononuclear [LCu^II^OOH]^+^ species.^12−14^ This metastable complex evolved to produce a species with UV–vis features centered at 410 nm, a transition observed in Cu^II^ complexes containing ligands derived from hydroxybenzophenone^13,14^ (Note that the addition of Cu^II^ to the independently synthesized PL_A_^β-*ipso*^ and PL_A_^γ^ systems depicted in Figure 7B led to UV–vis spectra similar to the spectra obtained in the above-mentioned LCu^I^/H_2_O_2_ reaction; see the S.I.).

The changes in absorbance at 370 and 410 nm indicated a two-step reaction in which the initial [LCu^I^]^+^ is quickly transformed to the [LCu^II^OOH]^+^ complex (0–5 s) before the rate-determining formation of the hydroxylation product (∼5 to 200 s). In a parallel experiment, we analyzed the evolution of the hydroxylation yields over time under the same reaction conditions (substrate–ligand/Cu^I^/H_2_O_2_ in acetone at −20 °C), and we found that the hydroxylation of the substrate–ligand occurred at short reaction times and only after the addition of H_2_O_2_ (∼5 to 200 s), in agreement with the UV–vis experiments.

Kinetic analysis of the reaction at different initial [LCu^I^]^+^ and H_2_O_2_ concentrations indicated that the hydroxylation rate was first order in copper and zeroth order in hydrogen peroxide (see Figure 8). These findings are in agreement with the proposed mechanism, which suggests that the intramolecular oxidation of the aromatic ring is not a one-step bimolecular process between Cu and H_2_O_2_ and that a putative [LCu^II^OOH]^+^ intermediate accumulates before the rds of the reaction. We hypothesize that the reaction between LCu^I^ and 0.5 equiv of H_2_O_2_ produces a [LCu^II^OH]^+^ intermediate that rapidly reacts with an additional molecule of H_2_O_2_ to generate the reactive [LCu^II^OOH]^+^ intermediate (see Figure 8). Additional evidence for this scenario was obtained in the reaction of the same substrate–ligand with Cu^II^, NMe_4_OH, and H_2_O_2_, which produced similar UV–vis spectral changes (i.e., fast generation of [LCu^II^OOH]^+^ before the formation of oxidation products) with similar kinetics (see the S.I.).

### Mechanistic Studies: DFT Calculations

**Figure 9:**
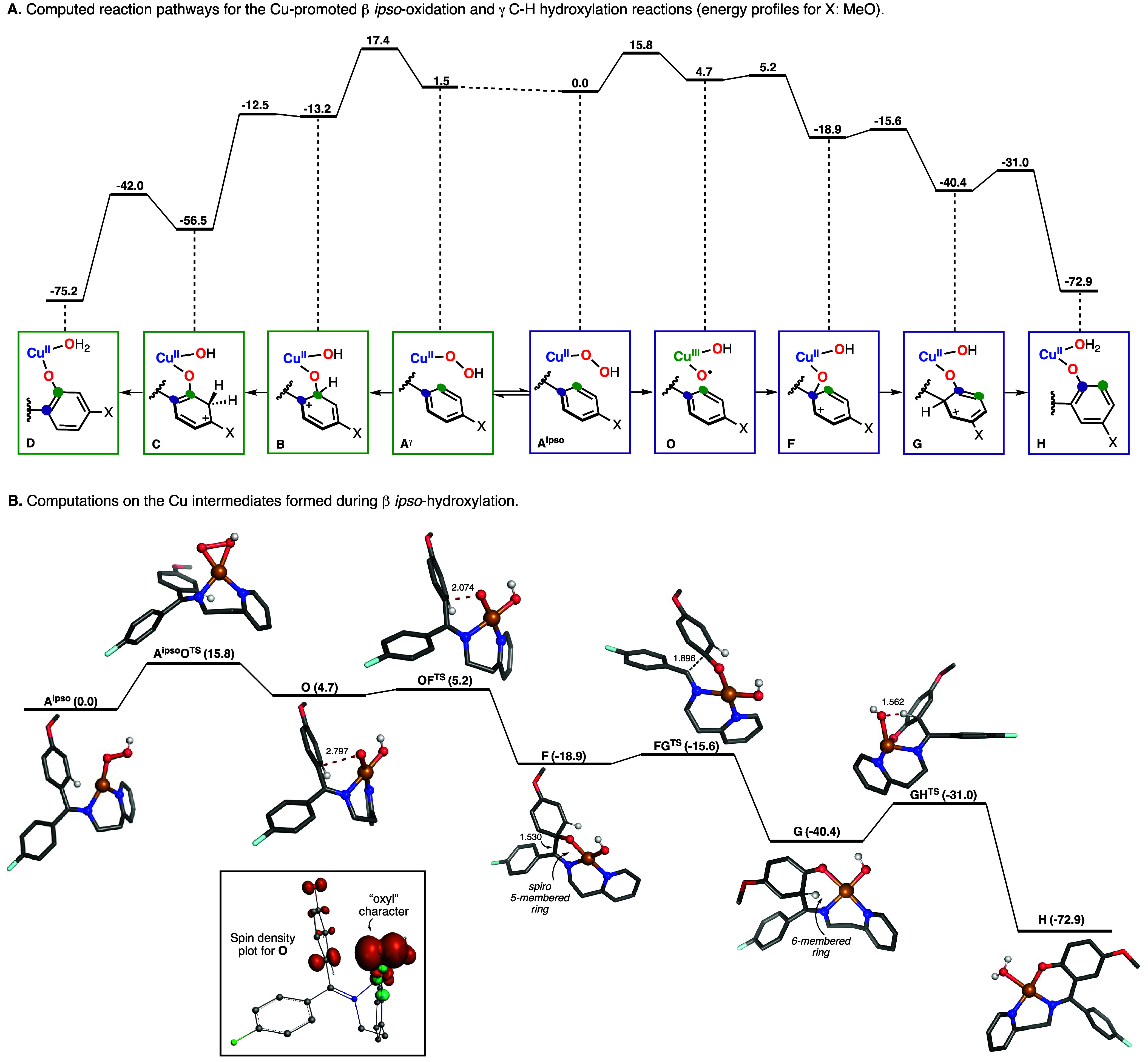
(A) Computed pathways for *ipso-* and γ-hydroxylation promoted by mononuclear CuOOH species. (B) Computed Cu intermediates formed during β *ipso*-hydroxylation. Note: the energies included in the text and in this figure are electronic energies in kcal/mol.

In order to gather further evidence of the mechanism(s) involved in the oxidation of the substrate–ligands derived from 2(2-aminoethyl)pyridine and substituted benzophenones, we performed DFT calculations. One of the first questions we aimed to answer was related to the mechanism by which β *ipso*-oxidation occurs. We also wondered if DFT calculations could explain the regioselectivity observed in the above-mentioned transformations. To answer both questions, we performed calculations on the Cu-promoted oxidation of the substrate–ligand derived from 4-methoxy-4′-fluorobenzophenone and 2-(2-aminoethyl)pyridine. We believed that this substrate would allow us to evaluate the mechanisms for β and γ oxidation but also analyzed the selectivity between the oxidation of the electron-rich ring (4-MeO substitution) and electron-withdrawing ring (4-F substitution). Our study was initiated by optimizing the Cu^II^OOH intermediate that is proposed to be formed before the rate-determining step of the sp^2^ oxidation. Given the fact that this substrate–ligand forms two imine stereoisomers and that two different orientations of the aryl ring are required before electrophilic attack of the corresponding β and γ carbon atoms, we optimized 4 Cu^II^OOH isomers. Our calculations indicate that the isomers have very similar electronic energies (see A^*ipso*^ and A^γ^ in Table 1). For each of the Cu^II^OOH isomers, we computed the mechanism by which O–O cleavage and C–O bond formation occur (i.e., 4 reaction pathways). The 2 Cu^II^OOH isomers that lead to γ C–H hydroxylation were found to undergo a concerted O–O bond cleavage and C–O bond formation in the rate-determining step (r.d.s.) followed by a hydride shift and rearomatization, a mechanism analogous to the one reported for the sp^2^ C–H hydroxylation with 2-picolylamine (see species A^γ^, B, C, and D in Figure 9A).^13,15^ For the 2 Cu^II^OOH isomers that lead to β *ipso*-oxidation, we computed that the r.d.s. involves O–O bond cleavage to produce a Cu-oxyl intermediate (species O in Figure 9) before aromatic electrophilic attack (species F in Figure 9). Species O could also attack at the C_γ_ position, but the corresponding barrier is ca. 6 kcal·mol^–1^ larger than the one following the β *ipso* pathway (see S.I.). A 1,2-rearrangement step involving C_α_–C_β_ cleavage and C_α_–C_γ_ bond formation (F to G in Figure 9) followed by rearomatization (G to H in Figure 9) led to the formation of the final hydroxylation product. For the 4 computed pathways, the energy of the r.d.s. for γ-hydroxylation was substantially higher than the energy of β *ipso*-oxidation, suggesting that 2(2-aminoethyl)pyridine favors β *ipso*-functionalization, which corroborates our experimental results.

**Table 1:** Computed Electronic Energies for the Cu-Directed Hydroxylation of the Substrate–Ligand Derived from 2(2-Aminoethyl)pyridine and 4-Methoxy-4′-fluorobenzophenone (in kcal/mol)

	**4-MeO**	**4-F**		**4-MeO**	**4-F**
**A**^***ipso***^	0.0	0.5	**A**^**γ**^	1.5	3.1
**A**^***ipso***^**O**^**TS**^	15.8	15.8	**A**^**γ**^**B**^**TS**^	17.4	18.8
**O**	4.7	4.5	**B**	–13.2	–10.3
**OF**^**TS**^	5.2	7.4	**BC**^**TS**^	–12.5	–9.2
**F**	–18.9	–12.8	**C**	–56.5	–49.4
**FG**^**TS**^	–15.6	–9.1	**CD**^**TS**^	–42.0	–37.6
**G**	–40.4	–40.1	**D**	–75.2	–75.1
**GH**^**TS**^	–31.0	–29.7			
**H**	–72.9	–73.4			

The Cu–O and O–O distances of the computed A^*ipso*^ intermediate (1.83 and 1.41 Å, respectively) are consistent with the distances measured for isolated end-on copper(II) hydroperoxide species.^19^ Our calculations suggest that the Cu^II^OOH core isomerizes from an end-on to a side-on binding mode during the rate-determining step of the reaction (A^*ipso*^O^TS^, see Figure 9B) prior to the O–O bond cleavage. The resulting species (intermediate O in Figure 9) is formulated as a Cu^III^-oxyl-hydroxo complex. The spin density plot (depicted in Figure 9B) indicates substantial electron density on the oxygen atom coordinated to the Cu ion, indicative of “oxyl” character. Moreover, d-orbital occupancy calculations^35^ indicate that the Cu ion in intermediate O is a d^8^ metal, in agreement with the Cu(III) formulation. (Details on the d-orbital occupancy calculations and spin density plots can be found in the S.I.) The formation of the high-valent intermediate O is followed by an *ipso* attack to the arene ring, a reaction step with a small activation energy. Interestingly, the barrier for the *ipso* attack on the arene containing the MeO substituent was substantially lower than the barrier for the *ipso* attack on the F-substituted system, in agreement with the electrophilic nature of the Cu-oxyl oxidant. The product derived from *ipso* oxidation (species F in Figure 9) can be described as a Wheland intermediate, in which the arene ring that has undergone electrophilic attack (nucleophile) showed a substantial loss of aromaticity (elongation of the C_β_–C_γ_ bonds from 1.40 Å in species O to 1.49 Å in species F).^20^

Undoubtedly, one of the most puzzling reaction steps of the *ipso* oxidation reported herein is the 1,2-rearrangement step (F to G in Figure 9) in which the C_α_–C_β_ bond is broken and a C_α_–C_γ_ bond is formed. This reaction step was also found to have a small barrier, and it was highly exergonic. We hypothesize that this is due to the strain release upon conversion of spiro intermediate F (in which the Cu forms a 5-membered ring, Cu–N_im_=C_α_–C_β_–O) to intermediate G (in which the Cu forms a 6-membered ring, Cu–N_im_=C_α_–C_γ_–C_β_–O). The conversion of spiro intermediate F to G entails elongation of the C_α_–C_β_ bond during the transition state (from 1.53 to 1.90 Å) and a slight shortening of the distance of the C_α_···C_γ_ bond to be formed (from 2.46 to 2.28 Å). Intermediate G is formulated as a Cu^II^OH species with the Cu ion in a square-planar geometry. To recover aromaticity, the CuOH core must accept a proton from the aromatic ring that has undergone *ipso* attack (also a Wheland intermediate) to produce the final Cu^II^ hydroxylation product. During the transition state of this proton transfer event, the square-planar CuOH complex will distort to a pseudotetrahedral geometry, shortening the distance between the hydroxide and the aromatic proton (from 3.80 to 1.56 Å).

### Contextualization of Our Findings

**Figure 10:**
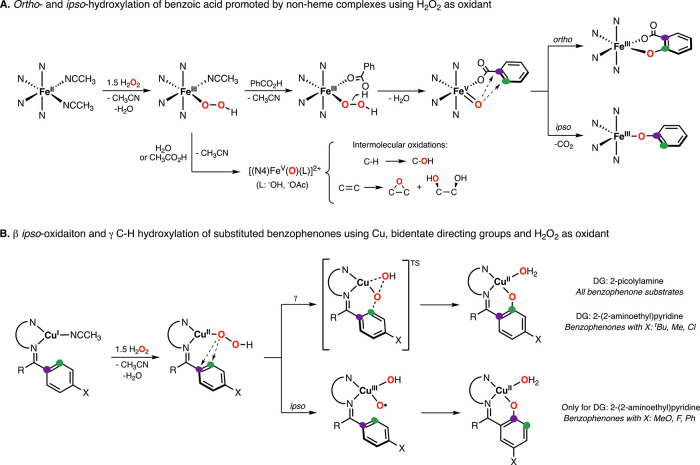
(A) Proposed mechanism for the intramolecular *ipso*- and *ortho*-hydroxylation of benzoic acids promoted by nonheme complexes using H_2_O_2_ as the oxidant. (B) Proposed mechanism(s) for the *ipso*- and γ-hydroxylation of benzophenone substrates using bidentate ligands as directing groups, Cu and H_2_O_2_.

Synthetic nonheme complexes bearing tetradentate ligands have been widely used to mimic the reactivity of iron-dependent monooxygenase and dioxygenase enzymes such as Rieske dioxygenases.^21,22^ For most of these enzymes, the iron center is proposed to react with oxygen and reducing equivalents (i.e., protons and electrons) in a stepwise fashion to produce reactive high-valent iron-oxo species (e.g., Fe^IV^(O) and Fe^V^(O)(OH)) before substrate oxidation.^23−25^ In model systems, the reaction of ferrous species with H_2_O_2_ can lead to the formation of Fe^III^(OOH) intermediates that undergo heterolytic O–O bond cleavage in the presence of water or carboxylic acids (Figure 10A).^26^ The resulting Fe^V^(O)(L) species (L: hydroxide, acetate), which have been spectroscopically characterized in selected cases,^27,28^ are proposed to promote regioselective C–H hydroxylation reactions, alkene epoxidation, and alkene *cis*-dihydroxylation.^29−31^ In 2009, Que, Rybak-Akimova, and co-workers reported that the reaction of nonheme complexes with H_2_O_2_ in the presence of benzoic acid led to *ortho*- and *ipso*-hydroxylation of the acid to generate the corresponding iron(III)-salicylate and iron(III)-phenolate products (Figure 10A).^32^ The authors proposed that the reaction of iron(II) complex with H_2_O_2_ produced a Fe^III^(OOH) species, which upon coordination of benzoic acid underwent heterolytic O–O bond cleavage to generate an iron(V)-oxo-benzoate intermediate before intramolecular sp^2^ hydroxylation. This mechanistic scenario is similar to the one we propose for the Cu-promoted intramolecular sp^2^ oxidations reported herein, in which the initial Cu^I^ complex reacts with H_2_O_2_ to form a mononuclear Cu^II^(OOH) species (step analogous to the formation of Fe^III^(OOH) from Fe^II^ and H_2_O_2_). The main difference between the Fe and Cu arises in the substrate oxidation step. While for iron the formation of a high-valent iron(V)-oxo complex prior to intramolecular benzoate oxidation is required,^33,34^ the mechanism of hydroxylation for Cu is highly dependent on the substrate oxidized and the directing group utilized. *ipso*-Hydroxylation, which is proposed to entail the formation of a high-valent Cu^III^(O·)(OH) species (analogous to the Fe^V^(O)(OH) formed in nonheme Fe/H_2_O_2_ chemistry), was observed only when 2-(2-aminoethyl)pyridine was utilized as DG and only for 4-MeO-, 4-F-, and F-Ph-substituted benzophenones. For the rest of the systems analyzed (including all substrate–ligands derived from benzophenone systems and 2-picolylamine and substrate–ligands derived from 4-^t^Bu-, 4-Me-, and 4-Cl-substituted benzophenones and 2-(2-aminoethyl)pyridine), our findings suggest that substrate hydroxylation occurs via a one-step concerted electrophilic attack.

**Figure 11:**
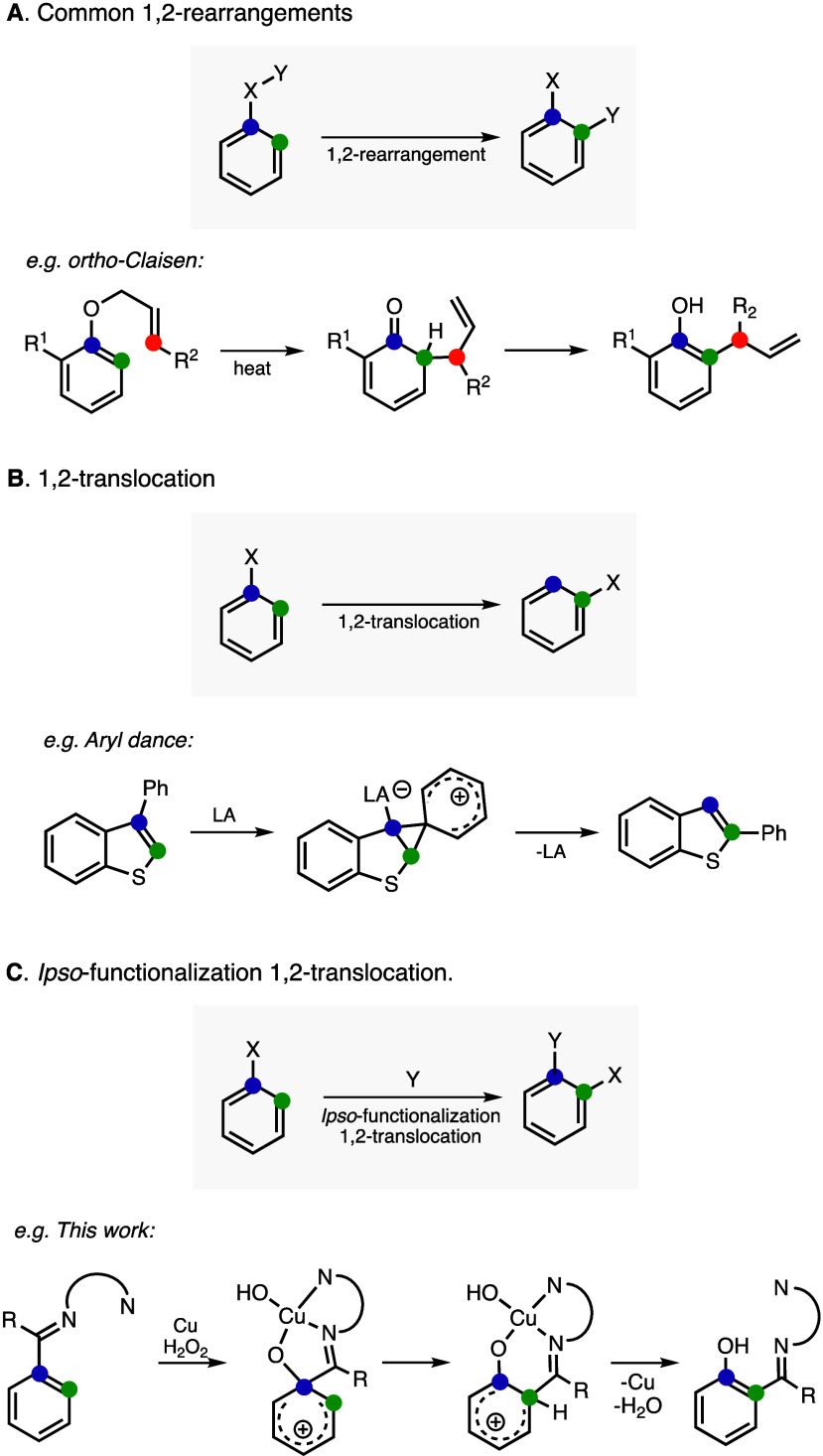
Comparison between the common aromatic 1,2-rearragements (A), 1,2-translocations (B), and *ipso*-functionalization 1,2-translocation reactions reported herein (C).

Aromatic rearrangement reactions are commonly used in organic synthesis because they allow the introduction of substituents into ring positions that are difficult to functionalize otherwise.^35−37^ Usually, aromatic rearrangements leave behind a fragment of the substituent at the original aromatic position. For example, in the *ortho*-Claisen rearrangement of allyl aryl ethers (see Figure 11A), *ortho*-C–H alkylation (which is proposed to occur via a [3,3] sigmatropic rearrangement) leads to the formation of an *ortho*-allylphenol product.^37^ Rearrangement reactions involving the translocation of the whole substituent are rare (Figure 11B).^38^ In a recent example, Yamaguchi and co-workers have reported the aromatic 1,2-translocation of aryl substituents in heteroles catalyzed by Lewis acids (named aryl dance reaction), which is proposed to occur via the formation of a Wheland intermediate.^39^

Despite the existence of numerous examples of aromatic 1,2-rearrangements (including several examples involving the formation of spiroindole intermediates^40−42^), we have not yet found any instance in which the electrophilic *ipso*-functionalization of a substituted arene substrate triggers 1,2-translocation (Figure 11C). The closest example was reported in 1970 by Tiecco and co-workers in which the *ipso*-addition of a 1-adamantly radical to thiophen-2,5-dicarbaldehyde formed 2-(1-adamantyl)thiophen-3,5-dicarbaldehyde, a product derived from 1,2-migration of the formyl group from the 2- to the 3-position.^43^ The scarcity of examples for this type of 1,2-rearrangement might be because, for most of *ipso*-functionalization reactions, one of the two *ipso*-substituents is eliminated, usually the H via hydride shift and subsequent rearomatization (as in the γ-C–H hydroxylation pathways shown in Figure 9). We believe that for this new class of rearrangement transformation, defined as the *ipso*-functionalization 1,2-translocation reaction, Cu chelation is critical for the observed vicinal migration of the iminyl substituent. It is also worth noting that the Cu-directed transformations reported herein (which can also be viewed as an *ipso*/*ortho* difunctionalization reaction^44,45^) convert 4-substituted benzophenones (e.g., 4,4′-(MeO)_2_-benzophenone) to unsymmetrical 2-hydroxy-5-substituted-benzophenones, products that are usually obtained via metal-promoted C_formyl_–H arylation of salicylaldehydes.^46^

## Conclusions

In this research article, we described the *ipso*-functionalization of benzophenone substrates using 2(2-aminoethyl)pyridine as the bidentate directing group, Cu, and H_2_O_2_, a reaction that involves 1,2-rearrangement of the iminyl group. The regioselectivity of this unprecedented transformation was highly dependent on the directing group utilized (i.e., 2-picolylamine led to γ-C–H hydroxylation) and on the substituents of the benzophenone substrate (highly favored with 4-MeO substituents). Our mechanistic investigations, which included labeling experiments, spectroscopic characterization of reaction intermediates, kinetics, and DFT calculations, suggest that the oxidation of the benzophenone substrate–ligands occurs via formation of the mononuclear Cu^II^OOH species before the rate-determining step. We also hypothesize that *ipso*-oxidation occurs via the O–O bond cleavage to produce a high-valent Cu^III^-oxyl-hydroxo species before electrophilic attack of the arene ring. Additional mechanistic studies (including DFT calculations) will aim to determine why *ipso*-hydroxylation is favored for some substituents (i.e.. MeO, Ph, F) and not for others (i.e., ^t^Bu, Me, Cl). Our future studies will also focus on performing *ipso*-oxidation on substrates beyond 4-substituted benzophenones (e.g., acetophenones, benzaldehydes, etc.) and broadening the reactivity scope of our Cu-promoted peroxygenase-like transformations such as Cu-directed epoxidations and/or *cis*-dihydroxylations of alkene, reactions that are typically promoted by high-valent metal-oxo-hydroxo species similar to the Cu^III^-oxyl-hydroxo intermediate proposed in this article but never observed for Cu.

## Experimental Section

### Materials

All reagents and solvents were purchased at the highest level of purity and used as received, except as noted. Solvents were purified and dried by passing them through an activated alumina purification system (mBRAUN SPS) or by conventional distillation techniques. The hydrogen peroxide adduct of phosphine oxide (*o*-Tol_3_P=O·H_2_O_2_)_2_ was synthesized using the synthetic protocol reported by J. Blümel and co-workers.^18^

***Caution!** Hydrogen peroxide and acetone (when mixed at high concentration) is a hazardous combination that can form various explosive peroxides.*

### Physical Methods

All NMR experiments were collected at 300 K on either a two-channel Bruker Avance III NMR instrument equipped with a Broad Band Inverse (BBI) probe or a Bruker NEO 500 NMR spectrometer equipped with the multinuclear BBO Prodigy cryoprobe. Both instruments operate at 500 MHz for ^1^H (125.7 MHz for ^13^C{^1^H}). The ^1^H NMR spectra are referenced to residual protio solvents (7.26 ppm for CDCl_3_ and 1.94 ppm for CD_3_CN), and the ^13^C{^1^H} NMR spectra are referenced to CDCl_3_ (77.2 ppm). Structural assignments were made with additional information from the COSY and NOESY experiments. ESI-MS high-resolution mass spectrometry was performed on a Thermo Scientific Exactive Plus EMR Orbitrap Mass Spectrometer in the Department of Chemistry at Carnegie Mellon University.

### Experimental Procedure for the Synthesis of Imine Substrate–Ligands

In an oven-dried flask, 2-(2-aminoethyl)pyridine (2.2 equiv) was added to the ketone substrate (9.85 mmol) and *p*-toluenesulfonic acid monohydrate (cat. 20 mg) in toluene (50 mL). The reaction mixture was refluxed under argon with a Dean–Stark apparatus until imine formation was completed. The reaction mixture was cooled to room temperature and diluted with diethyl ether (30 mL). The organic layer was washed with saturated ammonia chloride (50 mL × 2), saturated aqueous sodium bicarbonate (50 mL), and brine (50 mL) and dried with magnesium sulfate. The final product was isolated under a vacuum. The purity of the resulting imine substrate–ligands was analyzed by ^1^H NMR by adding a known amount of an internal standard (1,3,5-trimethoxybenzene).

### Experimental Procedure for the Hydroxylation of the Imine Substrate–Ligands

In the glovebox, 4 mL of acetone was added to an 8 mL vial containing 0.159 mmol of the imine substrate–ligand equipped with a stir bar. To the solution, 0.159 mmol of [Cu^I^(CH_3_CN)_4_](PF_6_) was added and allowed to react. The solution mixture was taken out of the glovebox, and 5 equiv of 30% H_2_O_2_ was added. After 30 min, the reaction was quenched using Na_2_EDTA (50 mL, pH = 4). The resulting mixture was extracted with EtOAc (50 mL × 3). The organic phases were separated, combined, dried over MgSO_4_, filtered, and dried under vacuum. The reaction products were dissolved in 1.4 mL of CDCl_3_ solution containing 27.1 mg of 1,3,5-trimethoxybenzene (internal standard). The reaction products were quantified by ^1^H NMR using integration signals that correspond to the starting material and products with the integration signal of the internal standard.

### Experimental Procedure for the Removal of the Directing Group

After hydroxylation, the products were dissolved in a round-bottomed flask, and 50 mL of EtOAc and 100 mL of 1 M HCl were added. The resulting mixture was stirred at room temperature for 30 min and was extracted with EtOAc (50 mL × 2). The organic phases were separated, combined, dried over MgSO_4_, filtered, and dried under vacuum. The reaction products were dissolved in 1.4 mL of CDCl_3_ solution containing 27.1 mg of 1,3,5-trimethoxybenzene (internal standard). The reaction products were quantified by ^1^H NMR using integration signals that correspond to the starting material and products with the integration signal of the internal standard.

### Calculation of the Ratio of Isomers for the Imine Substrate–Ligands, Hydroxylation Products, and Cleaved Hydroxylation Products

^1^H NMR, ^13^C NMR, and COSY and NOESY measurements were carried out to characterize the two isomers formed in the synthesis of the imine substrate–ligands. The ratio of the isomers was calculated using the average of the integration of CH_2_ peaks (between 3.1 and 3.9 ppm) and CH peaks (between 6.8 and 7.0 ppm). ^1^H NMR measurements were carried out to characterize the products derived from the hydroxylation of the imine substrate–ligands. The ratio of Aγ^Oxid^/Aβ^Oxid^ is calculated using the integration of CH peaks of the product derived from γ-C–H hydroxylation (PL_A_^γ^) and hydroxylation products derived from β *ipso*-hydroxylation (PL_A_^β*-ipso*^, 4-MeO-cat, and 1,4-dihydroquinone). The ratio of A^Oxid^/B^Oxid^ is calculated using the integration of CH peaks of products derived from the oxidation of the A ring (PL_A_^γ^, PL_A_^β*-ipso*^, 4-MeO-cat, and 1,4-dihydroquinone) and the oxidation product derived from the B ring (PL_B_^γ^). For each system, at least two hydroxylation reactions were analyzed and only slight variations in the reaction yield, mass balance (note that the mass balance includes the hydroxylation yields and starting material unreacted), and products ratio were observed. ^1^H NMR measurements were carried out to characterize the products derived from the cleavage of the directing group of the products derived from the hydroxylation of the imine substrate–ligands.

### UV–Vis Spectroscopy

UV–vis measurements were performed using a Hewlett-Packard 8454 diode array spectrophotometer with a 10 mm path quartz cell. The spectrometer was equipped with HP Chemstation software and a Unisoku cryostat for low-temperature experiments. *Sample preparation:* 2.8 mL of a solution of 0.5 mM substrate–ligand in acetone was placed in a 10 mm path quartz cell equipped with a stir bar and capped with a rubber septum. The solution was taken out of the glovebox and cooled to −20 °C. After cooling, 100 μL of an acetone solution containing 0.5 mM [Cu^I^(CH_3_CN)_4_](PF_6_) was added and the spectrum was recorded. After the addition of [Cu^I^(CH_3_CN)_4_](PF_6_), 100 μL of an acetone solution containing 10 mM of 30% H_2_O_2_ was added. (Note that the solution of H_2_O_2_ was deoxygenated by Ar/vacuum cycles before being injected into the Cu(I) complex.) The reaction spectral changes were recorded every 1 s for 3600 s.

### GC-MS Analysis

GC-MS analysis was performed on a Hewlett-Packard Agilent 6890-5973 GC-MS workstation. The GC column was a Restek fused silica capillary column (RTX-5). Helium was used as the carrier gas. The following conditions were used for all GC-MS analyses: injector temperature, 250 °C; initial temperature, 70 °C; temperature ramp, 10 °C/min; and final temperature, 290 °C. Samples were prepared by dissolving 0.2 mL of the NMR sample with 1 mL of ethyl acetate into a 2 mL vial for analysis.

### Computational Details

All DFT calculations were performed with the Amsterdam Density Functional (ADF)^47,48^ and QUILD^49^ programs. Molecular orbitals were expanded in an uncontracted set of Slater-type orbitals (STOs) of triple-ζ quality with double polarization functions (TZ2P).^50,51^ Core electrons were not treated explicitly during the geometry optimizations (frozen core approximation^48^). An auxiliary set of s, p, d, f, and g STOs were used to fit the molecular density and to represent the Coulomb and exchange potentials accurately for each SCF cycle.

Geometries were optimized with the QUILD^49^ program using adapted delocalized coordinates until the maximum gradient component was less than 10^–4^ a.u. Energies, gradients, and Hessians^52^ (for vibrational frequencies) were calculated using S12g,^53^ in all cases by including solvation effects through the COSMO^54^ dielectric continuum model with appropriate parameters for the solvent (acetone).^55^ To compute Gibbs free energies, all small frequencies were raised to 100 cm^–1^ in order to compensate for the breakdown of the harmonic oscillator model.^56,57^ Scalar relativistic corrections have been included self-consistently in all calculations by using the zeroth-order regular approximation (ZORA).^58^ Most S12g calculations were performed with a Becke grid^59,60^ of very good quality, except the frequencies which were computed with a Normal grid. All DFT calculations were performed by using the unrestricted Kohn–Sham scheme.

## Supporting Information Available

Experimental details, including characterization data, spectra, and DFT calculations (PDF)
Computational data, including xyz coordinate tables, .tgz files, and movies (gif files) of the computed pathways for *ipso-* and γ-hydroxylation promoted by mononuclear CuOOH species (ZIP)
