# Supplementary material for: Cu-Promoted ipso-Hydroxylation of sp2 Bonds with Concomitant Aromatic 1,2-Rearrangement Involving a Cu-oxyl-hydroxo Species
Source: Inorg Chem. 2024 Oct 18;63(43):20675–88. doi: 10.1021/acs.inorgchem.4c03304 (PMC11523237; doi:10.1021/acs.inorgchem.4c03304)
Supplement: Supplementary file 1 — ic4c03304_si_001.pdf [file ic4c03304_si_001.pdf]

# Supporting Information

# for

# Cu-promoted *ipso*-hydroxylation of sp$^2$ bonds with concomitant aromatic 1,2-rearrangement involving a Cu-oxyl-hydroxo species

Sunipa Goswami,† Karan Gill,† Xinyi Yin, † Marcel Swart, *,Ψ and Isaac Garcia-Bosch*,†

†*Department of Chemistry, Carnegie Mellon University, Pittsburgh, Pennsylvania 15213, United States.*

Ψ*University of Girona, Campus Montilivi (Ciències), IQCC, Girona, Spain; ICREA, Pg. Lluís Companys 23, 08010, Barcelona, Spain.*

*Corresponding authors**

*Email address:* igarciab@andrew.cmu.edu (Isaac Garcia Bosch) marcel.swart@udg.edu (Marcel Swart)

## Table of Contents

1. **Experimental section………………………………………………………………………..S3**
2. **Description of the general protocol for the synthesis and hydroxylation of the imine substrate-ligands……………………………………………………………………………….S4**
3. **Synthesis and hydroxylation of the symmetrical imine substrate-ligands……………..S7**
4. **Synthesis and hydroxylation of the unsymmetrical imine substrate-ligands…………S34**
5. **Synthesis and hydroxylation of the deuterated imine substrate-ligands………………S64**
6. **Labelled $H_2O$ reactions: GC-MS data……………………………………………………S71**
7. **Synthesis and oxidation of the product substrate-ligands………………………………S81**
8. **Summary and mass balance of Cu-directed hydroxylations…………………………….S88**
9. **UV-vis…………………………………………………………………………………………S90**
10. **Evaluation of reaction yields over time.……………………………………………….S103**
11. **DFT Studies…………………...……………………………………………………………...S104**
12. **$^{13}$C-NMR spectra of ligand scaffolds…………………………………………………...S113**
13. **References………………………………………………………………………………S127**

## 1. Experimental Section

**Physical methods and materials reagents:** All reagents and solvents were purchased at the highest level of purity and used as received except as noted. All substrate-containing ligands were synthesized as previously reported.[1,2] Hydrogen peroxide adduct of phosphine oxide (*o*-$Tol_3P{=}O{\cdot}H_2O_2)_2$ was synthesized using the synthetic protocol reported by J. Blümel and coworkers[3]. Solvents were purified and dried by passing through an activated alumina purification system (mBRAUN SPS) or by conventional distillation techniques.

**Glovebox:** The synthesis of copper complexes and preparation of some NMR samples was carried out under anaerobic conditions in an mBRAUN MB-Unilab Pro SP Glovebox system.

**UV-vis** measurements were carried out by using a Hewlett Packard 8454 diode array spectrophotometer with a 10 mm path quartz cell. The spectrometer was equipped with HP Chemstation software and a Unisoku cryostat for low-temperature experiments.

**All NMR experiments** were collected at 300 K on either a two-channel Bruker Avance III NMR instrument equipped with a Broad Band Inverse (BBI) probe, or a Bruker NEO 500 NMR spectrometer equipped with the multinuclear BBO Prodigy cryoprobe. Both instruments operate at 500 MHz for $^1$H (125.7 MHz for $^{13}$C{$^1$H}). The $^1$H NMR spectra are referenced to residual protio solvents (7.26 ppm for $CDCl_3$ and 1.94 ppm for $CD_3CN$) and the $^{13}$C{$^1$H} NMR spectra are referenced to $CDCl_3$ (77.2 ppm).

**ESI-MS:** High-resolution mass spectrometry was performed on Thermo Scientific Exactive Plus EMR Orbitrap Mass Spectrometer in the Department of Chemistry at Carnegie Mellon University.

**GC-MS:** GC-MS analysis was performed on a Hewlett-Packard Agilent 6890-5973 GC-MS workstation. The GC column was a Restek fused silica capillary column (RTX-5). Helium was used as the carrier gas. The following conditions were used for all GC-MS analyses: injector temperature, 250 °C; initial temperature, 70 °C; temperature ramp, 10 °C/min; final temperature, 290 °C.

## 2. Description of the general protocol for the synthesis and hydroxylation of the imine substrate-ligands

### 2.1. General procedure for the synthesis of imine substrate-ligands

In an oven-dried flask, 2-(2-aminoethyl)pyridine (2.2 equiv) was added to ketone (9.85 mmol) and p-toluenesulfonic acid monohydrate (cat. 20 mg) in toluene (50 mL). The reaction mixture was refluxed under argon with a Dean-Stark apparatus until imine formation was complete (24 h). The reaction was cooled to room temperature and diluted with diethyl ether (30 mL). The organic layer was washed with saturated ammonia chloride (50 mL x 2), saturated aqueous sodium bicarbonate (50 mL), brine (50 mL), and dried with magnesium sulfate. The final product was isolated under vacuum. The purity of the synthesized ligands was analyzed by $^1$H-NMR by adding a known amount of internal standard (1,3,5-trimethoxybenzene).

### 2.2. Experimental procedure for in situ oxidation of copper mediated oxidations using $H_2O_2$:

In the glovebox, 4 mL of acetone was added to an 8-mL vial containing 0.159 mmol of the imine substrate-ligand equipped with a stir bar. To the solution, 0.159 mmol of $[Cu^I(CH_3CN)_4](PF_6)$ was added and allowed to react. The solution mixture was taken out of the glovebox and 5 equiv of 30% $H_2O_2$ was added. After 30 minutes, the reaction was quenched using $Na_2EDTA$ (50 mL, pH = 4), and EtOAc (50 mL x 3). The organic phases were separated, combined, dried over $MgSO_4$, filtered, and dried under vacuum. The organic product was dissolved in 1.4 mL of $CDCl_3$ solution containing 27.1 mg of 1,3,5-trimethoxybenzene (internal standard). The reaction products were quantified by $^1$H-NMR using integration signals that correspond to the starting material and products with the integration signal of the internal standard.

### 2.3. Experimental procedure for in situ oxidation of copper-mediated oxidations using $O_2$:

In the glovebox, 4 mL of acetone was added to an 8-mL vial containing 0.159 mmol of the imine substrate-ligand equipped with a stir bar. To the solution, 0.159 mmol of $[Cu^I(CH_3CN)_4](PF_6)$ was added and allowed to react. Outside the glovebox at room temperature, oxygen was bubbled into the solution for 10 seconds and allowed to react for 12 h at 50 $^oC$. After 12 h, the acetone solution was quenched with $Na_2EDTA$ (50 mL, pH = 4) and EtOAc (50 mL x 3). The organic layers were separated, combined, dried with $MgSO_4$, filtered, and dried under vacuum. The organic product was dissolved in 1.4 mL of $CDCl_3$ solution containing 27.1 mg of 1,3,5-trimethoxybenzene (internal standard). The reaction products were quantified by $^1$H-NMR using integration signals

that correspond to the starting material and products with the integration signal of the internal standard.

### 2.4. Experimental procedure for in situ oxidation of copper-mediated C-H oxidation – Solvent Effect:

In the glovebox, 0.159 mmol [$Cu^{I}$ ($CH_3CN)_4$]($PF_6$) and 0.159 mmol of $^{4MeO4'H}L_{(A+B)}$ were added to an 8 mL reaction vial followed by the solvent of choice (4 mL). The solution mixture was taken out of the glovebox and 5 equiv of 30% $H_2O_2$ was added. After 30 minutes, the reaction was quenched using $Na_2EDTA$ (50 mL, pH = 4), and EtOAc (50 mL x 3). The organic phases were separated, combined, dried over $MgSO_4$, filtered, and dried under vacuum. The organic product was dissolved in 1.4 mL of $CDCl_3$ solution containing 27.1 mg of 1,3,5-trimethoxybenzene (internal standard). The reaction products were quantified by $^1$H-NMR using integration signals that correspond to the starting material and products with the integration signal of the internal standard.

### 2.5. Experimental procedure for in situ oxidation of copper-mediated C-H oxidation – Cu (II) + $H_2O_2$ + $Me_4NOH$ conditions:

In the glovebox, 4 mL of acetone was added to an 8-mL vial containing 0.159 mmol of the imine substrate-ligand equipped with a stir bar. To the solution, 0.159 mmol of [$Cu^{II}(NO_3)_2$. $3H_2O$] was added and allowed to react. The solution mixture was taken out of the glove box and 1 equivalent of $Me_4NOH$•$5H_2O$ was added followed by the addition of 5 equiv of 30% $H_2O_2$. After 30 minutes, the reaction was quenched using $Na_2EDTA$ (50 mL, pH = 4), and EtOAc (50 mL x 3). The organic phases were separated, combined, dried over $MgSO_4$, filtered, and dried under vacuum. The organic product was dissolved in 1.4 mL of $CDCl_3$ solution containing 27.1 mg of 1,3,5-trimethoxybenzene (internal standard). The reaction products were quantified by $^1$H-NMR using integration signals that correspond to the starting material and products with the integration signal of the internal standard.

### 2.6. Experimental procedure for in situ oxidation of copper-mediated oxidations using anhydrous $H_2O_2$:

In the glovebox, 2 mL of acetone was added to an 8-mL vial containing 0.08 mmol of the imine substrate-ligand equipped with a stir bar. To the solution, 0.08 mmol of [$Cu^{I}(CH_3CN)_4$]($PF_6$) was added and allowed to react. Then to the solution mixture 2.5 equiv of anhydrous $H_2O_2$[3] was added. After 30 minutes, the reaction was quenched using $Na_2EDTA$ (25 mL, pH = 4), and EtOAc

(25 mL x 3). The organic phases were separated, combined, dried over $MgSO_4$, filtered, and dried under vacuum. The organic product was dissolved in 1.4 mL of $CDCl_3$ solution containing 13.6 mg of 1,3,5-trimethoxybenzene (internal standard). The reaction products were quantified by $^1$H-NMR using integration signals that correspond to the starting material and products with the integration signal of the internal standard.

## 3. Synthesis and hydroxylation of the symmetrical imine substrate-ligands

### 3.1. Synthesis of L1

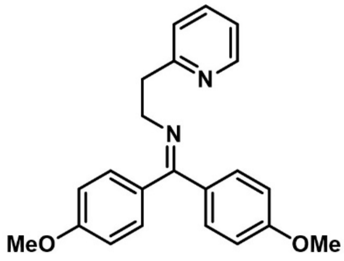

**L1**

In an oven-dried flask, 2-(2-pyridyl)ethylamine (2.60 mL, 21.7 mmol, 2.2 equiv) was added to 4,4'-dimethoxybenzophenone (2.38 g, 9.85 mmol) and p-toluenesulfonic acid monohydrate (cat. 20 mg, 1.2 mol%) in toluene (50 mL). The reaction mixture was refluxed under argon with a Dean-Stark apparatus until imine formation was complete (48 h). The reaction was cooled to room temperature and diluted with diethyl ether (30 mL). The organic layer was washed with saturated ammonia chloride (50 mL x 2), saturated aqueous sodium bicarbonate (50 mL), brine (50 mL), and dried with magnesium sulfate. The final product isolated was an orangish yellow solid (81% yield, 97% pure).

$^{1}$H NMR (500 MHz, $CDCl_3$): δ 8.49 (d, 1H), 7.56 (dt, 1H), 7.53 (d, 2H), 7.19 (t, 1H), 7.08 (m, 1H), 6.93 (d, 4H), 6.82 (d, 2H), 3.85 (s, 3H), 3.81(s, 3H), 3.79 (t, 2H), 3.18 (t, 2H).

$^{13}$C{$^{1}$H} NMR (500 MHz, $CDCl_3$): δ 167.78, 160.63, 159.40, 149.23, 136.00, 132.25, 129.98, 129.29, 123.65, 121.03, 113.71, 113.28, 55.27, 53.60, 40.27.

HRMS (ESI) m/z [M + Na]$^{+}$ calculated for $C_{22}H_{22}N_2O_2$ 346.1681, found 347.1759.

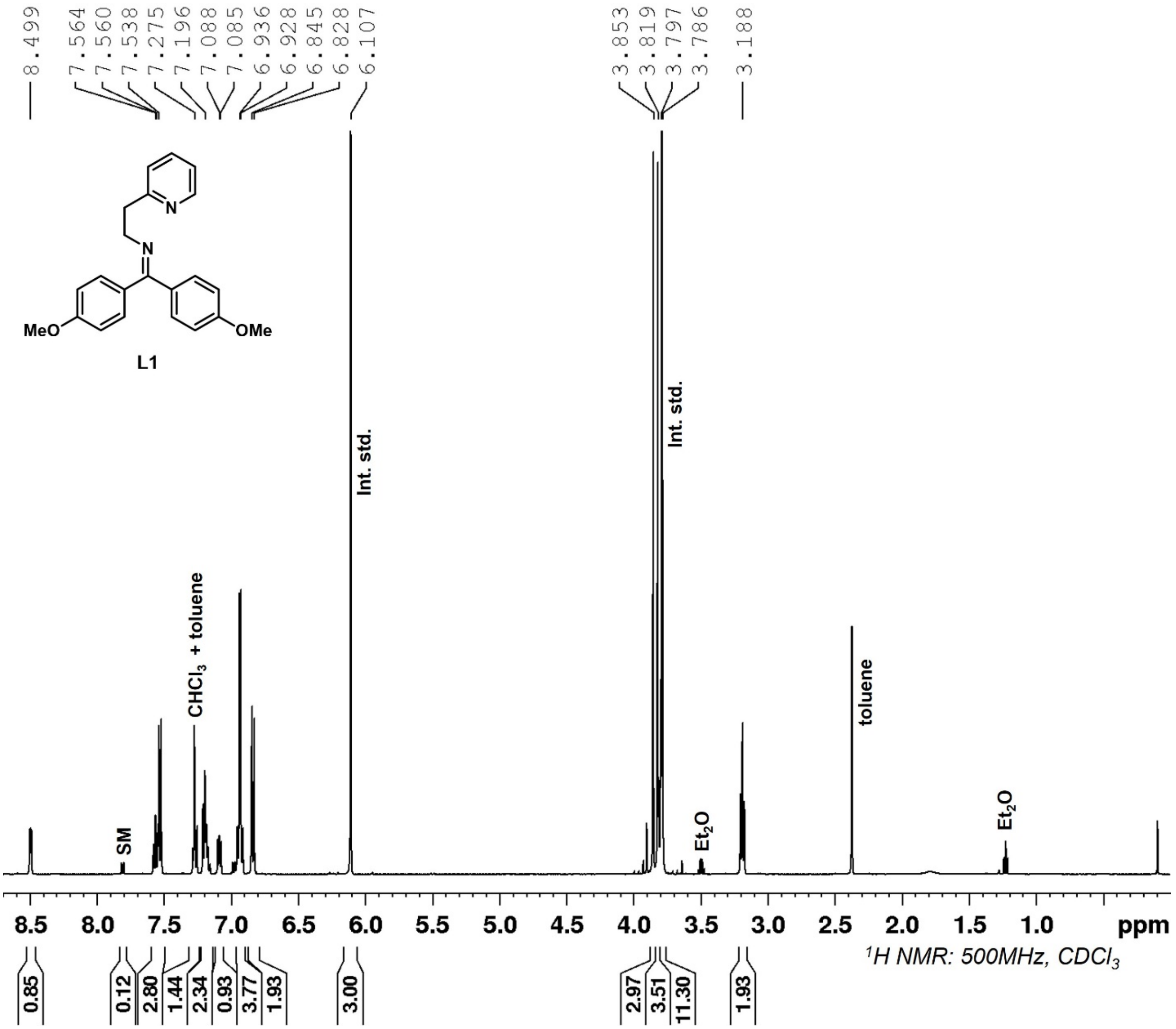


**Figure S1.** $^1$H-NMR spectra of L1.

### 3.1.1. Hydroxylation of L1

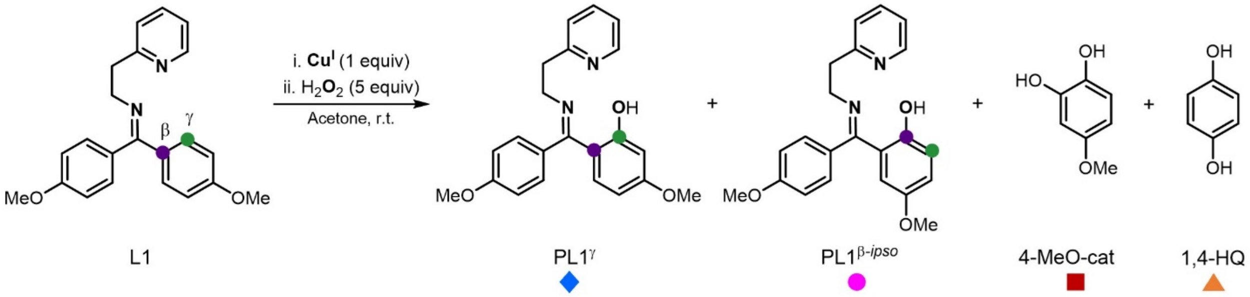


The reaction was carried out on 0.159 mmol scale using 56.8 mg of the imine according to the standard procedure. The brown crude product was quantified using 0.159 mmol of 1,3,5-trimethoxybenzene (int. std.) (45% yield, a mixture of PL1$^{\gamma}$, PL1$^{\beta\text{-ipso}}$, catechol, and HQ). The identity of the hydroxylation products was confirmed by $^{1}$H-NMR.

Note: The ratio of $\gamma^{Oxid}/\beta^{Oxid}$ (11/89) is calculated using the integration of CH peaks of product derived from γ C-H hydroxylation (PL1$^{\gamma}$) and hydroxylation products derived from β-ipso hydroxylation (PL1$^{\beta\text{-}ipso}$, 4-MeO-cat, and 1,4-dihydroquinone).

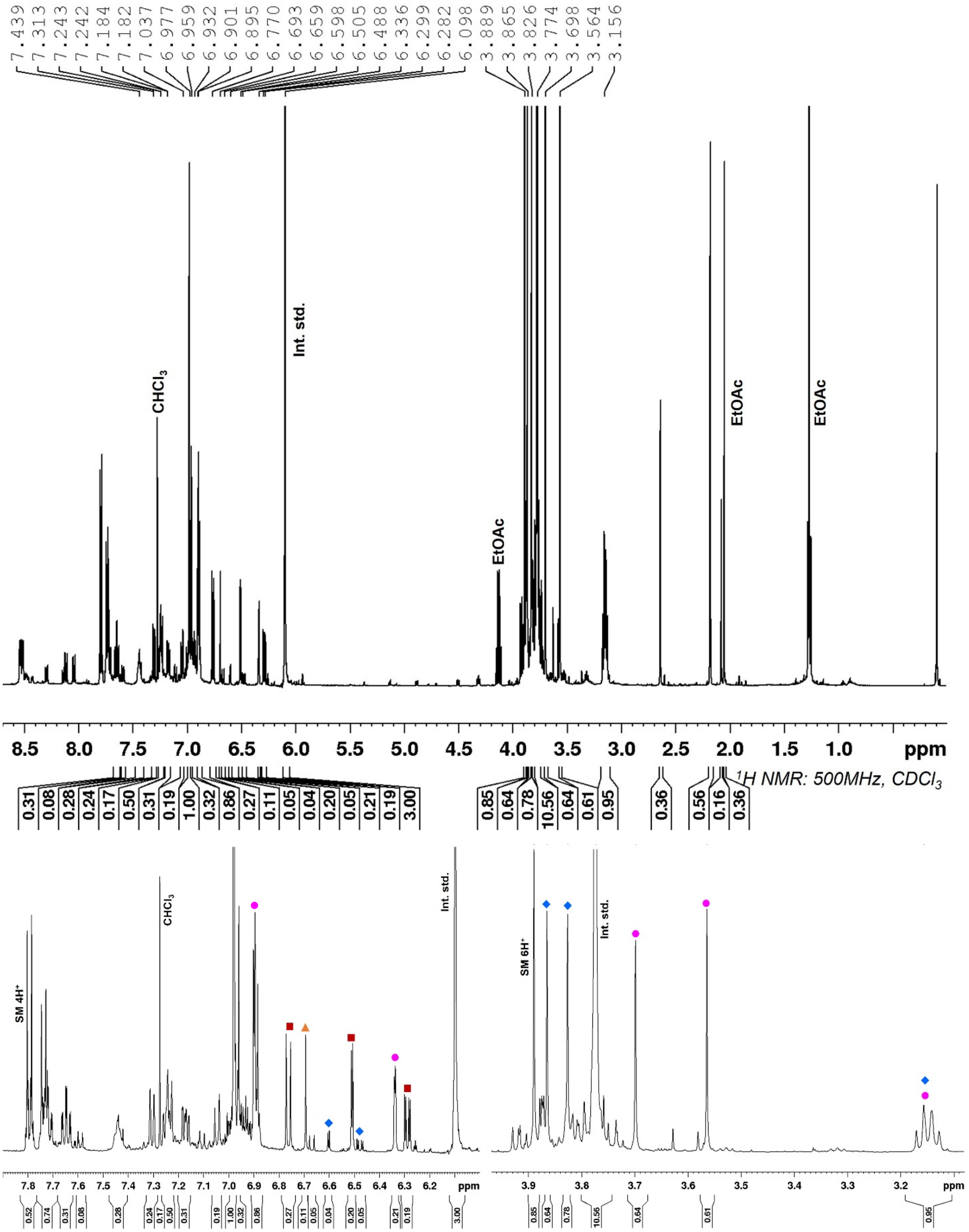


**Figure S2.** $^1$H-NMR spectra for the hydroxylation of L1.

### 3.1.2. Cleavage of DG of PL1

In a round bottom flask equipped with a stir bar, the mixture of $PL1^{\gamma}$, $PL1^{\beta\text{-ipso}}$, catechol, and HQ was dissolved using 50 mL EtOAc. To this mixture 100 mL 1M HCl was added slowly and let react for 30 min. The resulting mixture was extracted with EtOAc (50 mL X 2). The organic phases were separated, combined, dried over $MgSO_4$, filtered, and dried under vacuum. The organic products were quantified using 0.159 mmol of 1,3,5-trimethoxybenzene (int. std.) The identity of the cleaved products was confirmed by $^1$H-NMR.[4-5]

Note: The ratio of $\gamma^{Oxid}/\beta^{Oxid}$ (15/85) is calculated using the integration of CH peaks of cleaved product derived from γ C-H hydroxylation ($P1^{\gamma}$) and cleaved products derived from β-ipso hydroxylation ($P1^{\beta\text{-}ipso}$, 4-MeO-cat, and 1,4-dihydroquinone).

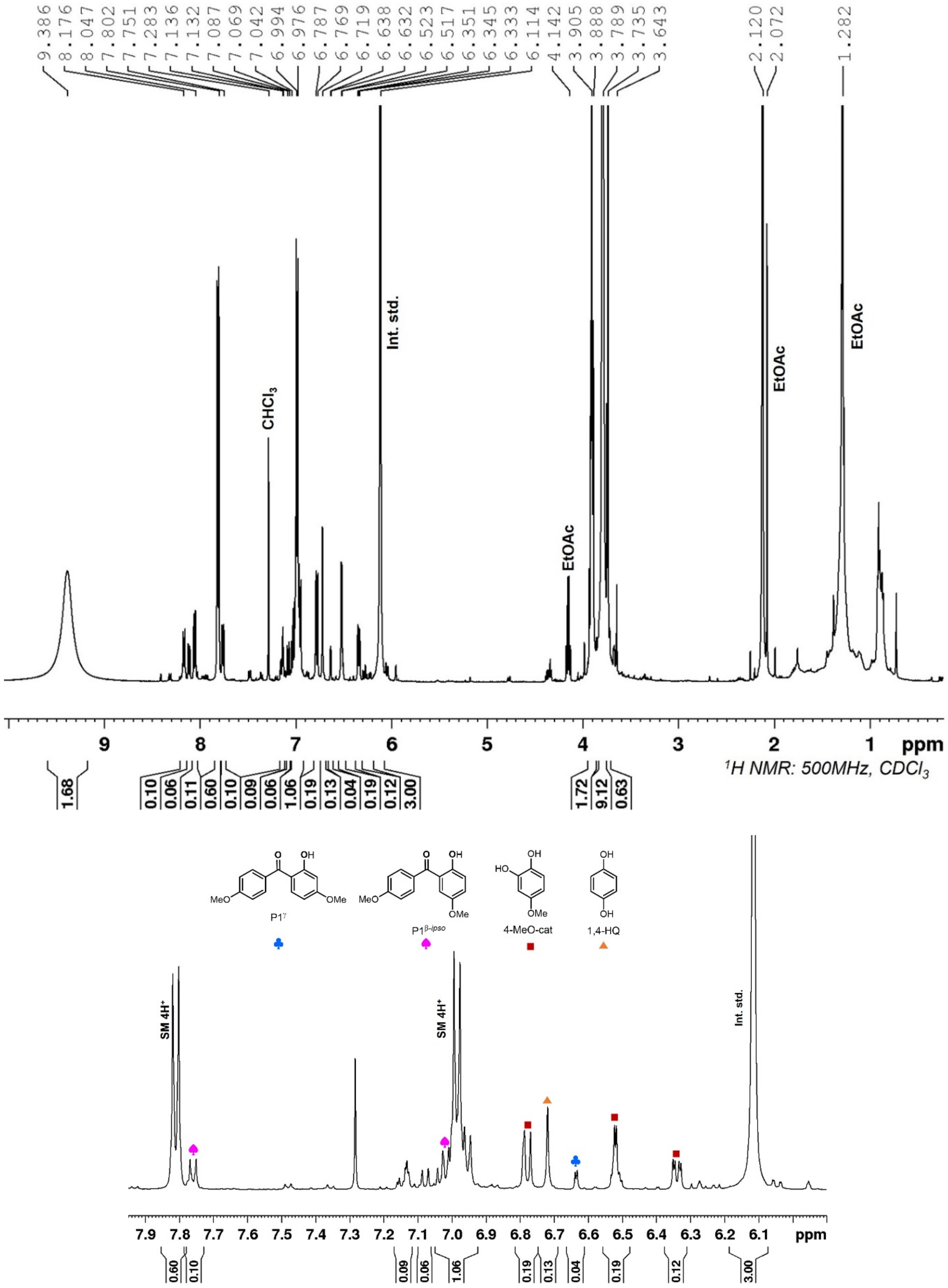


**Figure S3.** $^{1}$H-NMR spectra for the cleavage of DG of PL1.

### 3.2. Synthesis of L2

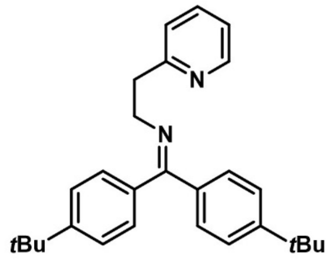

**L2**

In an oven-dried flask, 2-(2-pyridyl)ethylamine (0.41 mL, 3.63 mmol, 2.2 equiv) was added to 4,4'-ditert-butylbenzophenone (500 mg, 1.65 mmol) and p-toluenesulfonic acid monohydrate (cat. 10 mg, 4 mol%) in toluene (30 mL). The reaction mixture was refluxed under argon with a Dean-Stark apparatus until imine formation was complete (24 h). The reaction was cooled to room temperature and diluted with diethyl ether (20 mL). The organic layer was washed with saturated ammonia chloride (30 mL x 2), saturated aqueous sodium bicarbonate (30 mL), brine (30 mL), and dried with magnesium sulfate. The final product isolated was a beige solid (62% yield, 95% pure).

$^1$H-NMR (500 MHz, $CDCl_3$): δ 8.50 (d, 1H), 7.58 (dt, 1H), 7.51 (d, 2H), 7.39 (d, 2H), 7.36 (d, 2H), 7.23 (d, 1H), 7.09 (m, 1H), 6.95 (d, 2H), 3.81 (t, 2H), 3.21 (t, 2H), 1.37 (s, 9H), 3.32(s, 9H).

$^{13}$C{$^1$H} NMR (500 MHz, $CDCl_3$): δ 168.51, 160.71, 152.94, 151.07, 149.20, 137.55, 135.97, 133.81, 128.11, 127.49, 125.16, 124.89, 123.70, 121.00, 53.59, 40.23, 34.69, 31.34, 31.22.

HRMS (ESI) m/z $[M + Na]^+$ calculated for $C_{28}H_{34}N_2$ 398.2722, found 399.2814.

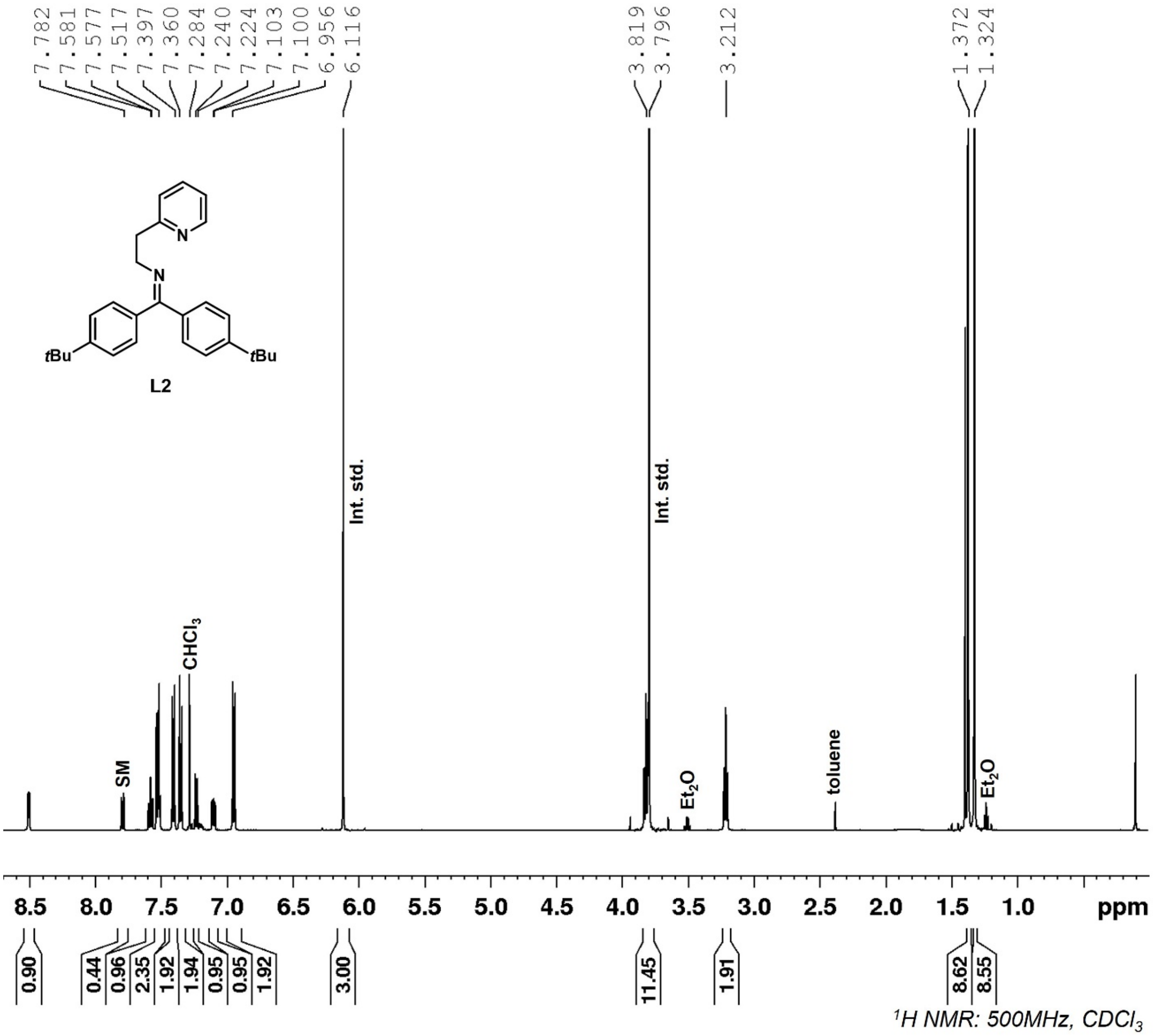


**Figure S4.** $^1$H-NMR spectra of L2.

### 3.2.1. Hydroxylation of L2

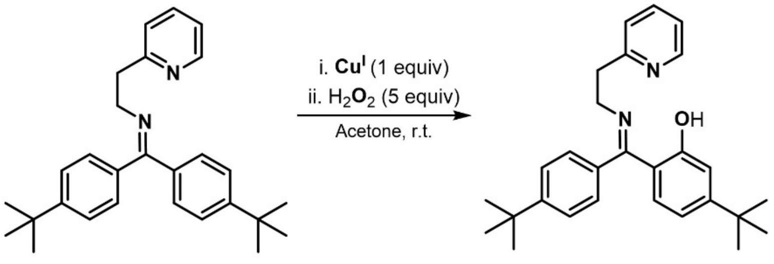


L2 PL2$^{\gamma}$

The reaction was carried out on 0.159 mmol scale using 64.0 mg of the imine according to the standard procedure. The crude product was quantified using 0.159 mmol of 1,3,5-trimethoxybenzene (int. std.) (41% yield). The identity of the hydroxylation products was confirmed by $^1$H-NMR.[1,2]

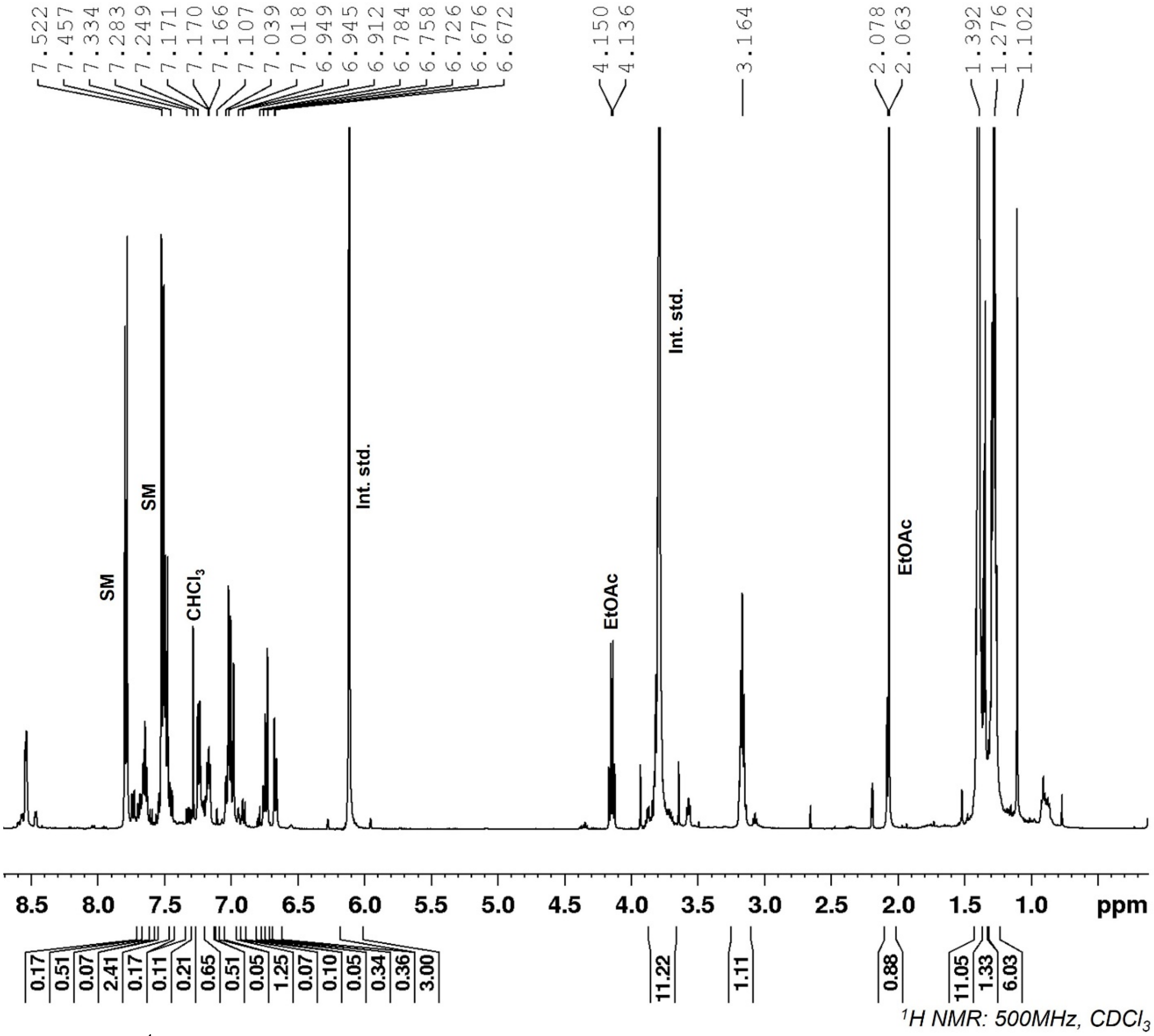


**Figure S5.** $^1$H-NMR spectra for the hydroxylation of L2.

### 3.2.2. Cleavage of DG of PL2

In a round bottom flask equipped with a stir bar, PL2$^{\gamma}$ was dissolved using 50 mL EtOAc. To this mixture 100 mL 1M HCl was added slowly and let react for 30 min. The resulting mixture was extracted with EtOAc (50 mL X 2). The organic phases were separated, combined, dried over $MgSO_4$, filtered, and dried under vacuum. The organic products were quantified using 0.159 mmol of 1,3,5-trimethoxybenzene (int. std.) The identity of the cleaved products was confirmed by $^1$H-NMR.[1,2]

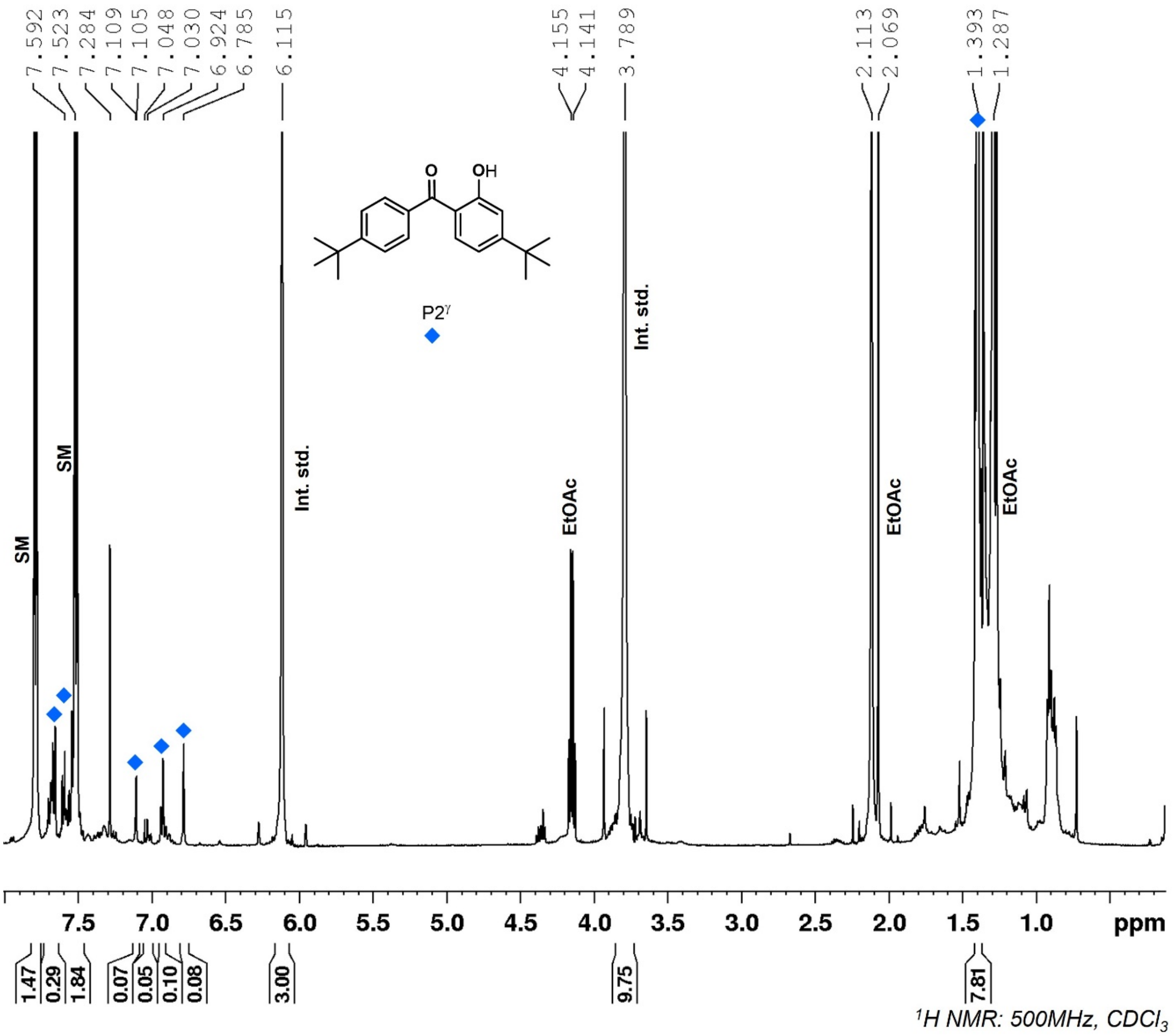


**Figure S6.** $^1$H-NMR spectra for the cleavage of DG of PL2.

### 3.3. Synthesis of L3

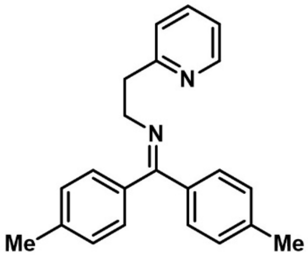

**L3**

In an oven-dried flask, 2-(2-pyridyl)ethylamine (2.60 mL, 21.7 mmol, 2.2 equiv) was added to 4,4'-dimethylbenzophenone (2.09 g, 9.85 mmol) and p-toluenesulfonic acid monohydrate (cat. 20 mg, 1.2 mol%) in toluene (50 mL). The reaction mixture was refluxed under argon with a Dean-Stark apparatus until imine formation was complete (24 h). The reaction was cooled to room temperature and diluted with diethyl ether (30 mL). The organic layer was washed with saturated ammonia chloride (50 mL x 2), saturated aqueous sodium bicarbonate (50 mL), brine (50 mL), and dried with magnesium sulfate. The final product isolated was an orange solid (95% yield, 97% pure).

$^1$H-NMR (500 MHz, $CDCl_3$): δ 8.49 (d, 1H), 7.55 (dt, 1H), 7.47(d, 2H), 7.21 (d, 3H), 7.13 (d, 2H), 7.08 (m, 1H), 6.89 (d, 2H), 3.79 (t, 2H), 3.19 (t, 2H), 2.39 (s, 3H), 2.35 (s, 3H).

$^{13}$C{$^1$H} NMR (500 MHz, $CDCl_3$): δ 168.59, 160.60, 149.23, 139.91, 137.99, 137.51, 133.87, 129.01, 128.70, 128.35, 127.70, 123.66, 121.02, 53.65, 40.18, 21.32.

HRMS (ESI) m/z $[M + Na]^+$ calculated for $C_{22}H_{22}N_2$ 314.1783, found 315.1859.

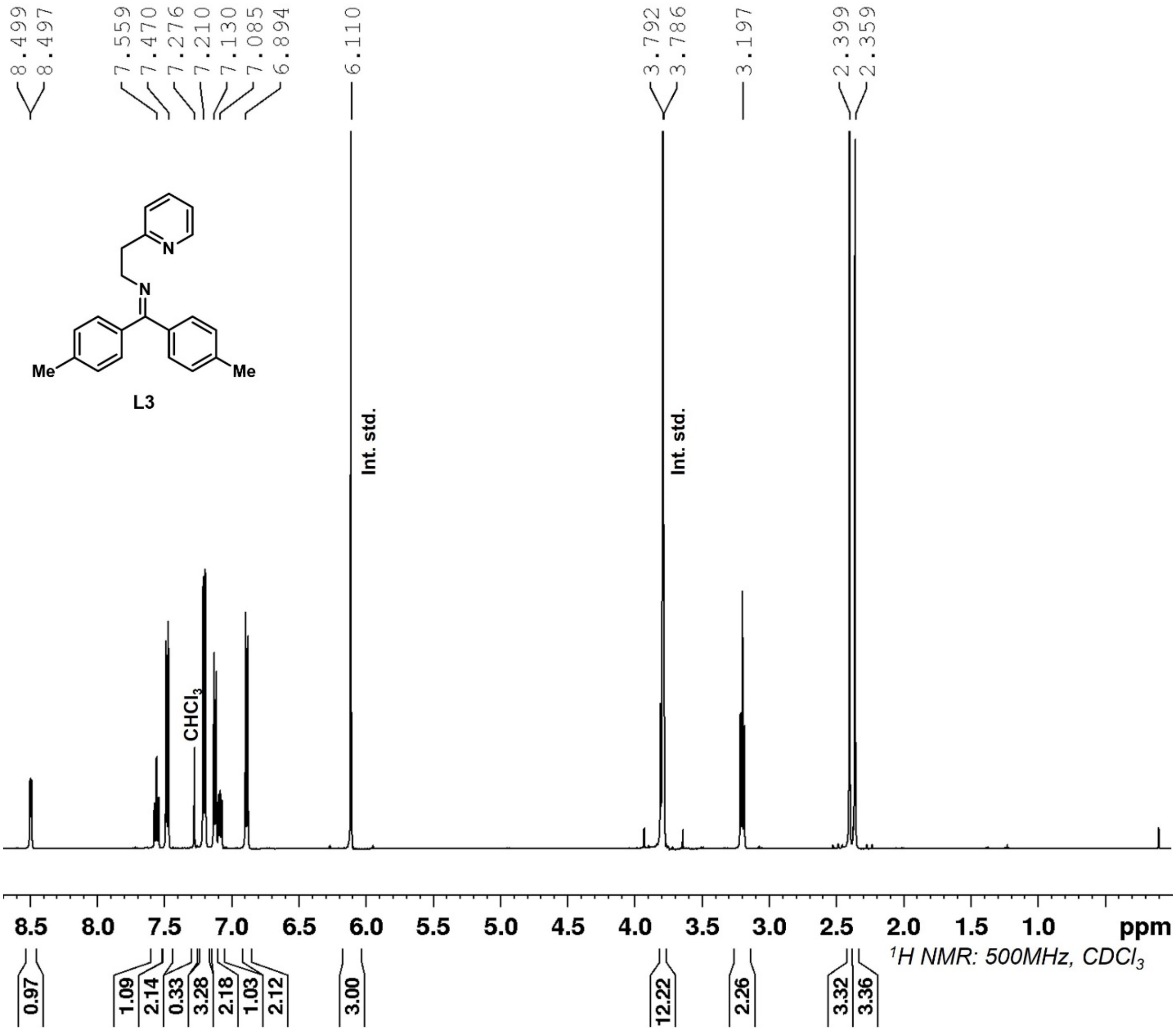


**Figure S7.** $^1$H-NMR spectra of L3.

### 3.3.1. Hydroxylation of L3

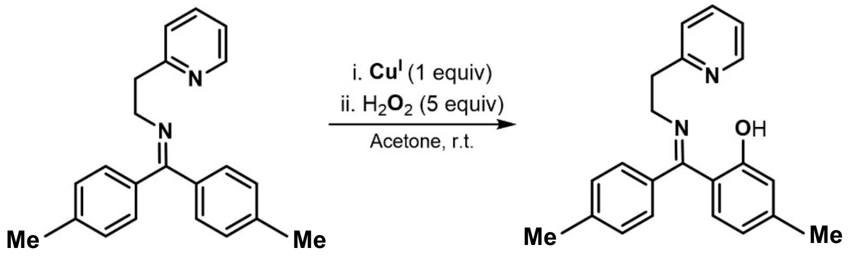


The reaction was carried out on 0.159 mmol scale using 51.5 mg of the imine according to the Standard Procedure. The brown crude product was quantified using 0.159 mmol of 1,3,5-trimethoxybenzene (int. std.) (49% yield). The identity of the hydroxylation products was confirmed by $^1$H-NMR.[1]

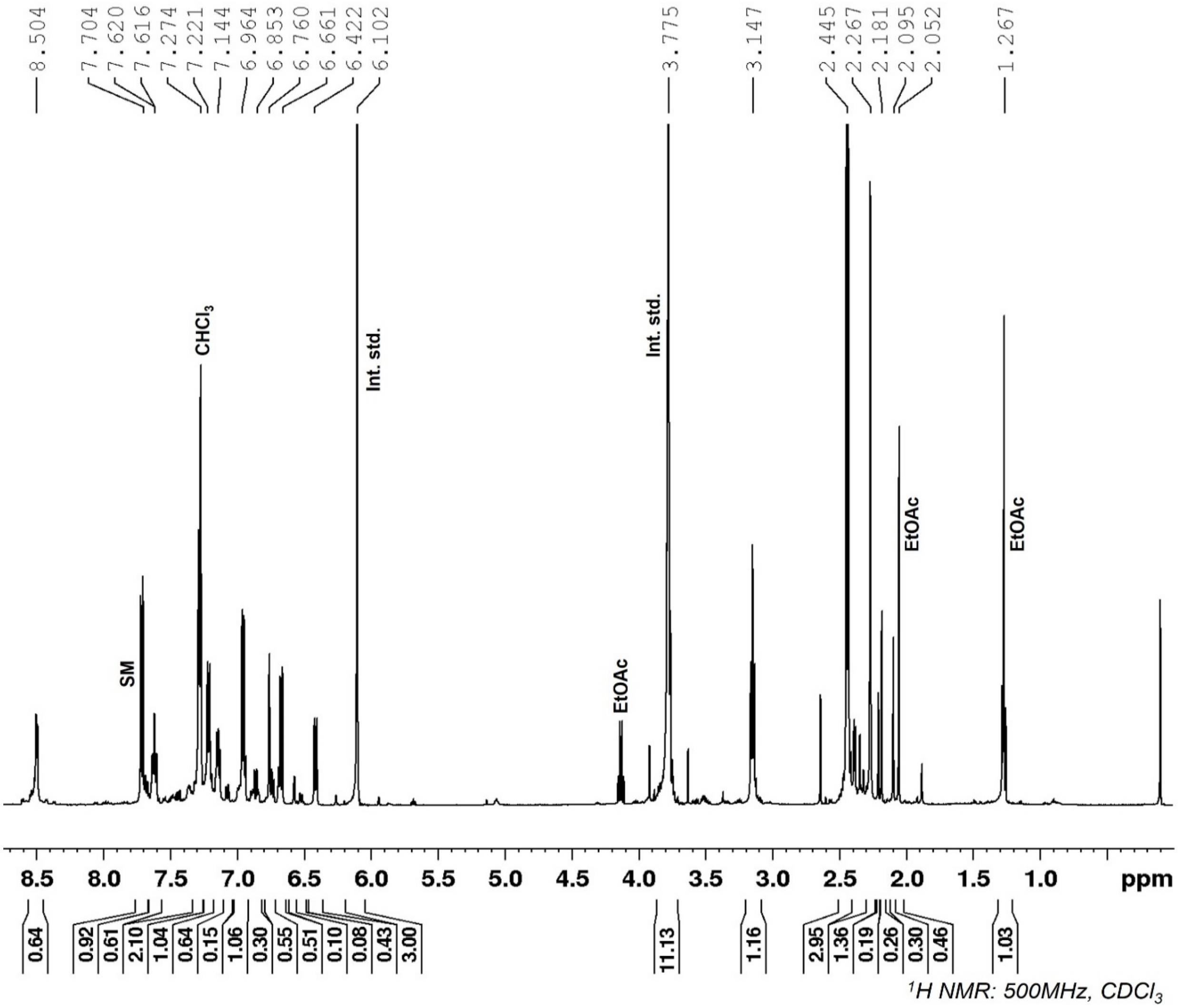


**Figure S8.** $^1$H-NMR spectra for the hydroxylation of L3.

### 3.4. Synthesis of L4

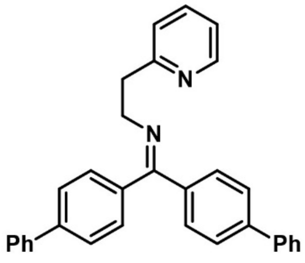

**L4**

In an oven-dried flask, 2-(2-pyridyl)ethylamine (0.75 mL, 6.45 mmol, 2.2 equiv) was added to 4,4'-diphenylbenzophenone (1 g, 2.93 mmol) and p-toluenesulfonic acid monohydrate (cat. 10 mg, 2 mol%) in toluene (30 mL). The reaction mixture was refluxed under argon with a Dean-Stark apparatus until imine formation was complete (24 h). The reaction was cooled to room temperature and diluted with diethyl ether (20 mL). The organic layer was washed with saturated ammonia chloride (30 mL x 2), saturated aqueous sodium bicarbonate (30 mL), brine (30 mL), and dried with magnesium sulfate. The final product isolated was a beige powder (72% yield, 96% pure).

$^1$H-NMR (500 MHz, $CDCl_3$): δ 8.52 (d, 1H), 7.70 (d, 2H), 7.65 (d, 4H), 7.61 (d, 2H), 7.59 (d, 2H), 7.49 (t, 2H), 7.46 (t, 2H), 7.40 (t, 1H), 7.39 (t, 1H), 7.26 (d, 1H), 7.13 (d, 3H), 3.90 (t, 2H), 3.26 (t, 2H).

$^{13}$C{$^1$H} NMR (500 MHz, $CDCl_3$): δ 168.05, 160.47, 149.30, 142.66, 141.21, 140.56, 140.48, 138.84, 136.08, 135.50, 130.69, 128.99, 128.88, 128.82, 128.29, 127.63, 127. 61, 127.33, 127.15, 126.80, 123.72, 121.13, 53.88, 40.14.

HRMS (ESI) m/z [M + Na]$^+$ calculated for $C3_2H_{26}N_2$ 438.2096, found 439.2187.

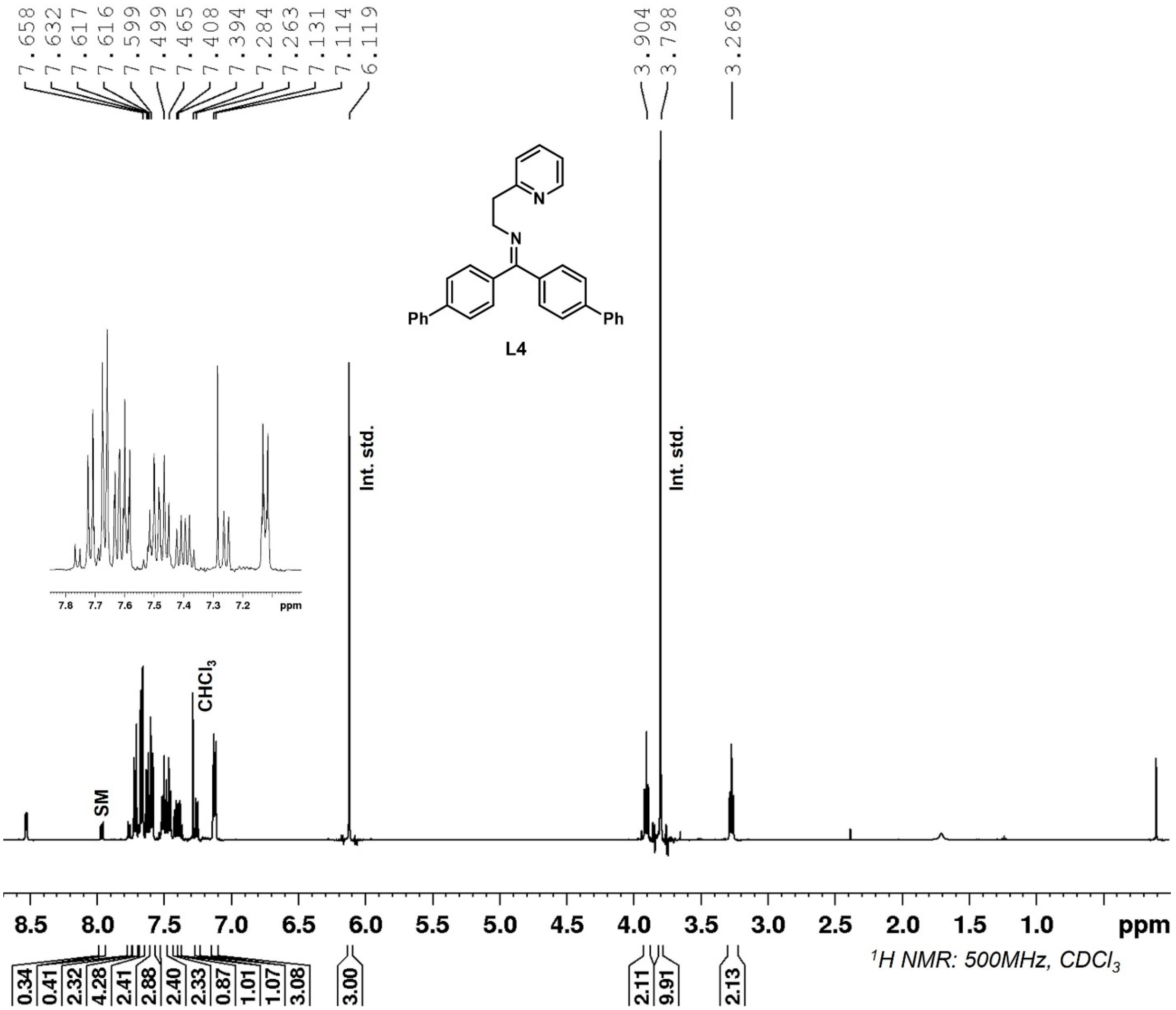


**Figure S9.** $^1$H-NMR spectra of L4.

### 3.4.1. Hydroxylation of L4

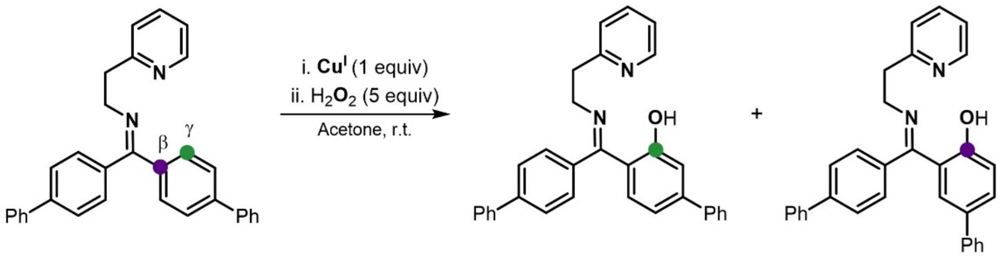


L4 PL4$^{\gamma}$ PL4$^{\beta\text{-}ipso}$

The reaction was carried out on 0.159 mmol scale using 75.6 mg of the imine according to the standard procedure. The crude product was quantified using 0.159 mmol of 1,3,5-trimethoxybenzene (int. std.) (65% yield, a mixture of PL4$^{\gamma}$, PL4$^{\beta\text{-ipso}}$). The identity of the hydroxylation products was confirmed by $^{1}$H-NMR.

Note: The ratio of $\gamma^{Oxid}/\beta^{Oxid}$ (49/51) is calculated using the integration of CH peaks of product derived from γ C-H hydroxylation (PL4$^{\gamma}$) and hydroxylation product derived from β-ipso hydroxylation (PL4$^{\beta\text{-}ipso}$).

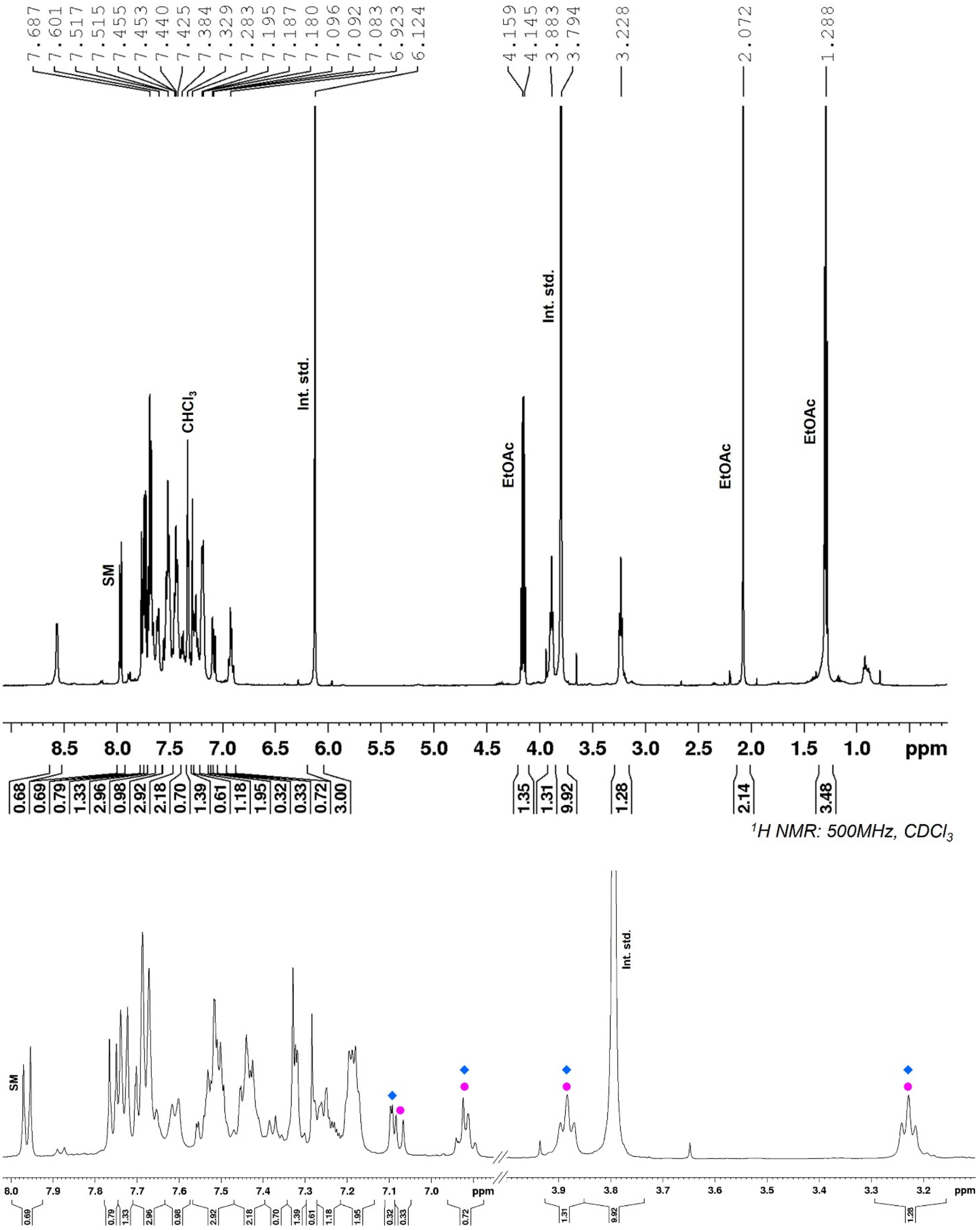


**Figure S10.** $^{1}$H-NMR spectra for the hydroxylation of L4.

### 3.4.2. Cleavage of DG of PL4

In a round bottom flask equipped with a stir bar, the mixture of $PL4^{\gamma}$, $PL4^{\beta\text{-ipso}}$ was dissolved using 50 mL EtOAc. To this mixture 100 mL 1M HCl was added slowly and let react for 30 min. The resulting mixture was extracted with EtOAc (50 mL X 2). The organic phases were separated, combined, dried over $MgSO_4$, filtered, and dried under vacuum. The organic products were quantified using 0.159 mmol of 1,3,5-trimethoxybenzene (int. std.) The identity of the cleaved products was confirmed by $^1$H-NMR (Compound 3e from *Eur. J. Org. Chem.* **2019**, *24*, 3877–3881 and 3c from *Chem. Comm.*, **2016**, *52*, 12372-12375 were referred to identify $P4^{\gamma}$, $P4^{\beta\text{-ipso}}$).

Note: The ratio of $\gamma^{Oxid}/\beta^{Oxid}$ (48/52) is calculated using the integration of OH peaks of cleaved product derived from γ C-H hydroxylation ($P4^{\gamma}$) and cleaved products derived from β-ipso hydroxylation ($P4^{\beta\text{-}ipso}$).

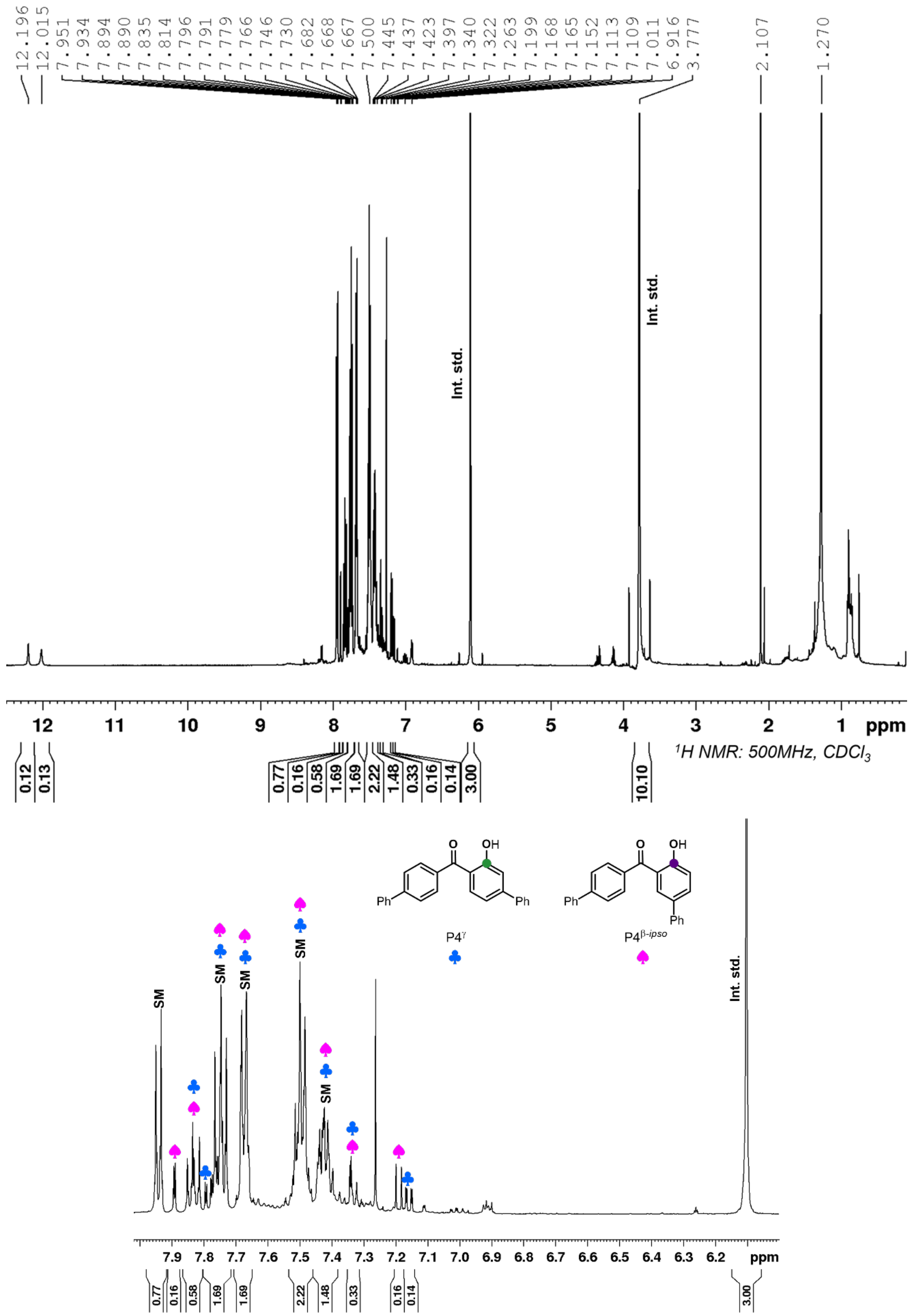


**Figure S11.** $^{1}$H-NMR spectra for the cleavage of DG of PL4.

### 3.5. Synthesis of L5

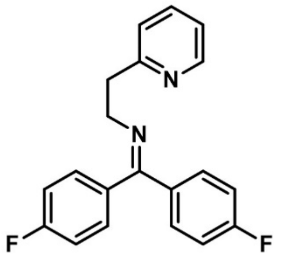

**L5**

In an oven-dried flask, 2-(2-pyridyl)ethylamine (2.60 mL, 21.7 mmol, 2.2 equiv) was added to 4,4'-difluorobenzophenone (2.17 g, 9.85 mmol) and p-toluenesulfonic acid monohydrate (cat. 20 mg, 1.2 mol%) in toluene (50 mL). The reaction mixture was refluxed under argon with a Dean-Stark apparatus until imine formation was complete (24 h). The reaction was cooled to room temperature and diluted with diethyl ether (30 mL). The organic layer was washed with saturated ammonia chloride (50 mL x 2), saturated aqueous sodium bicarbonate (50 mL), brine (50 mL), and dried with magnesium sulfate. The final product isolated was an orange solid (73% yield, 91% pure).

$^1$H-NMR (500 MHz, $CDCl_3$): δ 8.41 (d, 1H), 7.55 (m, 3H), 7.18 (m, 1H), 7.10 (m, 3H), 6.99-6.96 (m, 4H), 3.77 (t, 2H), 3.18 (t, 2H).

$^{13}C\{^1H\}$ NMR (500 MHz, $CDCl_3$): δ 166.32, 165.02, 163.56, 163.03, 161.58, 160.27, 149.32, 136.08, 132.55, 132.47, 132.24, 132.21, 130.23, 129.66, 129.60, 123.63, 121.16, 115.75, 115.58, 115.11, 114.94, 53.73, 39.97.

HRMS (ESI) m/z $[M + Na]^+$ calculated for $C_{22}H_{22}N_2$ 322.1282, found 323.1377.

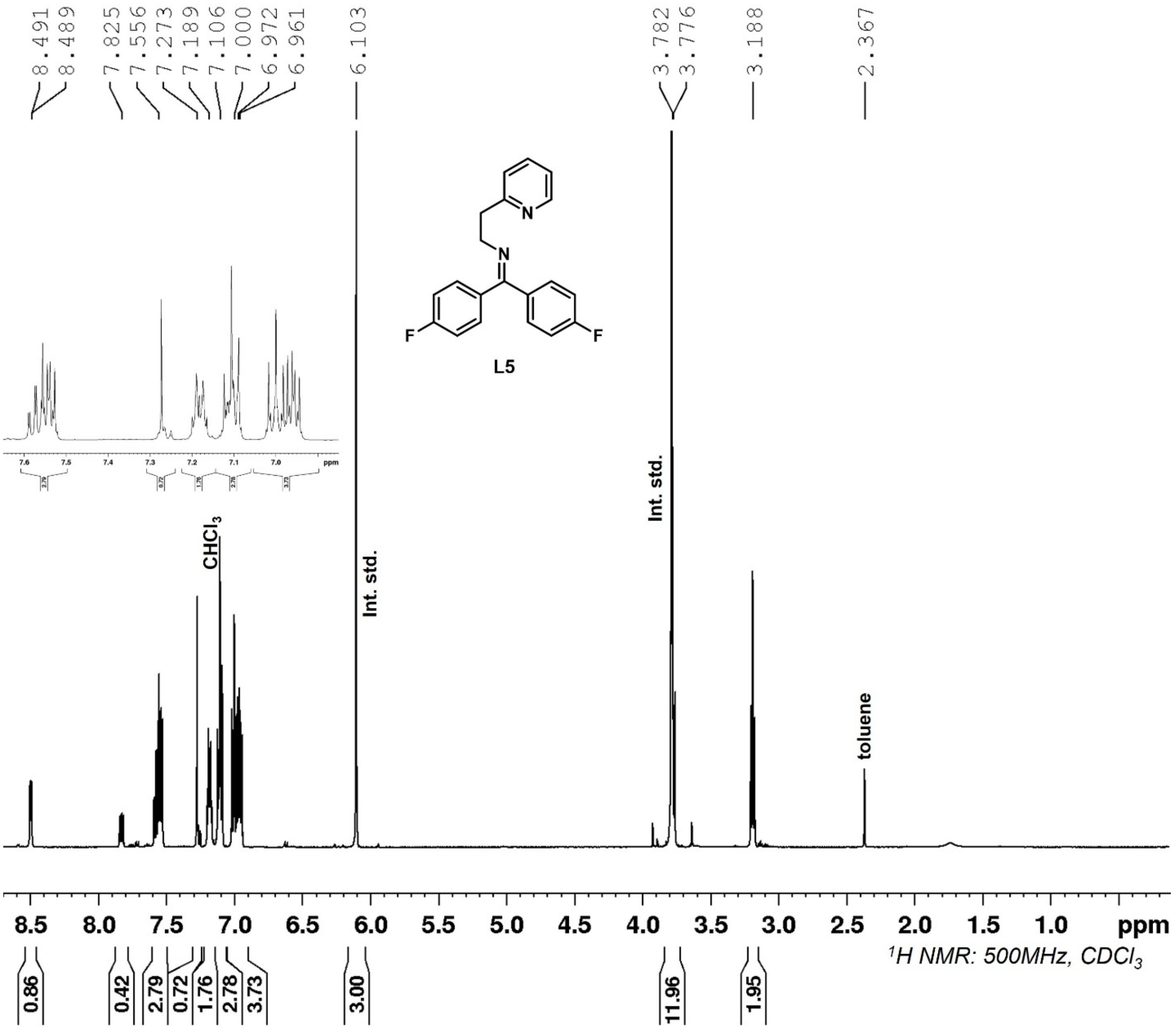


**Figure S12.** $^1$H-NMR spectra of L5.

### 3.5.1. Hydroxylation of L5

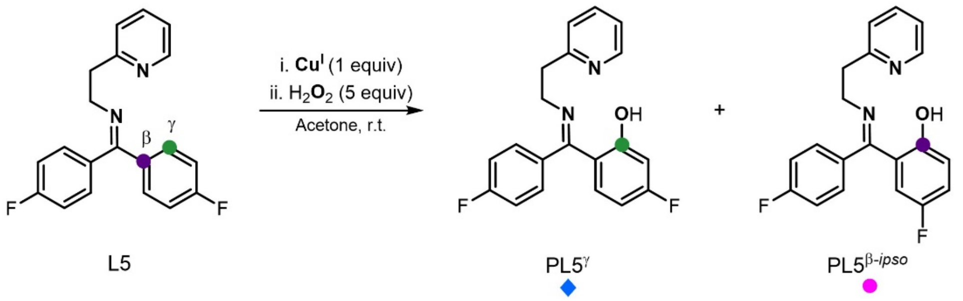


The reaction was carried out on 0.159 mmol scale using 55.7 mg of the imine according to the Standard Procedure. The crude product was quantified using 0.159 mmol of 1,3,5-trimethoxybenzene (int. std.) (32% yield, a mixture of PL5$^γ$, PL5$^{β\text{-}ipso}$). The identity of the hydroxylation products was confirmed by $^1$H-NMR.[1,2]

Note: The ration of $γ^{Oxid}/β^{Oxid}$ (56/44) is calculated using the integration of CH peaks of product derived from γ C-H hydroxylation (PL5$^γ$) and hydroxylation product derived from β-ipso hydroxylation (PL5$^{β\text{-}ipso}$).

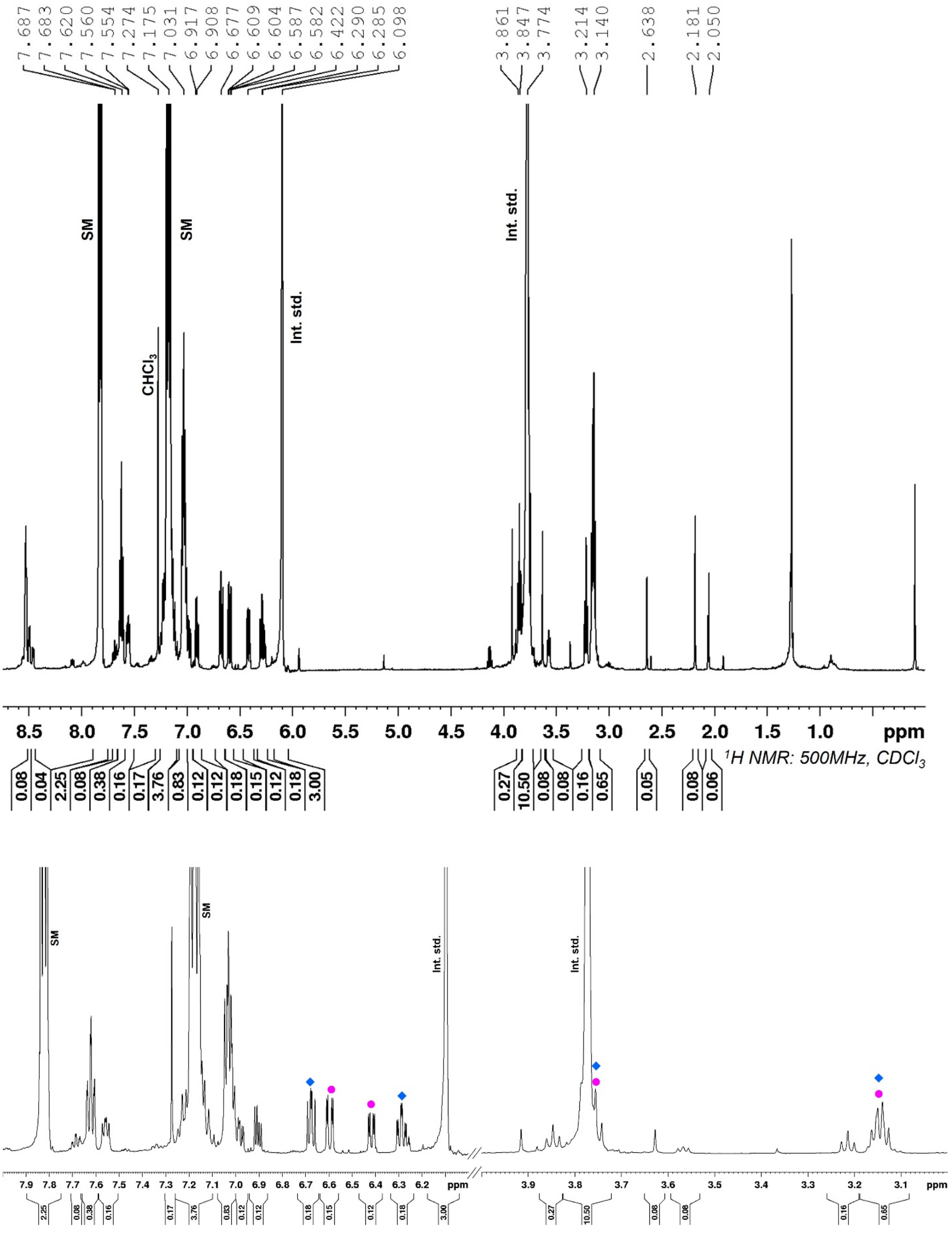


**Figure S13.** $^1$H-NMR spectra for the hydroxylation of L5.

### 3.5.2. Cleavage of DG of PL5

In a round bottom flask equipped with a stir bar, the mixture of PL5$^{\gamma}$, PL5$^{\beta\text{-ipso}}$ was dissolved using 50 mL EtOAc. To this mixture 100 mL 1M HCl was added slowly and let react for 30 min. The resulting mixture was extracted with EtOAc (50 mL X 2). The organic phases were separated, combined, dried over $MgSO_4$, filtered, and dried under vacuum. The organic products were quantified using 0.159 mmol of 1,3,5-trimethoxybenzene (int. std.) The identity of the cleaved products was confirmed by $^1$H-NMR.[6]

Note: The ratio of $\gamma^{Oxid}/\beta^{Oxid}$ (44/56) is calculated using the integration of CH peaks of cleaved product derived from $\gamma$ C-H hydroxylation (P5$^{\gamma}$) and cleaved products derived from β-ipso hydroxylation (P5$^{\beta\text{-}ipso}$).

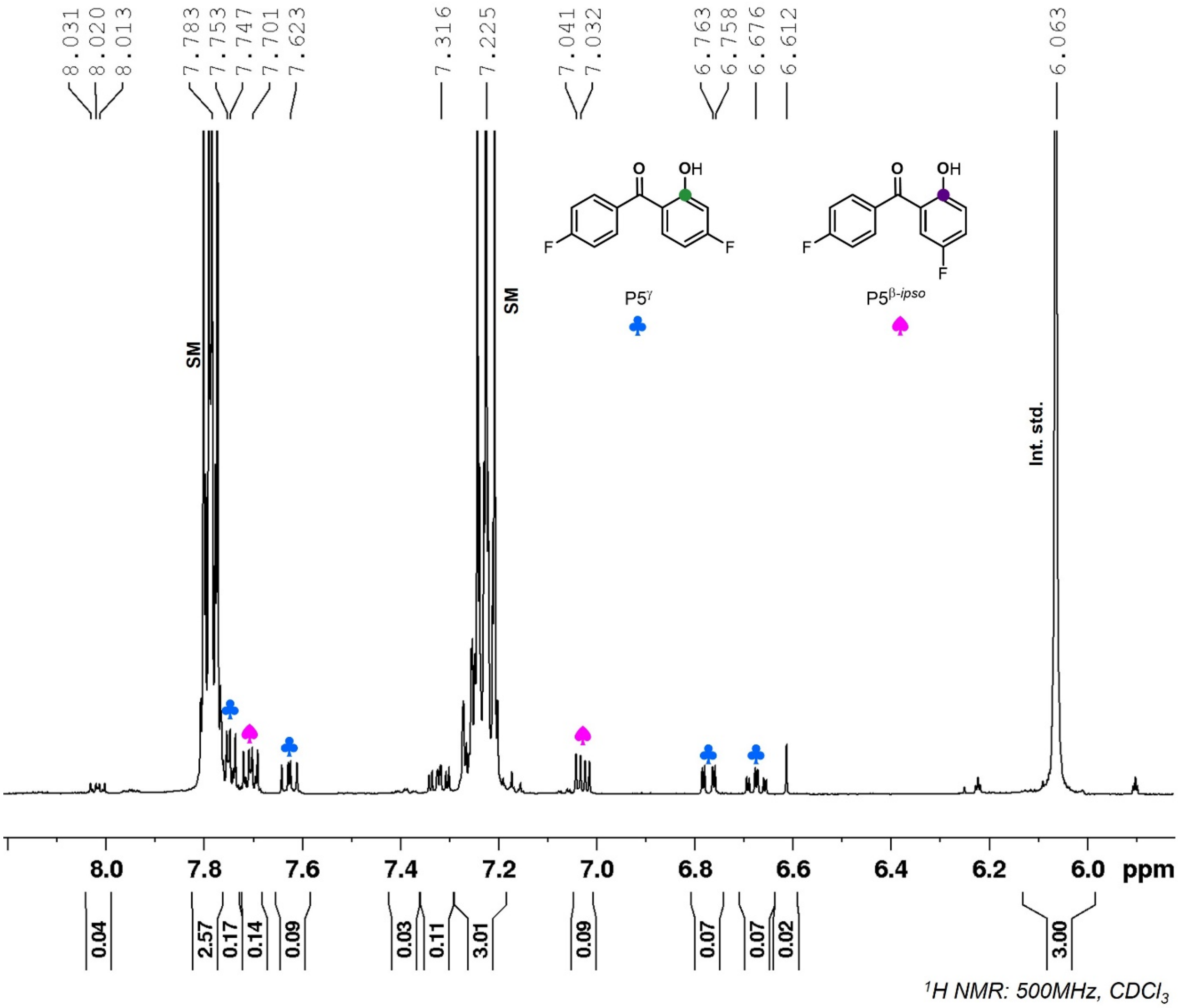


**Figure S14.** $^1$H-NMR spectra for the cleavage of DG of PL5.

### 3.6. Synthesis of L6

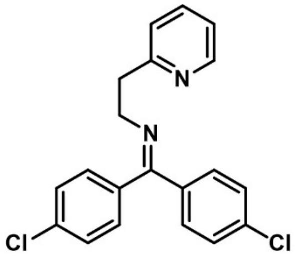

**L6**

In an oven-dried flask, 2-(2-pyridyl)ethylamine (2.60 mL, 21.7 mmol, 2.2 equiv) was added to 4,4'-dichlorobenzophenone (2.49 g, 9.85 mmol) and p-toluenesulfonic acid monohydrate (cat. 20 mg, 1.2 mol%) in toluene (50 mL). The reaction mixture was refluxed under argon with a Dean-Stark apparatus until imine formation was complete (24 h). The reaction was cooled to room temperature and diluted with diethyl ether (30 mL). The organic layer was washed with saturated ammonia chloride (50 mL x 2), saturated aqueous sodium bicarbonate (50 mL), brine (50 mL), and dried with magnesium sulfate. The final product isolated was a red-brown solid (80% yield, 95% pure).

$^{1}$H-NMR (500 MHz, $CDCl_3$): δ 8.48 (d, 1H), 7.56 (dt, 1H), 7.47(d, 2H), 7.39 (d, 2H), 7.29 (d, 2H), 7.17 (d, 1H), 7.09 (m, 1H), 6.90 (d, 2H), 3.77 (t, 2H), 3.18 (t, 2H).

$^{13}$C{$^{1}$H} NMR (500 MHz, $CDCl_3$): δ 166.19, 160.14, 149.33, 137.93, 136.26, 136.10, 134.64, 134.44, 129.55, 129.12, 128.91, 128.33, 123.65, 121.20, 53.82, 39.88.

HRMS (ESI) m/z $[M + Na]^+$ calculated for $C_{22}H_{22}N_2$ 355.2620, found 357.0761.

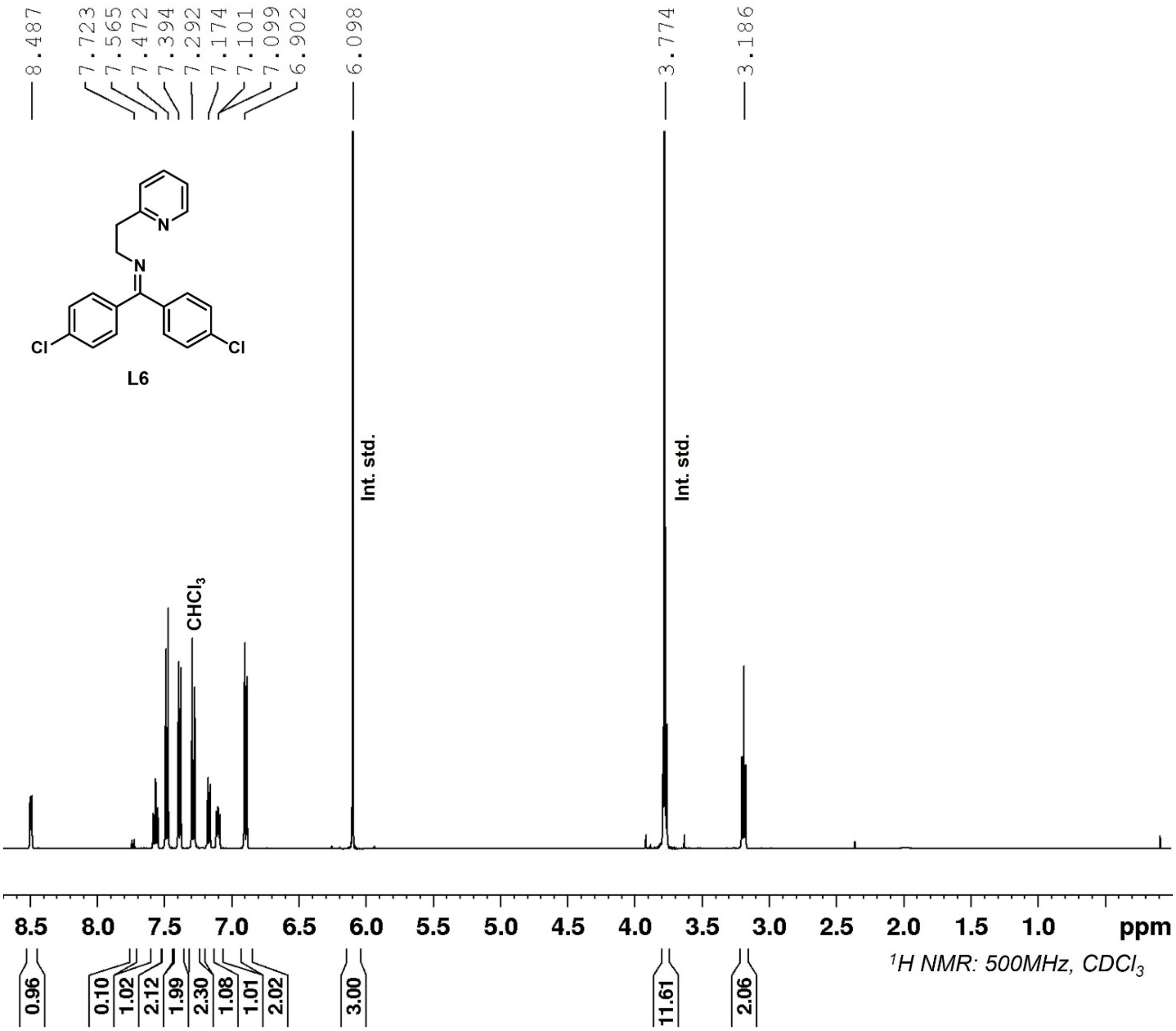


**Figure S15.** $^{1}$H-NMR spectra of L6.

### 3.6.1. Hydroxylation of L6

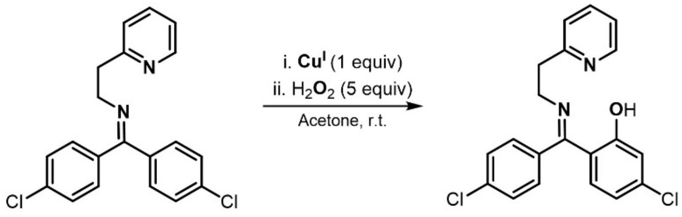


L6 PL6γ

The reaction was carried out on 0.159 mmol scale using 59.5 mg of the imine according to the standard procedure. The crude product was quantified using 0.159 mmol of 1,3,5-trimethoxybenzene (int. std.) (25% yield). The identity of the hydroxylation products was confirmed by $^1$H-NMR.[1]

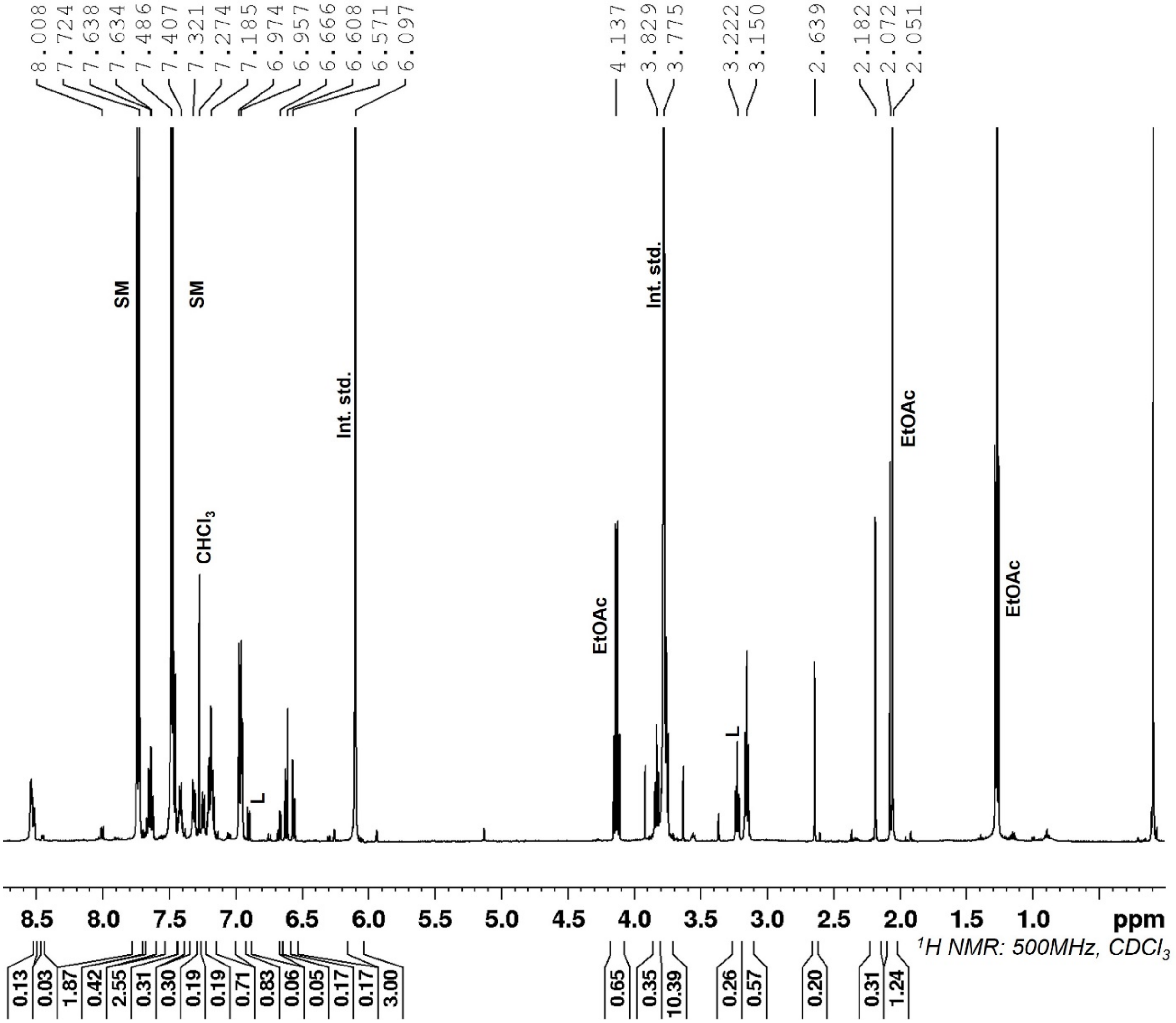


**Figure S16.** $^1$H-NMR spectra for the hydroxylation of L6.

## 4. Synthesis and hydroxylation of the unsymmetrical imine substrate-ligands

### 4.1. Synthesis of L7

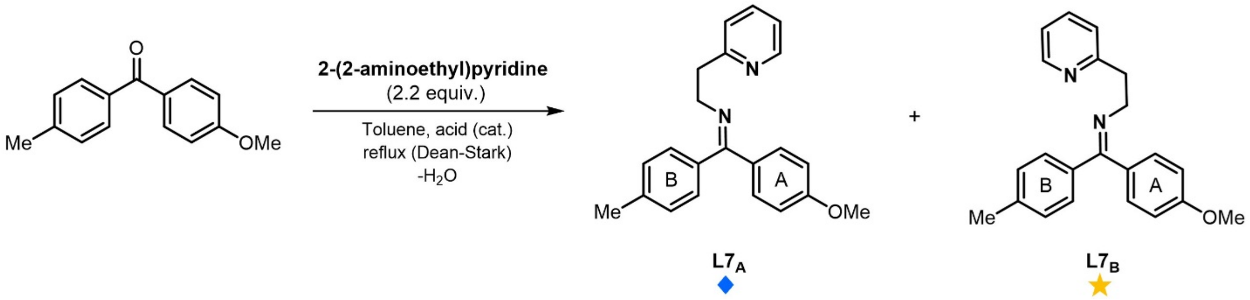


In an oven-dried flask, 2-(2-pyridyl)ethylamine (2.60 mL, 21.7 mmol, 2.2 equiv) was added to 4-methoxy-4'- methylbenzophenone (2.36 g, 9.85 mmol) and p-toluenesulfonic acid monohydrate (cat. 20 mg, 1.2 mol%) in toluene (50 mL). The reaction mixture was refluxed under argon with a Dean-Stark apparatus until imine formation was complete (24 h). The reaction was cooled to room temperature and diluted with diethyl ether (30 mL). The organic layer was washed with saturated ammonia chloride (50 mL x 2), saturated aqueous sodium bicarbonate (50 mL), brine (50 mL), and dried with magnesium sulfate. The final product isolated was an orange solid (96% yield, 95% pure).

$^1$H-NMR (500 MHz, $CDCl_3$): δ 8.49 (d, $L^A$+$L^B$: 2H), 7.57-7.54 (td, $L^A$+$L^B$: 2H), 7.53-7.51 (d, $L^A$: 2H), 7.47-7.45 (d, $L^B$: 2H), 7.19 (d, $L^A$+$L^B$: 4H), 7.13 (d, $L^A$+$L^B$: 2H), 7.08 (m, $L^A$+$L^B$: 2H), 6.93 (d, $L^B$: 4H), 6.89 (d, $L^A$: 2H), 6.83 (d, $L^A$: 2H), 3.85 (s, $L^B$: 3H), 3.81 (s, $L^A$: 3H). 3.83-3.80 (t, $L^B$: 2H), 3.77-3.74 (t, $L^A$: 2H), 3.17 (m, $L^A$+$L^B$: 4H), 2.39 (s, $L^A$: 3H), 2.35 (s, $L^B$: 3H).

HRMS (ESI) m/z $[M + Na]^+$ calculated for $C_{22}H_{22}N_2O$ 330.1732, found 331.1816.

Note: The ratio of $L7_A/L7_B$ (60/40) is calculated using the average of the integration of $CH_2$ peaks and CH peaks.

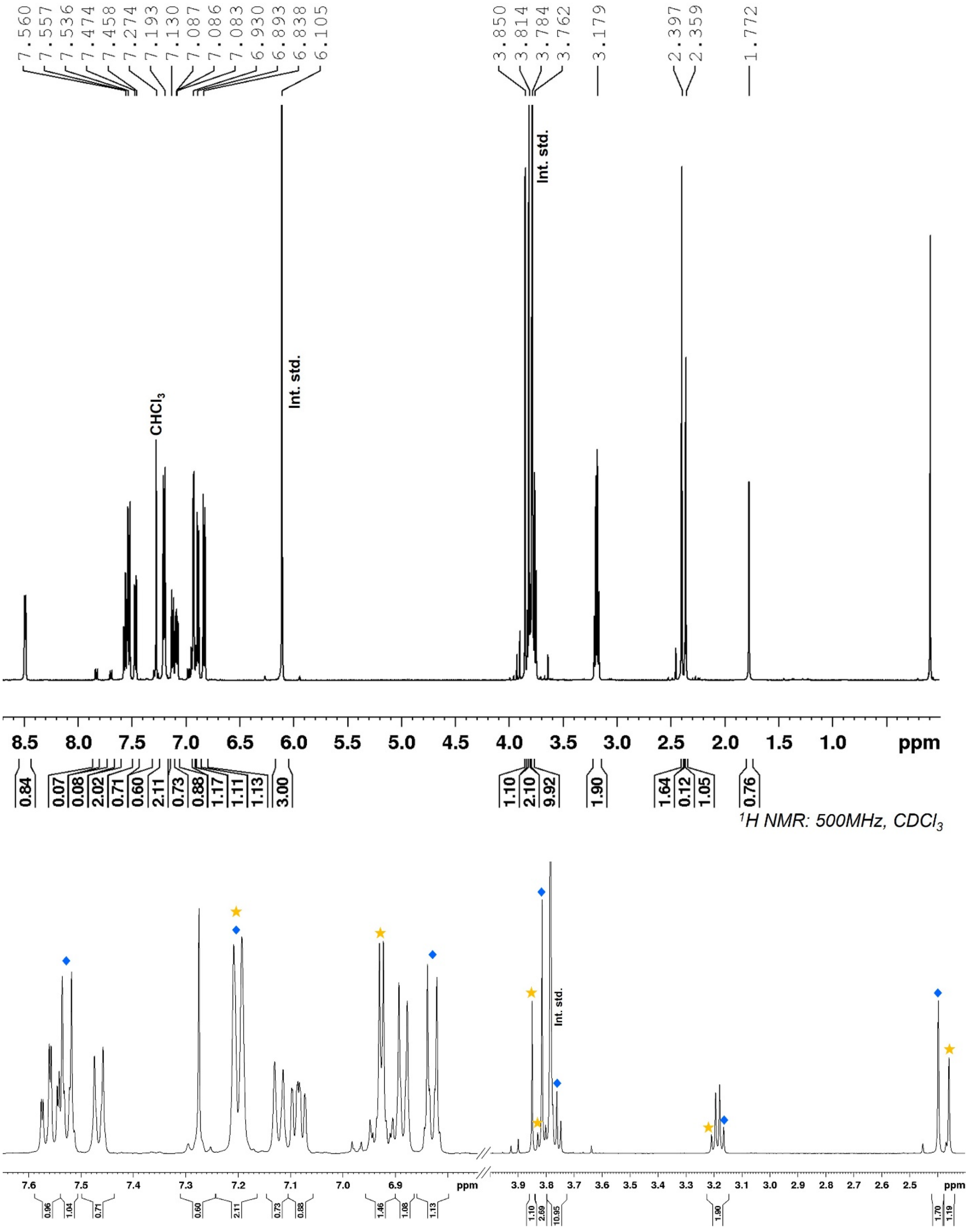


**Figure S17.** $^{1}$H-NMR spectra of L7.

#### 4.1.1. Hydroxylation of L7

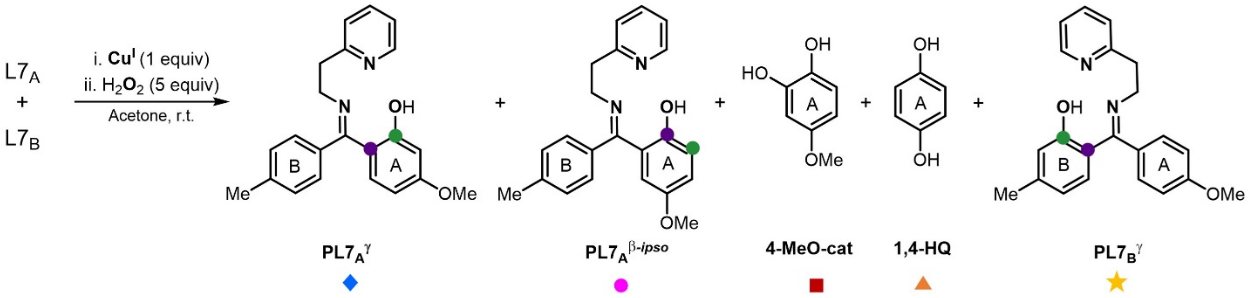

The reaction was carried out on a 0.159 mmol scale using 55.3 mg of the imine according to the Standard Procedure. The crude product was quantified using 0.159 mmol of 1,3,5-trimethoxybenzene (int. std.) (47% yield). The identity of the hydroxylation products was confirmed by $^1$H-NMR.

Note: The ratio of $A\gamma^{Oxid}/A\beta^{Oxid}$ (11/89) is calculated using the integration of CH peaks of product derived from γ C-H hydroxylation ($PL7_A^{\gamma}$) and hydroxylation products derived from β-ipso hydroxylation ($PL7_A^{\beta\text{-}ipso}$, 4-MeO-cat, and 1,4-dihydroquinone). The ratio of $A^{Oxid}/B^{Oxid}$ (81/19) is calculated using the integration of CH peaks of products derived from the oxidation of A ring ($PL7_A^{\gamma}$, $PL7_A^{\beta\text{-}ipso}$, 4-MeO-cat, and 1,4-dihydroquinone) and the oxidation product derived from the B ring ($PL7_B^{\gamma}$).

#### 4.1.2. Cleavage of DG of PL7

In a round bottom flask equipped with a stir bar, the mixture of $PL_A^{\gamma}$, $PL_A^{\beta\text{-ipso}}$, catechol, HQ and $PL_B^{\gamma}$ were dissolved using 50 mL EtOAc. To this mixture 100 mL 1M HCl was added slowly and let it react for 30 min. The resulting mixture was extracted with EtOAc (50 mL X 2). The organic phases were separated, combined, dried over $MgSO_4$, filtered, and dried under vacuum. The organic products were quantified using 0.159 mmol of 1,3,5-trimethoxybenzene (int. std.). The identity of the cleaved products was confirmed by $^1$H-NMR[2]. The identity of $P7_A^{\beta\text{-ipso}}$ was confirmed using the $^1$H-NMR reported by Le' an Hu and coworkers.[7]

Note: $P7_A^{\gamma}$ and $P7_B^{\gamma}$ were not detected.

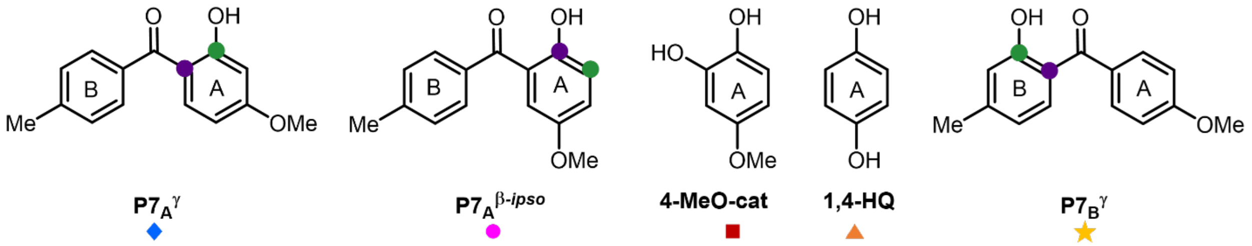

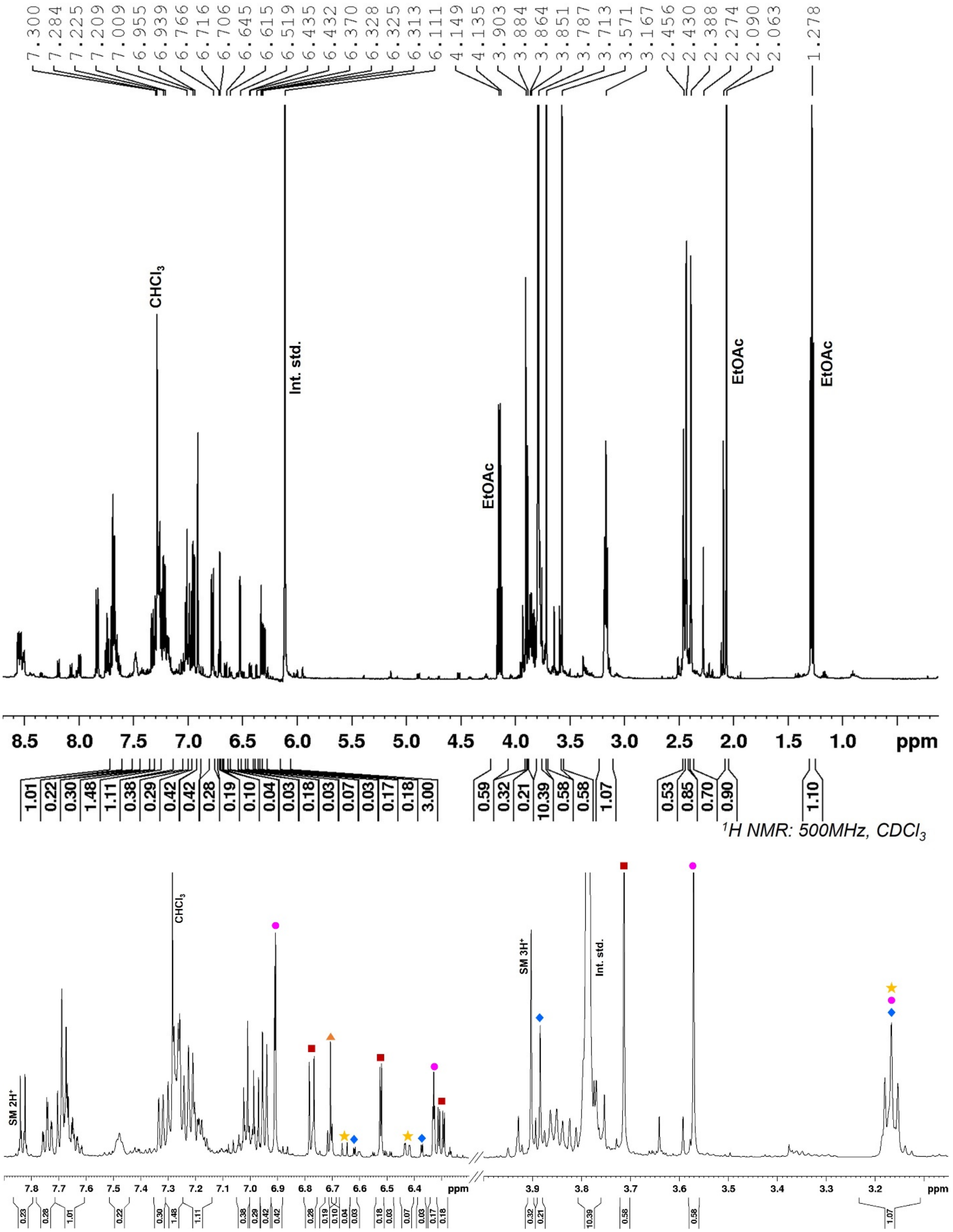


**Figure S18.** $^1$H-NMR spectra for the hydroxylation of L7.

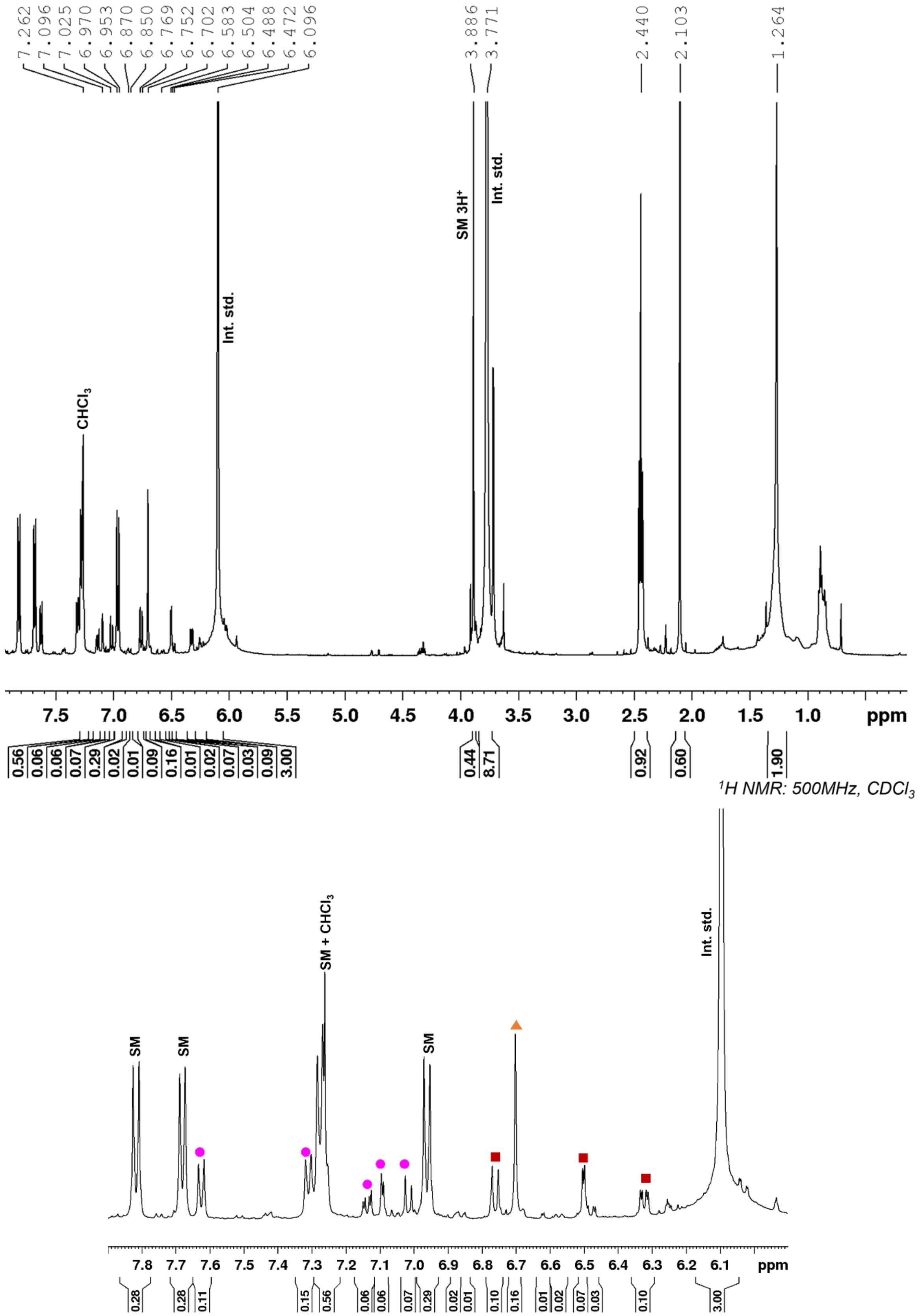


**Figure S19**. 1H-NMR spectra for the cleavage of DG of PL7

## 4.2. Synthesis of L8

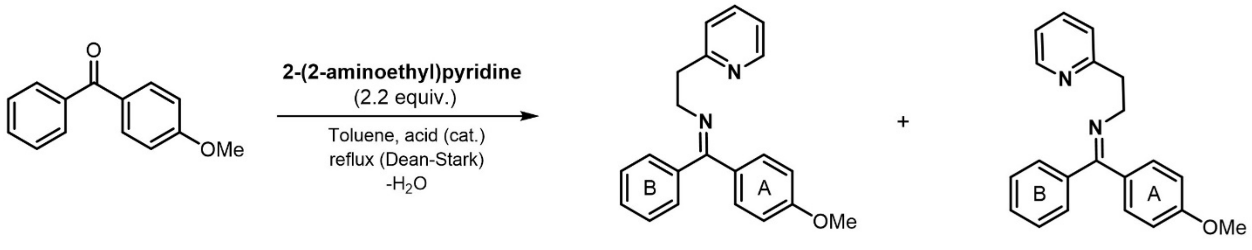


L8$_A$ ◆ L8$_B$ ★

In an oven-dried flask, 2-(2-pyridyl)ethylamine (2.60 mL, 21.7 mmol, 2.2 equiv) was added to 4-methoxybenzophenone (2.15 g, 9.85 mmol) and p-toluenesulfonic acid monohydrate (cat. 20 mg, 1.2 mol%) in toluene (50 mL). The reaction mixture was refluxed under argon with a Dean-Stark apparatus until imine formation was complete (24 h). The reaction was cooled to room temperature and diluted with diethyl ether (30 mL). The organic layer was washed with saturated ammonia chloride (50 mL x 2), saturated aqueous sodium bicarbonate (50 mL), brine (50 mL), and dried with magnesium sulfate. The final product isolated was a reddish orange solid (81% yield, 95% pure).

$^{1}$H-NMR (500 MHz, $CDCl_3$): δ 8.49 (m, $L^A$+$L^B$: 2H), 7.58-7.55 (m, $L^A$+$L^B$: 4H), 7.53-7.52 (d, $L^A$: 2H), 7.41-7.30 (m, $L^A$+$L^B$: 6H), 7.22-7.15 (m, $L^A$+$L^B$: 2H), 7.09 (m, $L^A$+$L^B$: 2H), 6.99-6.98 (m, $L^A$+$L^B$: 2H), 6.96-6.91 (d, $L^B$: 4H), 6.84 (d, $L^A$: 2H), 3.85 (s, $L^B$: 3H), 3.81 (s, $L^A$: 3H). 3.86-3.83 (t, $L^B$: 2H), 3.76-3.73 (t, $L^A$: 2H), 3.22-3.17 (t, $L^A$+$L^B$: 4H).
HRMS (ESI) m/z [M + Na]$^+$ calculated for $C_{21}H_{20}N_2O$ 316.1576, found 317.1661.
Note: The ratio of $^{4MeO4'H}L_A$/$^{4MeO4'H}L_B$ (63/37) is calculated using the average of the integration of $CH_2$ peaks and CH peaks.

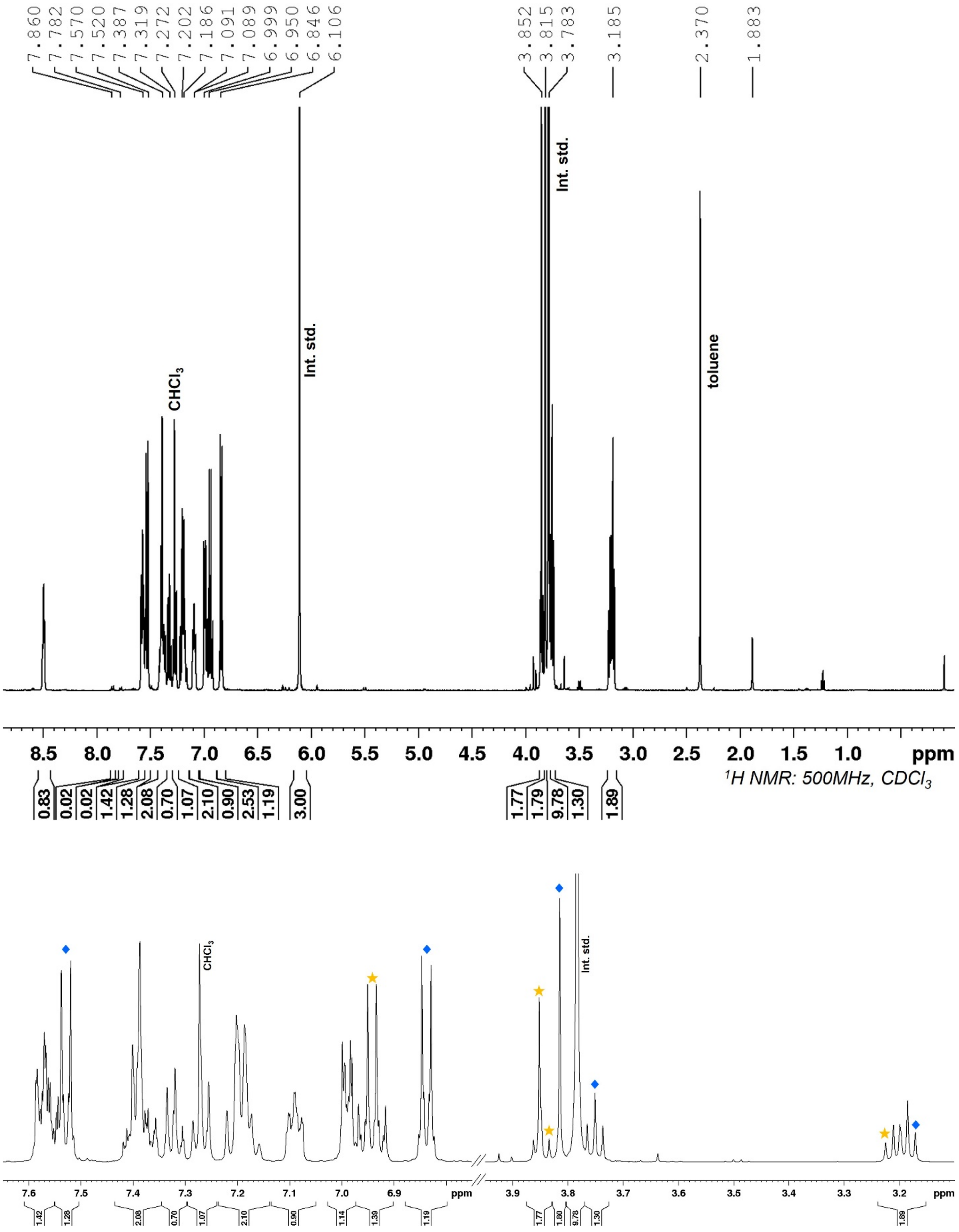


**Figure S20.** $^1$H-NMR spectra of L8.

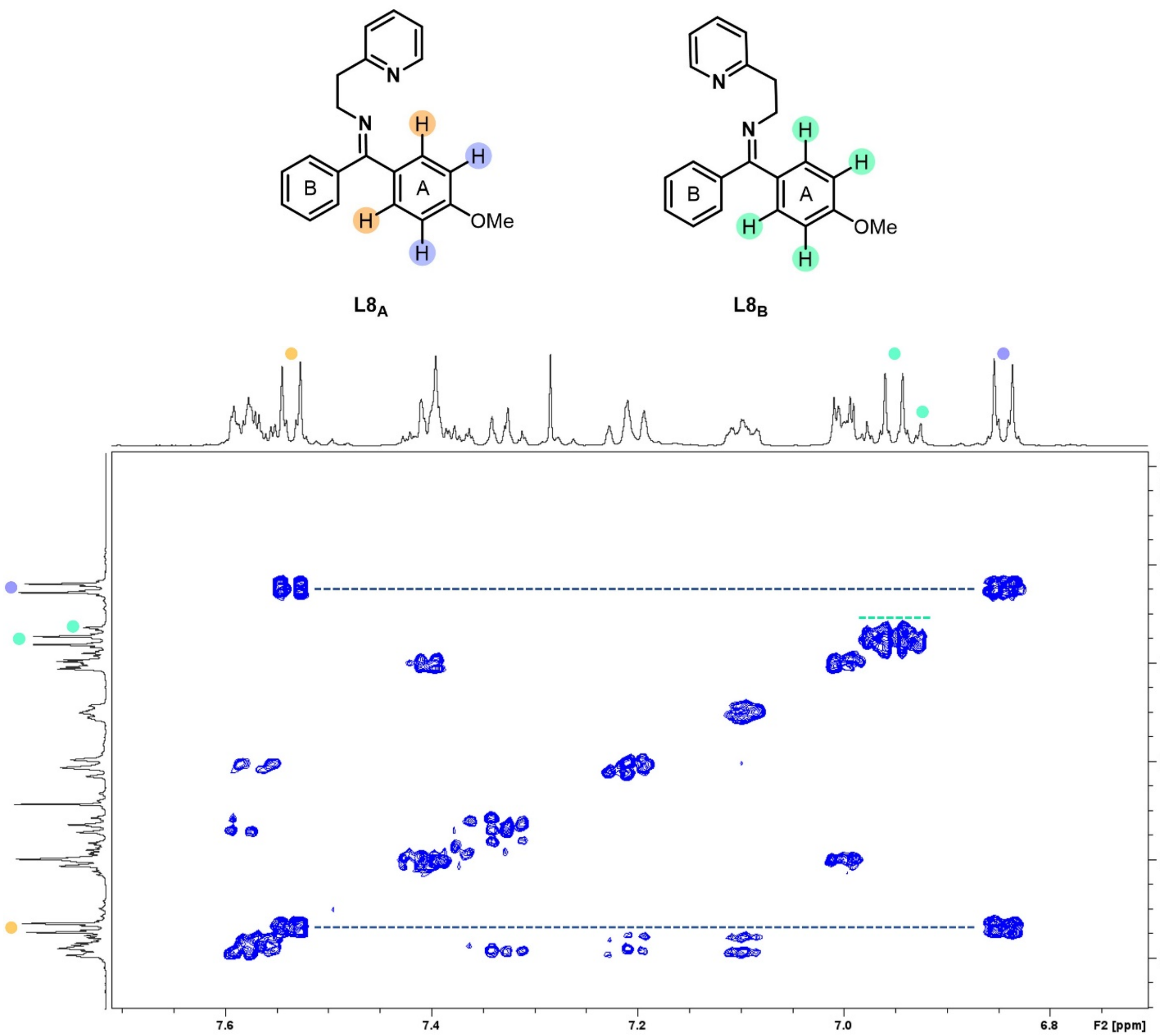


**Figure S21.** Correlation Spectroscopy (COSY) spectra of L8.

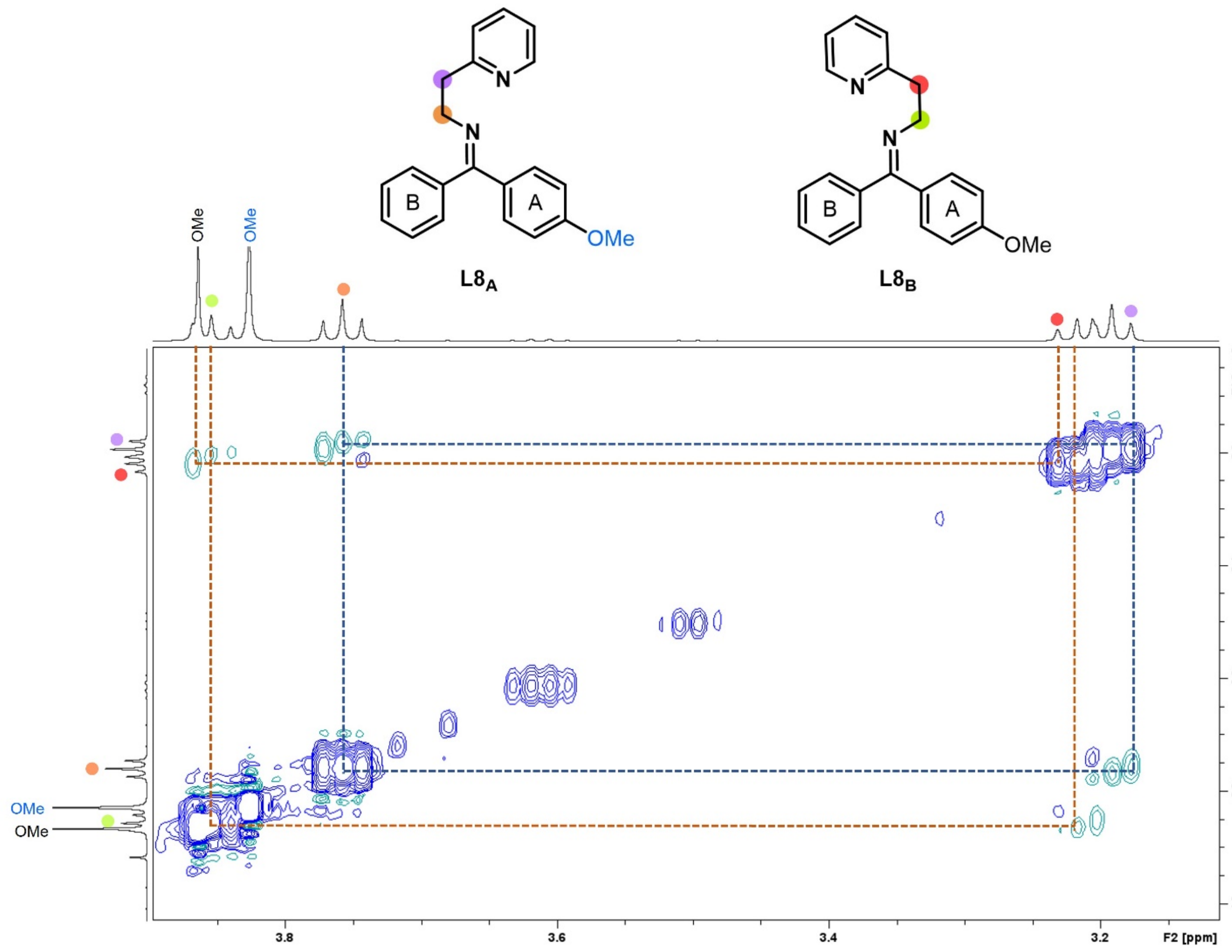


**Figure S22.** Nuclear Overhauser Effect Spectroscopy (NOESY) spectra of L8.

### 4.2.1. Hydroxylation of L8

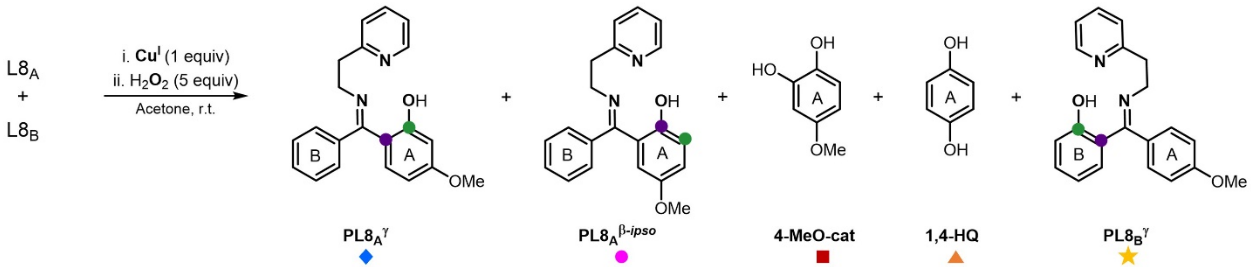


The reaction was carried out on a 0.159 mmol scale using 52.9 mg of imine according to the Standard Procedure. The brown crude product was quantified using 0.159 mmol of 1,3,5-trimethoxybenzene (int. std.) (53% yield). The identity of the hydroxylation products was confirmed by $^{1}$H-NMR. For independently synthesized $PL8_A^{\gamma}$ and $PL8_A^{\beta\text{-}ipso}$ see section 7.

Note: The ratio of $A\gamma^{Oxid}/A\beta^{Oxid}$ (14/86) is calculated using the integration of CH peaks of product derived from γ C-H hydroxylation ($PL8_A^{\gamma}$) and hydroxylation products derived from β-ipso hydroxylation ($PL8_A^{\beta\text{-}ipso}$, 4-MeO-cat, and 1,4-dihydroquinone). The ratio of $A^{Oxid}/B^{Oxid}$ (83/17) is calculated using the integration of CH peaks of products derived from the oxidation of A ring ($PL8_A^{\gamma}$, $PL8_A^{\beta\text{-}ipso}$, 4-MeO-cat, and 1,4-dihydroquinone) and the oxidation product derived from the B ring ($PL8_B^{\gamma}$).

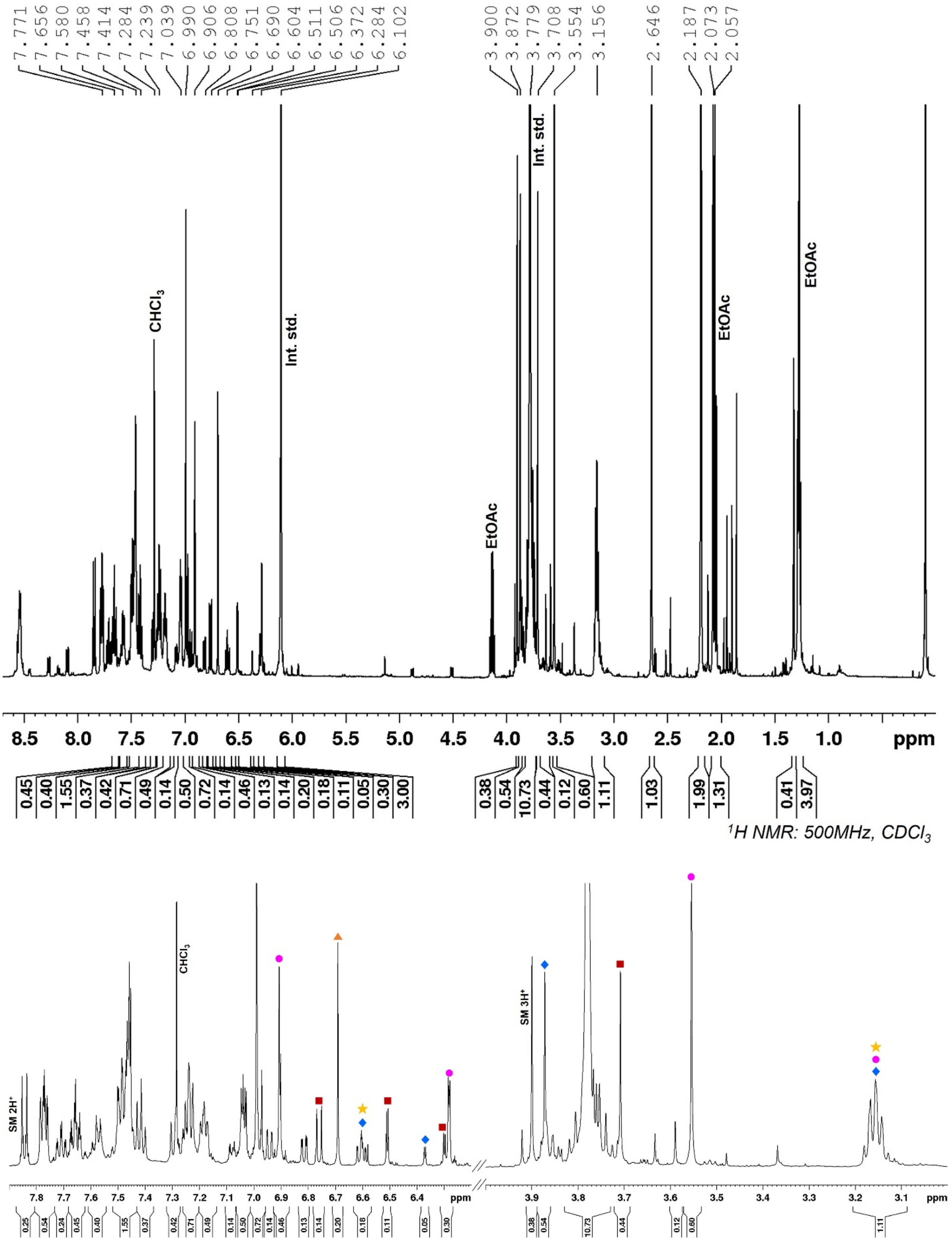


**Figure S23.** $^1$H-NMR spectra for the hydroxylation of L8.

#### 4.2.2. Cleavage of DG of PL8

In a round bottom flask equipped with a stir bar, the mixture of $PL_A^{\gamma}$, $PL_A^{\beta\text{-ipso}}$, catechol, HQ and $PL_B^{\gamma}$ were dissolved using 50 mL EtOAc. To this mixture 100 mL 1M HCl was added slowly and let it react for 30 min. The resulting mixture was extracted with EtOAc (50 mL X 2). The organic phases were separated, combined, dried over $MgSO_4$, filtered, and dried under vacuum. The organic products were quantified using 0.159 mmol of 1,3,5-trimethoxybenzene (int. std.). The identity of the cleaved products was confirmed by $^1$H-NMR.[2]

Note: The ratio of $A\gamma^{Oxid}/A\beta^{Oxid}$ (8/92) is calculated using the integration of CH peaks of cleaved product derived from γ C-H hydroxylation ($P8_A^{\gamma}$) and cleaved products derived from β-ipso hydroxylation ($P8_A^{\beta\text{-}ipso}$, 4-MeO-cat, and 1,4-dihydroquinone). The ratio of $A^{Oxid}/B^{Oxid}$ (92/8) is calculated using the integration of CH peaks of cleaved products derived from the oxidation of A ring ($P8_A^{\gamma}$, $P8_A^{\beta\text{-}ipso}$, 4-MeO-cat, and 1,4-dihydroquinone) and the cleaved oxidation product derived from the B ring ($P8_B^{\gamma}$).

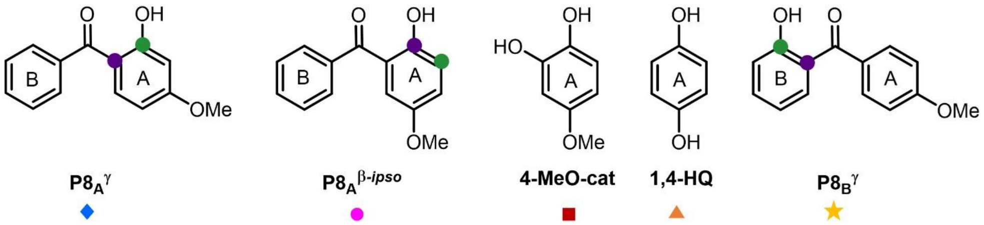

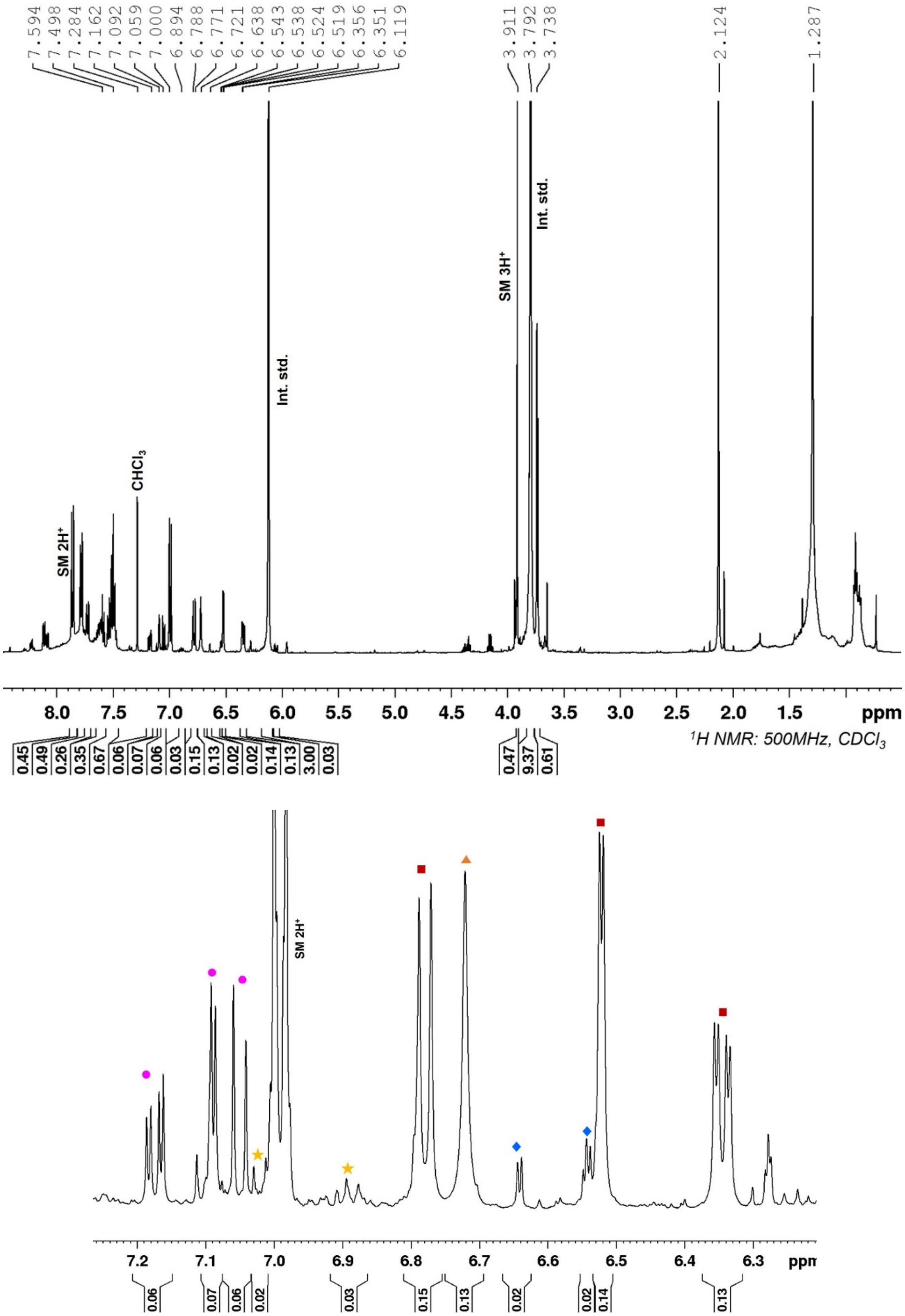


**Figure S24.** $^{1}$H-NMR spectra for the cleavage of DG of PL8.

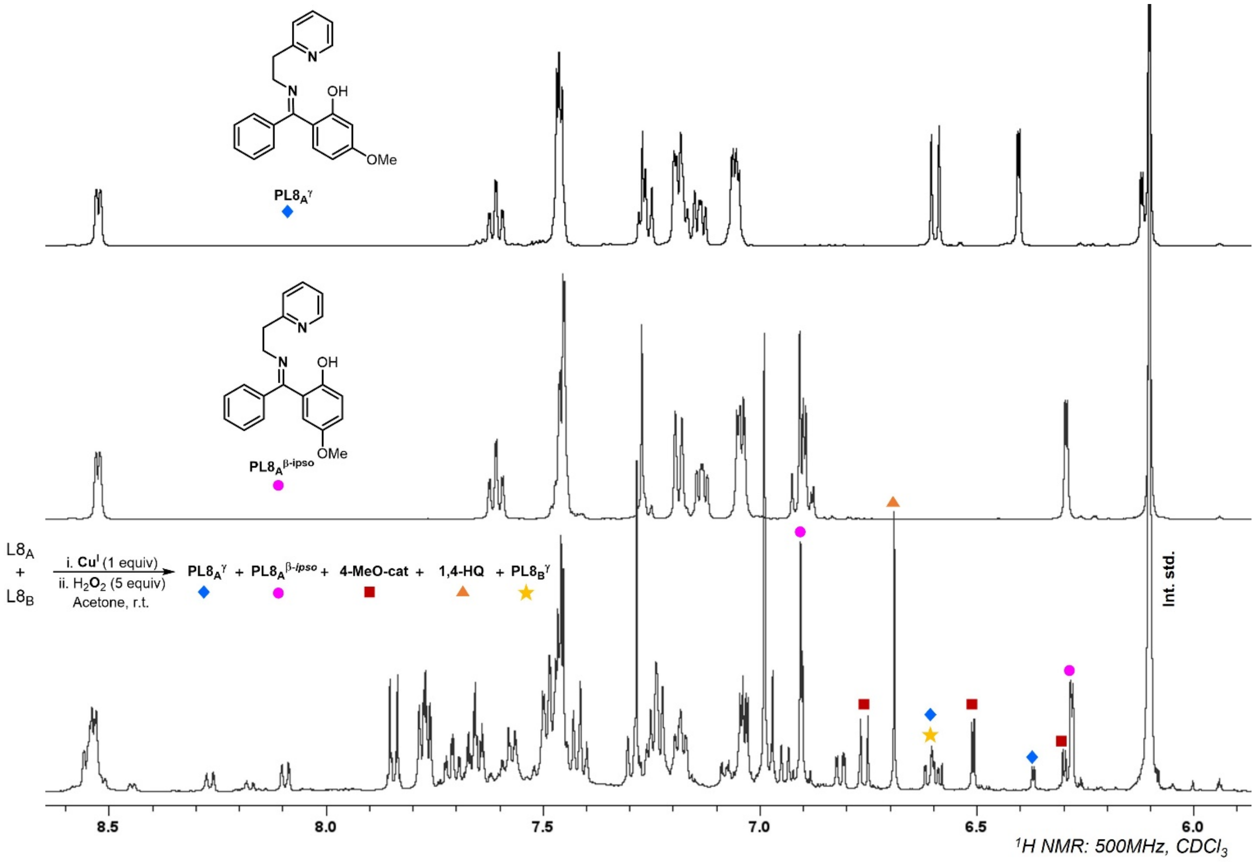


**Figure 25:** Stacked $^1$H-NMR spectra of the reaction mixture for hydroxylation of L8 with independently synthesized $PL8_A^{\beta\text{-ipso}}$ and $PL8_A^{\gamma}$.

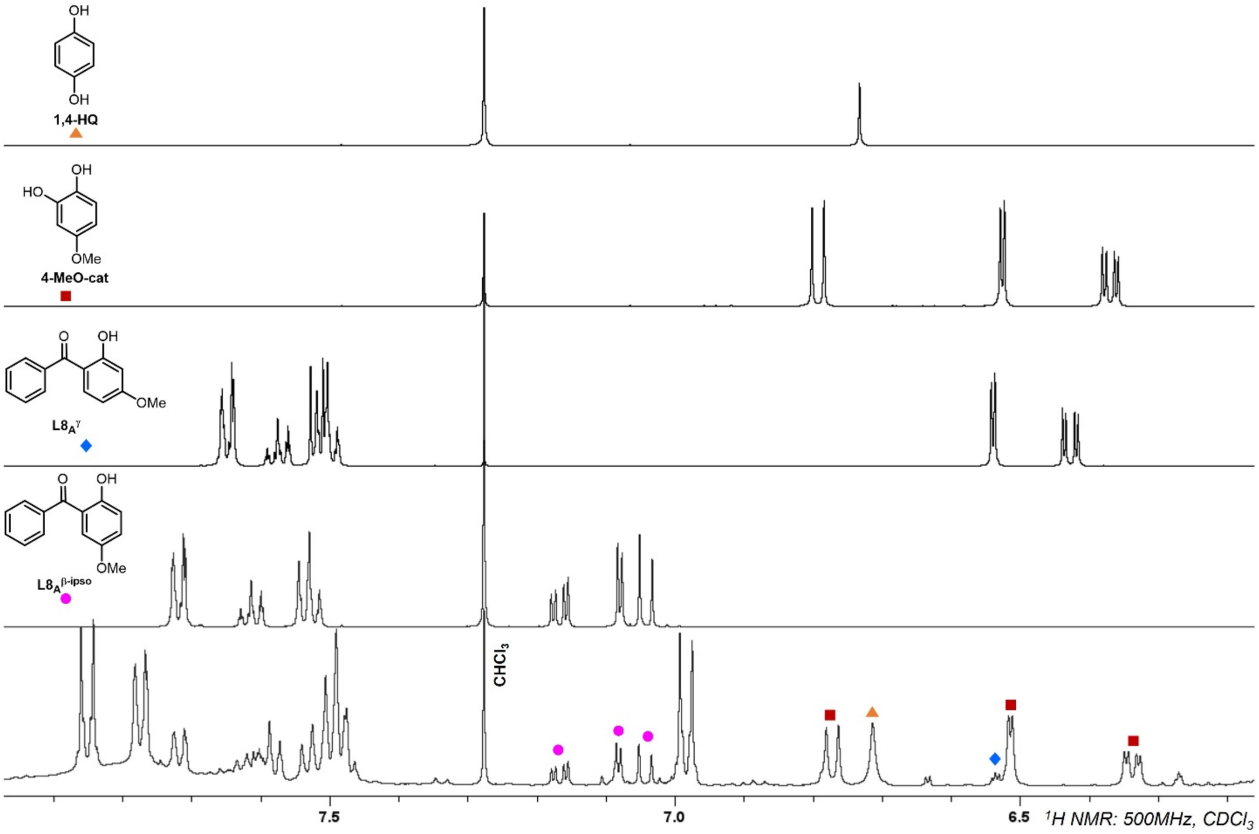


**Figure 26:** Stacked $^1$H-NMR spectra of the reaction mixture after the cleavage of DG of PL8 with $P8_A^{\beta\text{-ipso}}$, $P8_A^{\gamma}$, 4-MeO-catechol, and 1,4-HQ.

## 4.3. Synthesis of L9

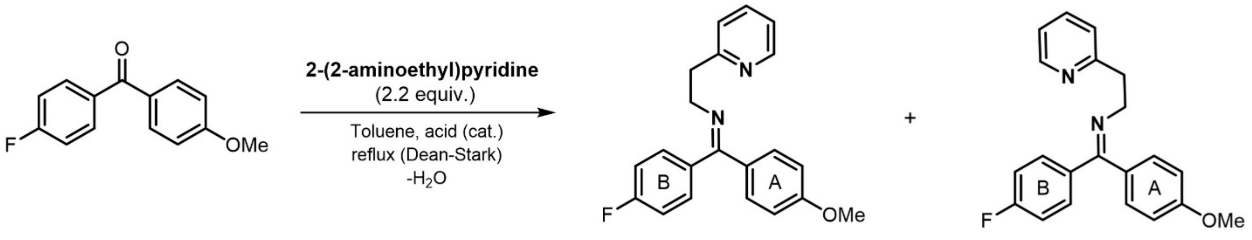


L9A ◆ L9B ★

In an oven-dried flask, 2-(2-pyridyl)ethylamine (2.60 mL, 21.7 mmol, 2.2 equiv) was added to 4-methoxy-4'- fluorobenzophenone (2.31 g, 9.85 mmol) and p-toluenesulfonic acid monohydrate (cat. 20 mg, 1.2 mol%) in toluene (50 mL). The reaction mixture was refluxed under argon with a Dean-Stark apparatus until imine formation was complete (24 h). The reaction was cooled to room temperature and diluted with diethyl ether (30 mL). The organic layer was washed with saturated ammonia chloride (50 mL x 2), saturated aqueous sodium bicarbonate (50 mL), brine (50 mL), and dried with magnesium sulfate. The final product isolated was an orangish yellow solid (71% yield, 92% pure).

$^{1}$H-NMR (500 MHz, $CDCl_3$): δ 8.50 (m, $L^A$+$L^B$: 2H), 7.58-7.55 (m, $L^A$+$L^B$: 4H), 7.52-7.50 (d, $L^A$: 2H), 7.20-7.18(m, $L^A$+$L^B$: 2H), 7.11-7.08 (m, $L^A$+$L^B$: 4H), 7.01-6.95 (m, $L^A$+$L^B$: 4H),-6.94 (d, $L^B$: 4H), 6.85-6.83 (d, $L^A$: 2H), 3.86 (s, $L^B$: 3H), 3.84-3.81 (m, $L^A$+$L^B$: 5H), 3.77-3.74 (t, $L^A$: 2H), 3.21-3.17 (t, $L^A$+$L^B$: 4H).

HRMS (ESI) m/z $[M + Na]^+$ calculated for $C_{21}H_{19}FN_2O$ 334.1481, found 335.1571.

Note: The ratio of $L9_A$/$L9_B$ (52/48) is calculated using the average of the integration of $CH_2$ peaks and CH peaks.

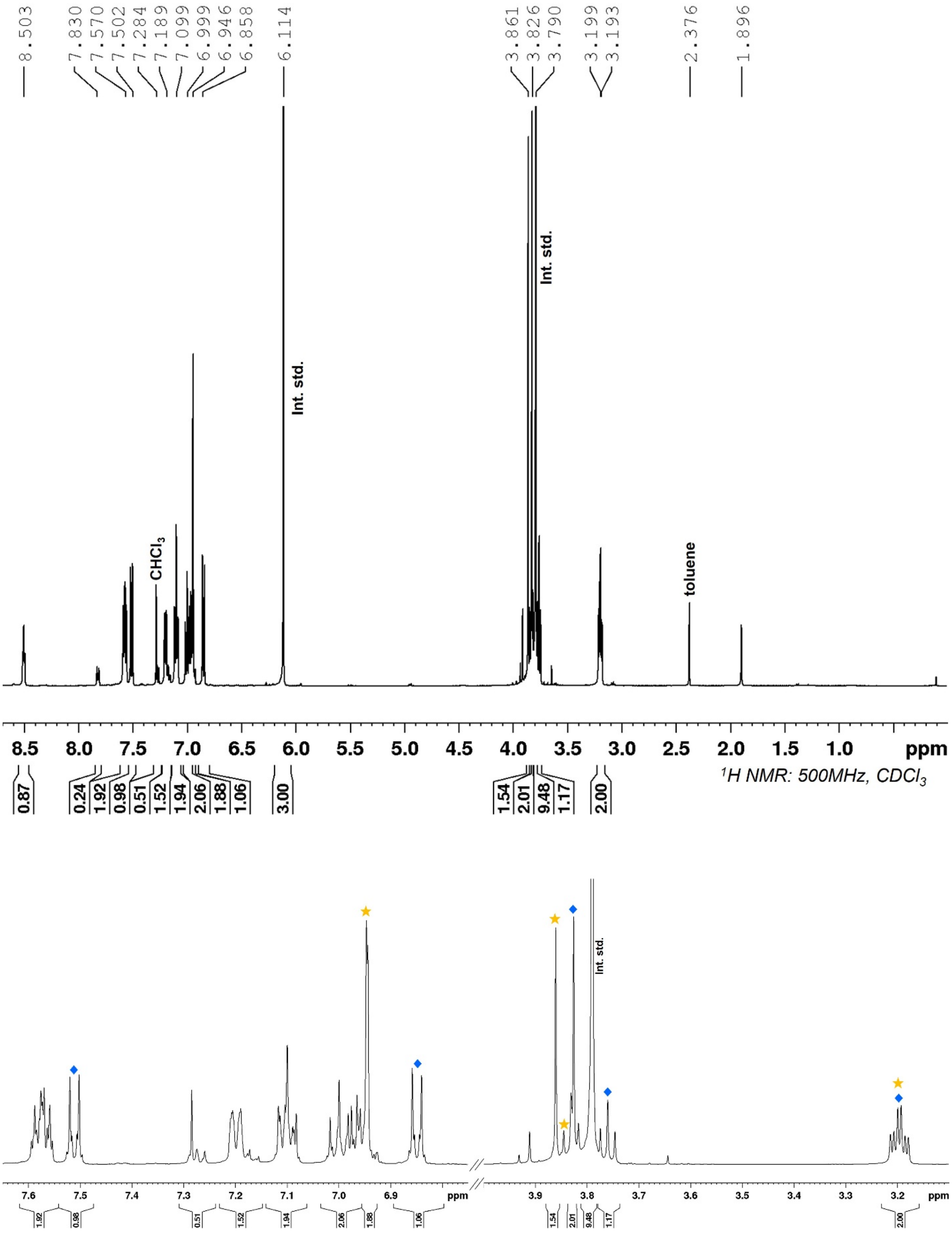


**Figure S27.** $^{1}$H-NMR spectra of L9.

### 4.3.1. Hydroxylation of L9

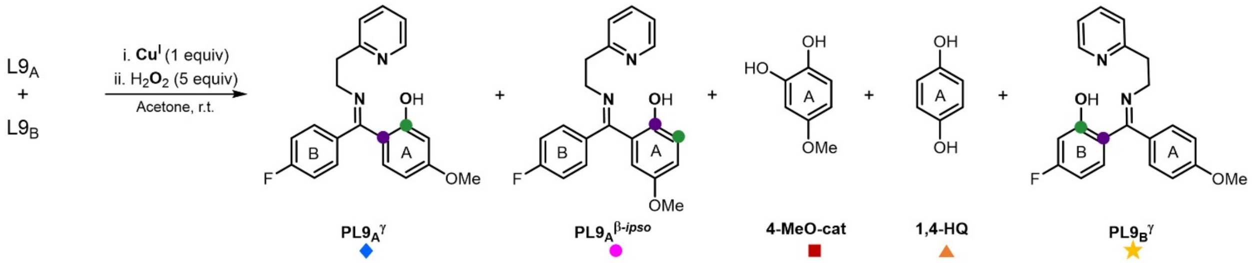

The reaction was carried out on a 0.159 mmol scale using 54.0 mg of imine according to the Standard Procedure. The crude product was quantified using 0.159 mmol of 1,3,5-trimethoxybenzene (int. std.) (42% yield). The identity of the hydroxylation products was confirmed by $^1$H-NMR.

Note: The ratio of A$\gamma^{Oxid}$/A$\beta^{Oxid}$ (8/92) is calculated using the integration of CH peaks of product derived from γ C-H hydroxylation (PL9$_A^{\gamma}$) and hydroxylation products derived from β-ipso hydroxylation (PL9$_A^{\beta\text{-}ipso}$, 4-MeO-cat, and 1,4-dihydroquinone). The ratio of A$^{Oxid}$/B$^{Oxid}$ (82/12) is calculated using the integration of CH peaks of products derived from the oxidation of A ring (PL9$_A^{\gamma}$, PL9$_A^{\beta\text{-}ipso}$, 4-MeO-cat, and 1,4-dihydroquinone) and the oxidation product derived from the B ring (PL9$_B^{\gamma}$).

### 4.3.2. Cleavage of DG of PL9

In a round bottom flask equipped with a stir bar, the mixture of PL$_A^{\gamma}$, PL$_A^{\beta\text{-ipso}}$, catechol, HQ, and PL$_B^{\gamma}$ were dissolved using 50 mL EtOAc. To this mixture 100 mL 1M HCl was added slowly and let it react for 30 min. The resulting mixture was extracted with EtOAc (50 mL X 2). The organic phases were separated, combined, dried over $MgSO_4$, filtered, and dried under vacuum. The organic products were quantified using 0.159 mmol of 1,3,5-trimethoxybenzene (int. std.). The identity of the cleaved products was confirmed by $^1$H-NMR.[2] The identity of P9$_A^{\beta\text{-ipso}}$ was confirmed using the $^1$H-NMR reported by Jun Hu and coworkers.[8]

Note: P9$_A^{\gamma}$ and P9$_B^{\gamma}$ were not detected.

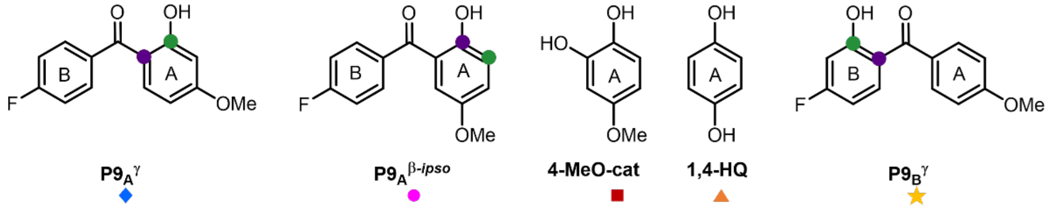

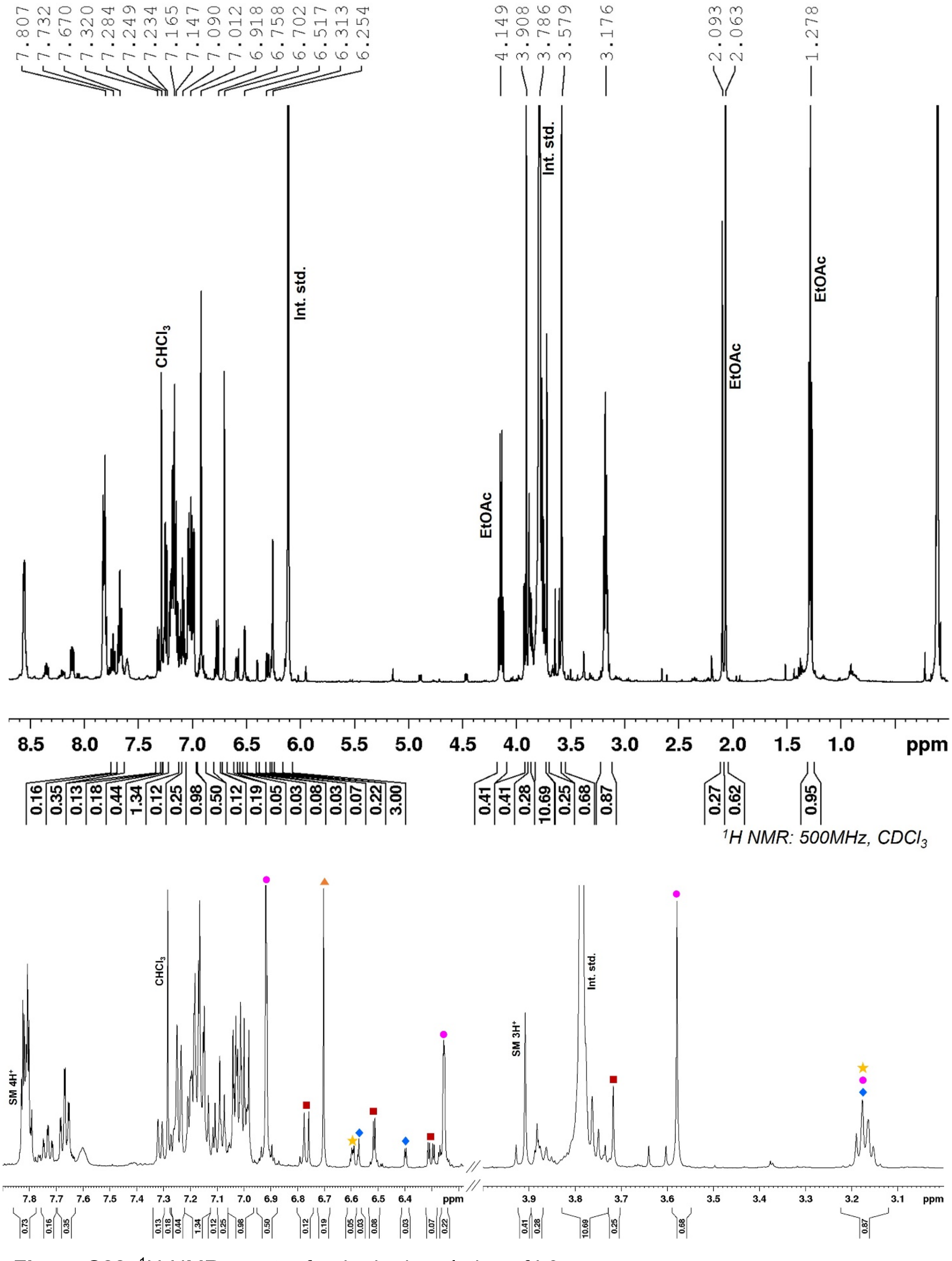


**Figure S28.** $^{1}$H-NMR spectra for the hydroxylation of L9.

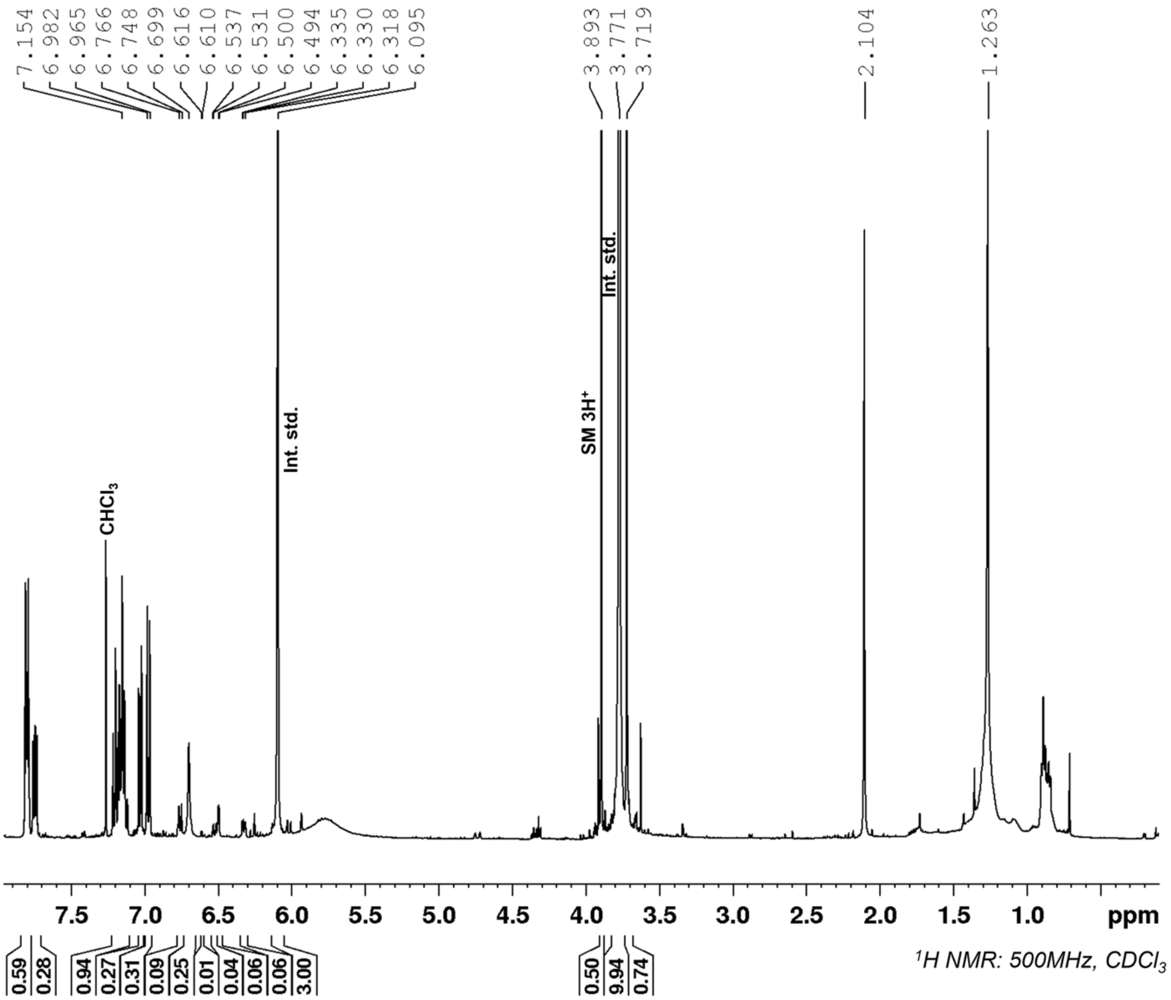


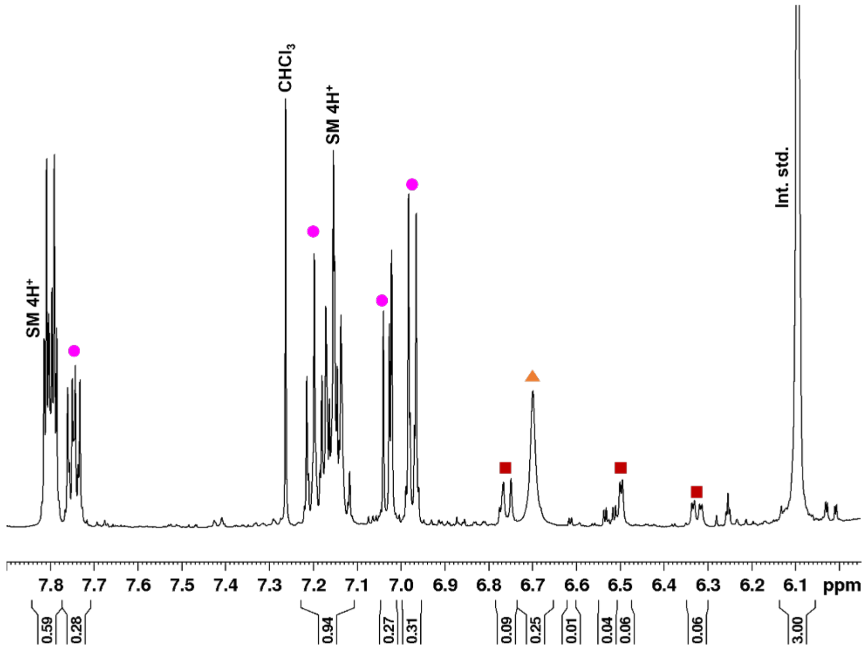


**Figure S29**. 1H-NMR spectra for the DG Cleavage of PL9.

## 4.4. Synthesis of L10

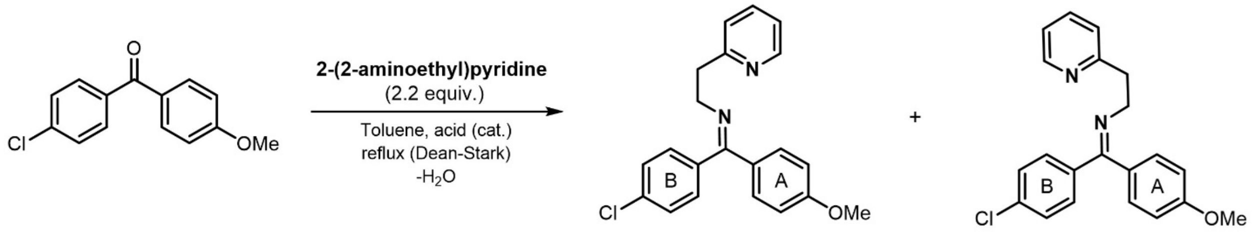


$L10_A$ ◆ $L10_B$ ★

In an oven-dried flask, 2-(2-pyridyl)ethylamine (1.5 mL, 10.8 mmol, 2.2 equiv) was added to 4-methoxy-4'-chlorobenzophenone (1.24 g, 4.9 mmol) and p-toluenesulfonic acid monohydrate (cat. 11 mg, 1.2 mol%) in toluene (30 mL). The reaction mixture was refluxed under argon with a Dean-Stark apparatus until imine formation was complete (24 h). The reaction was cooled to room temperature and diluted with diethyl ether (20 mL). The organic layer was washed with saturated ammonia chloride (30 mL x 2), saturated aqueous sodium bicarbonate (30 mL), brine (30 mL), and dried with magnesium sulfate. The final product isolated was a reddish-orange solid (91% yield, 99% pure).

$^1$H-NMR (500 MHz, $CDCl_3$): δ 8.49 (d, $L^A$+$L^B$: 2H), 7.58-7.55 (m, $L^A$+$L^B$: 2H), 7.51-7.49 (m, $L^A$+$L^B$: 4H), 7.38-7.37 (d, $L^A$: 2H), 7.19 (d, $L^A$+$L^B$: 4H), 7.11-7.08 (m, $L^A$+$L^B$: 2H), 6.93-6.90 (m, $L^A$+$L^B$: 6H), 6.83 (d, $L^A$: 2H), 3.85 (s, $L^B$: 3H), 3.84-3.82 (m, $L^A$+$L^B$: 5H). 3.75-3.72 (t, $L^A$: 2H), 3.20-3.17 (m, $L^A$+$L^B$: 4H).

HRMS (ESI) m/z $[M + Na]^+$ calculated for $C_{21}H_{19}ClN_2O$ 350.1186, found 351.1292.

Note: The ratio of $L10_A/L10_B$ (52/48) is calculated using the average of the integration of $CH_2$ peaks and CH peaks.

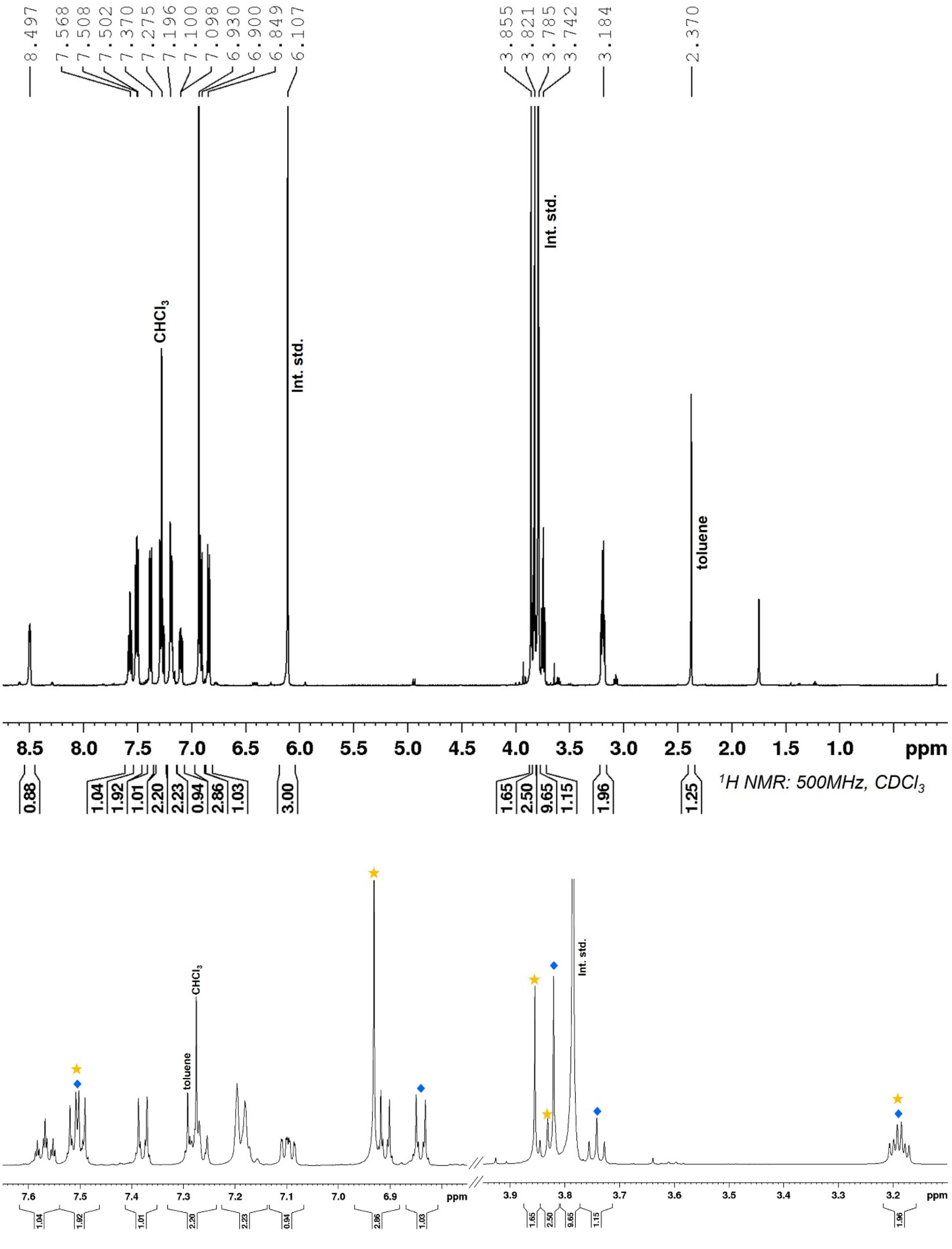


**Figure S30.** $^{1}$H-NMR spectra of L10.

### 4.4.1. Hydroxylation of L10

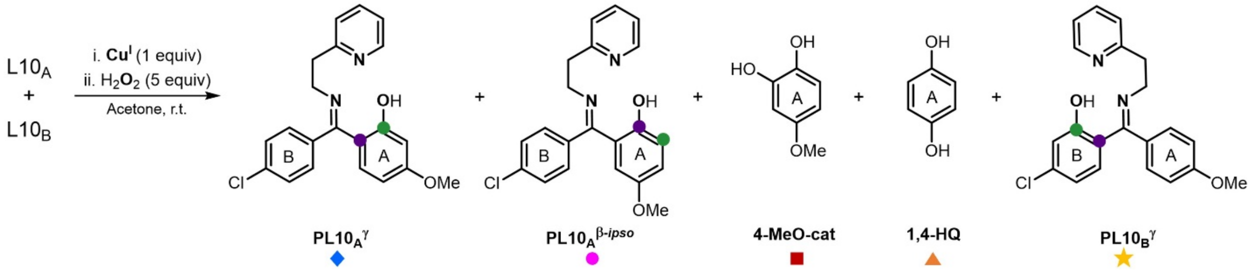


The reaction was carried out on a 0.159 mmol scale using 56.3 mg of imine according to the Standard Procedure. The crude product was quantified using 0.159 mmol of 1,3,5-trimethoxybenzene (int. std.) (47% yield). The identity of the hydroxylation products was confirmed by $^{1}$H-NMR.

Note: The ratio of $A\gamma^{Oxid}/A\beta^{Oxid}$ (12/88) is calculated using the integration of CH peaks of product derived from γ C-H hydroxylation ($PL10_A^{\gamma}$) and hydroxylation products derived from β-ipso hydroxylation ($PL10_A^{\beta\text{-}ipso}$, 4-MeO-cat, and 1,4-dihydroquinone). The ratio of $A^{Oxid}/B^{Oxid}$ (85/15) is calculated using the integration of CH peaks of products derived from the oxidation of A ring ($PL10_A^{\gamma}$, $PL10_A^{\beta\text{-}ipso}$, 4-MeO-cat, and 1,4-dihydroquinone) and the oxidation product derived from the B ring ($PL10_B^{\gamma}$).

### 4.4.2. Cleavage of DG of PL10

In a round bottom flask equipped with a stir bar, the mixture of $PL_A^{\gamma}$, $PL_A^{\beta\text{-ipso}}$, catechol, HQ, and $PL_B^{\gamma}$ were dissolved using 50 mL EtOAc. To this mixture 100 mL 1M HCl was added slowly and let it react for 30 min. The resulting mixture was extracted with EtOAc (50 mL X 2). The organic phases were separated, combined, dried over $MgSO_4$, filtered, and dried under vacuum. The organic products were quantified using 0.159 mmol of 1,3,5-trimethoxybenzene (int. std.). The identity of the cleaved products was confirmed by $^{1}$H-NMR.[2] The identity of $P10_A^{\beta\text{-ipso}}$ was confirmed using the $^{1}$H-NMR reported by Feberero and coworkers.[9]

Note: $P10_A^{\gamma}$ and $P10_B^{\gamma}$ were not detected.

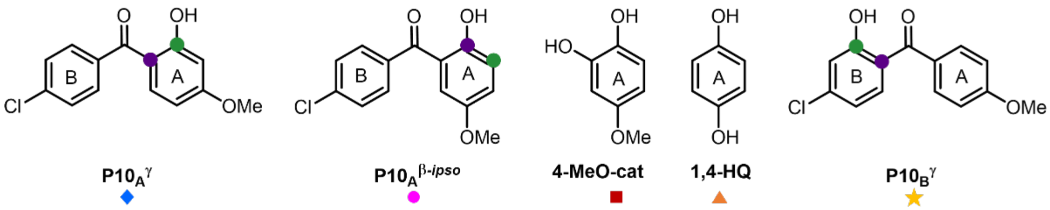

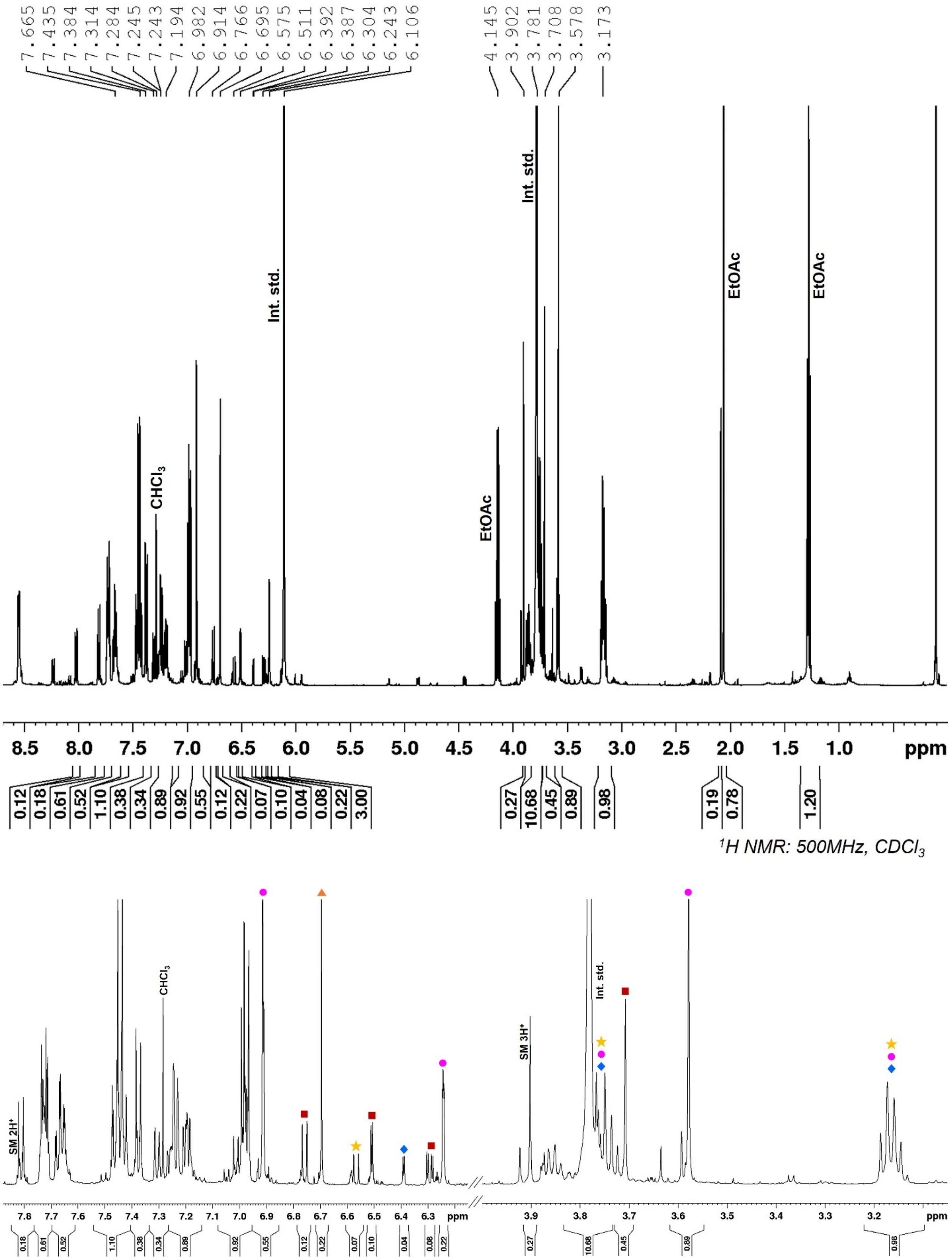


**Figure S31.** $^1$H-NMR spectra for the hydroxylation of L10.

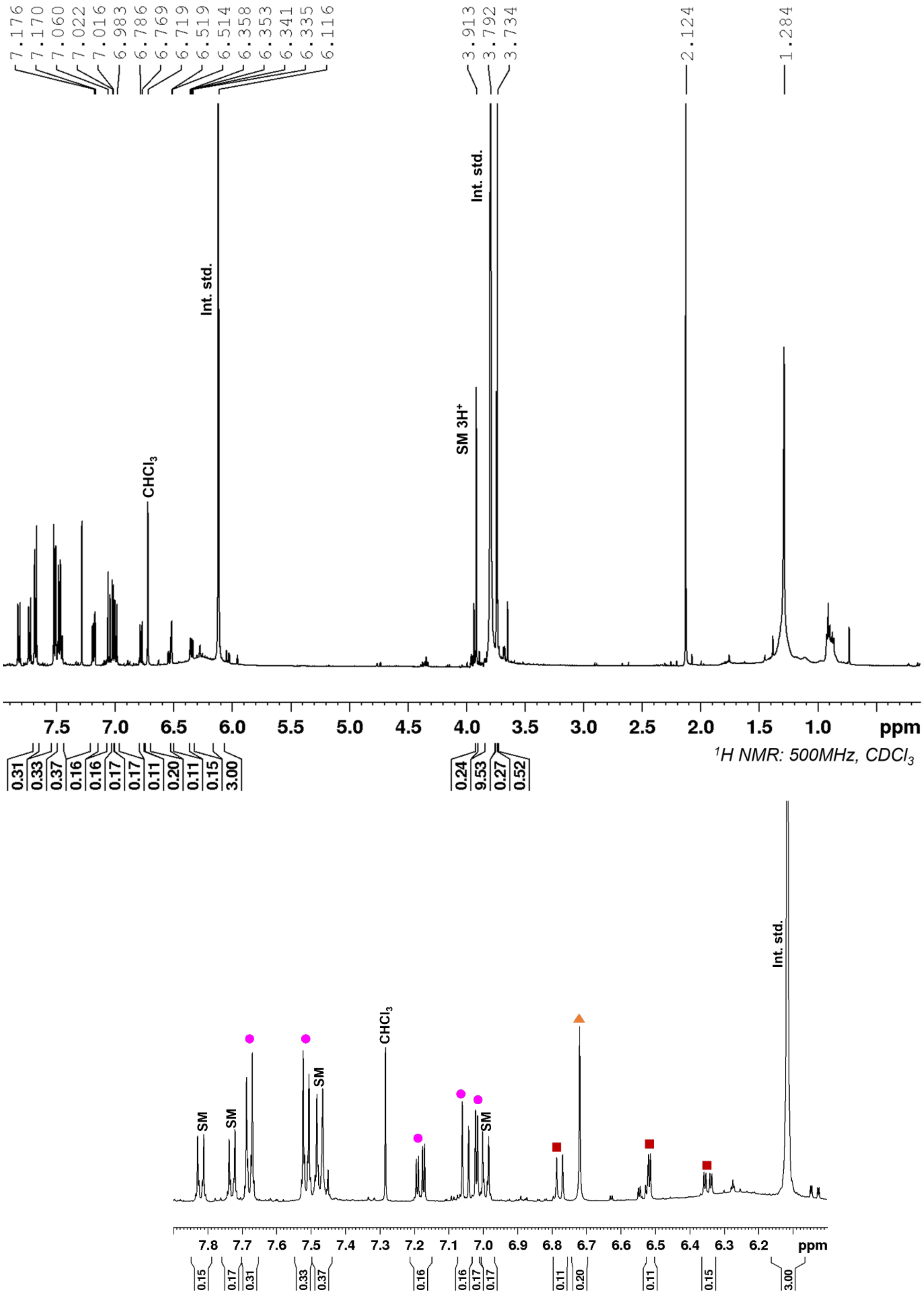


**Figure S32**. $^{1}$H-NMR spectra for the cleavage of DG of PL10.

### 4.5. Synthesis of L11

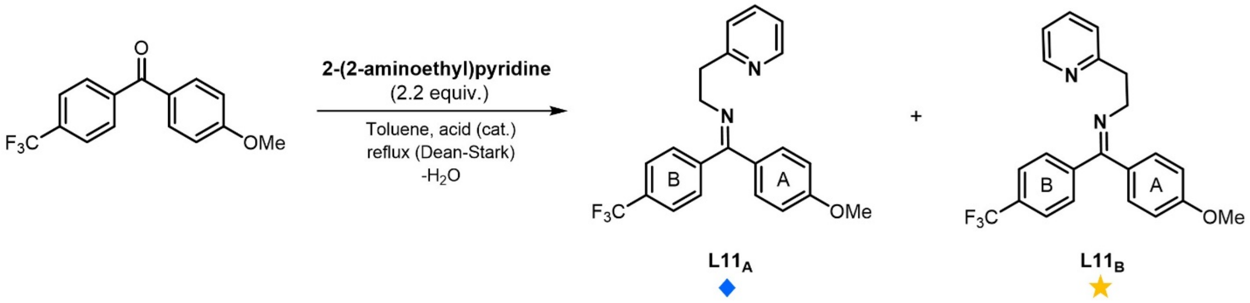


In an oven-dried flask, 2-(2-pyridyl)ethylamine (0.061 mL, 4.8 mmol, 2.2 equiv) was added to 4-methoxy-4'- trifluoromethylbenzophenone (619 mg, 2.2 mmol) and p-toluenesulfonic acid monohydrate (cat. 10 mg, 2.4 mol%) in toluene (30 mL). The reaction mixture was refluxed under argon with a Dean-Stark apparatus until imine formation was complete (24 h). The reaction was cooled to room temperature and diluted with diethyl ether (20 mL). The organic layer was washed with saturated ammonia chloride (30 mL x 2), saturated aqueous sodium bicarbonate (30 mL), brine (30 mL), and dried with magnesium sulfate. The final product isolated was a reddish orange solid (66% yield, 95% pure).

$^1$H-NMR (500 MHz, $CDCl_3$): δ 8.50 (d, $L^A$+$L^B$: 2H), 7.69-7.66 (m, $L^A$+$L^B$: 4H), 7.58-7.56 (m, $L^A$+$L^B$: 4H), 7.49-7.48 (d, $L^A$: 2H), 7.21-7.18 (d, $L^A$+$L^B$: 2H), 7.12-7.07 (m, $L^A$+$L^B$: 4H), 6.95 (d, 4H), 6.86-6.84 (d, $L^A$: 2H), 3.90-3.87 (t, $L^B$: 2H), 3.86 (s, $L^B$: 3H), 3.82 (s, $L^A$: 3H), 3.72 (t, $L^A$: 2H), 3.23-3.18 (m, $L^A$+$L^B$: 4H).

HRMS (ESI) m/z [M + Na]$^+$ calculated for $C_{22}H_{19}F_3N_2O$ 384.1449, found 385.1570.

Note: The ratio of $L11_A$/$L11_B$ (57/43) is calculated using the average of the integration of $CH_2$ peaks and CH peaks.

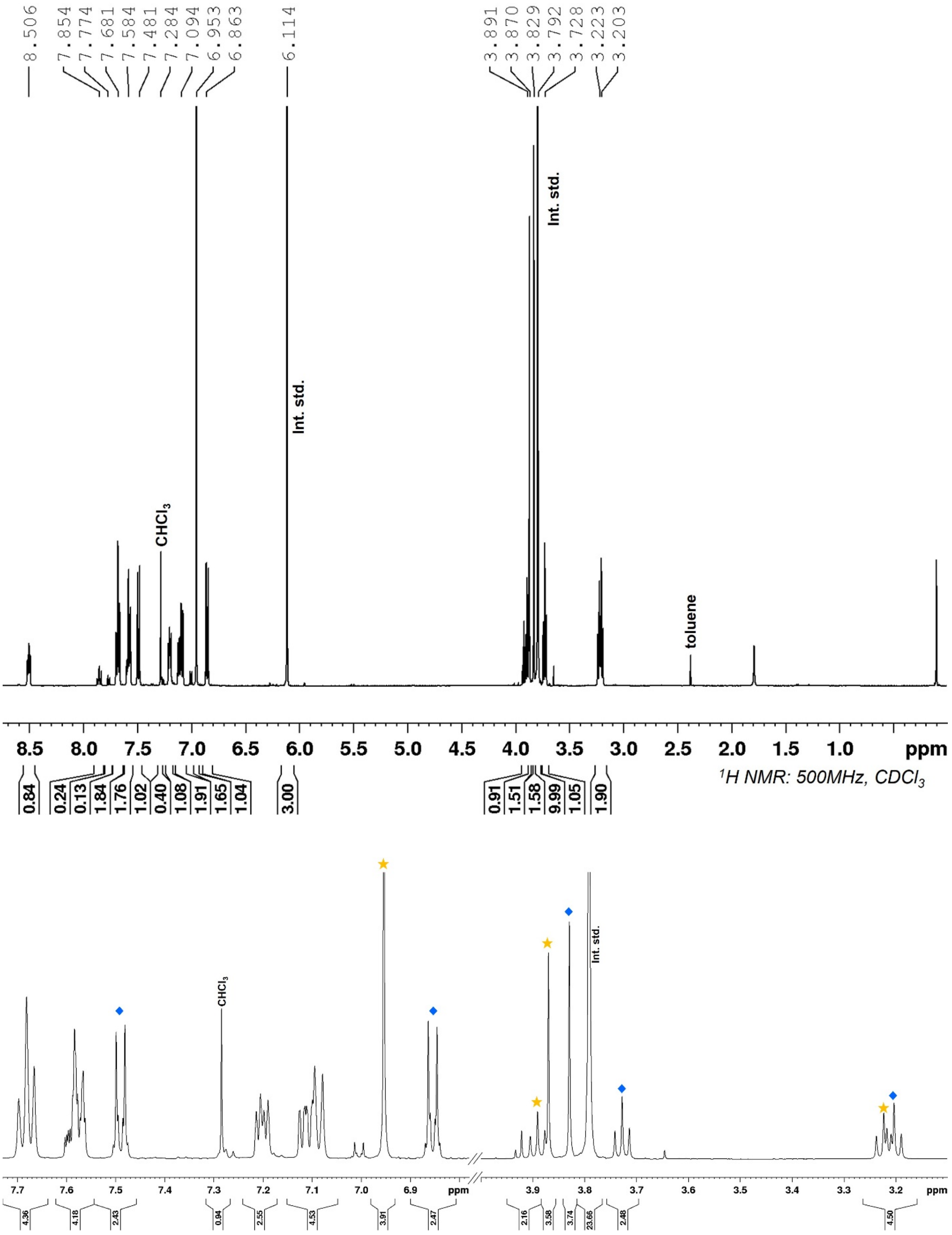


**Figure S33.** $^1$H-NMR spectra of L11.

### 4.5.1. Hydroxylation of L11

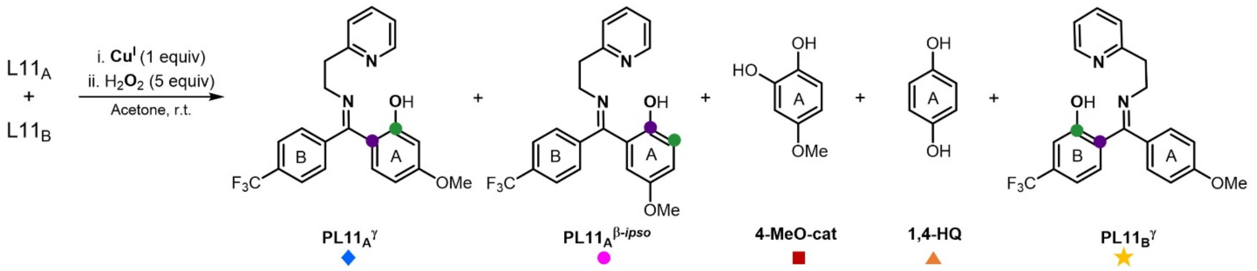


The reaction was carried out on a 0.159 mmol scale using 64.2 mg of imine according to the Standard Procedure. The crude product was quantified using 0.159 mmol of 1,3,5-trimethoxybenzene (int. std.) (45% yield). The identity of the hydroxylation products was confirmed by $^1$H-NMR.

Note: The ratio of $A\gamma^{Oxid}/A\beta^{Oxid}$ (12/88) is calculated using the integration of CH peaks of product derived from γ C-H hydroxylation ($PL11_A^{\gamma}$) and hydroxylation products derived from β-ipso hydroxylation ($PL11_A^{\beta\text{-}ipso}$, 4-MeO-cat, and 1,4-dihydroquinone). The ratio of $A^{Oxid}/B^{Oxid}$ (89/11) is calculated using the integration of CH peaks of products derived from the oxidation of A ring ($PL11_A^{\gamma}$, $PL11_A^{\beta\text{-}ipso}$, 4-MeO-cat, and 1,4-dihydroquinone) and the oxidation product derived from the B ring ($PL11_B^{\gamma}$).

### 4.5.2. Cleavage of DG of PL11

In a round bottom flask equipped with a stir bar, the mixture of $PL_A^{\gamma}$, $PL_A^{\beta\text{-}ipso}$, catechol, HQ, and $PL_B^{\gamma}$ were dissolved using 50 mL EtOAc. To this mixture 100 mL 1M HCl was added slowly and let it react for 30 min. The resulting mixture was extracted with EtOAc (50 mL X 2). The organic phases were separated, combined, dried over $MgSO_4$, filtered, and dried under vacuum. The organic products were quantified using 0.159 mmol of 1,3,5-trimethoxybenzene (int. std.). The identity of the cleaved products was confirmed by $^1$H-NMR[2]. $^1$H-NMR for $P11_A^{\beta\text{-}ipso}$ is being reported for the first time.

Note: $P11_A^{\gamma}$ and $P11_B^{\gamma}$ were not detected.

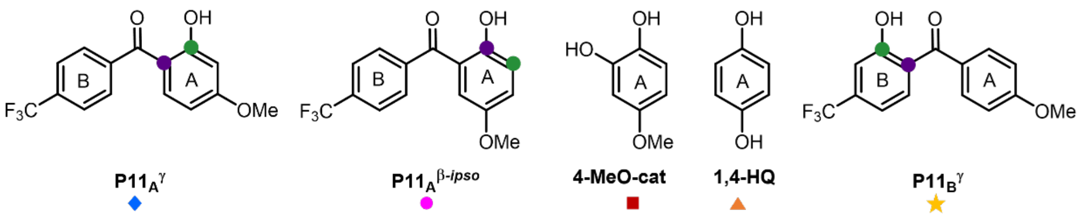

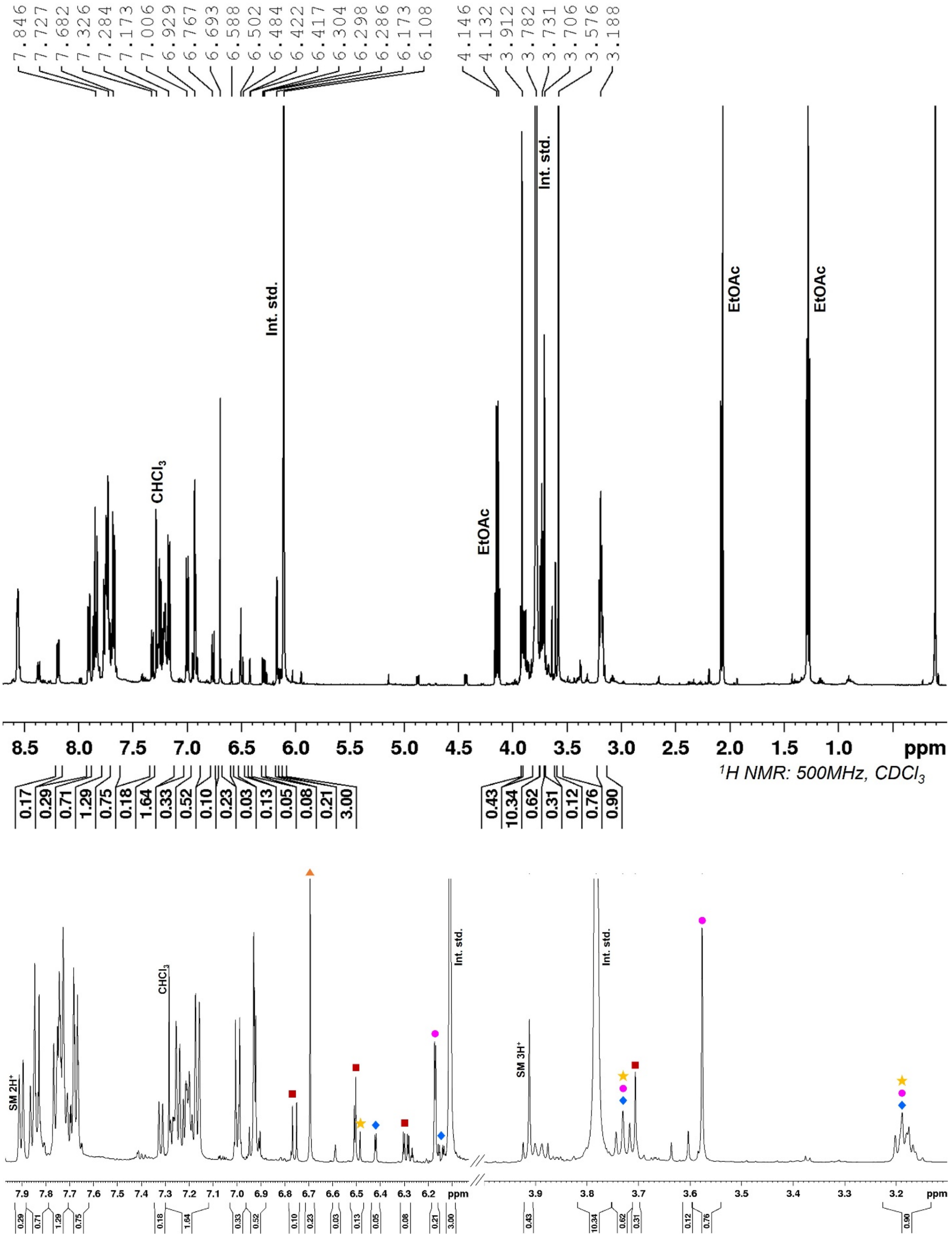


**Figure S34.** $^{1}$H-NMR spectra for the hydroxylation of L11.

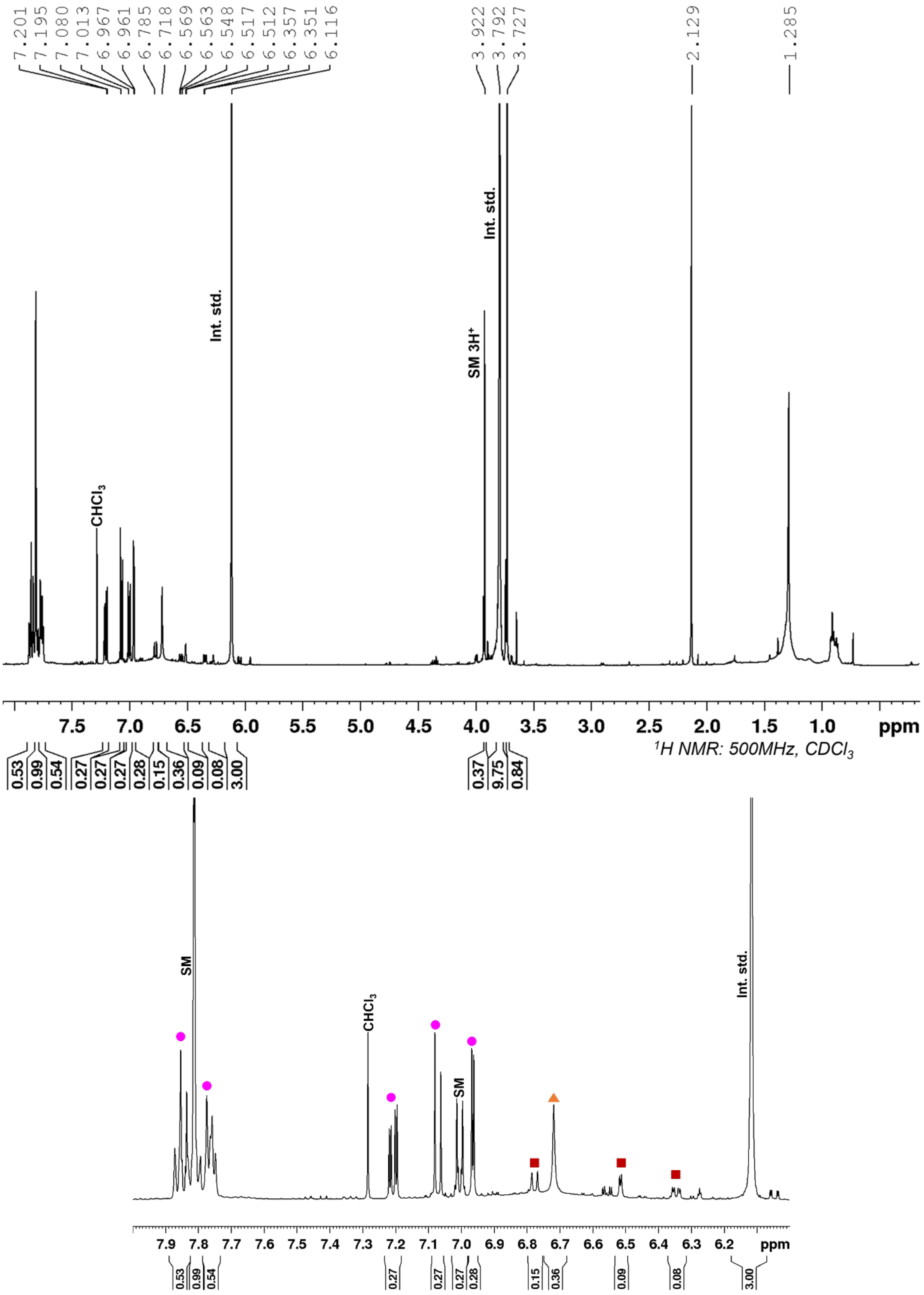


**Figure S35.** $^{1}$H-NMR spectra for the cleavage of DG of PL11.

## 5. Synthesis and hydroxylation of the deuterated imine substrate-ligands

### 5.1. Synthesis of L12

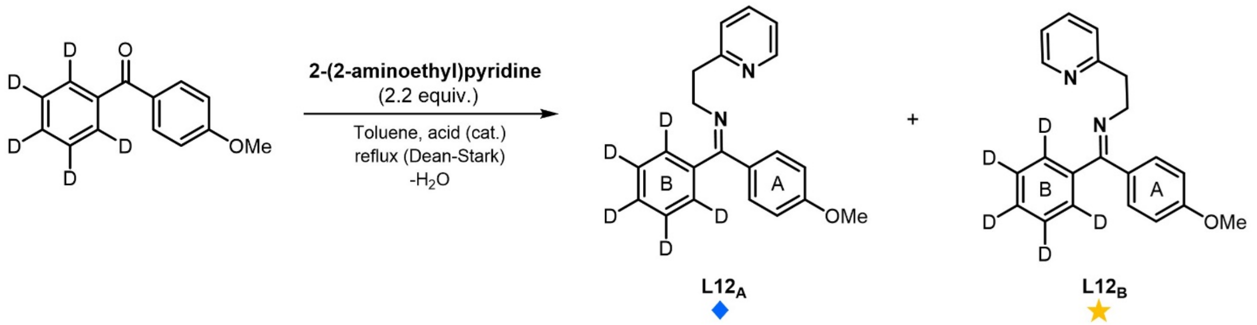


In an oven-dried flask, 2-(2-pyridyl)ethylamine (0.40 mL, 3.32 mmol, 2.2 equiv) was added to (4-methoxyphenyl)(phenyl-d5)methanone (synthesized using the synthetic protocol reported by G. Dong and coworkers)[7] (0.329 g, 1.51 mmol) and p-toluenesulfonic acid monohydrate (cat. 11 mg, 4 mol%) in toluene (30 mL). The reaction mixture was refluxed under argon with a Dean-Stark apparatus until imine formation was complete (24 h). The reaction was cooled to room temperature and diluted with diethyl ether (20 mL). The organic layer was washed with saturated ammonia chloride (30 mL x 2), saturated aqueous sodium bicarbonate (30 mL), brine (30 mL), and dried with magnesium sulfate. The final product isolated was an orange solid (95% yield, 86% pure).

$^1$H-NMR (500 MHz, $CDCl_3$): δ 8.50-8.49 (m, $L^A$+$L^B$: 2H), 7.58-7.54 (m, $L^A$+$L^B$: 4H), 7.23-7.16 (m, $L^A$+$L^B$: 2H), 7.11-7.08 (m, $L^A$+$L^B$: 2H), 6.98-6.92 (d, $L^B$: 4H), 6.86-6.84 (d, $L^A$: 2H), 3.88 (s, $L^B$: 3H), 3.82 (s, $L^A$: 3H). 3.87-3.84 (t, $L^B$: 2H), 3.79-3.75 (t, $L^A$: 2H), 3.23-3.19 (m, $L^A$+$L^B$: 4H).

HRMS (ESI) m/z $[M + Na]^+$ calculated for $C_{21}H_{15}D_5N_2O$ 321.1889, found 322.2003.

Note: The ratio of $L12_A$/ $L12_B$ (61/39) is calculated using the average of the integration of $CH_2$ peaks and CH peaks.

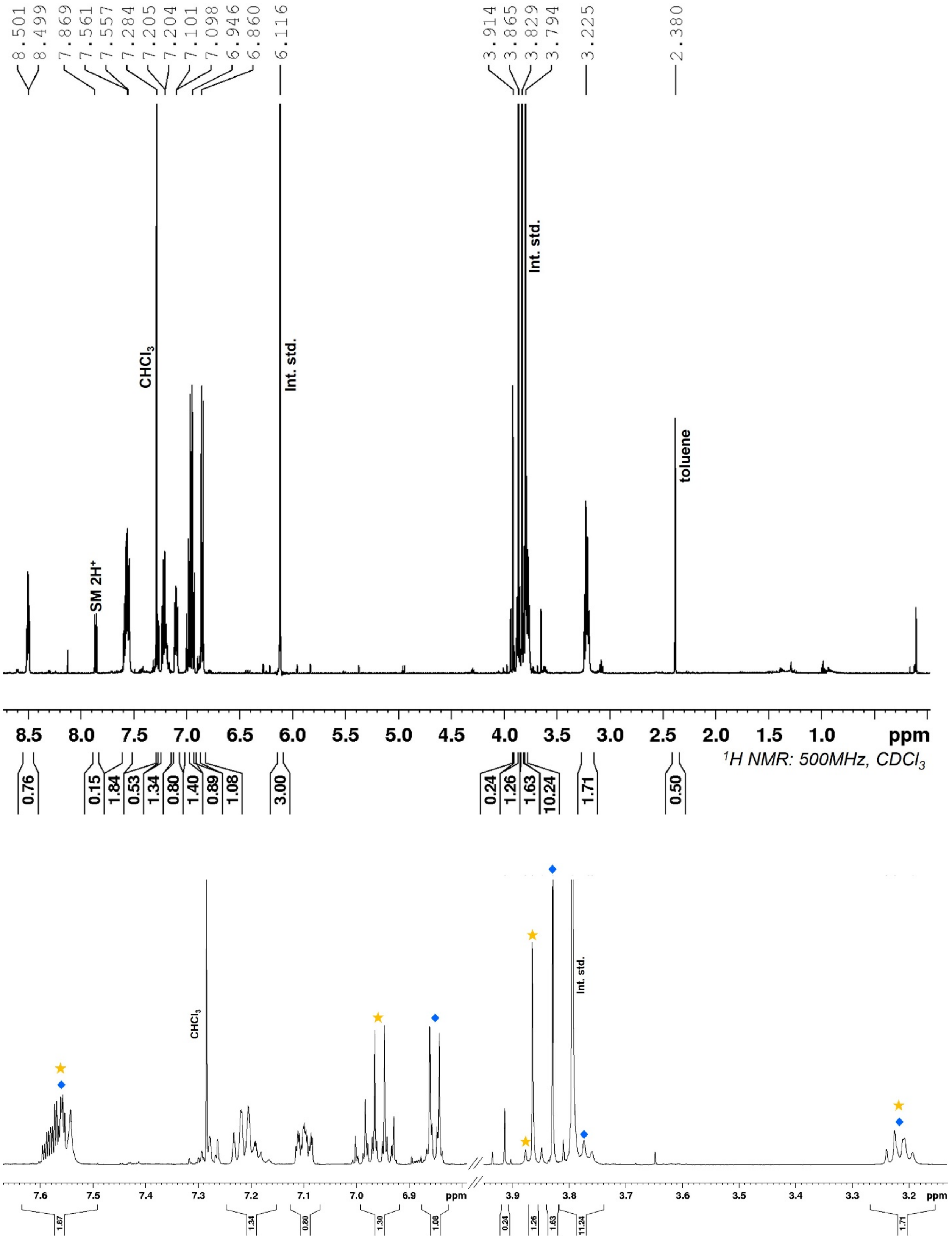


**Figure S36.** $^{1}$H-NMR spectra of L12.

### 5.1.1. Hydroxylation of L12

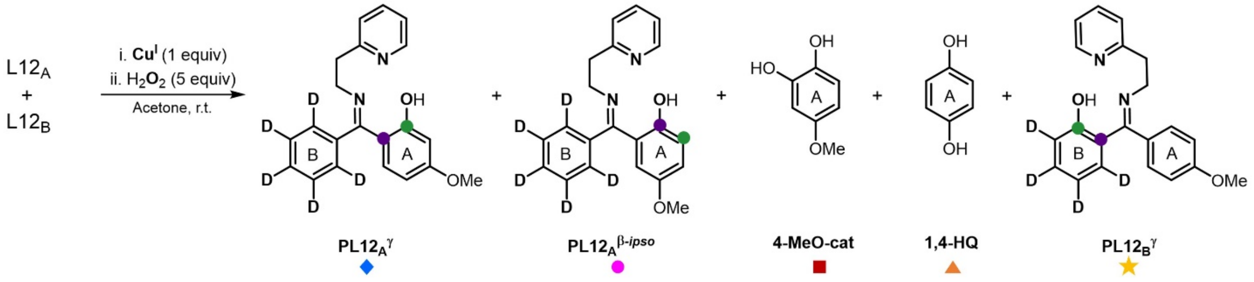


The reaction was carried out on a 0.159 mmol scale using 51.11 mg of imine according to the Standard Procedure. The crude product was quantified using 0.159 mmol of 1,3,5-trimethoxybenzene (int. std.) (yield of $PL12_A^{\gamma}$: 4%, $PL12_A^{\beta\text{-ipso}}$:16% catechol:7%, HQ:4% and $PL12_B^{\gamma}$ : 16%, 47% total yield). The identity of the hydroxylation products was confirmed by $^1$H-NMR.

### 5.1.2. Cleavage of DG of PL12

In a round bottom flask equipped with a stir bar, the mixture of $PL12_A^{\gamma}$, $PL12_A^{\beta\text{-ipso}}$, catechol, HQ and $PL12_B^{\gamma}$ were dissolved using 50 mL EtOAc. To this mixture 100 mL 1M HCl was added slowly and let it react for 30 min. The resulting mixture was extracted with EtOAc (50 mL X 2). The organic phases were separated, combined, dried over $MgSO_4$, filtered, and dried under vacuum. The organic products were identified using GC-MS analysis.

Note: GC-MS analysis of the reaction confirmed the sole formation of the unlabeled hydroquinone (i.e. no $d_4$-hydroquinone was detected, suggesting that the hydroquinone product is derived from the oxidation of the phenyl ring containing the MeO substituent).

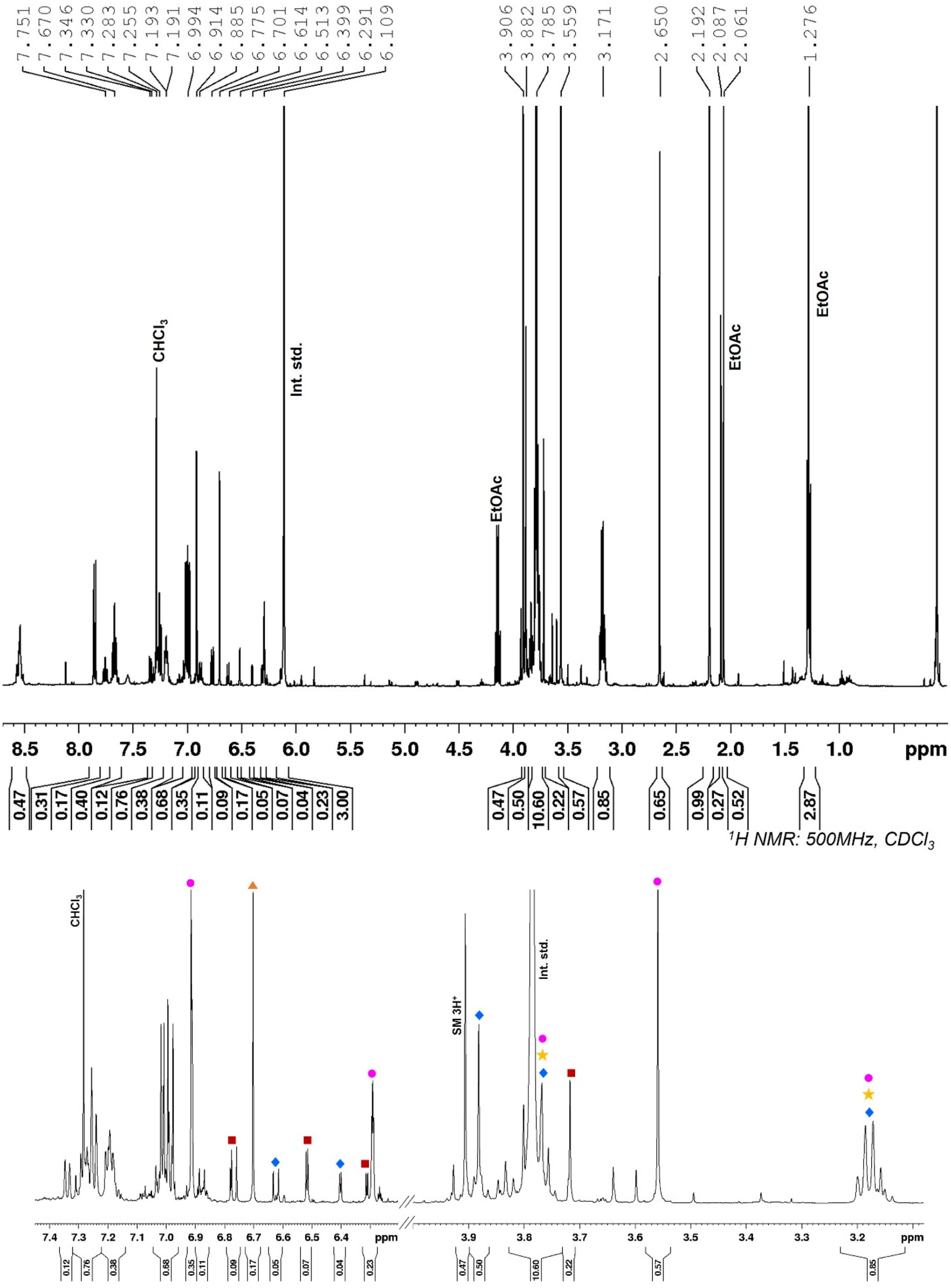


**Figure S37.** $^1$H-NMR spectra for the hydroxylation of L12.

**GC-MS Data for the cleavage of DG of PL12**

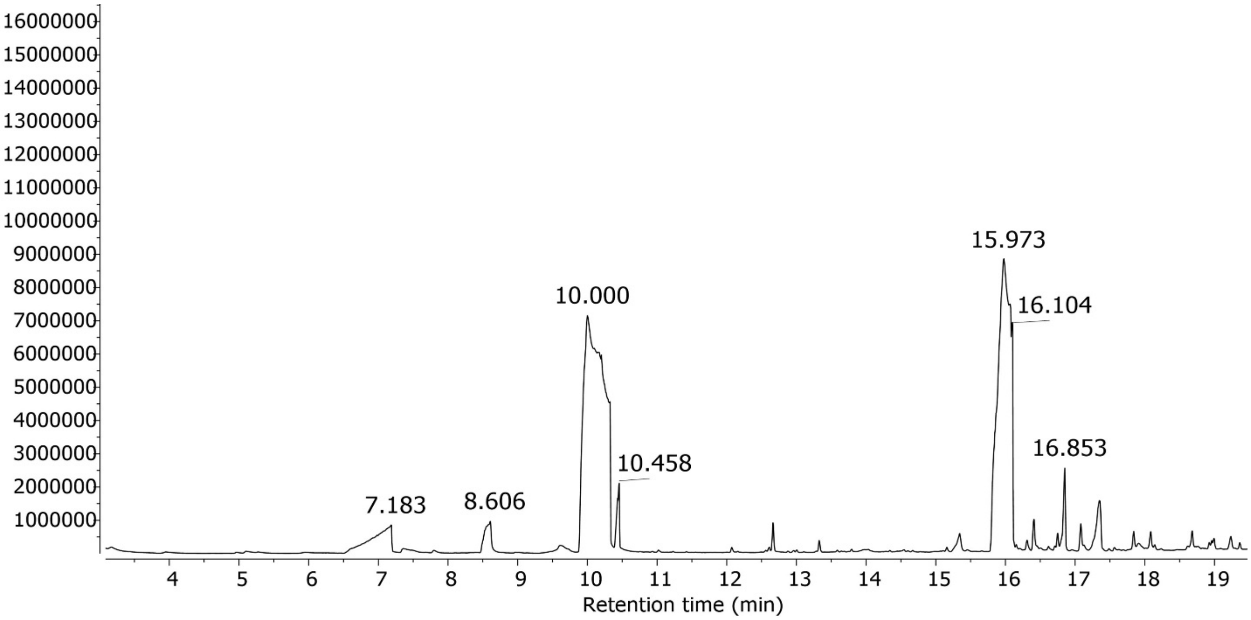


**Figure S38.** GC spectra of product mixture after cleavage of DG of PL12. Retention time (min) (m/z): 7.183 (127), 8.606 (110), 10.000 (168) (int.std.), 10.458 (140), 16.104 (233), 16.853 (233).

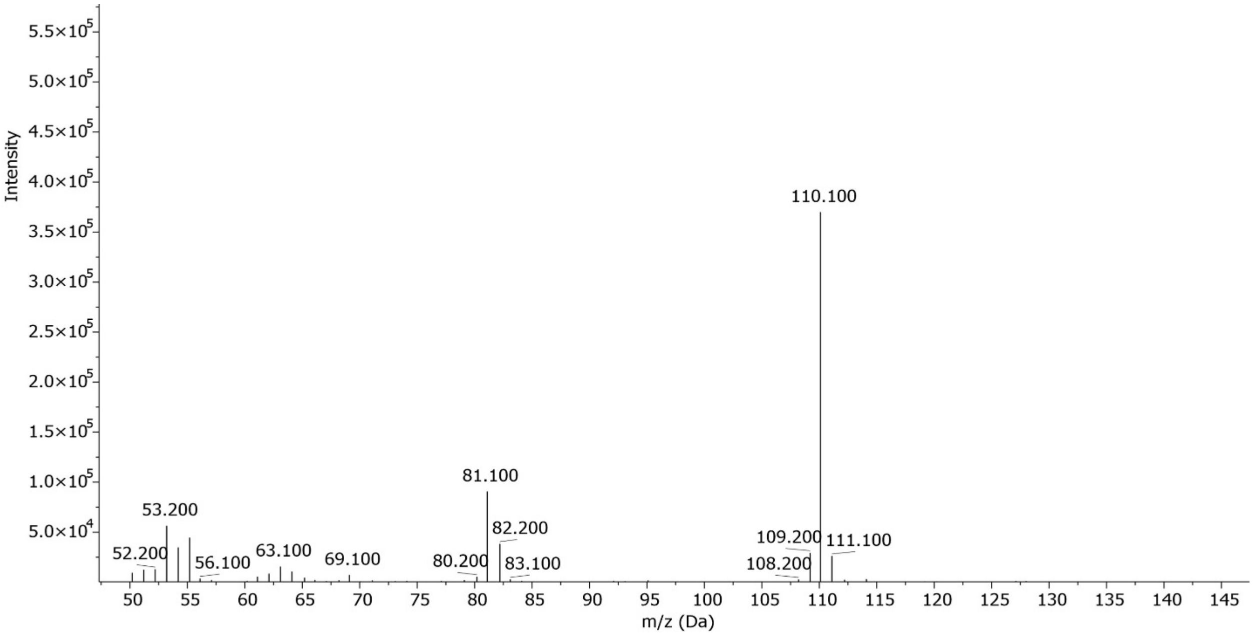


**Figure S39.** Mass spectrum of retention time (min) 8.606, Detected m/z = 110.10 (1,4-HQ**)**

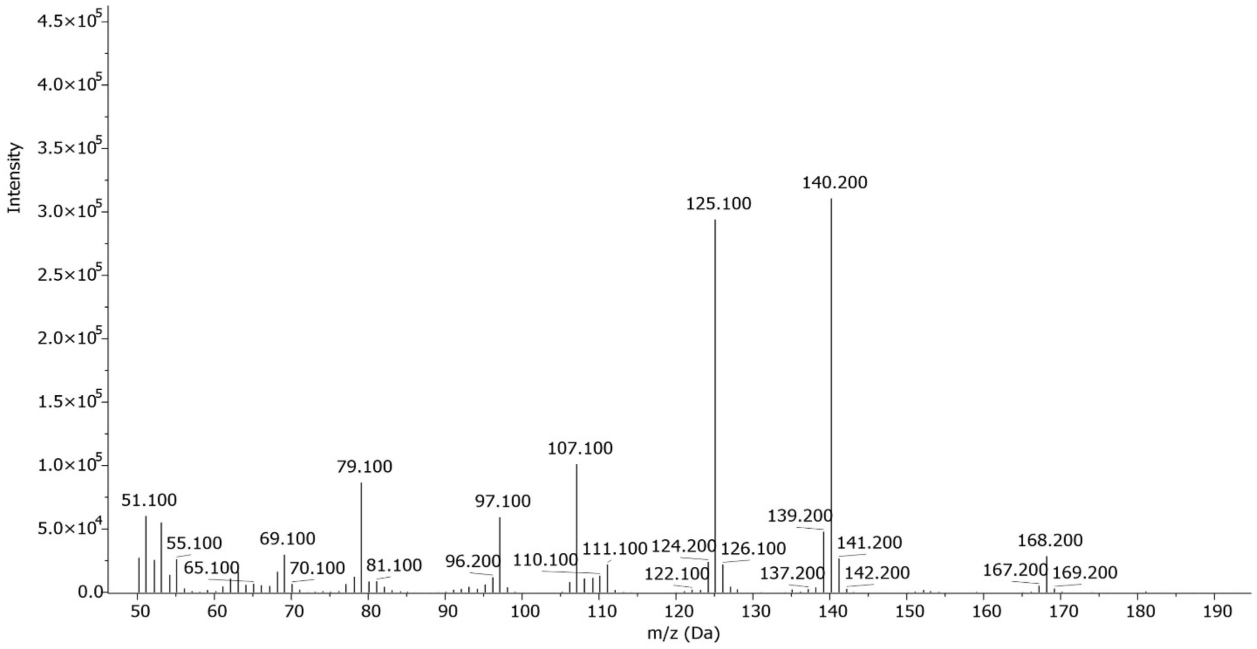


**Figure S40.** Mass spectrum of retention time (min) 10.458, Detected m/z = 140.20 (4-MeO catechol)

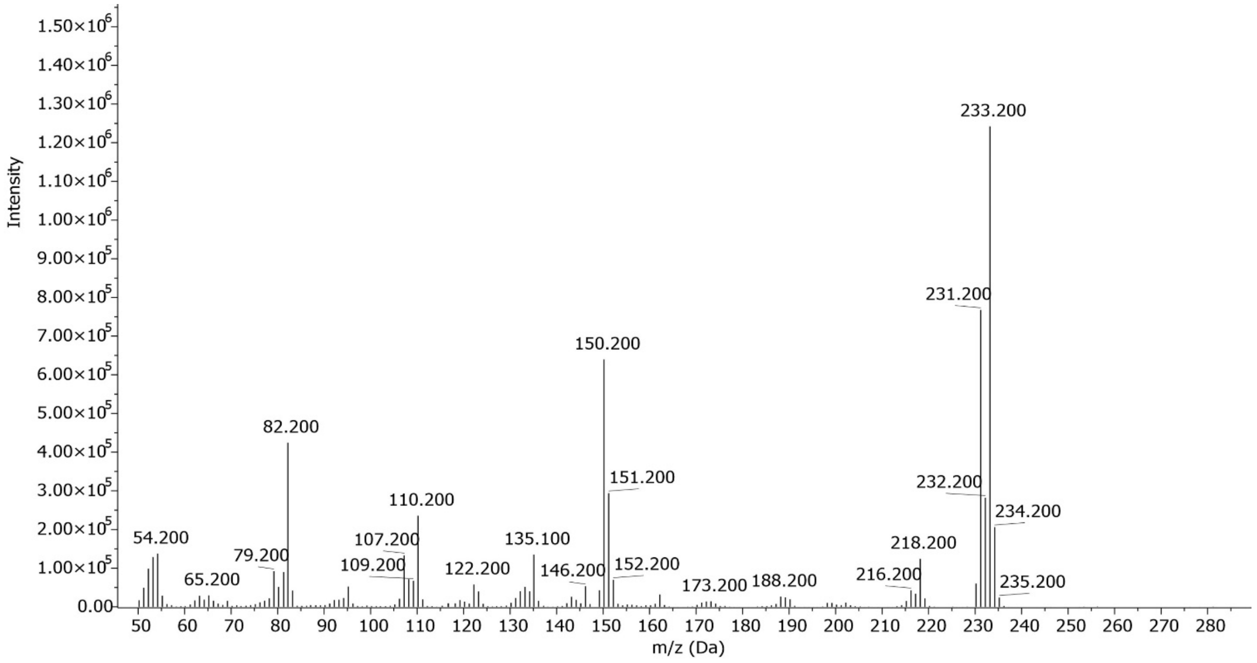


**Figure S41.** Mass spectrum of retention time (min) 16.104, Detected m/z = 233.20 ($P12_A^{\beta\text{-ipso}}$)

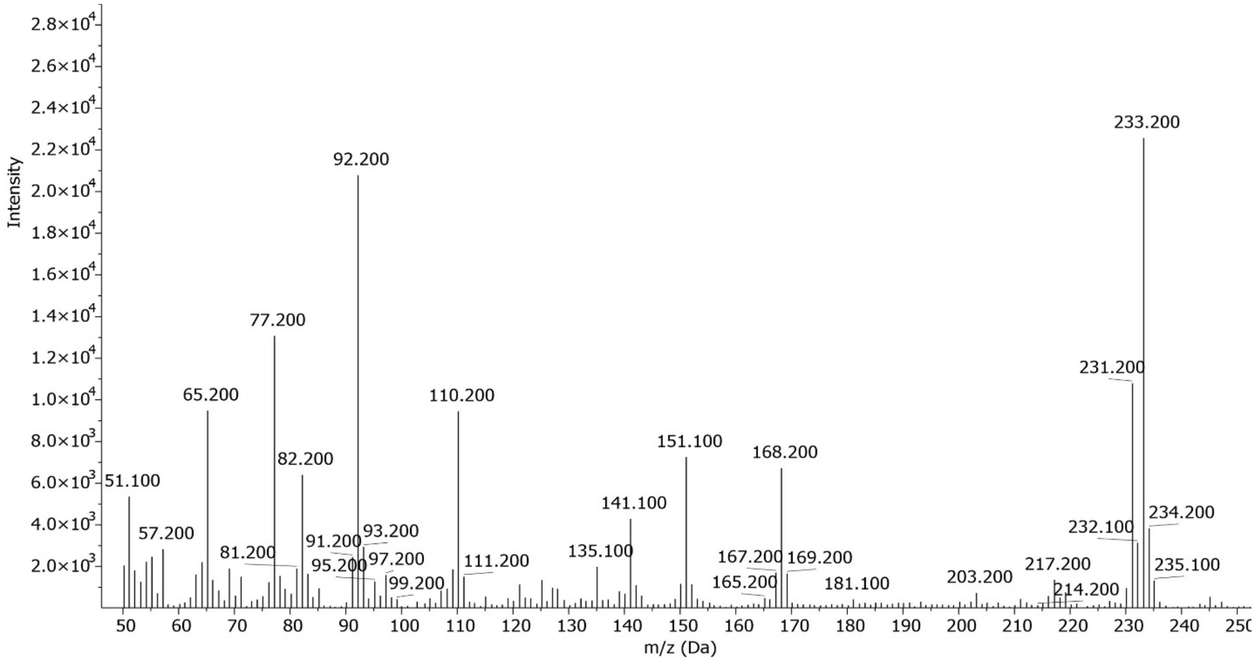


**Figure S42.** Mass spectrum of retention time (min) 16.853, Detected m/z = 233.20 ($P12_{A}^{\gamma}$)

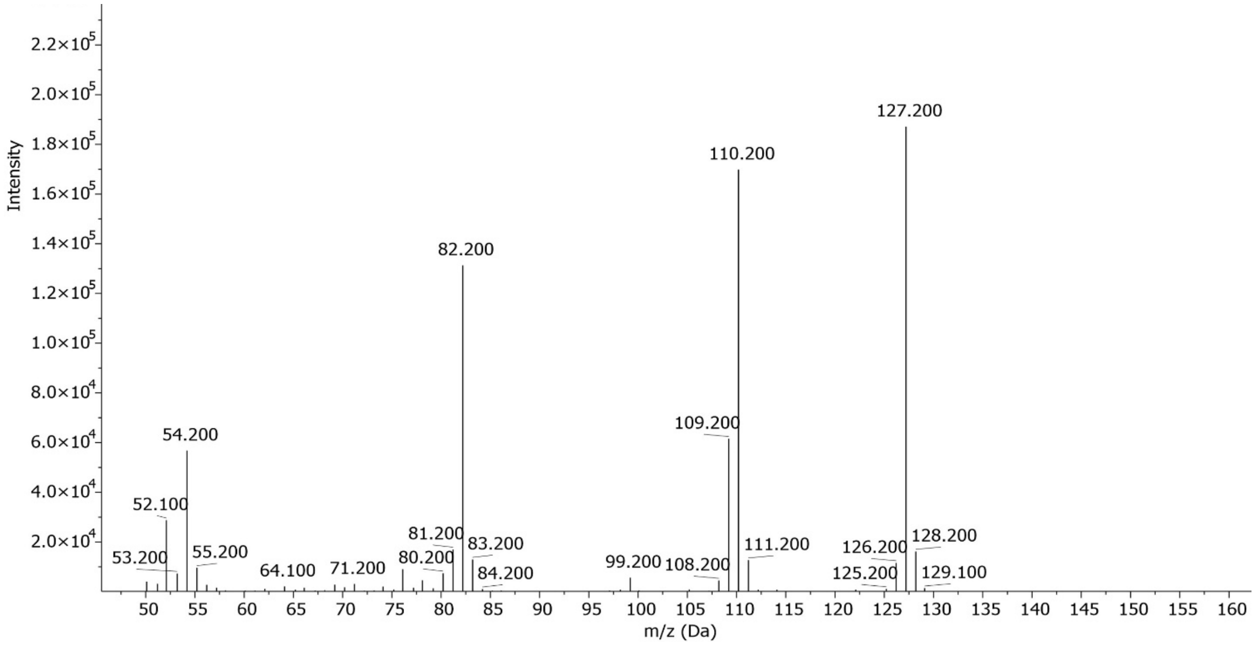


**Figure S43.** Mass spectrum of retention time (min) 7.183, Detected m/z = 127.20 (d5-benzoic acid)

## 6. Labelled $H_2O$ reactions

### 6.1. Hydroxylation of L8 with anhydrous $H_2O_2$ and $H_2{}^{16}O$

In the glovebox, 2 mL of acetone was added to an 8-mL vial containing 0.08 mmol of the imine substrate-ligand equipped with a stir bar. To the solution, 0.08 mmol of $[Cu^I(CH_3CN)_4](PF_6)$ was added and allowed to react. The solution mixture was taken out of the glove box and 2.5 equiv of anhydrous $H_2O_2$ was added along with 20equiv of DI $H_2{}^{16}O$. After 30 minutes, the reaction was quenched using $Na_2EDTA$ (25 mL, pH = 4), and EtOAc (25 mL x 3). The organic phases were separated, combined, dried over $MgSO_4$, filtered, and dried under vacuum.

#### 6.1.1. Cleavage of DG of hydroxylation products mixture

In a round bottom flask equipped with a stir bar, the hydroxylation products mixture was dissolved using 25 mL EtOAc. To this mixture 50 mL 1M HCl was added slowly and let it react for 30 min. The resulting mixture was extracted with EtOAc (25 mL X 2). The organic phases were separated, combined, dried over $MgSO_4$, filtered, and dried under vacuum. The organic products were identified using GC-MS analysis and yield was calculated using $^1$H-NMR ($P8_A{}^{\gamma}$: 1%, $P8_A{}^{\beta\text{-}ipso}$: 11%,, 4-MeO-cat: 15%,, and 1,4-dihydroquinone: 2%, Yield:29 %, MB: 59%) ($P8_B{}^{\gamma}$ was not detected).

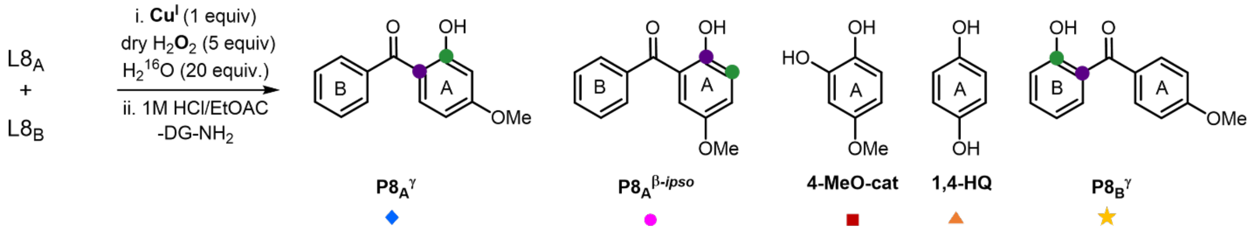

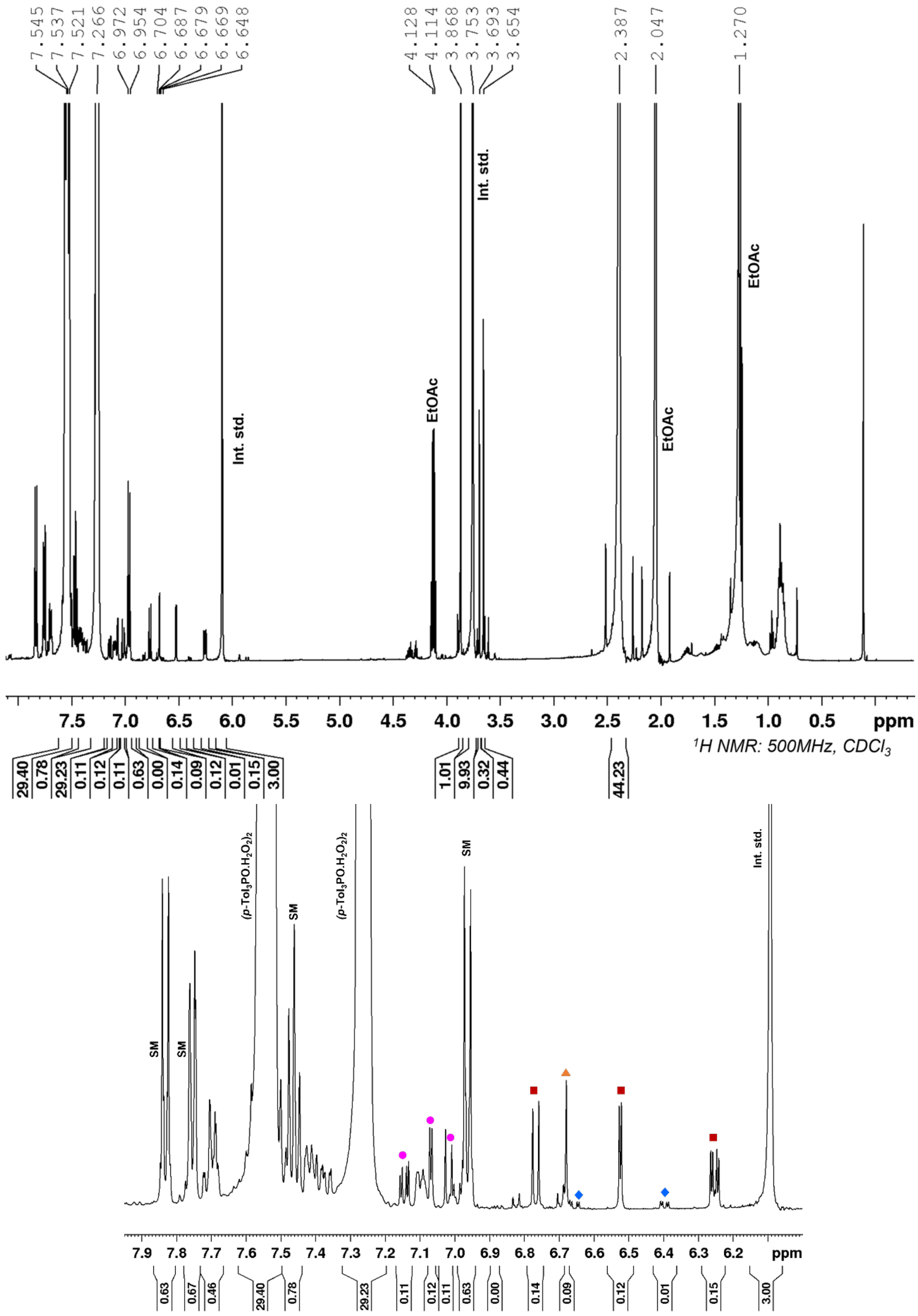


**Figure S44.** $^1$H-NMR spectra for the cleavage of DG of PL8 (for the reaction with anhydrous $H_2O_2$ and $H_2{}^{16}O$)

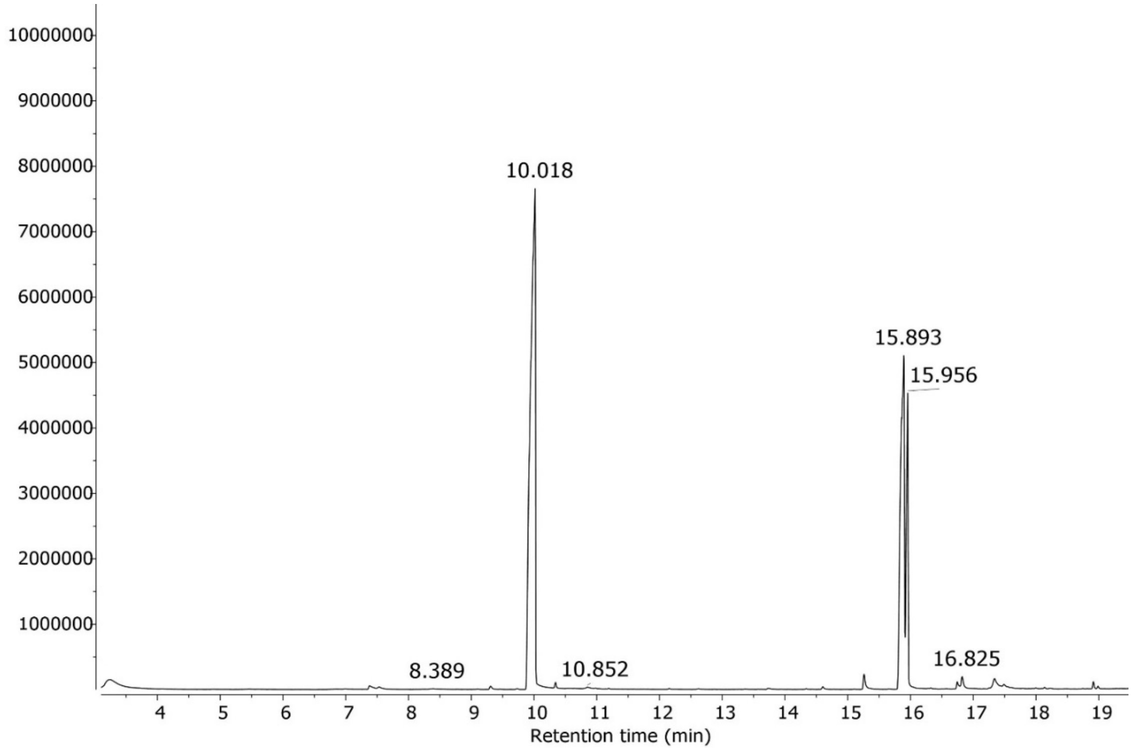


**Figure S45.** GC spectra of product mixture after hydroxylation of L8 with anhydrous $H_2O_2$ and $H_2{}^{16}O$**.** Retention time (min) (m/z): 8.389 (110), 10.018 (168) (int.std.), 10.852 (140), 15.956 (228), 16.825 (228).

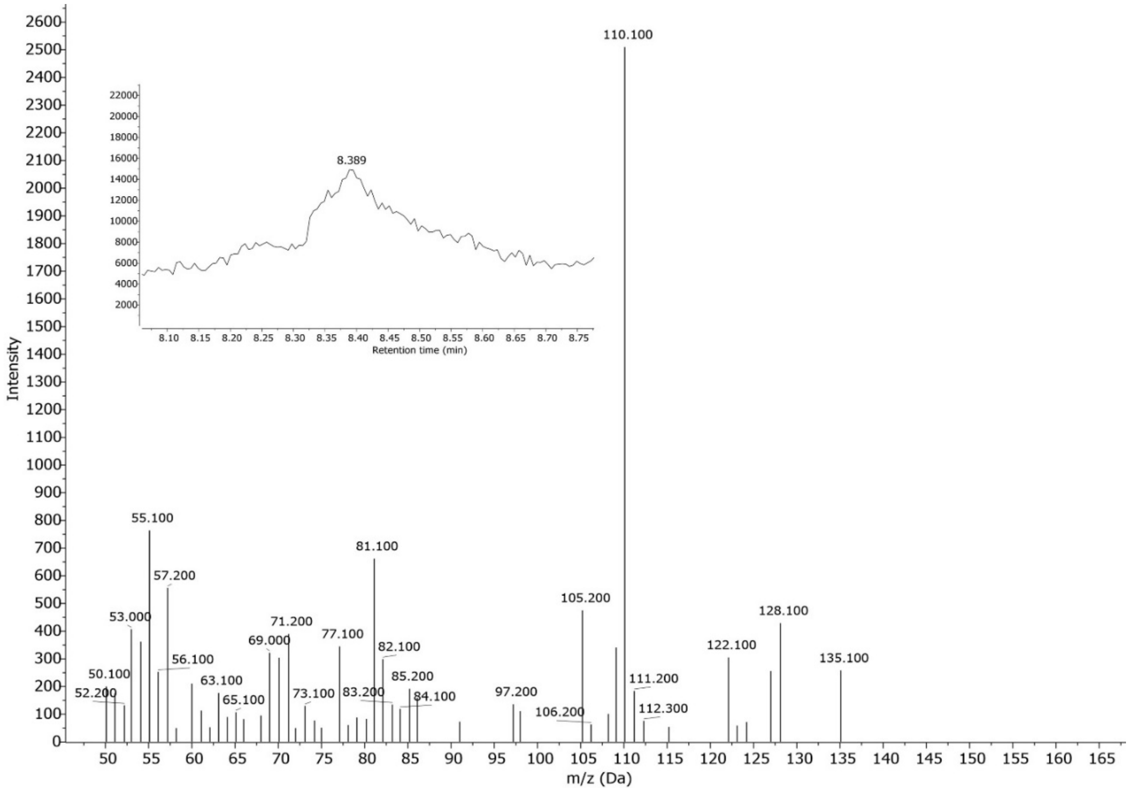


**Figure S46.** Mass spectrum of retention time (min) 8.389, Detected m/z = 110.10 (1,4-HQ)

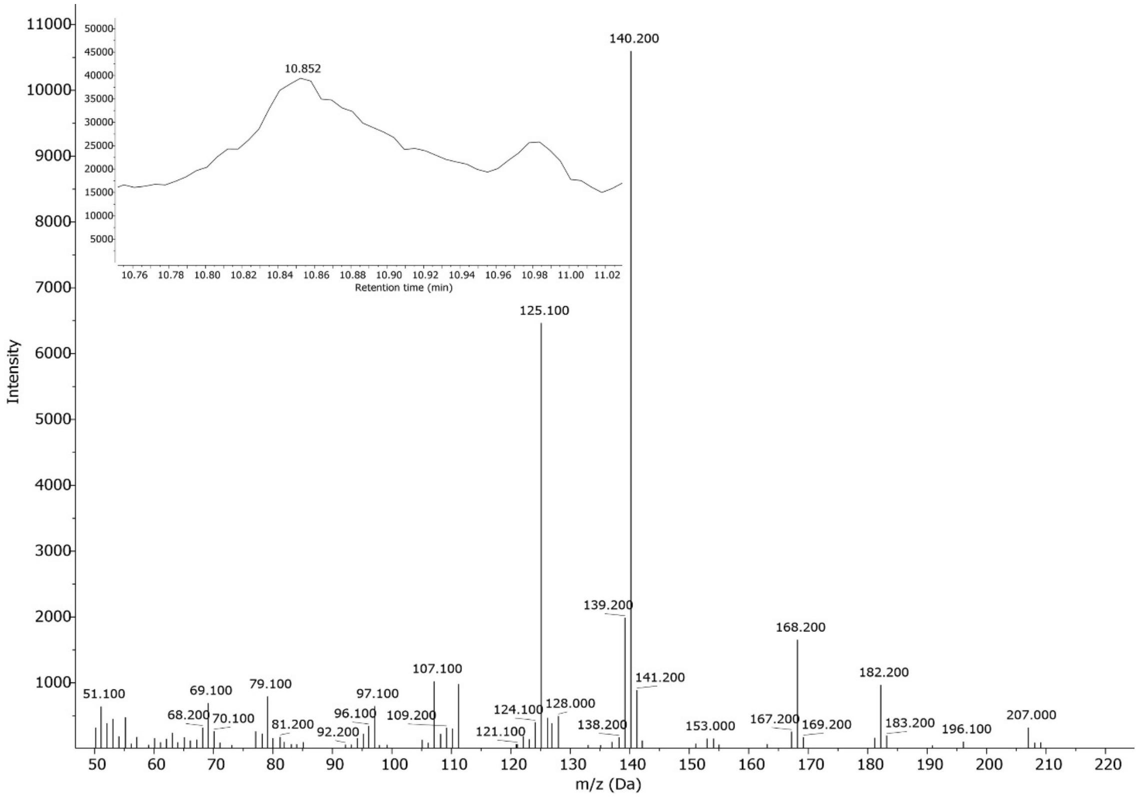


**Figure S47.** Mass spectrum of retention time (min) 10.852, Detected m/z = 140.20 (4-MeO-cat)

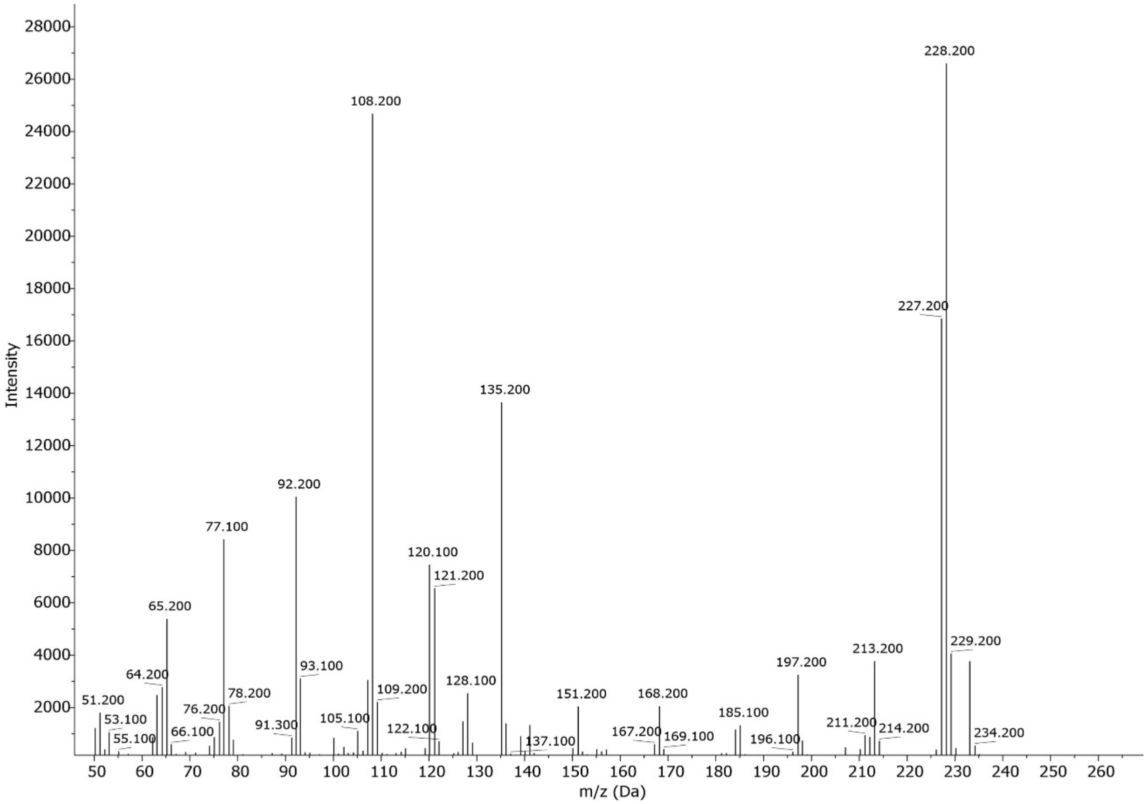


**Figure S48.** Mass spectrum of retention time (min) 15.956, Detected m/z = 228.2 ($P8_A^{\beta\text{-}ipso}$)

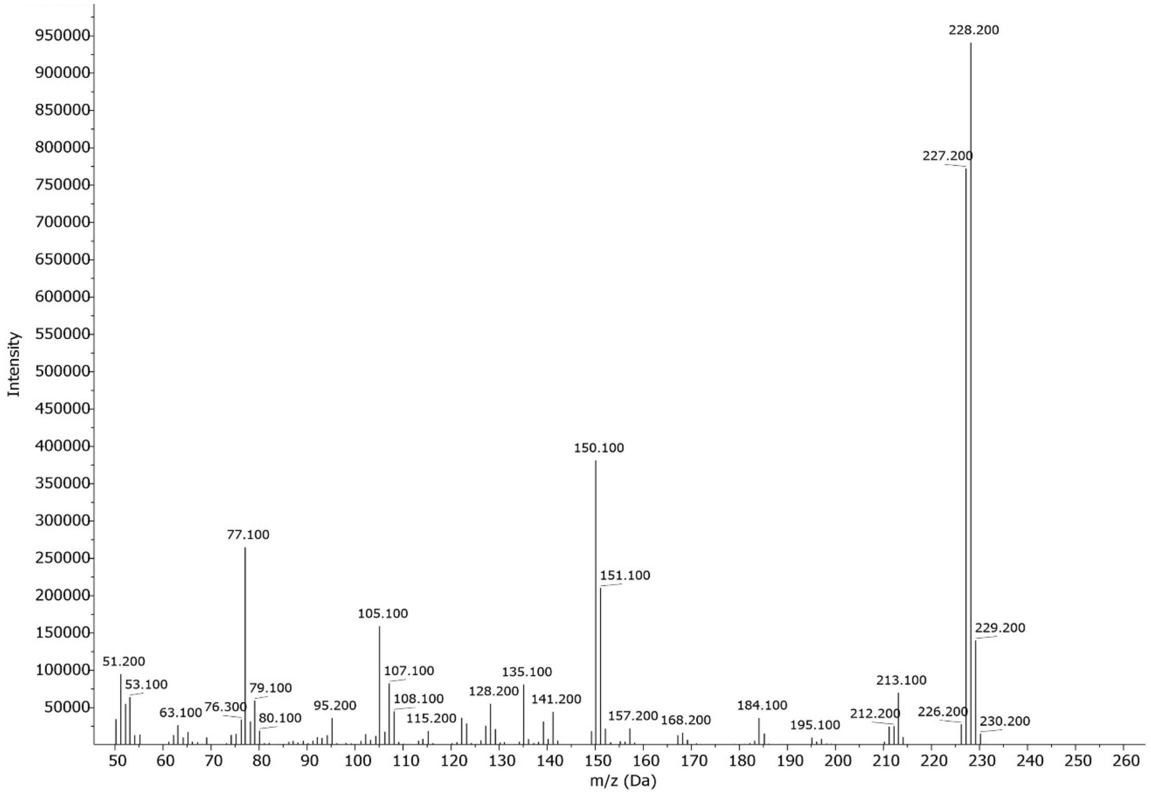


**Figure S49.** Mass spectrum of retention time (min) 16.825, Detected m/z = 228.2 ($P8_A{}^{\gamma}$)

### 6.2. Hydroxylation of L8 with anhydrous $H_2O_2$ and $H_2{}^{18}O$

In the glovebox, 2 mL of acetone was added to an 8-mL vial containing 0.08 mmol of the imine substrate-ligand equipped with a stir bar. To the solution, 0.08 mmol of $[Cu^I(CH_3CN)_4](PF_6)$ was added and allowed to react. The solution mixture was taken out of the glove box and 2.5 equiv of anhydrous $H_2O_2$ was added along with 20equiv of DI $H_2{}^{18}O$. After 30 minutes, the reaction was quenched using $Na_2EDTA$ (25 mL, pH = 4), and EtOAc (25 mL x 3). The organic phases were separated, combined, dried over $MgSO_4$, filtered, and dried under vacuum.

#### 6.2.1. Cleavage of DG of hydroxylation products mixture

In a round bottom flask equipped with a stir bar, the hydroxylation products mixture was dissolved using 25 mL EtOAc. To this mixture 50 mL 1M HCl was added slowly and let it react for 30 min. The resulting mixture was extracted with EtOAc (25 mL X 2). The organic phases were separated, combined, dried over $MgSO_4$, filtered, and dried under vacuum. The organic products were identified using GC-MS analysis and yield was calculated using $^1$H-NMR ($P8_A{}^{\gamma}$: 1%, $P8_A{}^{\beta\text{-}ipso}$: 11%,, 4-MeO-cat: 3%,, and 1,4-dihydroquinone: 3%, MB: 29%) ($P8_B{}^{\gamma}$ was not detected).

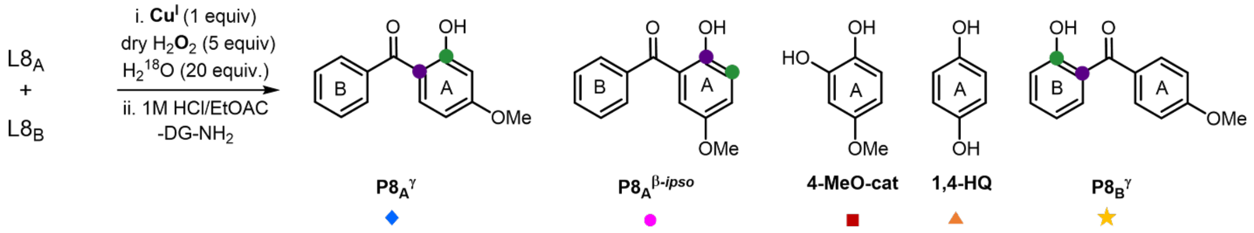

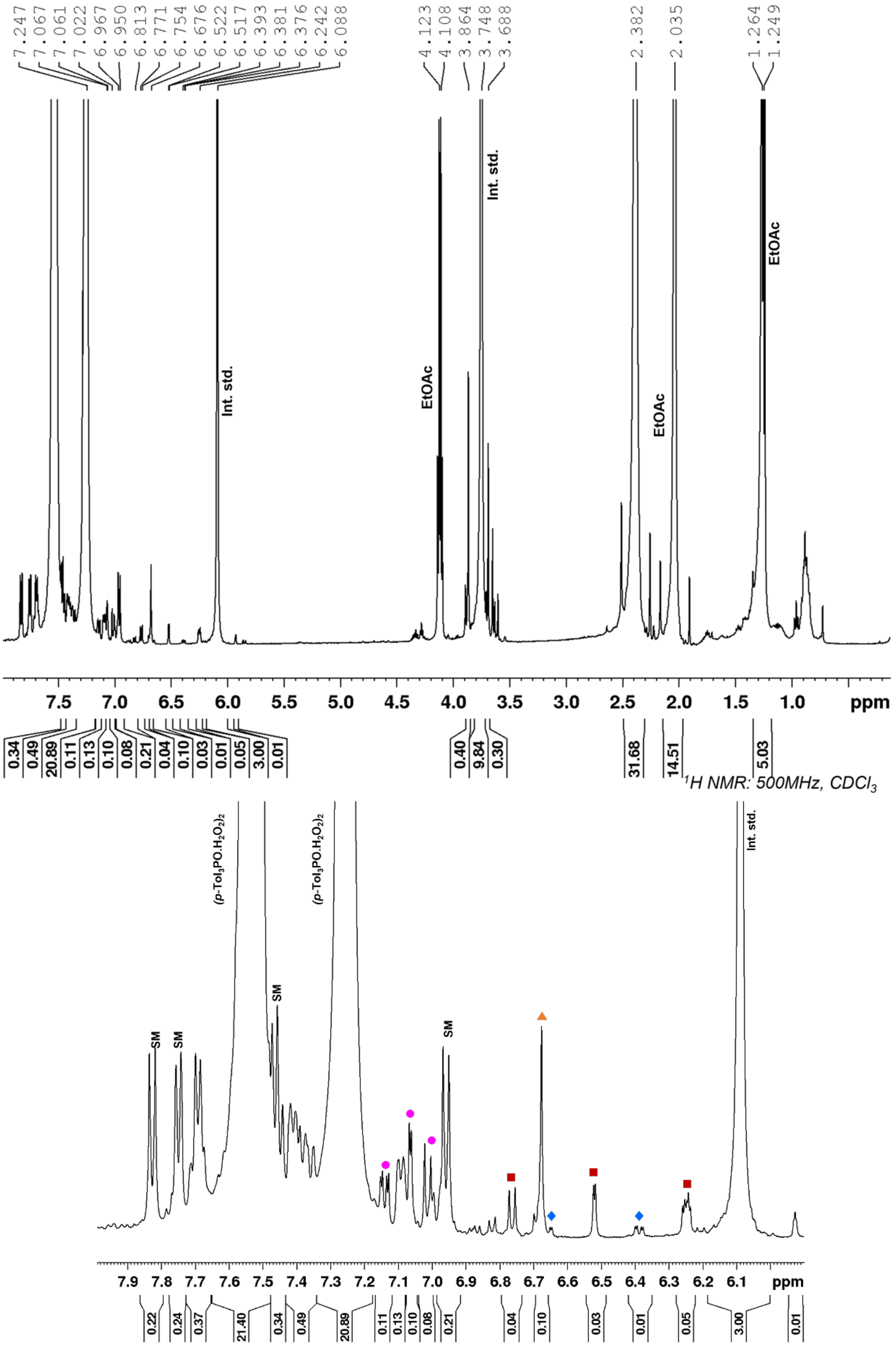


**Figure S50.** $^1$H-NMR spectra for the cleavage of DG of PL8 (for the reaction with anhydrous $H_2O_2$ and $H_2{}^{16}O$)

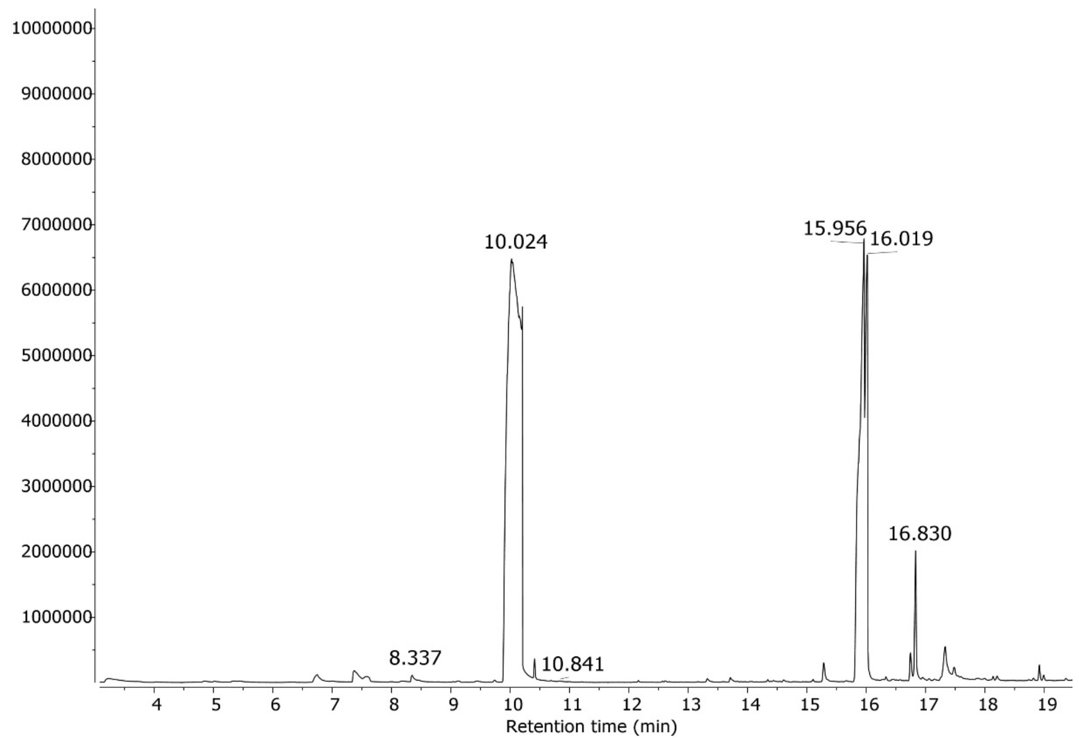


**Figure S51.** GC spectra of product mixture after hydroxylation of L8 with anhydrous $H_2O_2$ and $H_2{}^{18}O$**.** Retention time (min) (m/z): 8.337 (110/112), 10.024 (168) (int.std.), 10.841 (140), 16.019 (228), 16.830 (228).

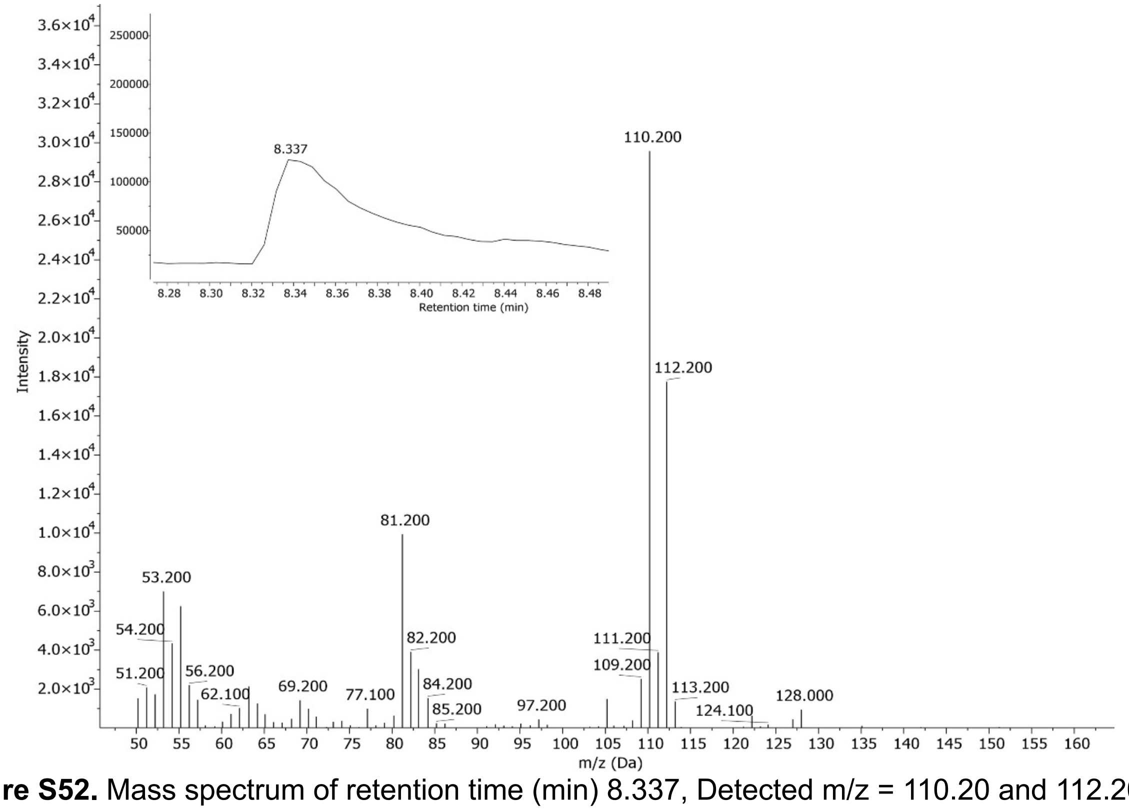


**Figure S52.** Mass spectrum of retention time (min) 8.337, Detected m/z = 110.20 and 112.20 indicated the formation of $^{16}O,^{18}O$-hydroquinone.

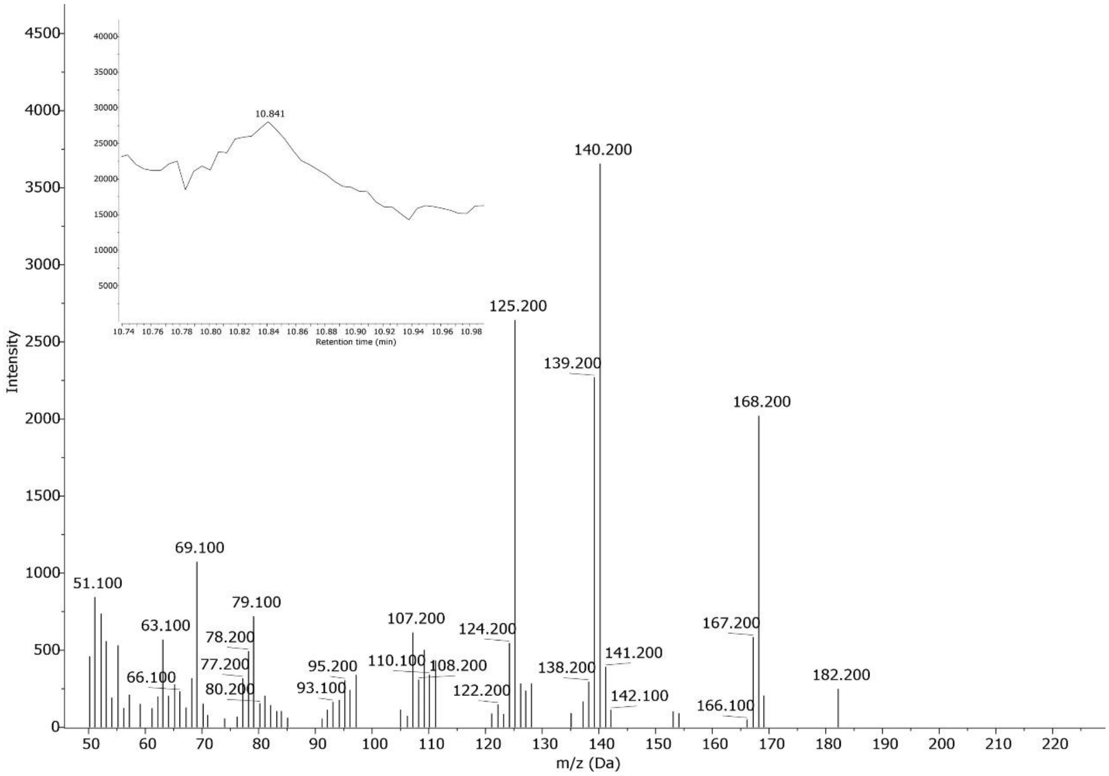


**Figure S53.** Mass spectrum of retention time (min) 10.841, Detected m/z = 140.20 (4-MeO-cat)

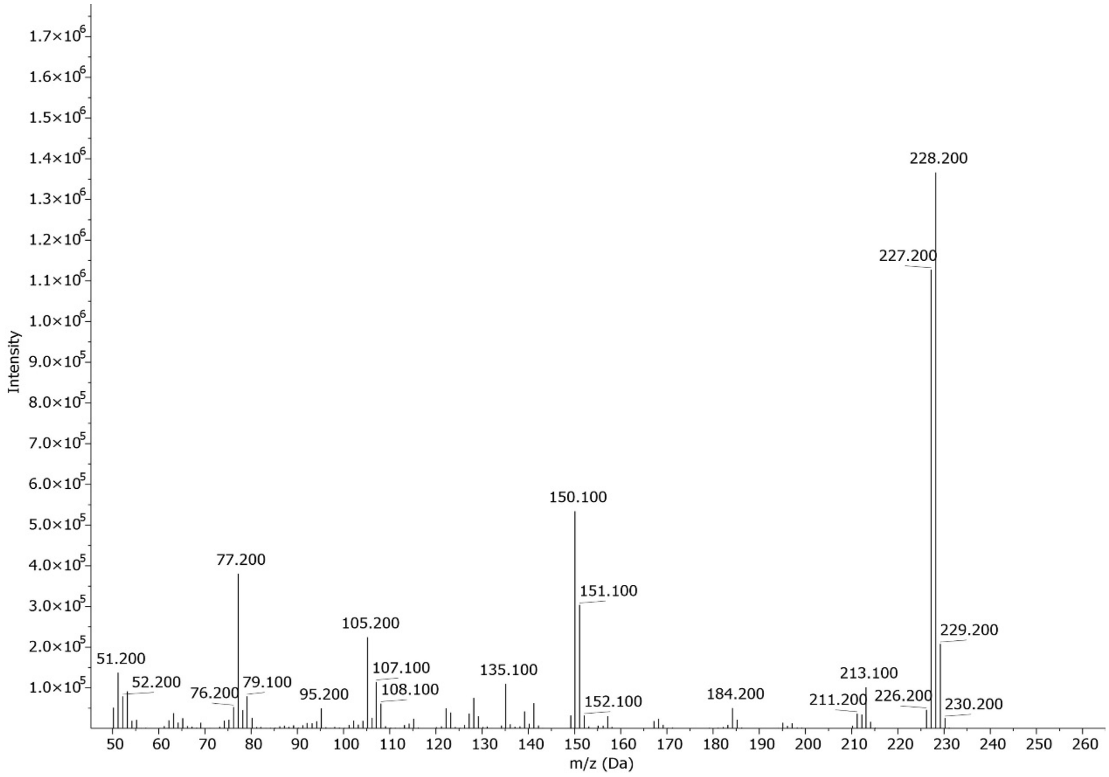


**Figure S54.** Mass spectrum of retention time (min) 16.019, Detected m/z = 228.2 ($P8_A^{\beta\text{-ipso}}$)

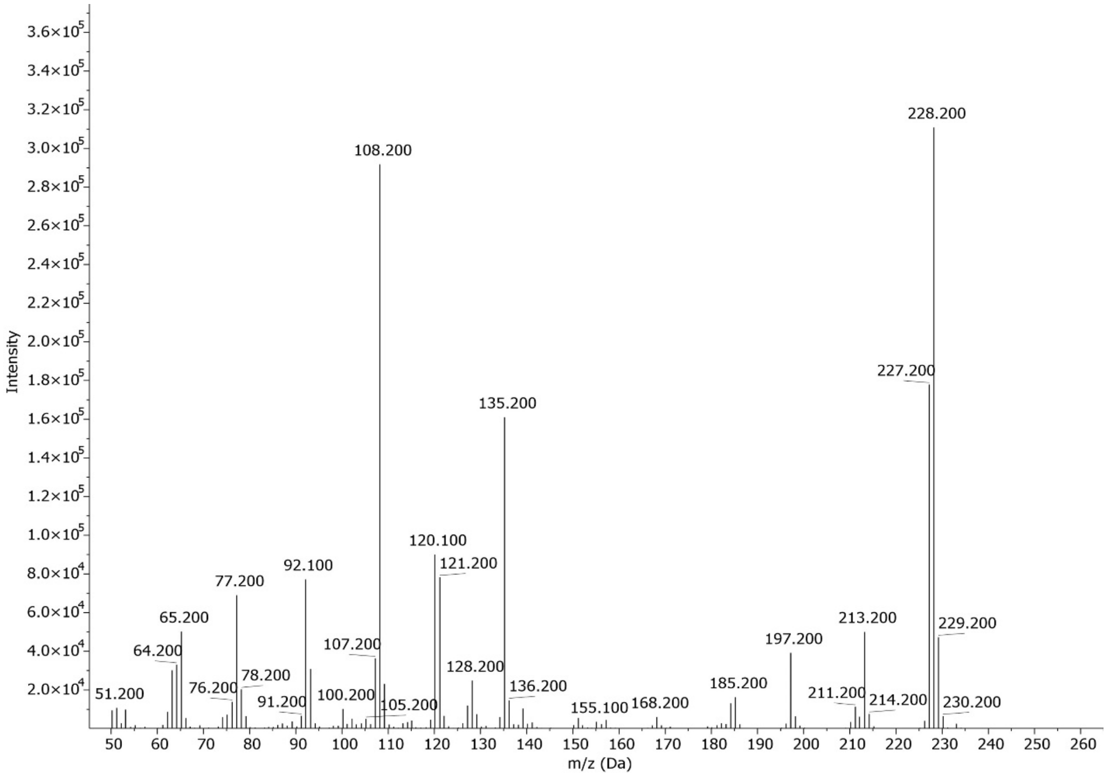


**Figure S55.** Mass spectrum of retention time (min) 16.830, Detected m/z = 228.2 ($P8_A^{\gamma}$)

## 7. Synthesis and oxidation of the product substrate-ligands

### 7.1. Synthesis of 2-hydroxy-4-methoxy substrate ligand

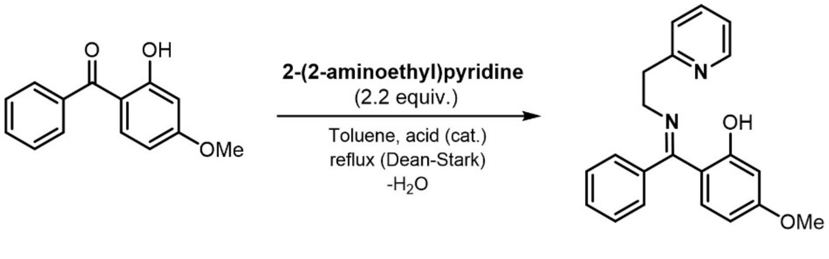


$PL8_A{}^{\gamma}$

In an oven-dried flask, 2-(2-pyridyl)ethylamine (0.6 mL, 4.82 mmol, 2.2 equiv) was added to 2-hydroxy-4-methoxybenzophenone (0.500 g, 2.19 mmol) and p-toluenesulfonic acid monohydrate (cat. 15 mg, 3.7 mol%) in toluene (40 mL). The reaction mixture was refluxed under argon with a Dean-Stark apparatus until imine formation was complete (24 h). The reaction was cooled to room temperature and diluted with diethyl ether (30 mL). The organic layer was washed with saturated ammonia chloride (50 mL x 2), saturated aqueous sodium bicarbonate (50 mL), brine (50 mL), and dried with magnesium sulfate. The final product isolated was a yellow solid (86% yield, 86% pure).

$^1$H-NMR (500 MHz, $CDCl_3$): δ 8.54 (d, 1H), 7.63-7.60 td, 1H), 7.47 (m, 2H), 7.29-7.26 (m, 1H), 7.21-7.18 (m, 2H), 7.16-7.13(m, 1H), 7.06 (m, 1H), 6.60 (d, 1H), 6.41 (d, 1H), 6.13-6.11 (dd, 1H), 3.79 (s, 3H), 3.74-3.71 (t, 2H), 3.14-3.11 (t, 2H).

HRMS (ESI) m/z [M + Na]$^+$ calculated for $C_{21}H_{20}N_2O_2$ 332.1525, found 333.1616.

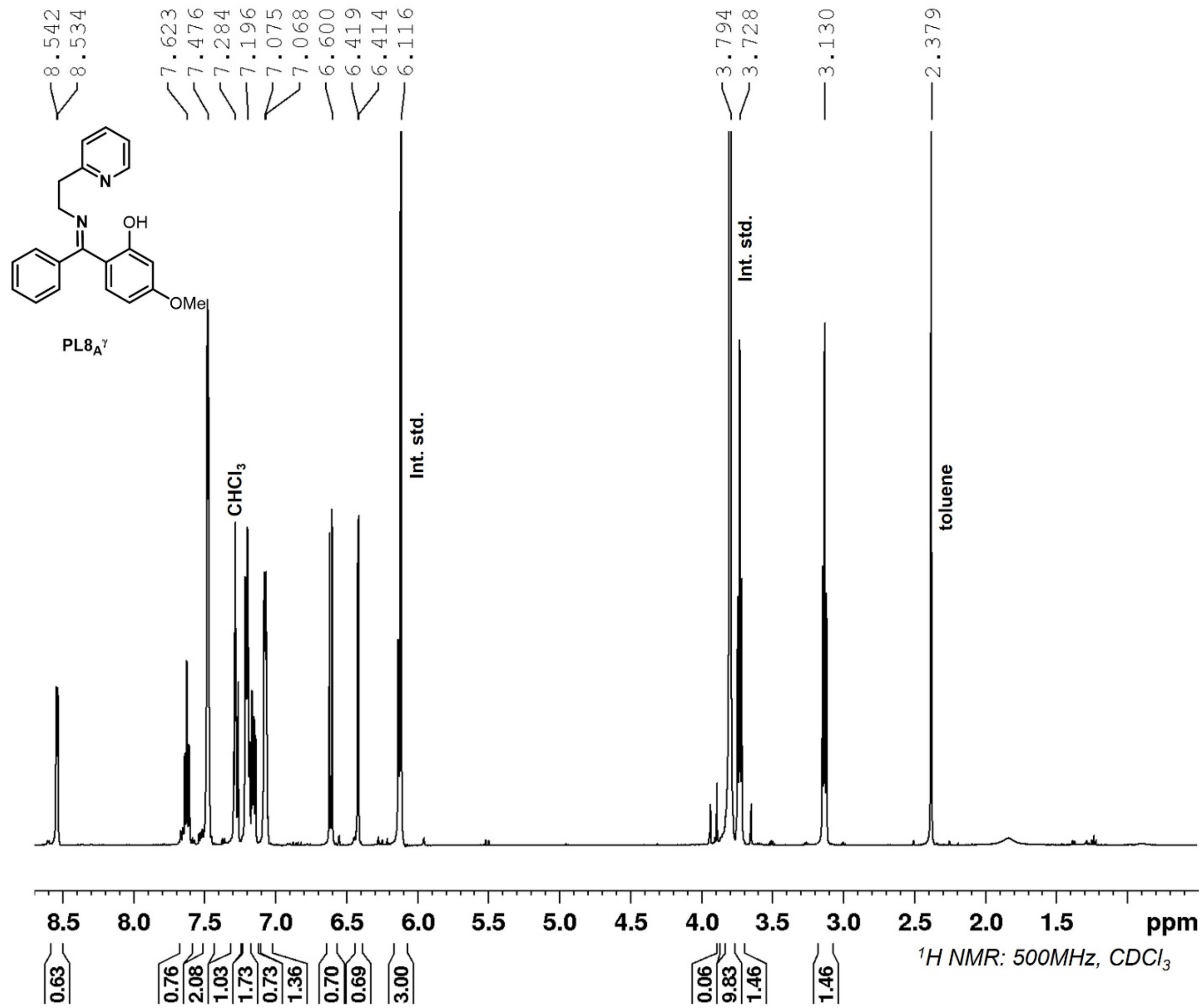


**Figure S56.** $^{1}$H-NMR spectra of PL8$_{A}^{\gamma}$.

### 7.1.1. Oxidation of $PL8_A^{\gamma}$

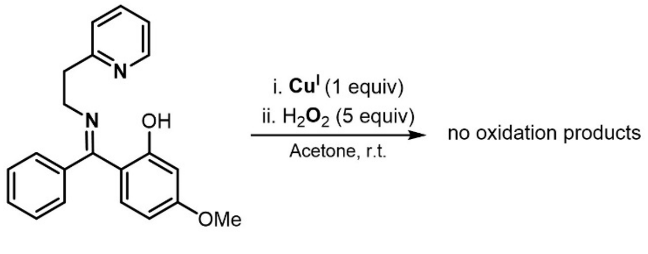


**$PL8_A^{\gamma}$**

The reaction was carried out on a 0.08 mmol scale using 28.00 mg of imine according to the Standard Procedure. The crude product was quantified using 0.08 mmol of 1,3,5-trimethoxybenzene (int. std.). The identity of the products was confirmed by $^1$H-NMR. 81% unreacted imine ligand was recovered.

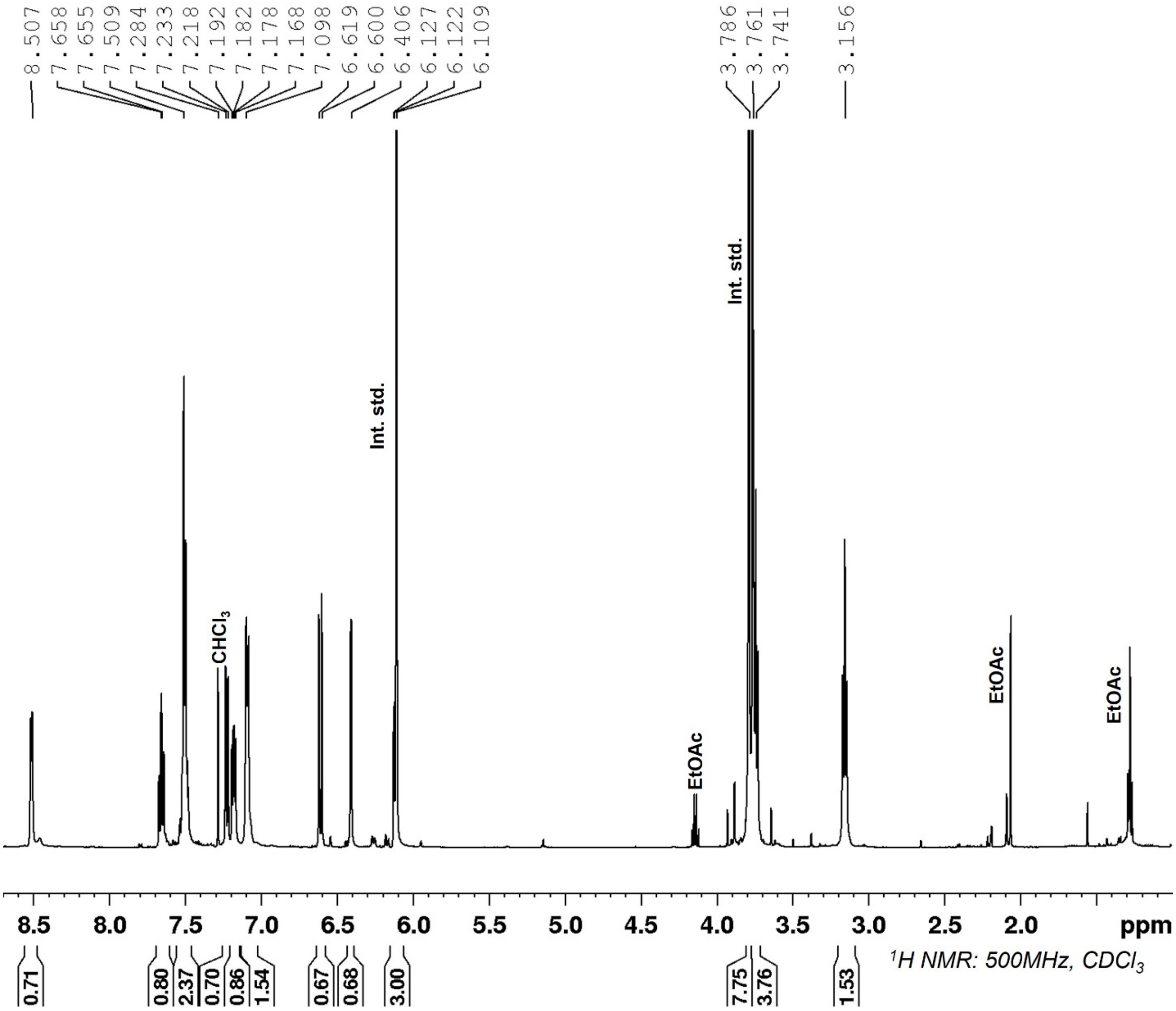


**Figure S57.** $^1$H-NMR spectra for the oxidation of $PL8_A^{\gamma}$.

## 7.2. Synthesis of 2-hydroxy-5-methoxy substrate ligand

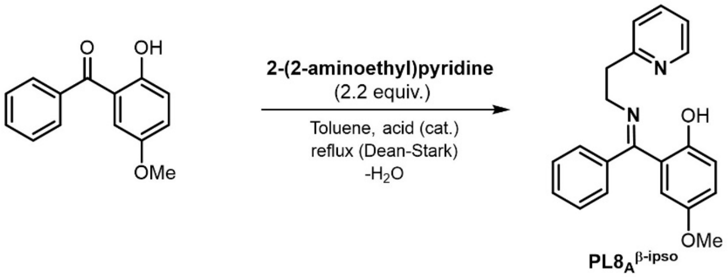

PL8$_A$$^{\beta\text{-ipso}}$

In an oven-dried flask, 2-(2-pyridyl)ethylamine (0.53 mL, 4.20 mmol, 2.2 equiv) was added to 2-hydroxy-4-methoxybenzophenone (0.450 g, 1.91 mmol) and p-toluenesulfonic acid monohydrate (cat. 15 mg, 4 mol%) in toluene (30 mL). The reaction mixture was refluxed under argon with a Dean-Stark apparatus until imine formation was complete (24 h). The reaction was cooled to room temperature and diluted with diethyl ether (20 mL). The organic layer was washed with saturated ammonia chloride (30 mL x 2), saturated aqueous sodium bicarbonate (30 mL), brine (30 mL), and dried with magnesium sulfate. The final product isolated was an orange solid (80% yield, 96% pure).

$^1$H-NMR (500 MHz, $CDCl_3$): δ 8.53 (d, 1H), 7.63-7.60 (td, 1H), 7.46 (m, 3H), 7.20-7.19 (d, 1H), 7.16-7.13(m, 1H), 7.06-7.04 (m, 2H), 6.93-6.90 (m, 2H), 6.30 (dd, 1H), 3.77-3.75 (t, 2H), 3.57 (s, 3H), 3.16-3.13 (t, 2H).

HRMS (ESI) m/z $[M + Na]^+$ calculated for $C_{21}H_{20}N_2O_2$ 332.1525, found 333.1624.

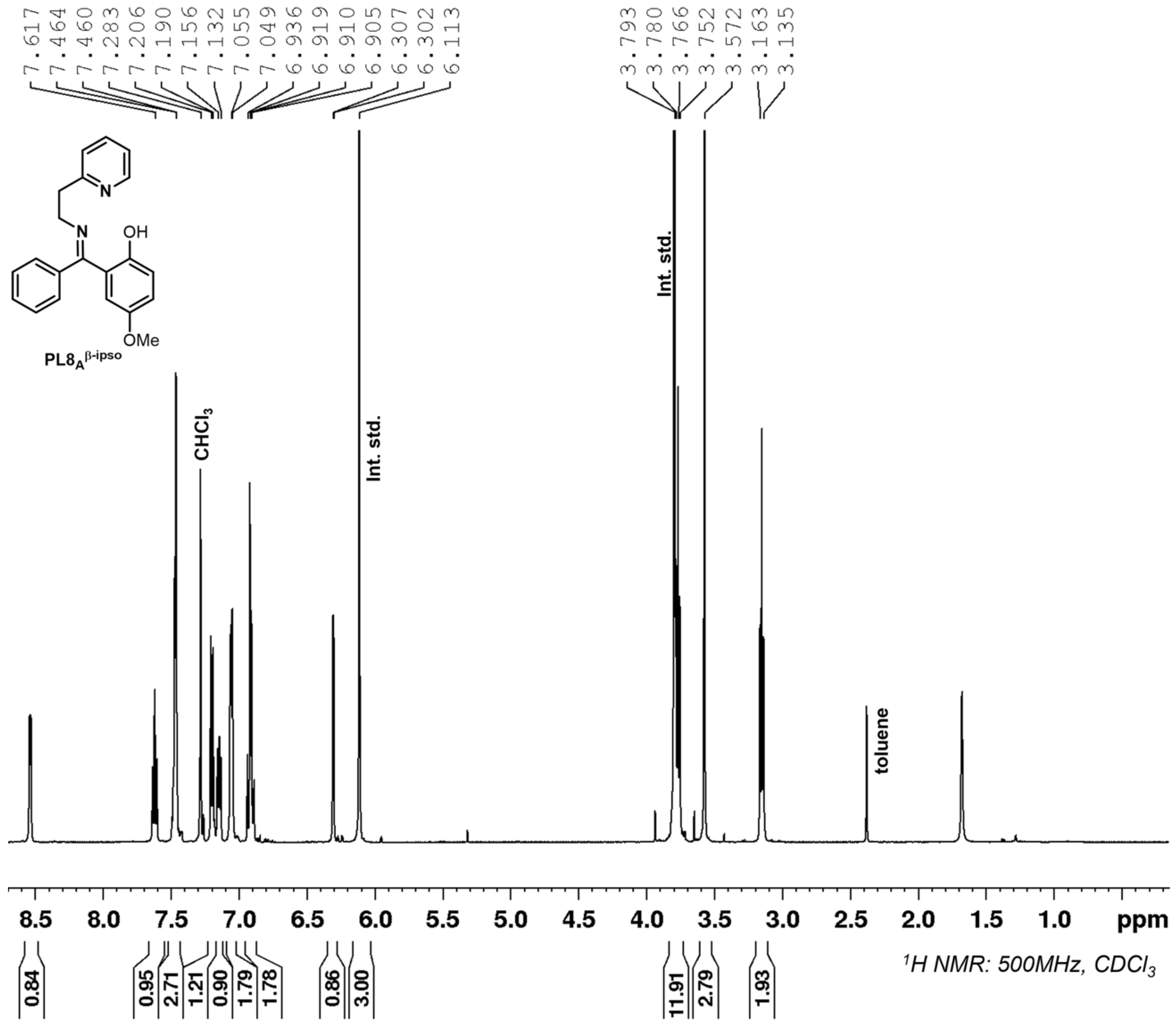


**Figure S58.** $^{1}$H-NMR spectra of $PL8_{A\text{-ipso}}$.

### 7.2.1. Oxidation of PL8$_{A\text{-ipso}}$

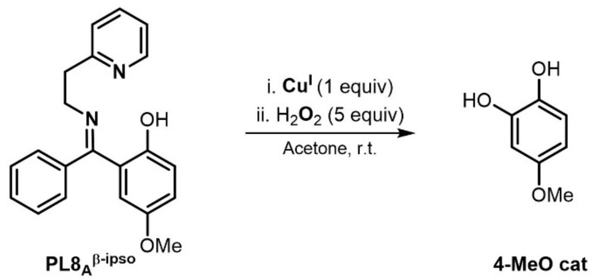


The reaction was carried out on a 0.08 mmol scale using 28.00 mg of imine according to the Standard Procedure. The brown crude product was quantified using 0.08 mmol of 1,3,5-trimethoxybenzene (int. std.). The identity of the product was confirmed by $^{1}$H-NMR.

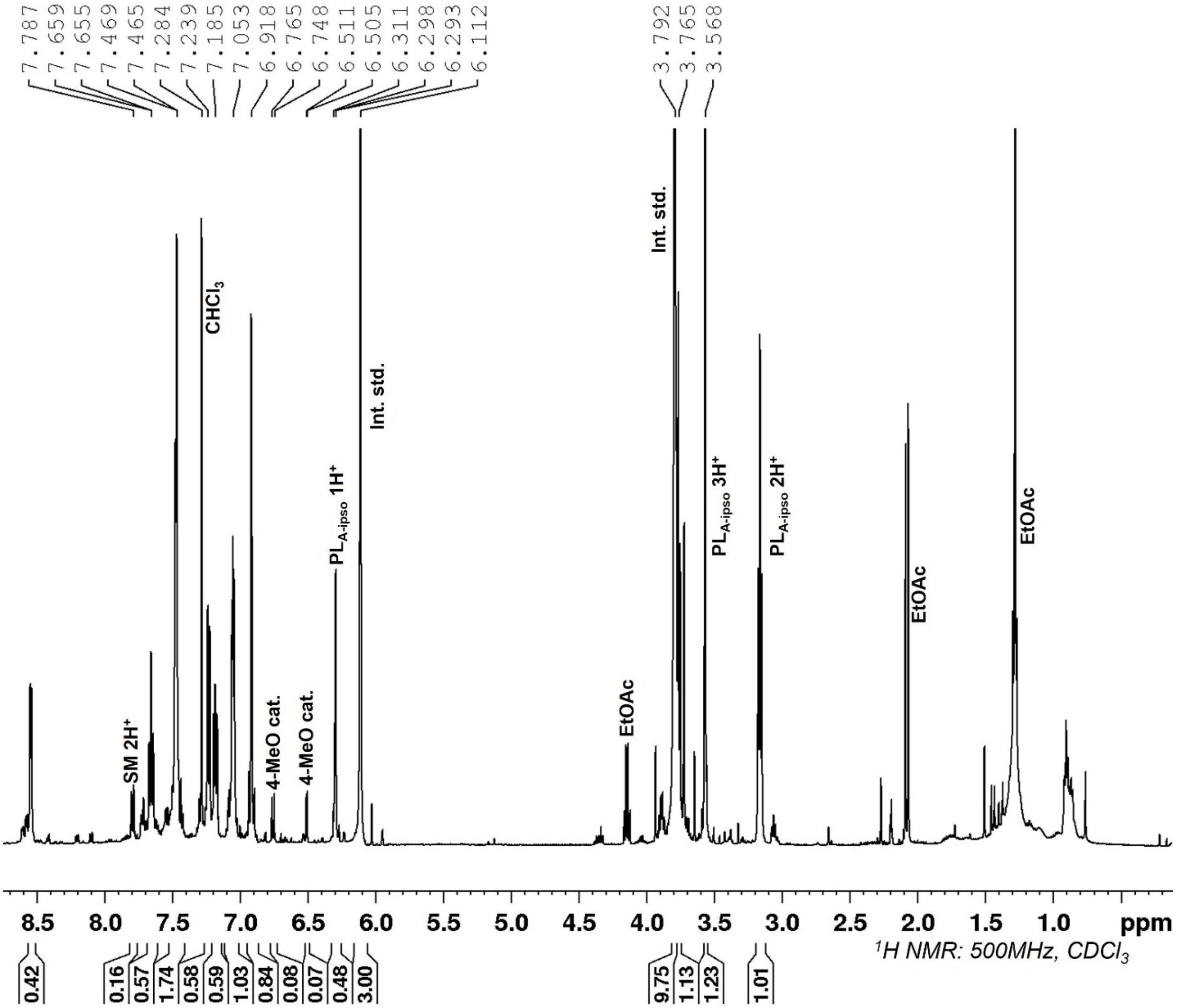


**Figure S59.** $^{1}$H-NMR spectra for the oxidation of PL8$_{A\text{-ipso}}$.

### 7.3 Oxidation of 2-hydroxy-5-methoxy substrate

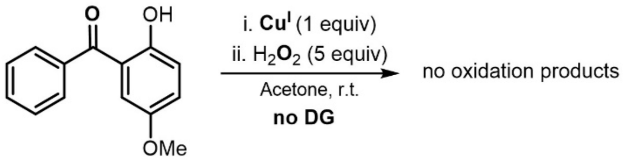


The reaction was carried out on a 0.08 mmol scale using 28.00 mg of imine according to the Standard Procedure. The brown crude product was quantified using 0.08 mmol of 1,3,5-trimethoxybenzene (int. std.). The identity of the product was confirmed by $^{1}$H-NMR.

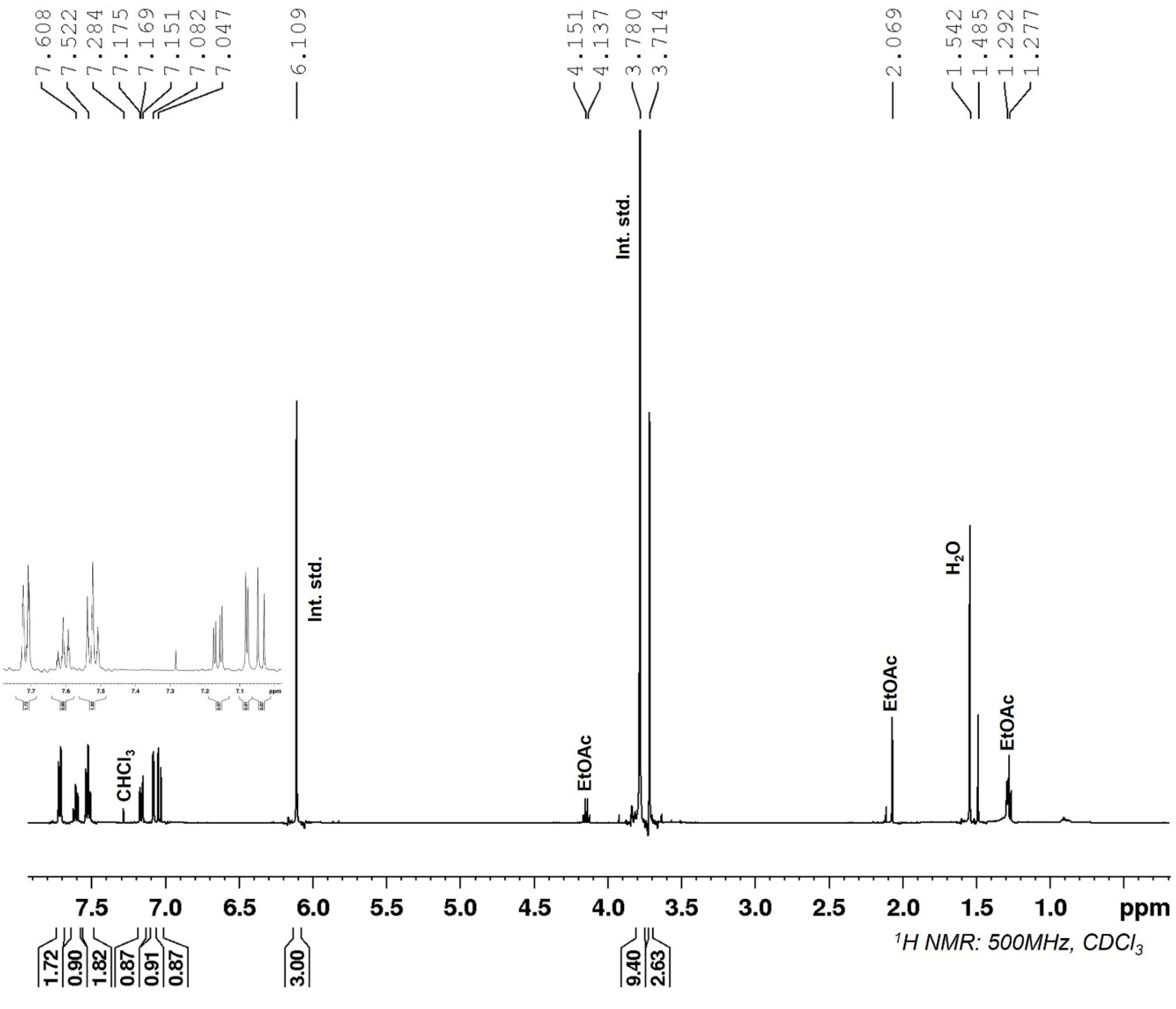


**Figure S60.** $^{1}$H-NMR spectra for the oxidation of 2-hydroxy-5-methoxy substrate.

## 8. Summary and mass balance of Cu-directed hydroxylations

**Table S1:** Summary and mass balance of Cu-directed hydroxylation of symmetrical imine substrate ligands(L1-L6)

| Ligand-substrate | SMX (%) | LX (%) | $PLX^{\gamma}$ (%) | $PLX^{\beta\text{-ipso}}$ (%) | 4-X-cat (%) | 1,4-HQ (%) | Yield (%) | Mass Balance (%) | $\gamma^{Oxid}/\beta^{Oxid}$ |
|---|---|---|---|---|---|---|---|---|---|
| L1 | 0 | 0 | 5 | 20 | 17 | 3 | 45 | 60 | 11/89 |
| L2 | 34 | 0 | 41 | 0 | 0 | 0 | 41 | 75 | 100/0 |
| L3 | 23 | 0 | 49 | 0 | 0 | 0 | 49 | 72 | 100/0 |
| L4 | 17 | 0 | 32 | 33 | 0 | 0 | 65 | 82 | 48/52 |
| L5 | 56 | 0 | 18 | 14 | 0 | 0 | 32 | 88 | 56/44 |
| L6 | 41 | 6 | 25 | 0 | 0 | 0 | 25 | 72 | 100/0 |

**Table S2:** Summary and mass balance of Cu-directed hydroxylation of unsymmetrical imine substrate ligands (L7-L11)

| Ligand-substrate | $LX_A/LX_B$ | SMX (%) | LX (%) | $PLX_A^{\gamma}$ (%) | $PLX_A^{\beta\text{-ipso}}$ (%) | 4-MeO-cat (%) | 1,4-HQ (%) | $PLX_B^{\gamma}$ (%) | Yield (%) | Mass Balance (%) | $A\gamma^{Oxid}/A\beta^{Oxid}$ | $A^{Oxid}/B^{Oxid}$ |
|---|---|---|---|---|---|---|---|---|---|---|---|---|
| L7 | 60/40 | 15 | 0 | 4 | 16 | 15 | 3 | 9 | 47 | 60 | 11/89 | 81/19 |
| L8 | 63/37 | 18 | 0 | 6 | 16 | 18 | 4 | 9 | 53 | 71 | 14/86 | 83/17 |
| L9 | 52/48 | 19 | 0 | 3 | 22 | 8 | 4 | 5 | 42 | 61 | 8/92 | 82/12 |
| L10 | 52/48 | 9 | 0 | 5 | 22 | 8 | 5 | 7 | 47 | 56 | 12/88 | 85/15 |
| L11 | 57/43 | 15 | 0 | 5 | 21 | 8 | 6 | 5 | 45 | 60 | 12/88 | 89/11 |

**Table S3:** Summary and mass balance of Cu-directed oxidation of L8 under varying reaction conditions (all reactions were done in acetone unless mentioned)

| Conditions | SM (%) | L (%) | $PL_A^{\gamma}$ (%) | $PL_A^{\beta\text{-ipso}}$ (%) | 4-MeO-cat (%) | 1,4-HQ (%) | $PL_B^{\gamma}$ (%) | Yield (%) | Mass Balance (%) | $A\gamma^{Oxid}$ /$A\beta^{Oxid}$ | $A^{Oxid}$ /$B^{Oxid}$ |
|---|---|---|---|---|---|---|---|---|---|---|---|
| $Cu^{I}$ /$H_2O_2$ (5 equiv.) | 18 | 0 | 6 | 16 | 18 | 4 | 9 | 53 | 71 | 14/86 | 83/17 |
| $Cu^{I}$ /$H_2O_2$ (5 equiv.)/THF | 16 | 0 | 4 | 15 | 11 | 6 | 10 | 46 | 62 | 11/89 | 78/22 |
| $Cu^{I}$ /$H_2O_2$ (5 equiv.)/$CH_3CN$ | 48 | 0 | 5 | 10 | 8 | 2 | 8 | 35 | 83 | 19/81 | 77/23 |
| $Cu^{I}$ /$H_2O_2$ (5equiv.)/$CH_2Cl$ | 36 | 0 | 3 | 5 | 19 | 1 | 5 | 33 | 69 | 11/89 | 85/15 |
| $Cu^{I}$ /$O_2$ | 40 | 0 | 4 | 14 | 0 | 0 | 4 | 22 | 62 | 22/78 | 82/18 |
| $Cu^{II}$ /$OH^-$/$H_2O_2$ (5 equiv.) | 13 | 0 | 13 | 17 | 8 | 6 | 18 | 61 | 74 | 30/70 | 70/30 |
| $Cu^{I}$ /$H_2O_2$ (1.5 equiv.) | 29 | 0 | 4 | 25 | 5 | 2 | 3 | 39 | 68 | 11/89 | 77/23 |
| $Cu^{I}$ /$H_2O_2$ (2.5 equiv.) | 32 | 0 | 4 | 20 | 10 | 2 | 5 | 41 | 73 | 11/89 | 88/12 |
| $Cu^{I}$ /$H_2O_2$ (10 equiv.) | 14 | 0 | 6 | 13 | 15 | 4 | 15 | 53 | 67 | 16/84 | 72/28 |
| $Cu^{I}$ /$H_2O_2$ (50 equiv.) | 29 | 0 | 4 | 0 | 17 | 2 | 6 | 29 | 58 | 17/83 | 79/21 |
| $Cu^{I}$ /$H_2O_2$ (100 equiv.) | 20 | 0 | 0 | 0 | 8 | 1 | 0 | 9 | 29 | 0/100 | 100/0 |
| $Cu^{I}$ /'Dry' $H_2O_2$ (5 equiv.) | 17 | 0 | 8 | 19 | 13 | 2 | 7 | 49 | 66 | 19/81 | 86/14 |
| $Cu^{I}$ /$H_2O_2$ (5 equiv.)/$H_2O$ (70 equiv.) | 12 | 0 | 5 | 14 | 13 | 5 | 11 | 48 | 60 | 14/86 | 77/23 |
| $Cu^{I}$ /$H_2O_2$ (5 equiv.)/$H_2O$ (100 equiv.) | 15 | 0 | 5 | 10 | 14 | 8 | 10 | 47 | 62 | 13/87 | 83/17 |

## 9. UV-vis Spectroscopy

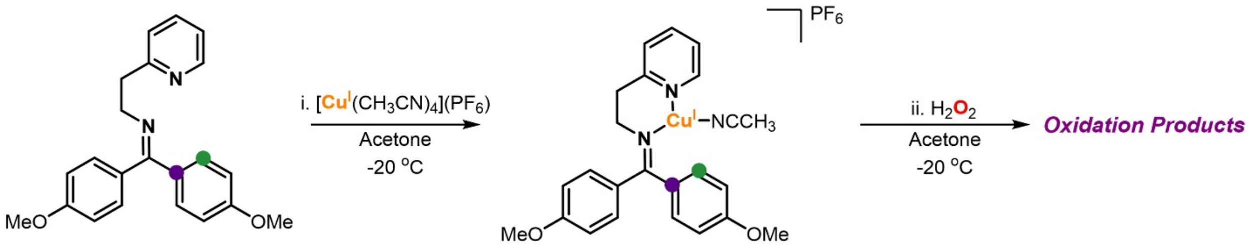


### 9.1. Oxidation of L1 with $[Cu^{I}(CH_3CN)_4](PF_6)$ and $H_2O_2$

2.8 mL of a solution of 0.5 mM L1 in acetone was placed in a 10 mm path quartz cell equipped with a stir bar and capped with a rubber septum. The solution was taken out of the glovebox and cooled to -20 ºC. After cooling 100 µL of an acetone solution containing 0.5 mM $[Cu^{I}(CH_3CN)_4](PF_6)$ was added and the spectrum was recorded. After the addition of $[Cu^{I}(CH_3CN)_4](PF_6)$, 100 µL of an acetone solution containing 20 equiv of $H_2O_2$ was added (note: the solution of $H_2O_2$ was deoxygenated by Ar/vacuum cycles before being injected into the Cu(I) complex). The reaction spectral changes were recorded every 1 second for 3600 seconds.

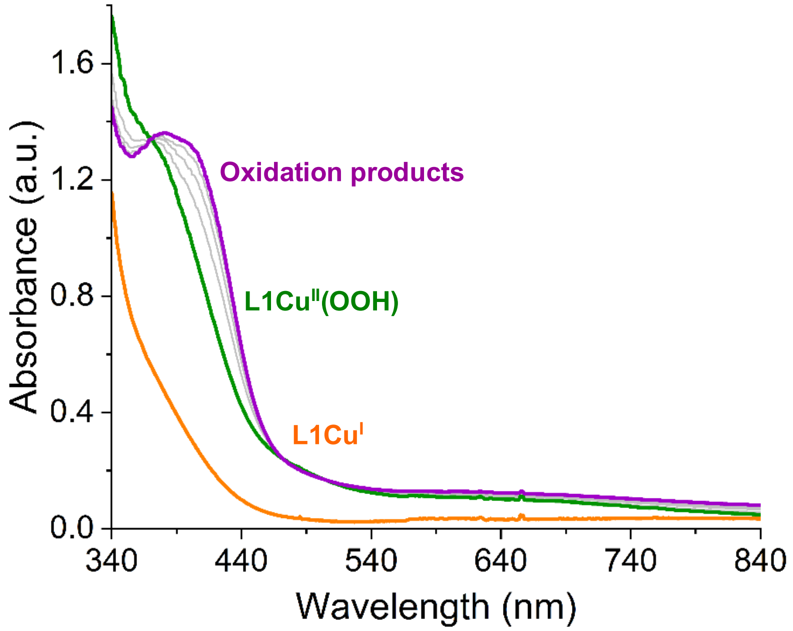


**Figure S61.** Oxidation of L1 with 0.5mM $[Cu^{I}(CH_3CN)_4](PF_6)$ and 10 mM $H_2O_2$ at -20 °C monitored by UV-vis with the possible intermediates and the graph of the evolution of the oxidation products.

*Kinetic analysis*: The reaction of Ligand with $Cu^{I}$ and $H_2O_2$ to form the putative $LCu^{II}OH$ and $LCu^{II}OOH$ intermediates is very fast (1-5 seconds after the addition of the oxidant to the cuprous complex). Kinetic analysis was performed by fitting the exponential formation of the oxidation products. We propose that the decay of the $LCu^{II}(OOH)$ is the rate-determining step and that this reaction leads to the formation of the oxidation products during the first 200 seconds of the reaction. After that, we observe a second slow process which we propose is associated with the transformation of $(L\text{-}O)Cu^{II}(OH_2)$ to $(L\text{-}O)Cu^{II}$ (200 - 3000 seconds).

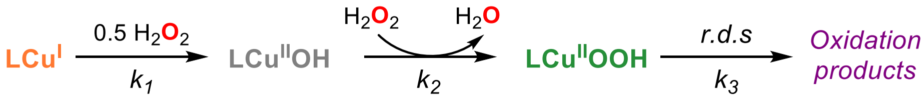


$$k_3 <<< k_1, k_2 \longrightarrow \frac{d\,[\text{Oxid. prod.}]}{dt} = \frac{-d\,[LCu^{II}OOH]}{dt} = -k_3\,[LCu^{II}OOH]$$

The rate of oxidation product formation could be calculated by fitting the change in absorbance between 5 to 200 seconds to a single exponential function:

$$Abs_t = Abs_f + (Abs_0 - Abs_f) \cdot e^{(-kobs \cdot t)}$$

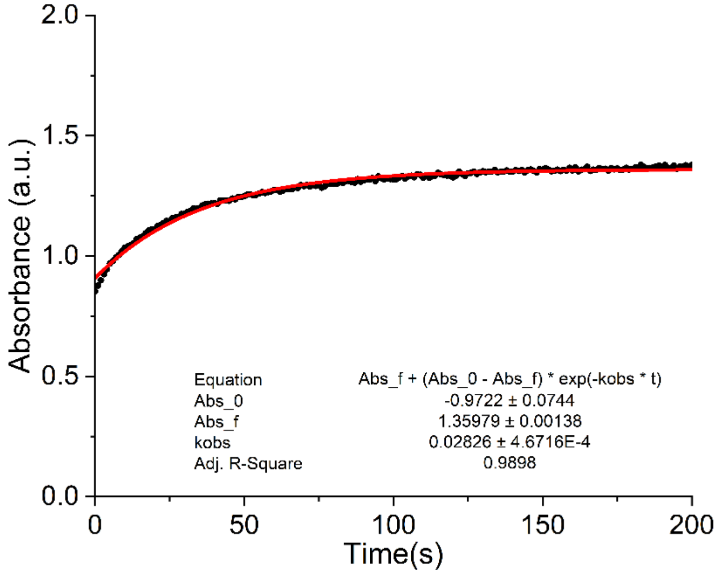


**Figure S62.** Single exponential fitting of the changes in absorbance at 410 nm from 5 to 200s obtained in the oxidation of L1 with 0.5mM $[Cu^{I}(CH_3CN)_4](PF_6)$ and 10 mM $H_2O_2$ at -20°C monitored by UV-vis (black points: experimental data; red trace: exponential fitting).

We used the same methodology to fit the UV-vis data for the oxidation of L1 under different reaction conditions ($[Cu]_0$ dependence and $[H_2O_2]$ dependence).

### 9.2. Oxidation of L1 with $[Cu^I(CH_3CN)_4](PF_6)$ and $H_2O_2$ – $[Cu]_0$ dependence

2.8 mL of a solution of 0.5 mM L1 in acetone was placed in a 10 mm path quartz cell equipped with a stir bar and capped with a rubber septum. The solution was taken out of the glovebox and cooled to -20 °C. After cooling 100 μL of an acetone solution containing 0.5 mM, 0.25 mM, or 0.125 mM $[Cu^I(CH_3CN)_4](PF_6)$ was added and the spectrum was recorded. After the addition of $[Cu^I(CH_3CN)_4](PF_6)$, 100 μL of an acetone solution containing 20 equiv of $H_2O_2$ was added (note: the solution of $H_2O_2$ was deoxygenated by Ar/vacuum cycles before being injected into the Cu(I) complex). The reaction spectral changes were recorded every 1 second for 3600 seconds for each varied reaction.

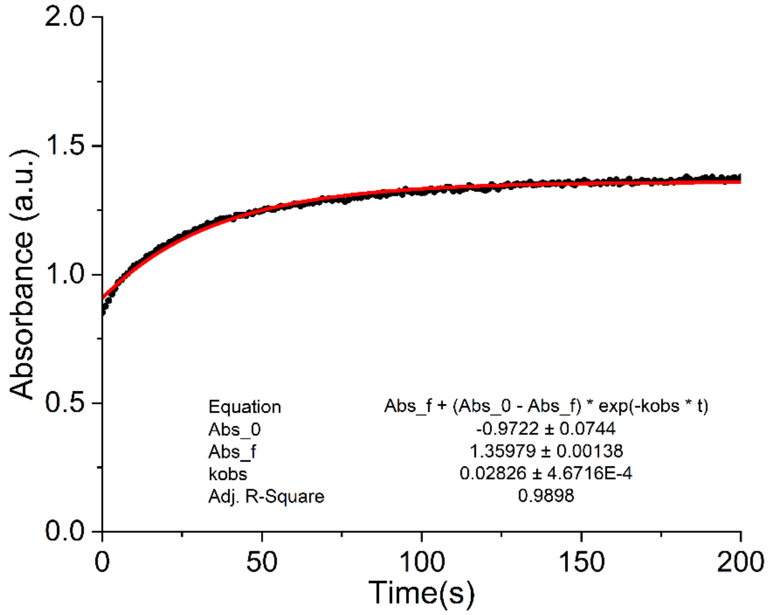


**Figure S63.** Single exponential fitting of the changes in absorbance at 410 nm from 5 to 200s obtained in the oxidation of L1 with 0.5mM $[Cu^I(CH_3CN)_4](PF_6)$ and 10 mM $H_2O_2$ at -20°C monitored by UV-vis (black points: experimental data; red trace: exponential fitting).

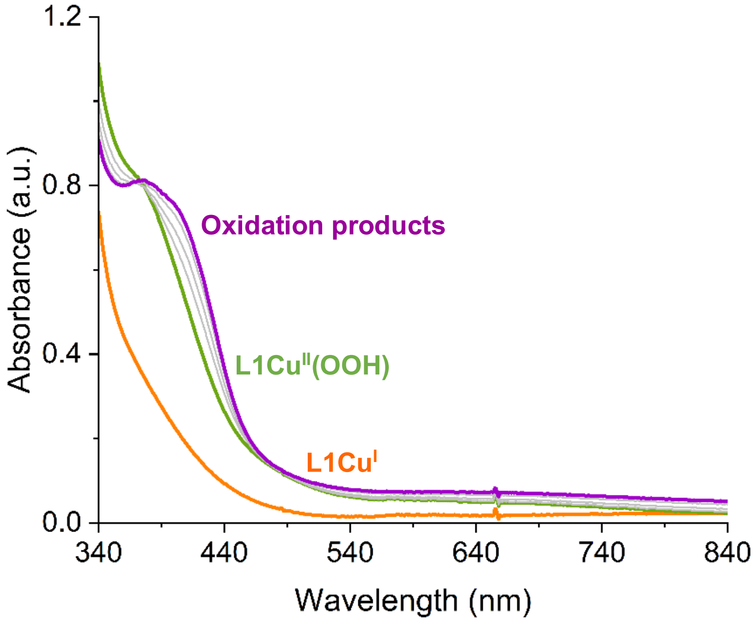


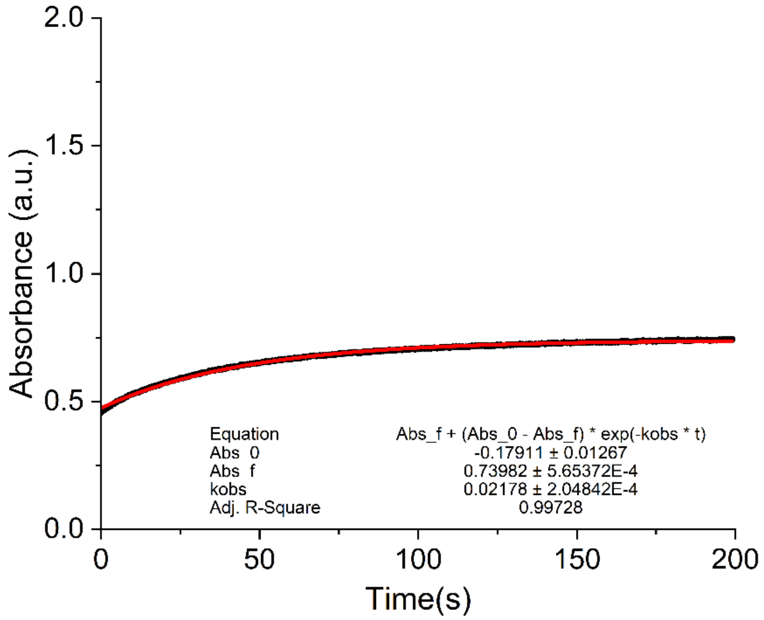


**Figure S64.** Single exponential fitting of the changes in absorbance at 410 nm from 5 to 200s obtained in the oxidation of L1 with 0.25mM $[Cu^{I}(CH_3CN)_4](PF_6)$ and 10 mM $H_2O_2$ at -20°C monitored by UV-vis (black points: experimental data; red trace: exponential fitting).

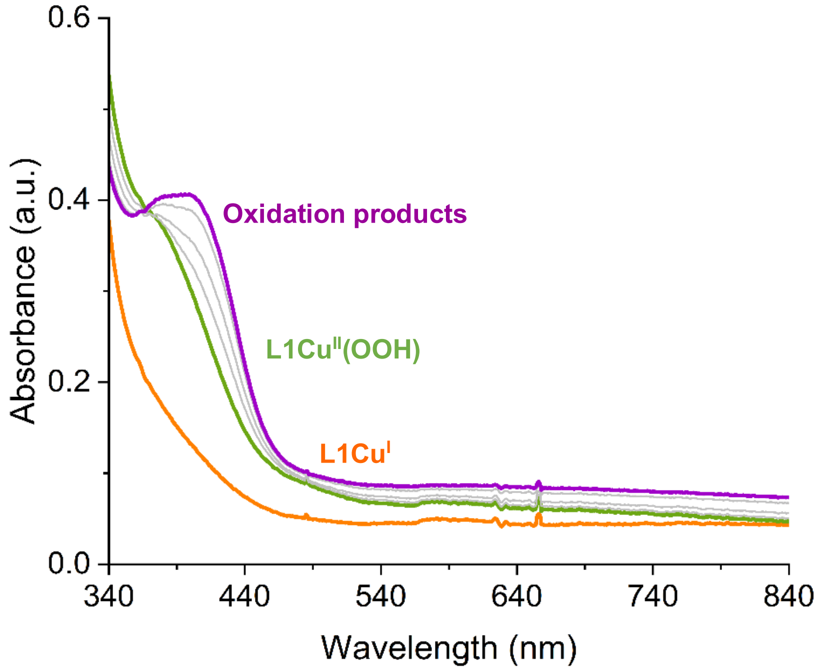


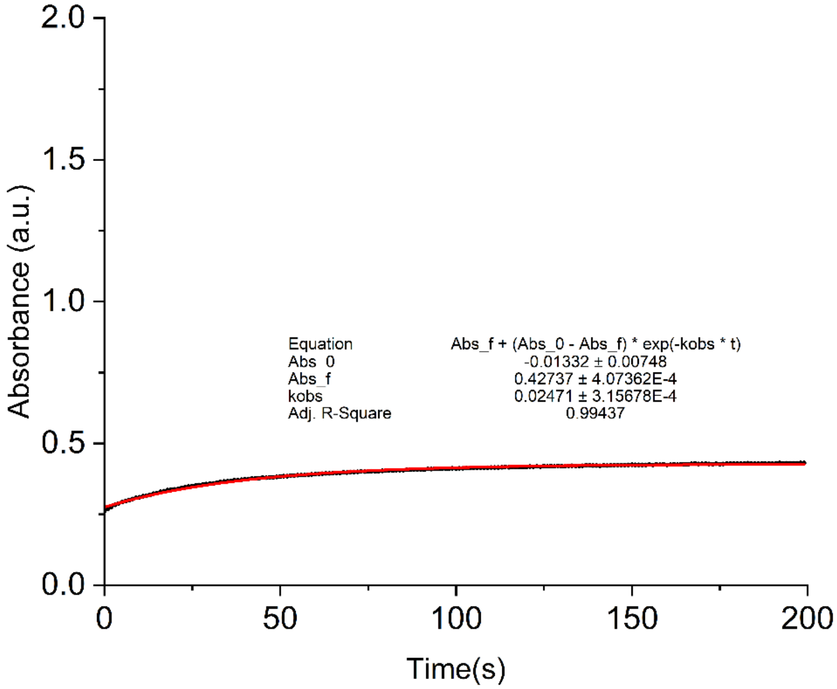


**Figure S65.** Single exponential fitting of the changes in absorbance at 410 nm from 5 to 200s obtained in the oxidation of L1 with 0.125mM $[Cu^{I}(CH_3CN)_4](PF_6)$ and 10 mM $H_2O_2$ at -20°C monitored by UV-vis (black points: experimental data; red trace: exponential fitting).

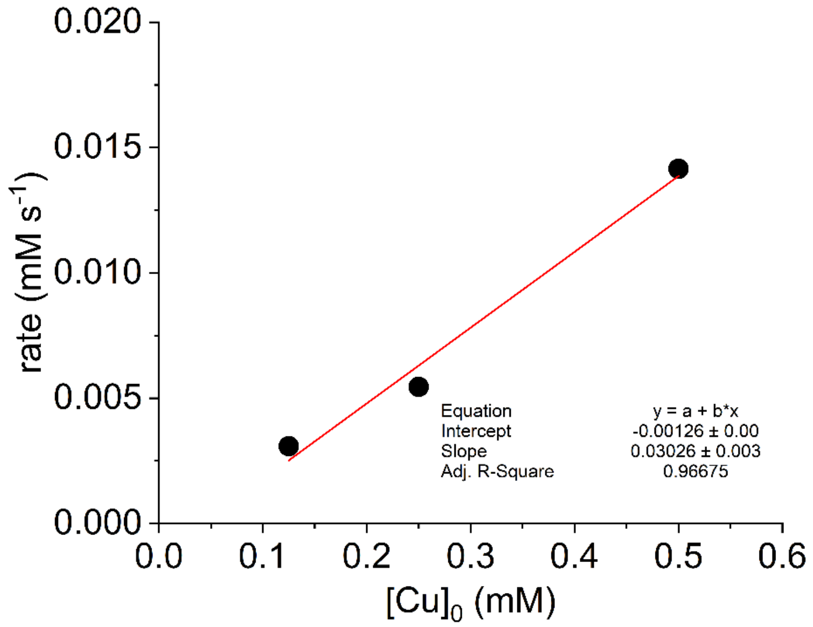


**Figure S66.** Rate dependence on the concentration of $[Cu^{I}(CH_3CN)_4](PF_6)$.

### 9.3. Oxidation of L1 with $[Cu^{I}(CH_3CN)_4](PF_6)$ and $H_2O_2$ – $[H_2O_2]_0$ dependence

2.8 mL of a solution of 0.5 mM L1 in acetone was placed in a 10 mm path quartz cell equipped with a stir bar and capped with a rubber septum. The solution was taken out of the glovebox and cooled to -20 °C. After cooling 100 µL of an acetone solution containing 0.5 mM $[Cu^{I}(CH_3CN)_4](PF_6)$ was added and the spectrum was recorded. After the addition of $[Cu^{I}(CH_3CN)](PF_6)$, 100 µL of an acetone solution containing 5, 10, 20, or 30 equiv of $H_2O_2$ was added (note: the solution of $H_2O_2$ was deoxygenated by Ar/vacuum cycles before being injected into the Cu(I) complex). The reaction spectral changes were recorded every 1 second for 3600 seconds for each varied reaction.

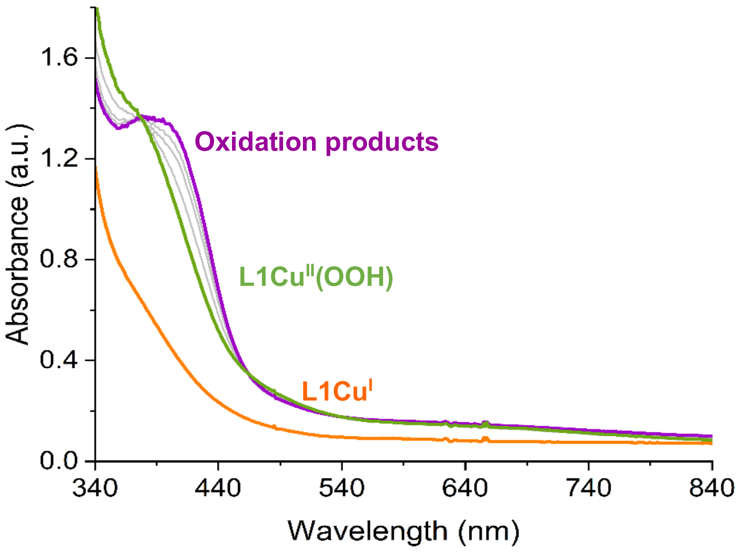


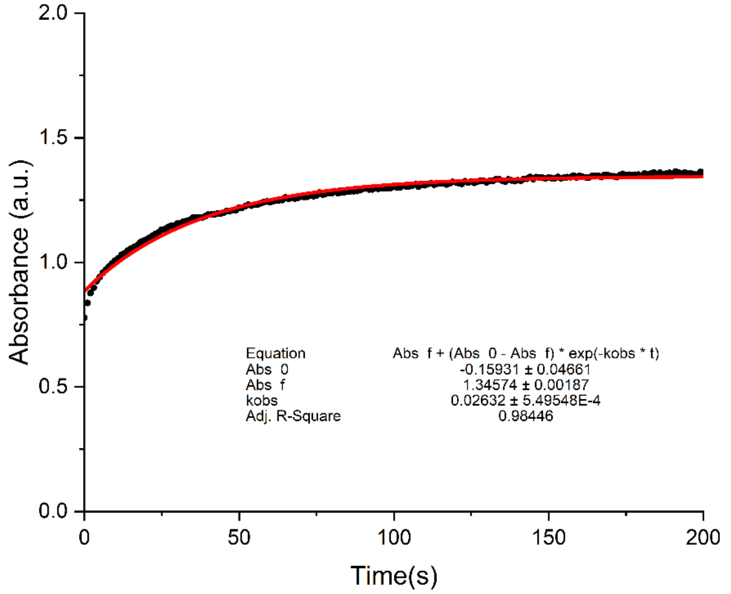


**Figure S67.** Single exponential fitting of the changes in absorbance at 410 nm from 5 to 200s obtained in the oxidation of L1 with 0.5mM $[Cu^{I}(CH_3CN)_4](PF_6)$ and 15 mM $H_2O_2$ at -20°C monitored by UV-vis (black points: experimental data; red trace: exponential fitting).

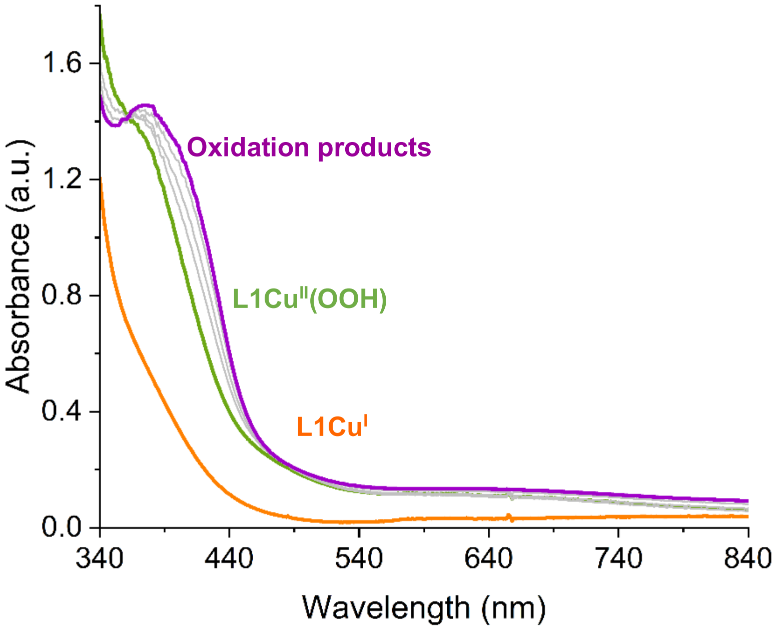


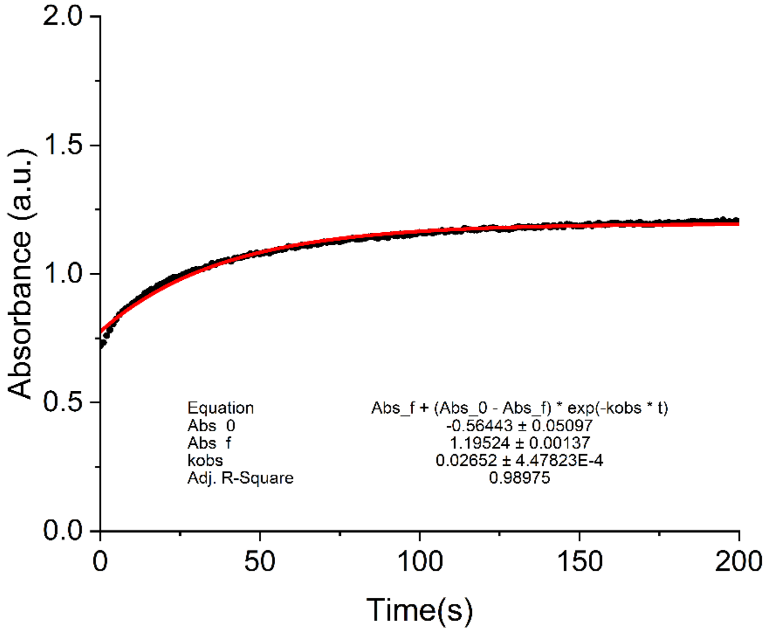


**Figure S68.** Single exponential fitting of the changes in absorbance at 410 nm from 5 to 200s obtained in the oxidation of L1 with 0.5mM $[Cu^{I}(CH_3CN)_4](PF_6)$ and 5 mM $H_2O_2$ at -20°C monitored by UV-vis (black points: experimental data; red trace: exponential fitting).

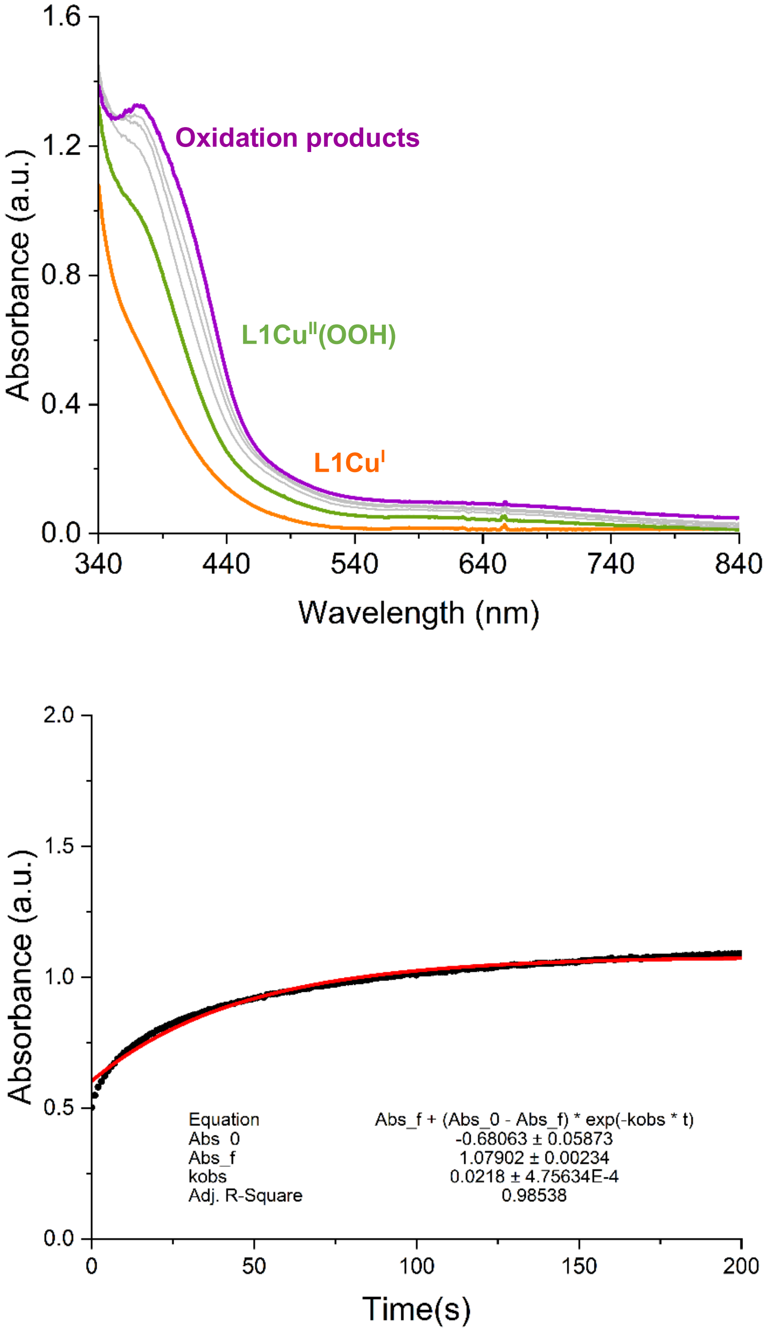


**Figure S69.** Single exponential fitting of the changes in absorbance at 410 nm from 5 to 200s obtained in the oxidation of L1 with 0.5mM $[Cu^{I}(CH_3CN)_4](PF_6)$ and 2.5 mM $H_2O_2$ at -20°C monitored by UV-vis (black points: experimental data; red trace: exponential fitting).

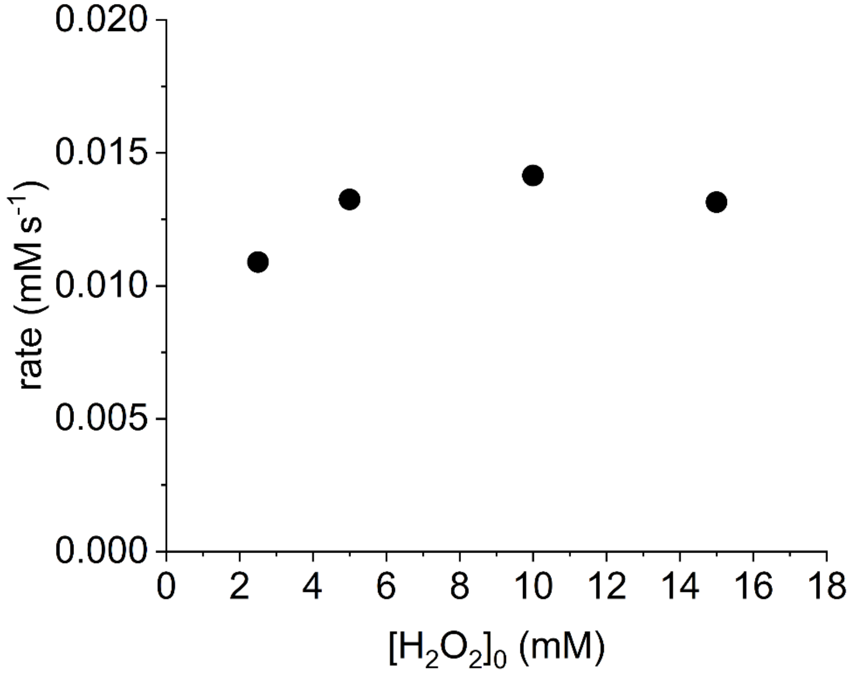


**Figure S70.** Rate dependence on the concentration of $H_2O_2$.

### 9.4. Oxidation of L1 with $Cu^{II}$, hydroxide and $H_2O_2$

2.7 mL of a solution of 0.5 mM L1 in acetone was placed in a 10 mm path quartz cell equipped with a stir bar and capped with a rubber septum. The solution was taken out of the glovebox and cooled to -20 ºC. After cooling 100 µL of an acetone solution containing 0.5 mM $Cu^{II}(CF_3SO_3)_2$ was added, and the spectrum was recorded. After the addition of $Cu^{II}(CF_3SO_3)_2$, 100 µL of an acetone solution containing 0.5mM of $Me_4NOH$ was added. Immediately after the addition of $Me_4NOH$, 100 µL of an acetone solution containing 20 equiv of $H_2O_2$ was added (note: the solution of $H_2O_2$ was deoxygenated by Ar/vacuum cycles before being injected into the Cu(I) complex). The reaction spectral changes were recorded every 1 second for 3600 seconds.

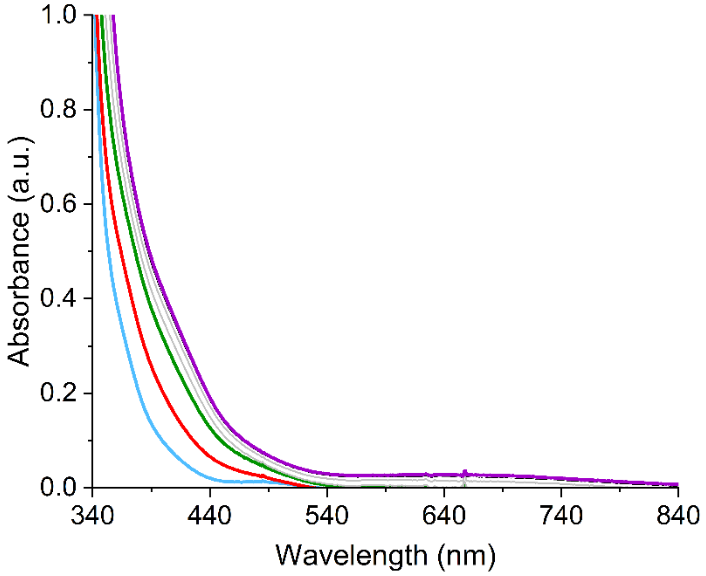


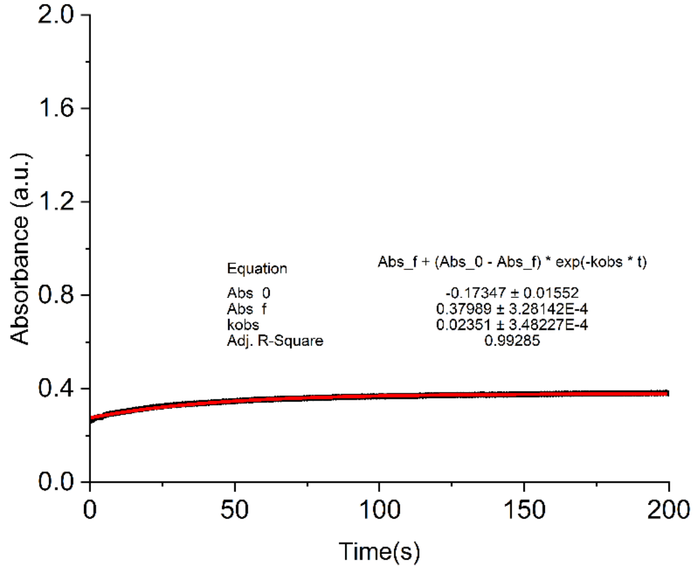


**Figure S71.** Oxidation of L1 with 0.5mM $Cu^{II}(CF_3SO_3)_2$, 0.5mM of $Me_4NOH$, and 10 mM $H_2O_2$ at -20 °C monitored by UV-vis with the possible intermediates and the graph of the evolution of the oxidation products.

### 9.5. Addition of $Cu^{II}(CF_3SO_3)_2$ to $PL8_A^{\gamma}$

2.9 mL of a solution of 0.5 mM $PL8_A^{\gamma}$ in acetone was placed in a 10 mm path quartz cell equipped with a stir bar and capped with a rubber septum. The solution was taken out of the glovebox and cooled to -20 ºC. After cooling 100 µL of an acetone solution containing 0.25 mM $Cu^{II}(CF_3SO_3)_2$ was added and the spectrum was recorded.

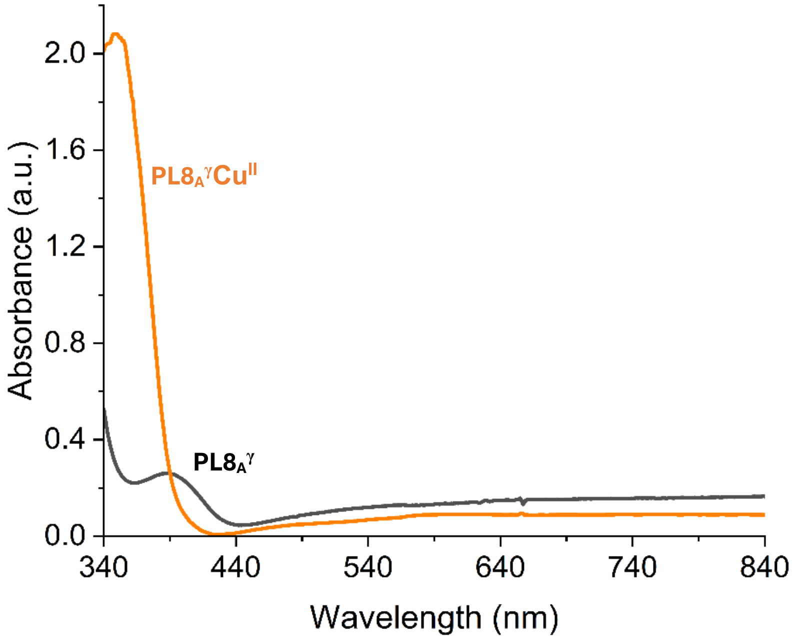


**Figure S72.** $PL8_A^{\gamma}$ with 0.25mM $Cu^{II}(CF_3SO_3)_2$ at -20 °C monitored by UV-vis. $LCu^{II}$ : $\lambda_{max}$: 350 nm, ε: 8320 $M^{-1}$ $cm^{-1}$

### 9.6. Addition of $Cu^{II}(CF_3SO_3)_2$ to $PL8_A^{\beta\text{-ipso}}$

2.9 mL of a solution of 0.5 mM $PL8_A^{\beta\text{-ipso}}$ in acetone was placed in a 10 mm path quartz cell equipped with a stir bar and capped with a rubber septum. The solution was taken out of the glovebox and cooled to -20 ºC. After cooling 100 µL of an acetone solution containing 0.25 mM $Cu^{II}(CF_3SO_3)_2$ was added, and the spectrum was recorded.

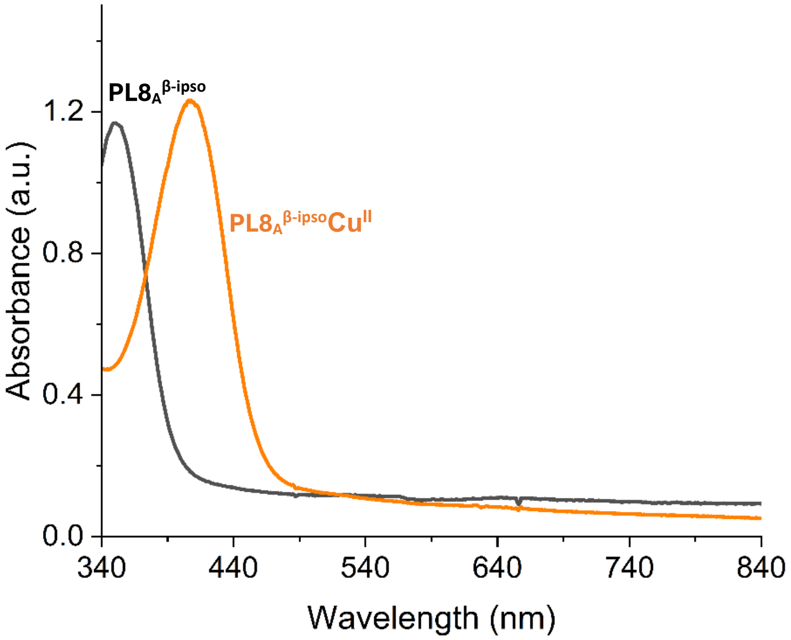


**Figure S73.** $PL8_A^{\beta\text{-ipso}}$ with 0.25mM $Cu^{II}(CF_3SO_3)_2$ at -20 ºC monitored by UV-vis. $LCu^{I}$ : $\lambda_{max}$: 408nm, ε: 4920 $M^{-1}$ $cm^{-1}$

## 10. Evolution of reaction yields over time

### 10.1. Oxidation of L1

In the glovebox, 5 mL of acetone was added to a 20-mL vial containing 0.318 mmol of L1 and equipped with a stir bar. To the solution, 1 mL of a solution containing 0.318 mmol of 1,3,5-trimethoxybenzene (internal standard) was added. The solution was taken out of the glovebox and quickly cooled to -20 °C. To the solution, 2 mL of a solution containing 0.318 mmol of $[Cu^{I}(CH_3CN)_4](PF_6)$ was added and immediately aliquot of 1 mL of the solution was quenched. Then 5 equiv of $H_2O_2$ were added and aliquots of 1 mL of the solution were quenched after 5 s, 10 s, 30 s, 60 s, 90 s, 120 s, 150 s, and 200 s in a vial containing 9 mL of chilled $Na_2EDTA$ (pH = 4). After all the times were quenched, EtOAc (9 mL X 3) was added. The organic phases were separated, combined, dried over $MgSO_4$, filtered, and dried under vacuum. The reaction products were quantified by $^1$H-NMR using integration signals that correspond to the starting material and products with the integration signal of the internal standard.

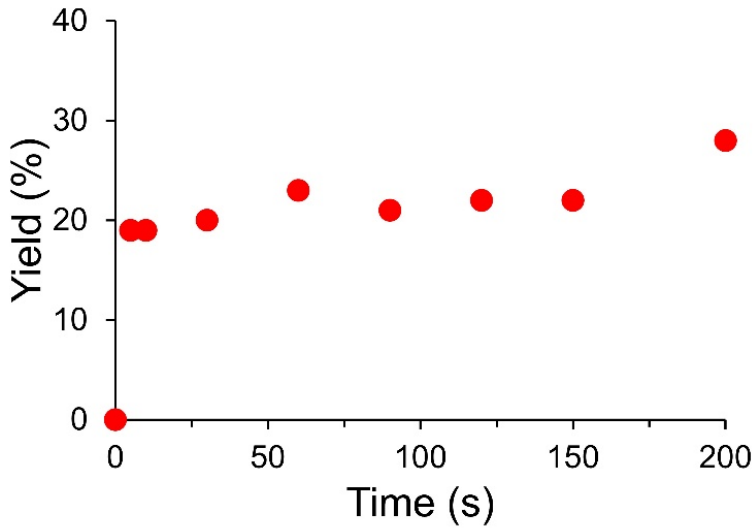


**Figure S74.** Analysis of the oxidation yields over time. In this reaction, $PL^{\beta\text{-ispo}}$ is the major product and only small amounts of catechol and hydroquinone are observed (e.g., time 200 s: $PL^{\beta\text{-ispo}}$ (21%), $PL^{\gamma}$ (0%), catechol (4%) and hydroquinone (3%).

# 11. DFT Calculations

## 11.1. Computational Details

All DFT calculations were performed with the Amsterdam Density Functional (ADF),[11,12] and QUILD[13] programs. Molecular orbitals were expanded in an uncontracted set of Slater-type orbitals (STOs) of triple-ζ quality with double polarization functions (TZ2P).[14,15] Core electrons were not treated explicitly during the geometry optimizations (frozen core approximation[12]). An auxiliary set of s, p, d, f, and g STOs was used to fit the molecular density and to represent the Coulomb and exchange potentials accurately for each SCF cycle.

Geometries were optimized with the QUILD[13] program using adapted delocalized coordinates until the maximum gradient component was less than $10^{-4}$ a.u. Energies, gradients, Hessians[16] (for vibrational frequencies) were calculated using S12g,[17] in all cases by including solvation effects through the COSMO[18] dielectric continuum model with appropriate parameters for the solvent (acetone).[19] For computing Gibbs free energies, all small frequencies were raised to 100 $cm^{-1}$ in order to compensate for the breakdown of the harmonic oscillator model.[20,21] Scalar relativistic corrections have been included self-consistently in all calculations by using the zeroth-order regular approximation (ZORA).[22] Most S12g calculations were performed with a Becke grid[23,24] of Very Good quality, except the frequencies which were computed with a Normal grid. All DFT calculations were performed using the unrestricted Kohn-Sham scheme.

All computational data have been uploaded onto the IOCHEM-BD platform (www.iochem-bd.org), DOI: https://doi.org/10.19061/iochem-bd-4-75, to facilitate data exchange and dissemination, according to the FAIR principles[25] of OpenData sharing.

**Table S4.** Sit analysis[26] for the occupation of MOs on copper, for the gamma-pathway for L9 system (oxidation of the arene with 4-MeO substituent)

| | Eigenvalues | | | | |
|---|---|---|---|---|---|
| **A** | | | | | |
| α | 0.968 | 0.978 | 0.991 | 0.991 | 0.993 |
| β | **0.621** | 0.946 | 0.947 | 0.987 | 0.990 |
| | #Occp. D-orb | 9 | | Oxid.state | 2 |
| **ts-AB** | | | | | |
| α | 0.890 | 0.930 | 0.987 | 0.998 | 0.999 |
| β | **0.639** | 0.825 | 0.986 | 0.996 | 0.999 |
| | #Occp. D-orb | 9 | | Oxid.state | 2 |
| **B** | | | | | |
| α | 0.841 | 0.911 | 0.941 | 0.946 | 0.993 |
| β | **0.798** | 0.889 | 0.912 | 0.933 | 0.963 |
| | #Occp. D-orb | 9/10 | | Oxid.state | 2/1 |
| **ts-BC** | | | | | |
| α | **0.738** | 0.904 | 0.993 | 0.996 | 1.003 |
| β | **0.649** | 0.856 | 0.993 | 0.996 | 0.999 |
| | #Occp. D-orb | 8 | | Oxid.state | 3 |
| **C** | | | | | |
| α | 0.982 | 0.990 | 0.993 | 0.995 | 0.997 |
| β | **0.571** | 0.907 | 0.929 | 0.957 | 0.992 |
| | #Occp. D-orb | 9 | | Oxid.state | 2 |
| **ts-CD** | | | | | |
| α | 0.983 | 0.990 | 0.993 | 0.995 | 1.005 |
| β | **0.587** | 0.917 | 0.941 | 0.962 | 0.985 |
| | #Occp. D-orb | 9 | | Oxid.state | 2 |
| **D** | | | | | |
| α | 0.984 | 0.986 | 0.991 | 0.993 | 1.001 |
| β | **0.586** | 0.865 | 0.976 | 0.978 | 0.980 |
| | #Occp. D-orb | 9 | | Oxid.state | 2 |

**Table S5.** Sit analysis for the occupation of MOs on copper, for *ipso*-pathway for L9 system (oxidation of the arene with 4-MeO substituent)

| | Eigenvalues | | | | |
|---|---|---|---|---|---|
| **A** | | | | | |
| α | 0.976 | 0.977 | 0.986 | 0.988 | 0.993 |
| β | **0.649** | 0.940 | 0.949 | 0.962 | 0.980 |
| | #Occp. D-orb | 9 | | Oxid.state | 2 |
| **ts-AO** | | | | | |
| α | 0.856 | 0.910 | 0.991 | 0.999 | 1.002 |
| β | **0.652** | 0.833 | 0.986 | 0.998 | 1.002 |
| | #Occp. D-orb | 9 | | Oxid.state | 2 |
| **O** | | | | | |
| α | **0.577** | 0.963 | 0.995 | 0.998 | 1.001 |
| β | **0.605** | 0.954 | 0.962 | 0.972 | 0.999 |
| | #Occp. D-orb | 8 | | Oxid.state | 3 |
| **ts-OF** | | | | | |
| α | **0.688** | 0.871 | 0.993 | 0.999 | 1.000 |
| β | **0.694** | 0.863 | 0.975 | 0.976 | 1.000 |
| | #Occp. D-orb | 8 | | Oxid.state | 3 |
| **F** | | | | | |
| α | 0.940 | 0.956 | 0.978 | 0.981 | 0.987 |
| β | **0.781** | 0.862 | 0.867 | 0.903 | 0.979 |
| | #Occp. D-orb | 9/10 | | Oxid.state | 2/1 |
| **ts-FG** | | | | | |
| α | 0.968 | 0.978 | 0.995 | 0.998 | 0.998 |
| β | **0.558** | 0.842 | 0.977 | 0.989 | 0.996 |
| | #Occp. D-orb | 9 | | Oxid.state | 2 |
| **G** | | | | | |
| α | 0.989 | 0.989 | 0.991 | 0.992 | 0.998 |
| β | **0.701** | 0.828 | 0.881 | 0.956 | 0.996 |
| | #Occp. D-orb | 9 | | Oxid.state | 2 |
| **ts-GH** | | | | | |
| α | 0.982 | 0.991 | 0.995 | 0.995 | 0.997 |
| β | **0.713** | **0.739** | 0.971 | 0.978 | 0.987 |
| | #Occp. D-orb | 8 | | Oxid.state | 3 |
| **H** | | | | | |
| α | 0.980 | 0.987 | 0.991 | 0.997 | 0.999 |
| β | **0.561** | 0.910 | 0.966 | 0.974 | 0.979 |
| | #Occp. D-orb | 9 | | Oxid.state | 2 |

**Table S6.** Sit analysis for the occupation of MOs on copper, for the gamma-pathway for L9 system (oxidation of the arene 4-F substituent).

| | Eigenvalues | | | | |
|---|---|---|---|---|---|
| **A** | | | | | |
| α | 0.970 | 0.978 | 0.989 | 0.993 | 0.993 |
| β | **0.629** | 0.949 | 0.951 | 0.981 | 0.986 |
| | #Occp. D-orb | 9 | | Oxid.state | 2 |
| **ts-AB** | | | | | |
| α | 0.856 | 0.916 | 0.978 | 1.001 | 1.001 |
| β | **0.662** | 0.842 | 0.976 | 1.000 | 1.001 |
| | #Occp. D-orb | 9 | | Oxid.state | 2 |
| **B** | | | | | |
| α | 0.809 | 0.903 | 0.938 | 0.956 | 0.993 |
| β | **0.781** | 0.878 | 0.931 | 0.947 | 0.976 |
| | #Occp. D-orb | 9 | | Oxid.state | 2 |
| **ts-BC** | | | | | |
| α | 0.918 | 0.959 | 0.995 | 0.998 | 1.001 |
| β | **0.578** | 0.833 | 0.992 | 0.995 | 0.997 |
| | #Occp. D-orb | 9 | | Oxid.state | 2 |
| **C** | | | | | |
| α | 0.984 | 0.987 | 0.992 | 0.994 | 0.998 |
| β | **0.597** | 0.891 | 0.920 | 0.956 | 0.993 |
| | #Occp. D-orb | 9 | | Oxid.state | 2 |
| **ts-CD** | | | | | |
| α | 0.980 | 0.990 | 0.995 | 0.997 | 1.005 |
| β | **0.578** | 0.930 | 0.936 | 0.961 | 0.982 |
| | #Occp. D-orb | 9 | | Oxid.state | 2 |
| **D** | | | | | |
| α | 0.980 | 0.990 | 0.993 | 0.995 | 1.002 |
| β | **0.511** | 0.945 | 0.965 | 0.971 | 0.989 |
| | #Occp. D-orb | 9 | | Oxid.state | 2 |

**Table S7.** Sit analysis for the occupation of MOs on copper, *ipso*-pathway for L9 system (oxidation of the arene with 4-F substituent)

| | Eigenvalues | | | | |
|---|---|---|---|---|---|
| **A** | | | | | |
| α | 0.963 | 0.973 | 0.989 | 0.991 | 1.001 |
| β | **0.800** | 0.865 | 0.900 | 0.955 | 0.962 |
| | #Occp. D-orb | 9/10 | | Oxid.state | 2/1 |
| **ts-AO** | | | | | |
| α | 0.858 | 0.910 | 0.991 | 0.999 | 1.001 |
| β | **0.654** | 0.833 | 0.986 | 0.997 | 1.002 |
| | #Occp. D-orb | 9 | | Oxid.state | 2 |
| **O** | | | | | |
| α | **0.568** | 0.962 | 0.997 | 0.999 | 1.001 |
| β | **0.597** | 0.953 | 0.961 | 0.993 | 0.997 |
| | #Occp. D-orb | 8 | | Oxid.state | 3 |
| **ts-OF** | | | | | |
| α | **0.684** | 0.868 | 0.993 | 1.000 | 1.001 |
| β | **0.695** | 0.859 | 0.974 | 0.977 | 1.000 |
| | #Occp. D-orb | 8 | | Oxid.state | 3 |
| **F** | | | | | |
| α | 0.913 | 0.922 | 0.950 | 0.966 | 0.992 |
| β | **0.764** | 0.840 | 0.895 | 0.938 | 0.990 |
| | #Occp. D-orb | 9/10 | | Oxid.state | 2/1 |
| **ts-FG** | | | | | |
| α | 0.936 | 0.955 | 0.993 | 0.997 | 0.998 |
| β | **0.586** | 0.839 | 0.967 | 0.995 | 0.996 |
| | #Occp. D-orb | 9 | | Oxid.state | 2 |
| **G** | | | | | |
| α | 0.989 | 0.989 | 0.992 | 0.993 | 0.997 |
| β | **0.721** | 0.830 | 0.851 | 0.979 | 0.979 |
| | #Occp. D-orb | 9 | | Oxid.state | 2 |
| **ts-GH** | | | | | |
| α | 0.983 | 0.991 | 0.995 | 0.996 | 0.996 |
| β | **0.716** | **0.741** | 0.969 | 0.972 | 0.985 |
| | #Occp. D-orb | 8 | | Oxid.state | 3 |
| **H** | | | | | |
| α | 0.980 | 0.989 | 0.992 | 0.995 | 1.001 |
| β | **0.558** | 0.905 | 0.961 | 0.967 | 0.983 |
| | #Occp. D-orb | 9 | | Oxid.state | 2 |

**Table S8.** Spin density plots analysis for the intermediates formed in *gamma*-pathway for L9 system (oxidation of the arene with 4-MeO substituent).

| | |
|---|---|
| A | 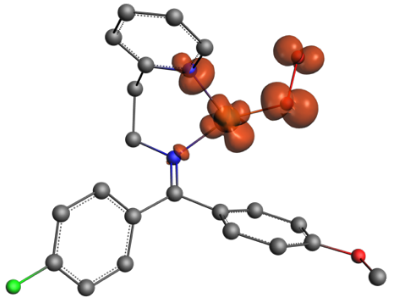 |
| AB$^{TS}$ | 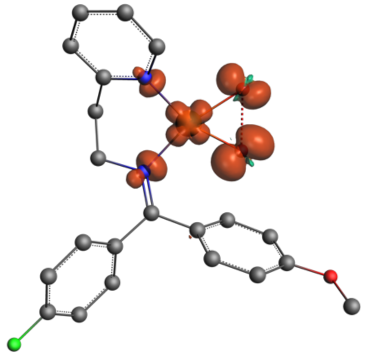 |
| B | 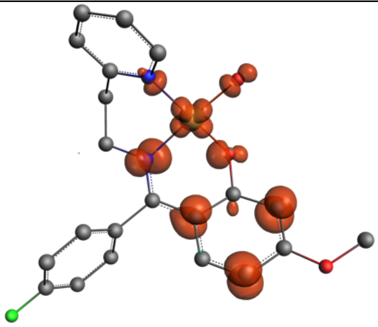 |
| BC$^{TS}$ | 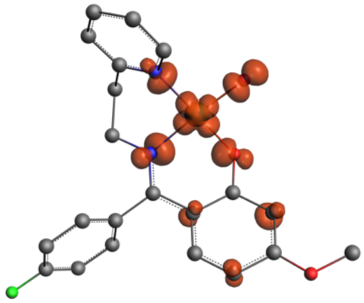 |

| | |
|---|---|
| C | 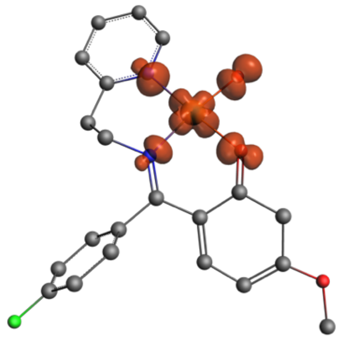 |
| $CD^{TS}$ | 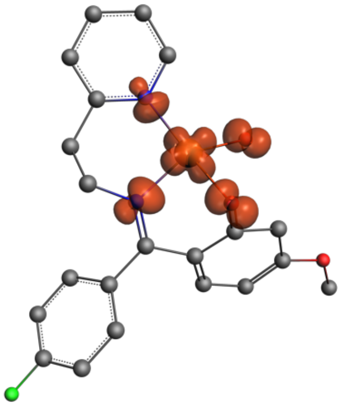 |
| D | 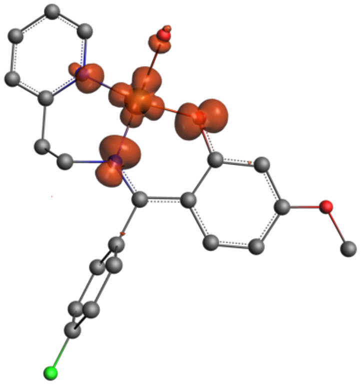 |

**Table S9.** Spin density plots analysis for the intermediates formed in *ipso*-pathway for L9 system (oxidation of the arene with 4-MeO substituent).

| | |
|---|---|
| A | 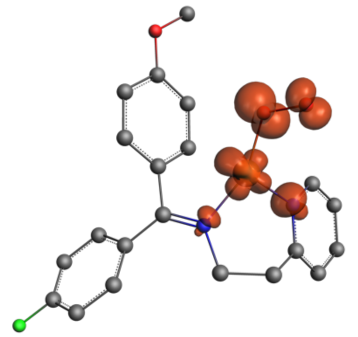 |
| $AO^{TS}$ | 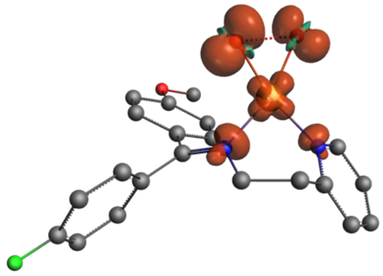 |
| O | 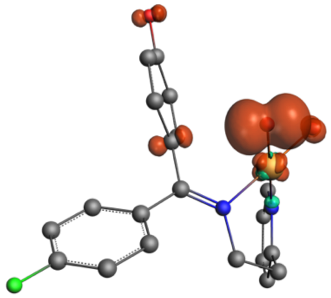 |
| $OF^{TS}$ | 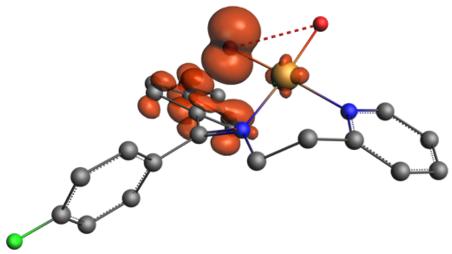 |

| | |
|---|---|
| F | 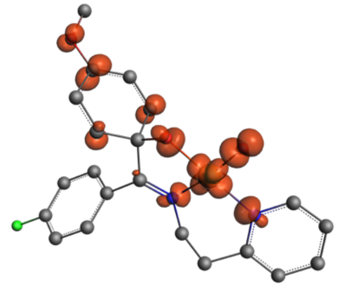 |
| FG$^{TS}$ | 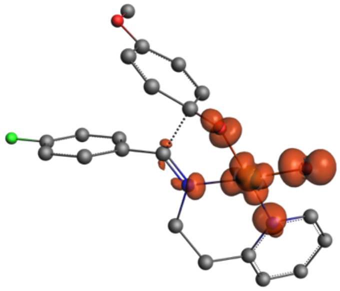 |
| G | 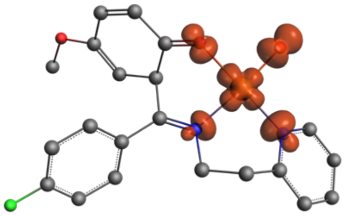 |
| GH$^{TS}$ | 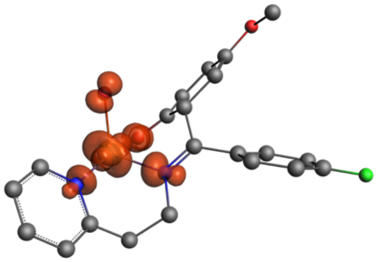 |
| H | 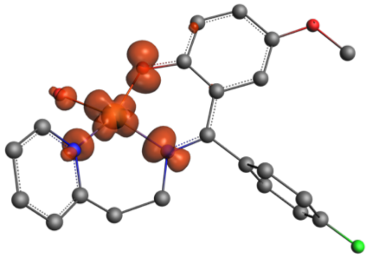 |

## 12. $^{13}$C-NMR spectra of ligand scaffolds

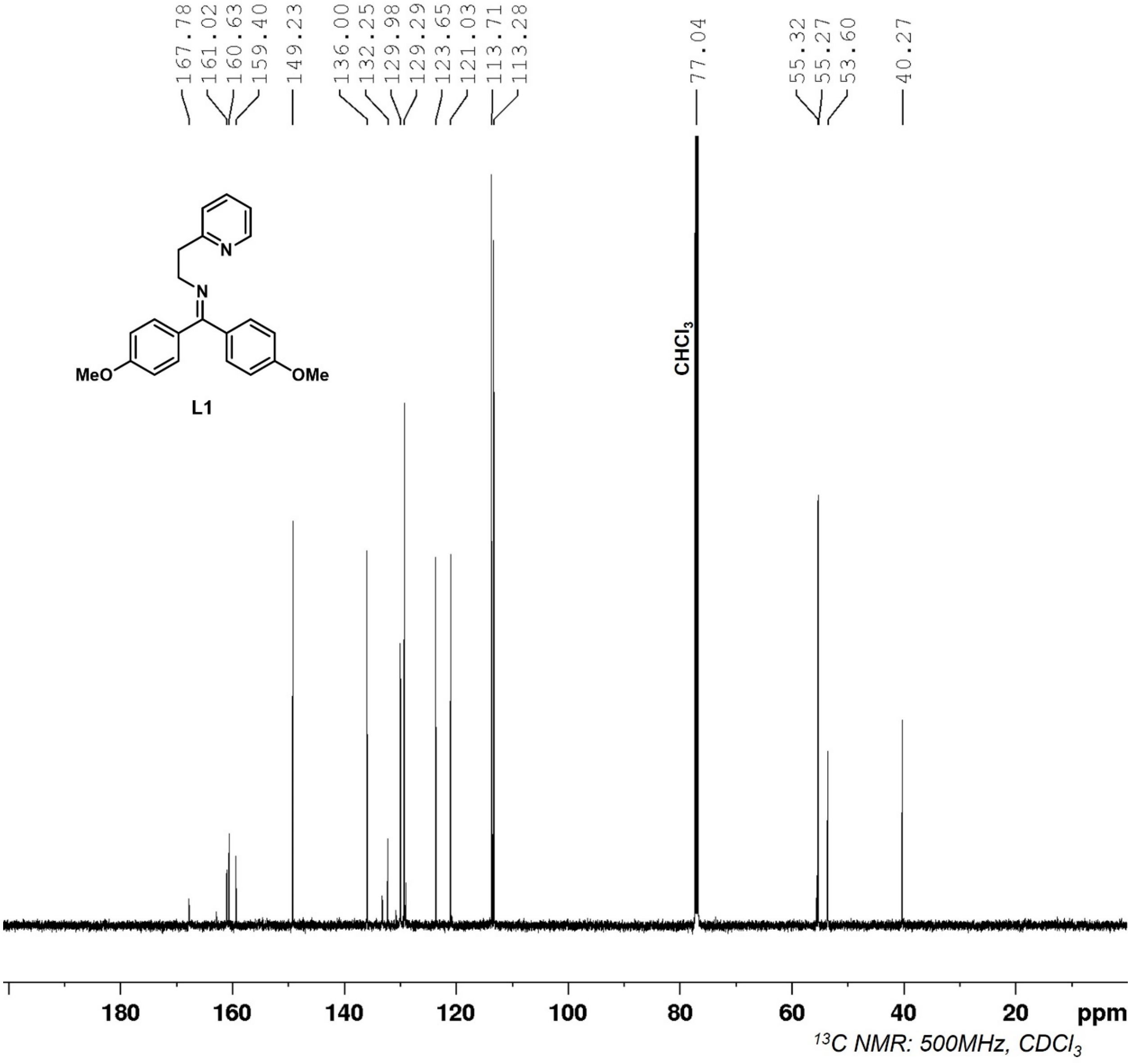

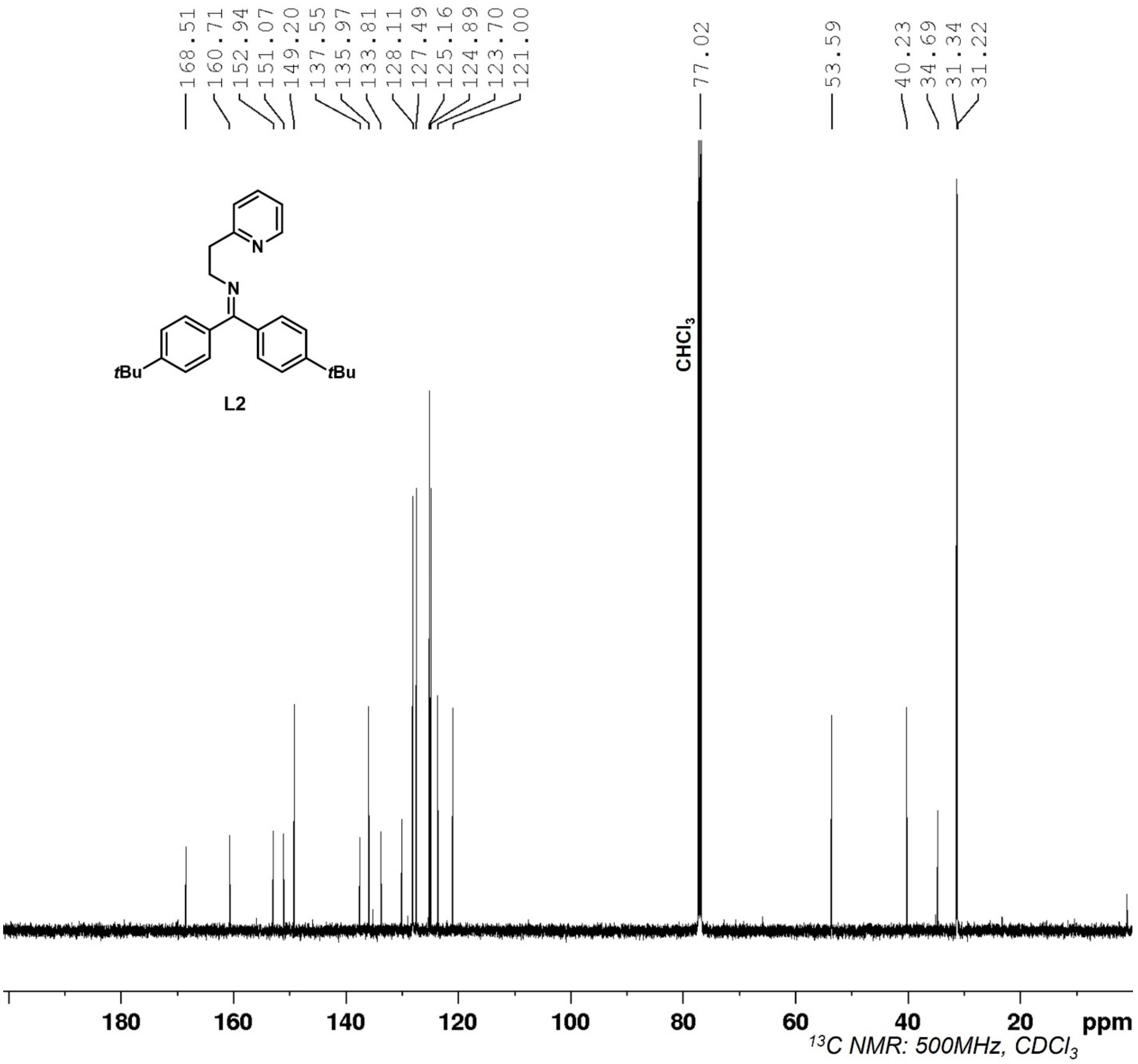
168.51
160.71
152.94
151.07
149.20
137.55
135.97
133.81
128.11
127.49
125.16
124.89
123.70
121.00
77.02
53.59
40.23
34.69
31.34
31.22
N
N
tBu
tBu
L2
CHCl3
180
160
140
120
100
80
60
40
20
ppm
13C NMR: 500MHz, CDCl3

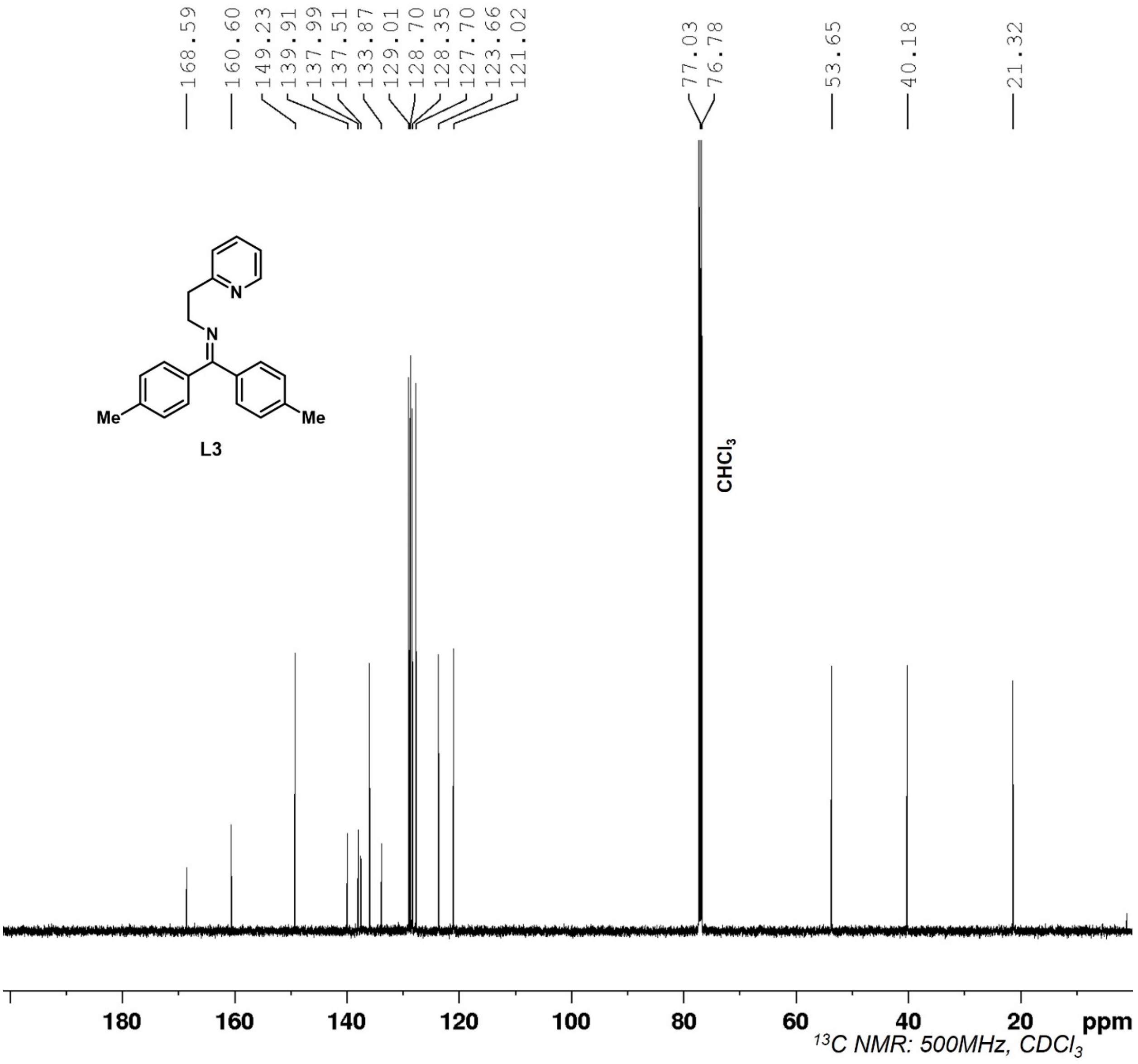
168.59
160.60
149.23
139.91
137.99
137.51
133.87
129.01
128.70
128.35
127.70
123.66
121.02
77.03
76.78
53.65
40.18
21.32
N
N
Me
Me
L3
CHCl$_3$
180
160
140
120
100
80
60
40
20
ppm
$^{13}$C NMR: 500MHz, CDCl$_3$

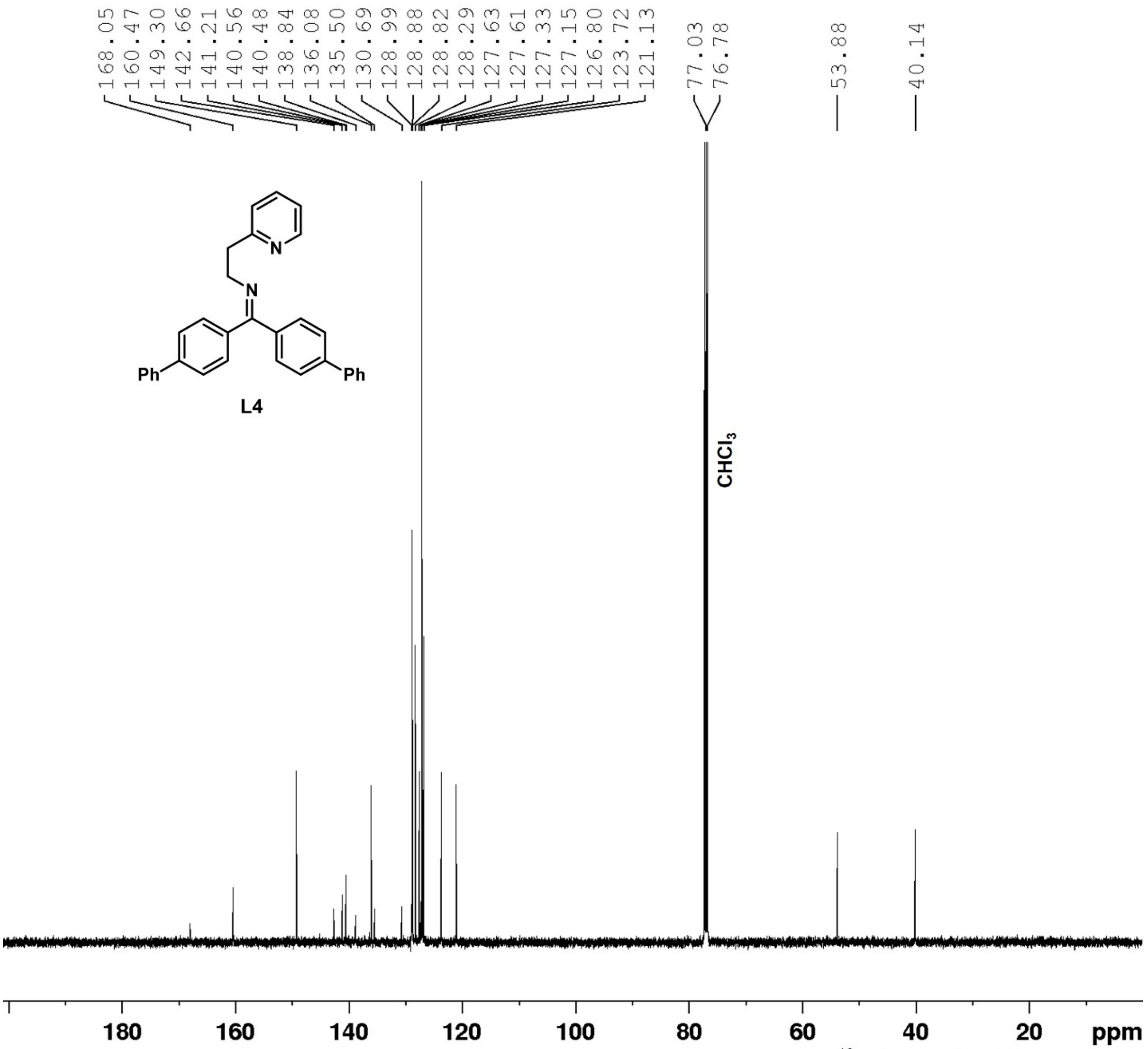


*$^{13}C$ NMR: 500MHz, $CDCl_3$*

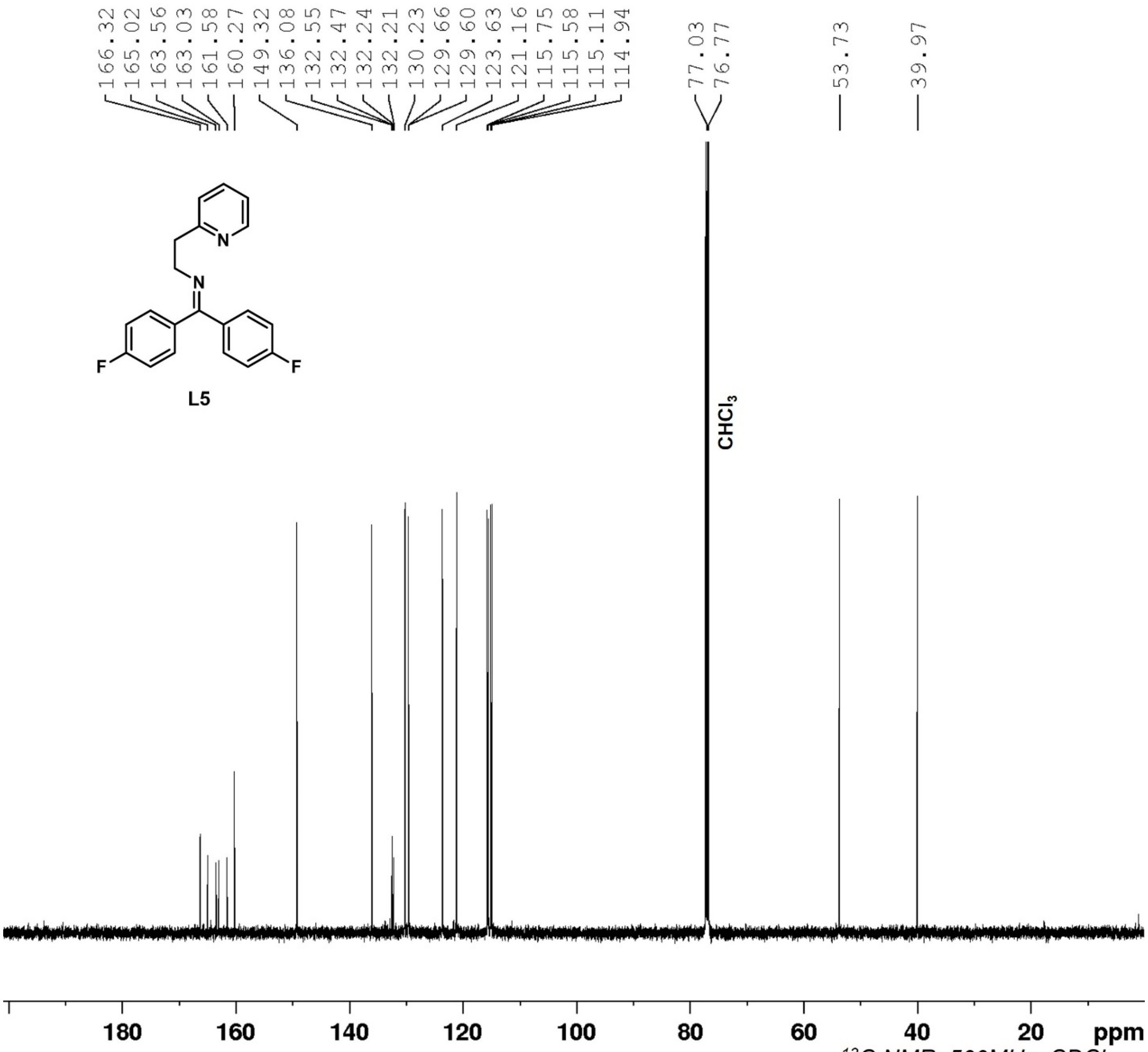


$^{13}C$ NMR: 500MHz, $CDCl_3$

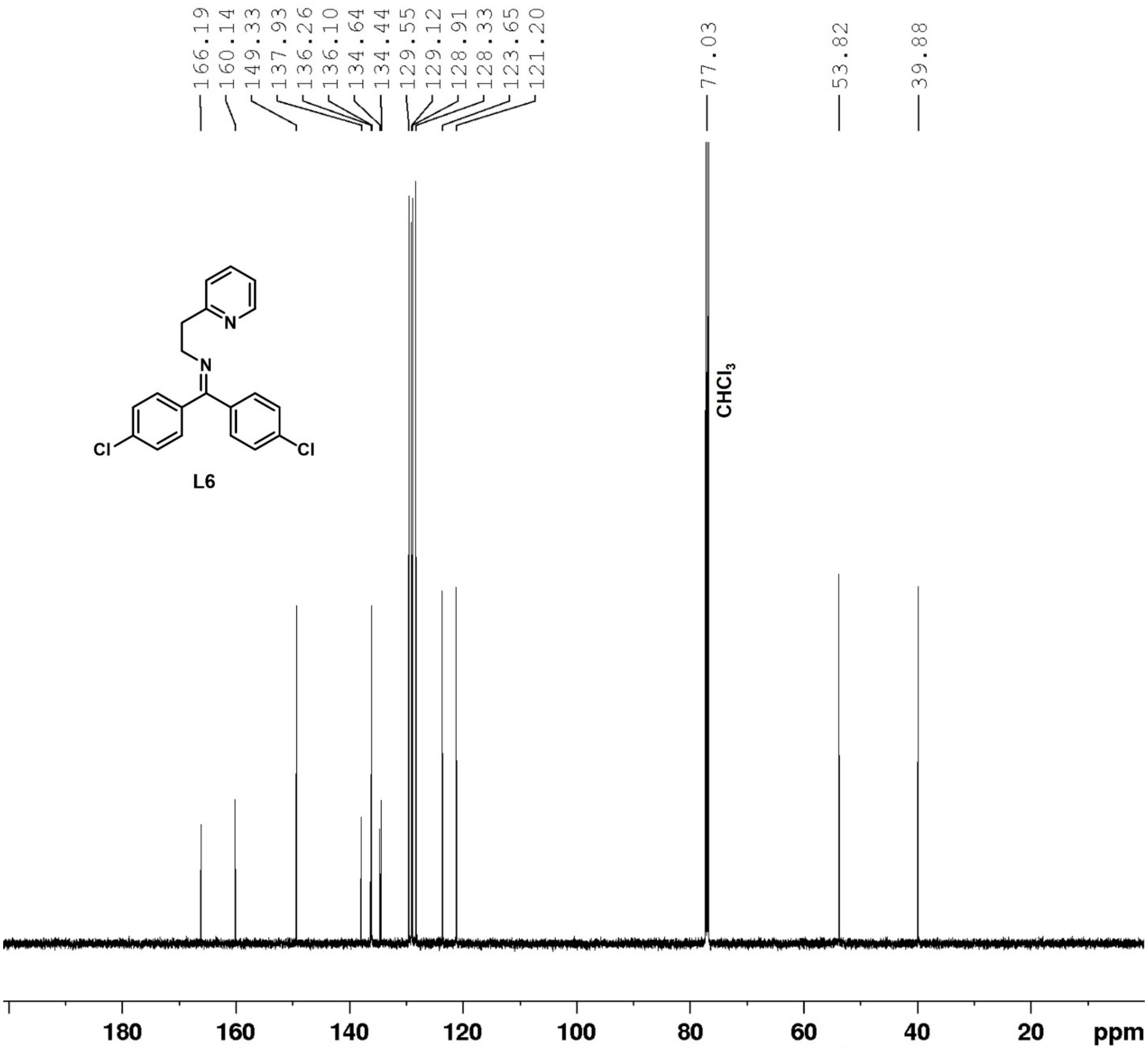

$^{13}C$ NMR: 500MHz, $CDCl_3$

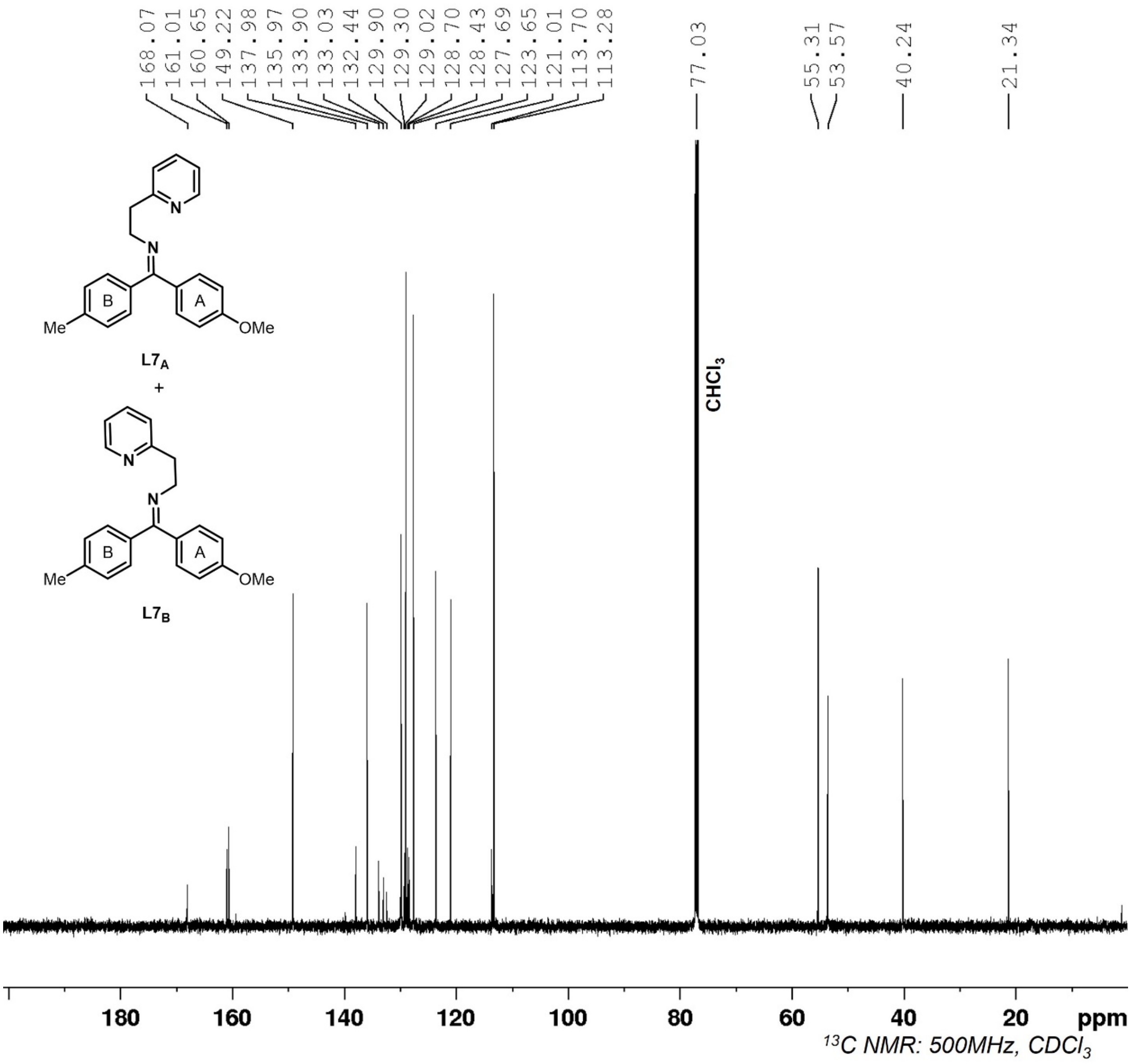
168.07
161.01
160.65
149.22
137.98
135.97
133.90
133.03
132.44
129.90
129.30
129.02
128.70
128.43
127.69
123.65
121.01
113.70
113.28
77.03
55.31
53.57
40.24
21.34
N
B
A
Me
OMe
L7A
+
L7B
CHCl3
180
160
140
120
100
80
60
40
20
ppm
13C NMR: 500MHz, CDCl3

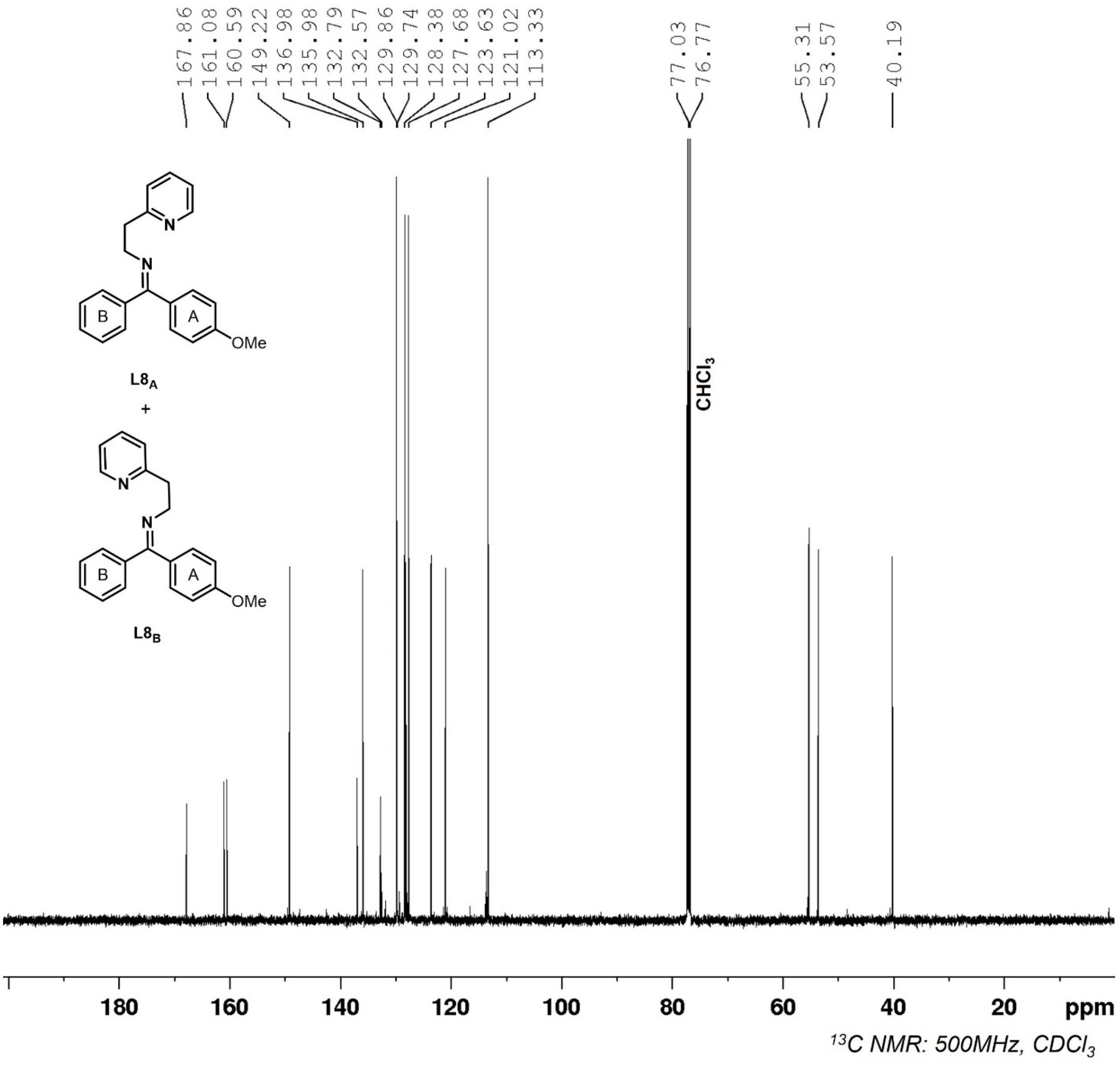
167.86
161.08
160.59
149.22
136.98
135.98
132.79
132.57
129.86
129.74
128.38
127.68
123.63
121.02
113.33
77.03
76.77
55.31
53.57
40.19
N
B
A
OMe
L8A
+
N
N
B
A
OMe
L8B
CHCl3
180
160
140
120
100
80
60
40
20
ppm
13C NMR: 500MHz, CDCl3

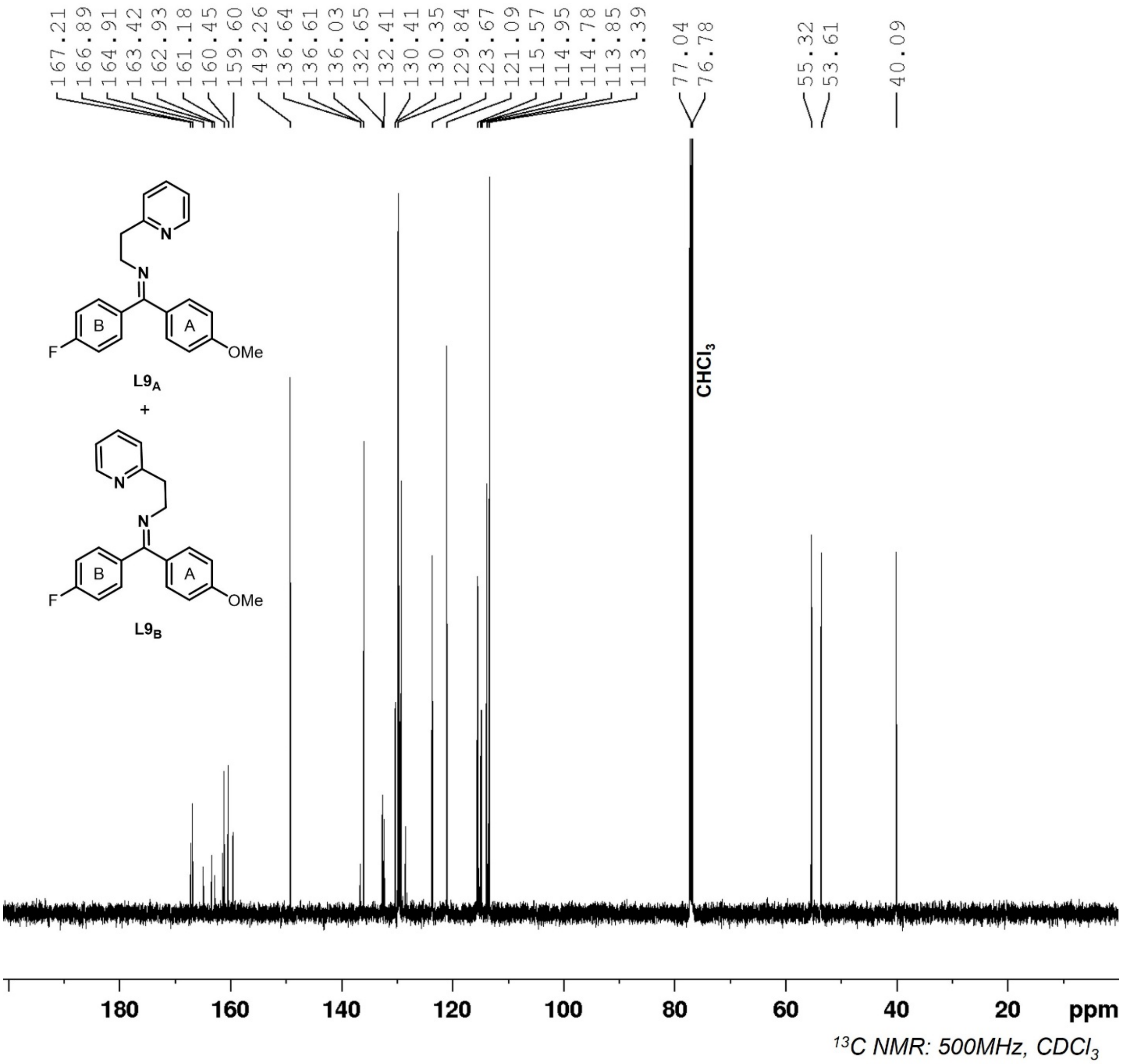
167.21
166.89
164.91
163.42
162.93
161.18
160.45
159.60
149.26
136.64
136.61
136.03
132.65
132.41
130.41
130.35
129.84
123.67
121.09
115.57
114.95
114.78
113.85
113.39
77.04
76.78
55.32
53.61
40.09
N
N
B
A
F
OMe
L9A
+
N
N
B
A
F
OMe
L9B
CHCl3
180
160
140
120
100
80
60
40
20
ppm
13C NMR: 500MHz, CDCl3

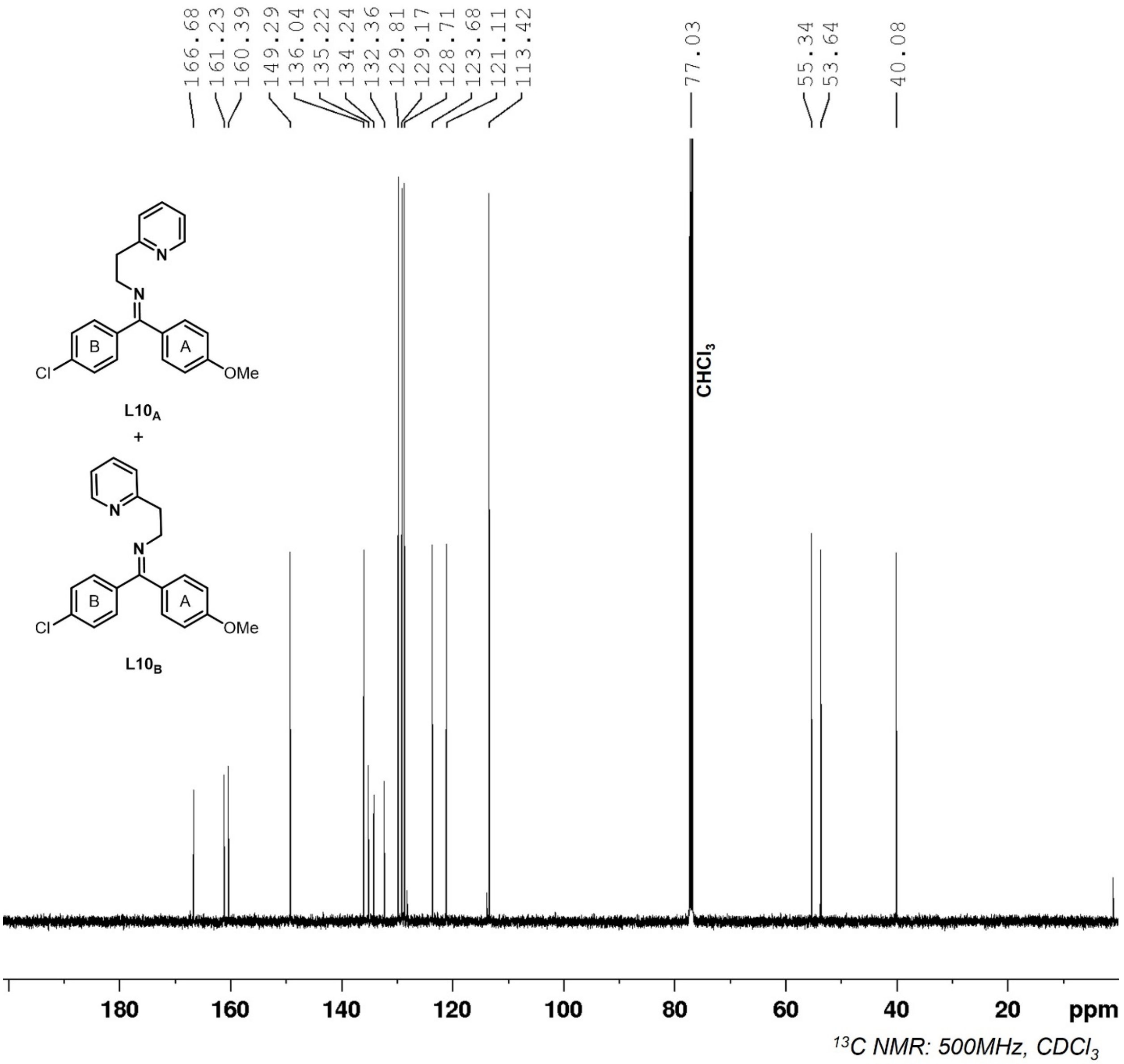
166.68
161.23
160.39
149.29
136.04
135.22
134.24
132.36
129.81
129.17
128.71
123.68
121.11
113.42
77.03
55.34
53.64
40.08
CHCl3
Cl
B
A
OMe
N
L10A
+
L10B
180
160
140
120
100
80
60
40
20
ppm
13C NMR: 500MHz, CDCl3

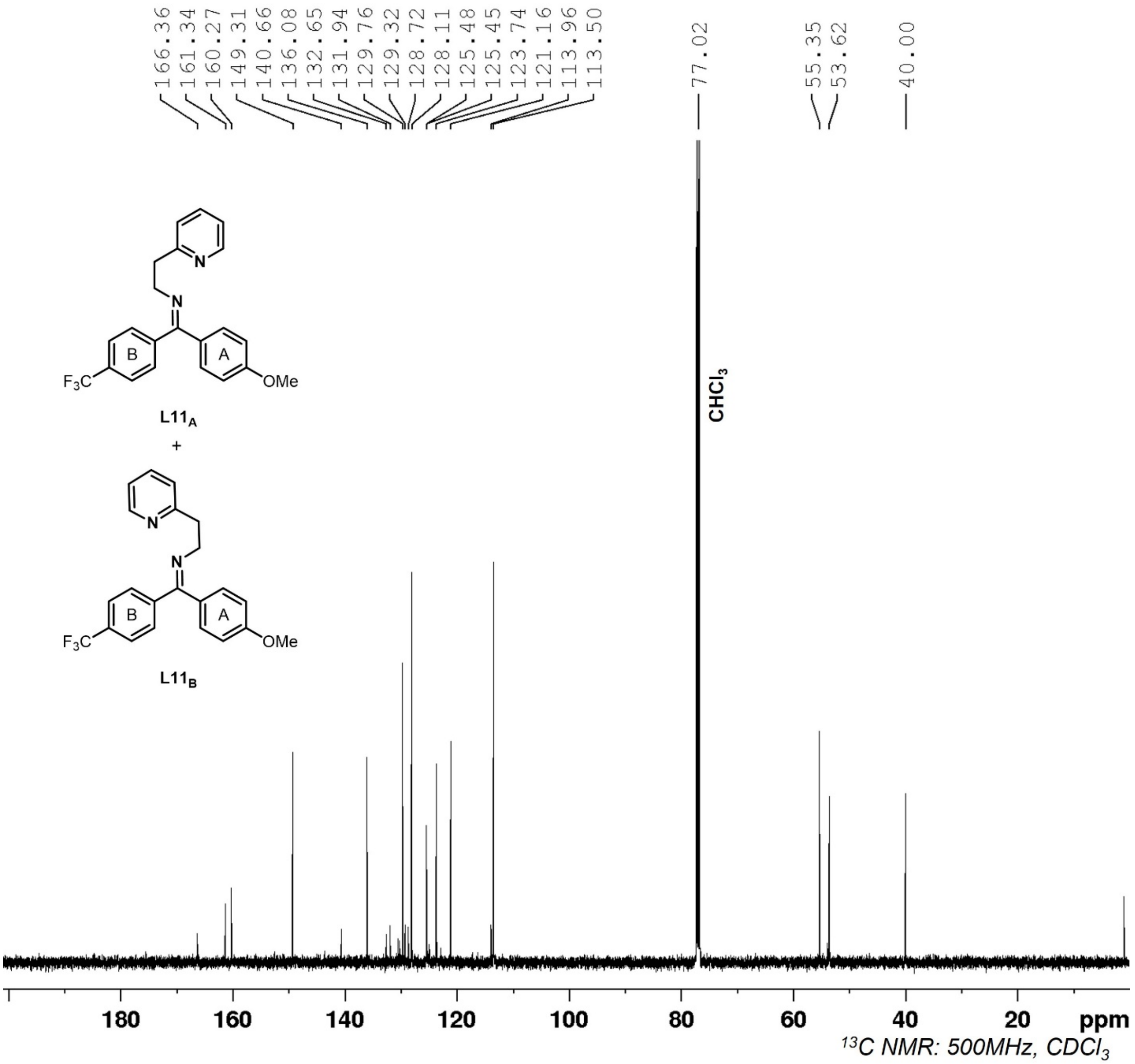

166.36
161.34
160.27
149.31
140.66
136.08
132.65
131.94
129.76
129.32
128.72
128.11
125.48
125.45
123.74
121.16
113.96
113.50
77.02
55.35
53.62
40.00
N
N
B
A
F3C
OMe
L11A
+
N
N
B
A
F3C
OMe
L11B
CHCl3
180
160
140
120
100
80
60
40
20
ppm
13C NMR: 500MHz, CDCl3

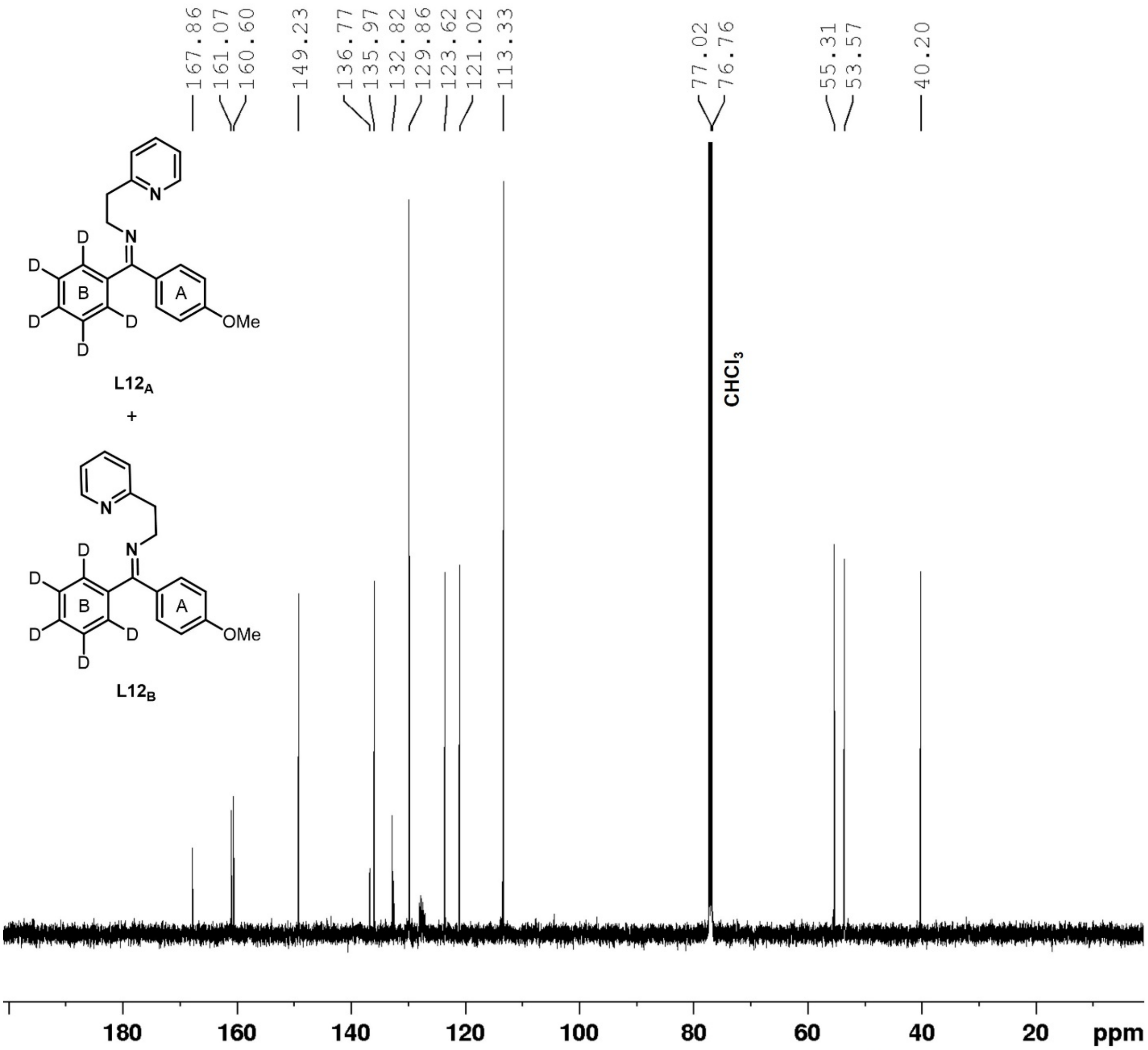


$^{13}C$ NMR: 500MHz, $CDCl_3$

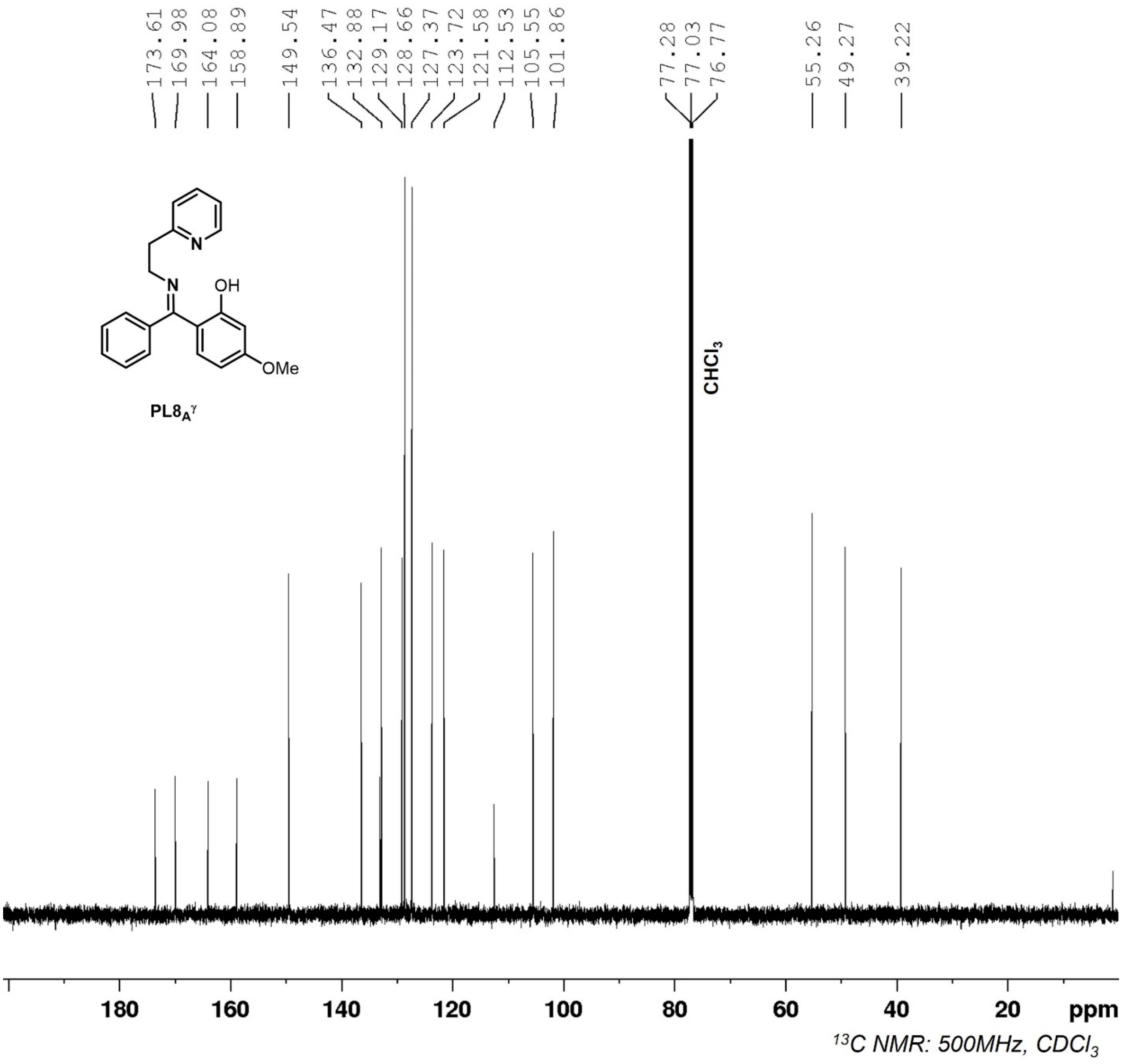
173.61
169.98
164.08
158.89
149.54
136.47
132.88
129.17
128.66
127.37
123.72
121.58
112.53
105.55
101.86
77.28
77.03
76.77
55.26
49.27
39.22
N
N
OH
OMe
PL8A γ
CHCl3
180
160
140
120
100
80
60
40
20
ppm
13C NMR: 500MHz, CDCl3

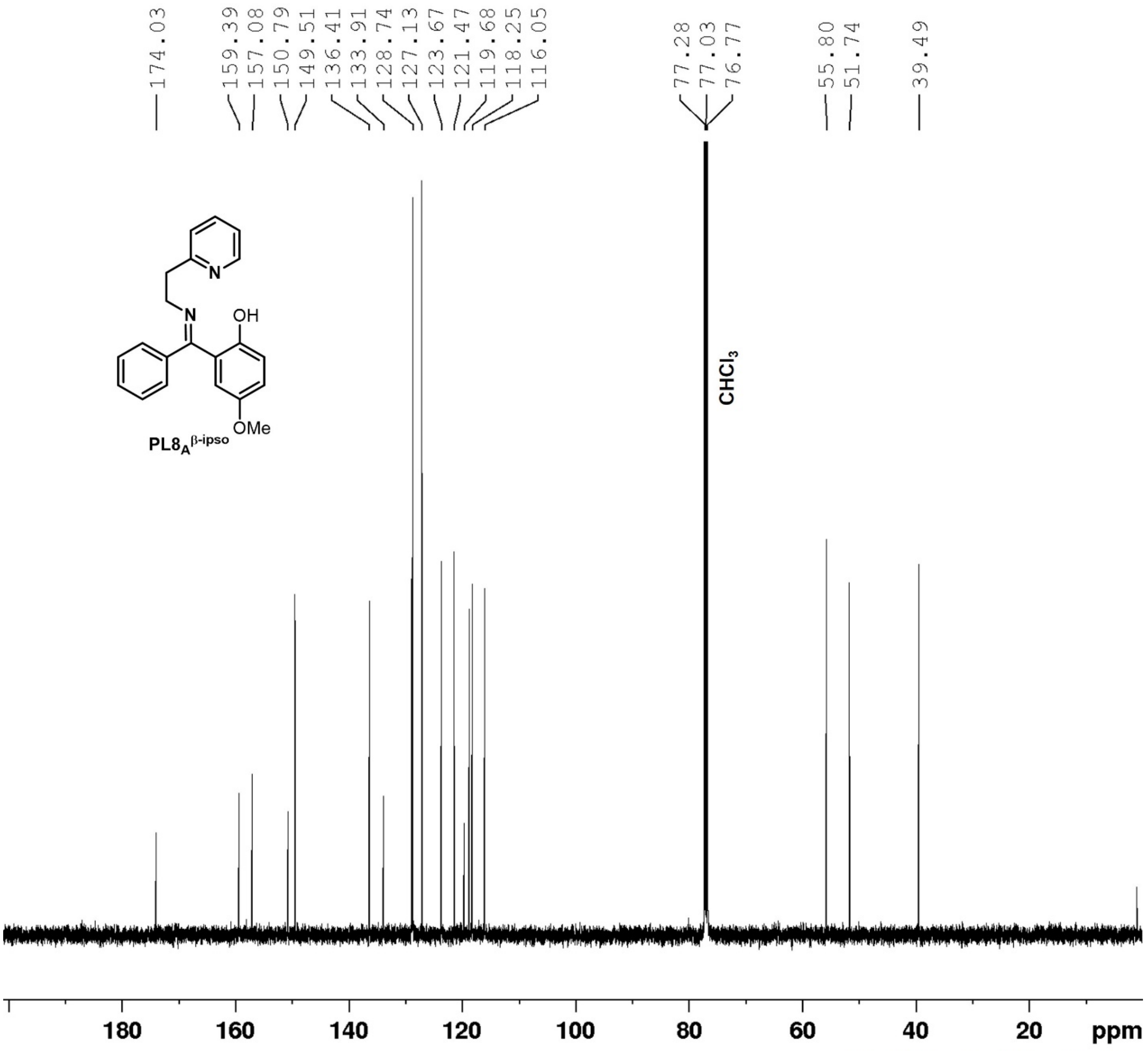


$^{13}C$ NMR: 500MHz, $CDCl_3$

## 13. References:


1. Trammell, R.; D'Amore, L.; Cordova, A.; Polunin, P.; Xie, N.; Siegler, M. A.; Belanzoni, P.; Swart, M.; Garcia-Bosch, I. Directed Hydroxylation of $sp^2$ and $sp^3$ C–H Bonds Using Stoichiometric Amounts of Cu and $H_2O_2$, *Inorg. Chem.* **2019**, *58* (11), 7584-7592.
2. Zhang, S.; Goswami, S.; Schulz, K. H. G.; Gill, K.; Yin, X.; Hwang, J.; Wiese, J.; Jaffer, I.; Gil, R. R.; Garcia-Bosch, I. Regioselective Hydroxylation of Unsymmetrical Ketones Using Cu, $H_2O_2$, and Imine Directing Groups via Formation of an Electrophilic Cupric Hydroperoxide Core. *J. Org. Chem.* 2024, *89* (4), 2622-2636.
3. Arp, F. F.; Bhuvanesh, N.; Blümel, J. Hydrogen peroxide adducts of triarylphosphine oxides *Dalton Trans.* **2019**, *48* (38), 14312-14325.
4. Choy, P. Y.; Kwong, F. Y. Palladium-Catalyzed *Ortho*-CH-Bond Oxygenation of Aromatic Ketones. *Org. Lett.* **2013**, *15* (2), 270–273.
5. Kwon. E. M. *et. al*. Preparation of Benzoyloxy Benzophenone Derivatives and Their Inhibitory Effects of ICAM-1 Expression, *Bull. Korean Chem. Soc.*, **2012**, *33*, 1939 – 1944.
6. Quandt, G.; Georg Höfner; Wanner, K. T. Synthesis and Evaluation of N-Substituted Nipecotic Acid Derivatives with an Unsymmetrical Bis-Aromatic Residue Attached to a Vinyl Ether Spacer as Potential GABA Uptake Inhibitors. *Bioorg. & Med. Chem.* **2013**, *21* (11), 3363–3378.
7. Le' an Hu; Zhang, Y.; Zhang, Q.; Yin, Q.; Zhang, X. Ruthenium-Catalyzed Direct Asymmetric Reductive Amination of Diaryl and Sterically Hindered Ketones with Ammonium Salts and $H_2$. *Angew. Chem. Int. Ed.* **2020**, *59* (13), 5321–5325.
8. Hu, J.; Adogla, E. A.; Ju, Y.; Fan, D.; Wang, Q. Copper-Catalyzed Ortho-Acylation of Phenols with Aryl Aldehydes and Its Application in One-Step Preparation of Xanthones. *Chem. Comm.* **2012**, *48* (91), 11256.
9. Feberero, C.; Sedano, C.; Suárez-Pantiga, S.; López, C. S.; Sanz, R. Experimental and Computational Study of the 1,5-O → N Carbamoyl Snieckus–Fries-Type Rearrangement. *The J. Org. Chem.* **2020**, *85* (19), 12561–12578.
10. Mo, F.; Trzepkowski, L. J.; Dong, G. Synthesis of *ortho*-Acylphenols through the Palladium-Catalyzed Ketone-Directed Hydroxylation of Arenes, *Angew. Chem. Int. Ed.,* **2012**, *51* (52), 13075–13079.
11. E.J. Baerends, T. Ziegler, A.J. Atkins, J. Autschbach, O. Baseggio, D. Bashford, A. Bérces, F.M. Bickelhaupt, C. Bo, P.M. Boerrigter, L. Cavallo, C. Daul, D.P. Chong, D.V. Chulhai, L. Deng, R.M. Dickson, J.M. Dieterich, D.E. Ellis, M. van Faassen, L. Fan, T.H. Fischer, A. Förster, C. Fonseca Guerra, M. Franchini, A. Ghysels, A. Giammona, S.J.A. van Gisbergen, A. Goez, A.W. Götz, J.A. Groeneveld, O.V. Gritsenko, M. Grüning, S. Gusarov, F.E. Harris, P. van den Hoek, Z. Hu, C.R. Jacob, H. Jacobsen, L. Jensen, L. Joubert, J.W. Kaminski, G. van Kessel, C. König, F. Kootstra, A. Kovalenko, M.V. Krykunov, E. van Lenthe, D.A. McCormack, A. Michalak, M. Mitoraj, S.M. Morton, J. Neugebauer, V.P. Nicu, L. Noodleman, V.P. Osinga, S. Patchkovskii, M. Pavanello, C.A. Peeples, P.H.T. Philipsen, D. Post, C.C. Pye, H. Ramanantoanina, P. Ramos, W. Ravenek, J.I. Rodríguez, P. Ros, R. Rüger, P.R.T. Schipper, D. Schlüns, H. van Schoot, G. Schreckenbach, J.S. Seldenthuis, M. Seth, J.G. Snijders, M. Solà, M. Stener, M. Swart, D. Swerhone, V. Tognetti, G. te Velde, P. Vernooijs, L. Versluis, L. Visscher, O. Visser, F. Wang, T.A. Wesolowski, E.M. van Wezenbeek, G. Wiesenekker, S.K.

Wolff, T.K. Woo, A.L. Yakovlev, ADF 2019.3, SCM, Theoretical Chemistry, Vrije Universiteit, Amsterdam, The Netherlands (**2019**)

12. G. te Velde, F. M. Bickelhaupt, E. J. Baerends, C. Fonseca Guerra, S. J. A. van Gisbergen, J. G. Snijders and T. Ziegler, Chemistry with ADF. *J. Comput. Chem.*, **2001**, *22*, 931–967.
13. M. Swart and F. M. Bickelhaupt, Quantum-regions Interconnected by Local Descriptions. *J. Comput. Chem.*, **2008**, *29*, 724–734.
14. E. van Lenthe and E. J. Baerends, Optimized Slater-type Basis Sets for the Elements 1–118. *J. Comput. Chem.*, **2003**, *24*, 1142-1156.
15. D. P. Chong, E. van Lenthe, S. J. A. van Gisbergen, and E. J. Baerends, Even-tempered Slater-type Orbitals Revisited: From Hydrogen to Krypton. *J. Comput. Chem.*, **2004**, *25*, 1030–1036.
16. S. K. Wolff, Analytical Second Derivatives in the Amsterdam Density Functional Package. *Int. J. Quantum Chem.*, **2005**,*104*, 645–659.
17. M. Swart, A new family of hybrid density functionals, *Chem. Phys. Let*t., **2013**, *580*, 166-171.
18. A. Klamt and G. Schüürmann, COSMO: A New Approach to Dielectric Screening in Solvents with Explicit Expressions for the Screening Energy and its Gradient. *J. Chem. Soc. Perkin Trans. 2*, **1993**, *5*, 799–805.
19. M. Swart, E. Rösler and F. M. Bickelhaupt, Proton Affinities in Water of Maingroup-Element Hydrides–Effects of Hydration and Methyl Substitution. *Eur. J. Inorg. Chem.*, **2007**, *23*, 3646–3654.
20. B. B. Averkiev and D. G. Truhlar, Free Energy of Reaction by Density Functional Theory: Oxidative Addition of Ammonia by an Iridium Complex with PCP Pincer Ligands. *Catal. Sci. Technol.*, **2011**, *1*, 1526–1529.
21. J. E. M. N. Klein, B. Dereli, L. Que Jr. and C. J. Cramer, Why Metal–oxos React with Dihydroanthracene and Cyclohexadiene at Comparable Rates, Despite Having Different C–H Bond Strengths. A Computational Study. *Chem. Commun.*, **2016**, *52*, 10509–10512.
22. E. van Lenthe, E. J. Baerends and J. G. Snijders, Relativistic Regular Two-component Hamiltonians. *J. Chem. Phys.*, **1993**, *99*, 4597–4610.
23. A. D. Becke, A multicenter numerical integration scheme for polyatomic molecules, *J. Chem. Phys.,* **1988**, *88*, 2547-2553.
24. M. Franchini, P. H. T. Philipsen, L. Visscher, *J. Comput. Chem.*, **2013**, *34*, 1819-1827.
25. M. D. Wilkinson, M. Dumontier, I. J. Aalbersberg, G. Appleton, M. Axton, A. Baak, N. Blomberg, J.-W. Boiten, L. B. da Silva Santos, P. E. Bourne, J. Bouwman, A. J. Brookes, T. Clark, M. Crosas, I. Dillo, O. Dumon, S. Edmunds, C. T. Evelo, R. Finkers, A. Gonzalez-Beltran, A. J. G. Gray, P. Groth, C. Goble, J. S. Grethe, J. Heringa, P. A. C. ’t Hoen, R. Hooft, T. Kuhn, R. Kok, J. Kok, S. J. Lusher, M. E. Martone, A. Mons, A. L. Packer, B. Persson, P. Rocca-Serra, M. Roos, R. van Schaik, S.-A. Sansone, E. Schultes, T. Sengstag, T. Slater, G. Strawn, M. A. Swertz, M. Thompson, J. van der Lei, E. van Mulligen, J. Velterop, A. Waagmeester, P. Wittenburg, K. Wolstencroft, J. Zhao and B. Mons, *Scientific Data*, **2016**, *3*, 160018.
26. P.H. Sit, R. Car, M.H. Cohen and A. Selloni, Simple, Unambiguous Theoretical Approach to Oxidation State Determination via First-Principles Calculations, *Inorg. Chem.* **2011**, *50*, 10259-10267.
