# Supplementary material for: Cu-Promoted ipso-Hydroxylation of sp2 Bonds with Concomitant Aromatic 1,2-Rearrangement Involving a Cu-oxyl-hydroxo Species
Source: Inorg Chem. 2024 Oct 18;63(43):20675–88. doi: 10.1021/acs.inorgchem.4c03304 (PMC11523237; doi:10.1021/acs.inorgchem.4c03304)
Supplement: Supplementary file 2 — ic4c03304_si_002.zip [file ic4c03304_si_002.zip › DFT Calculations/Link-Iochembd (1).docx]

Reviewer’s link to the open data on iochem-bd

<https://iochem.udg.edu/browse/review-collection/100/6112/7bbfedd2ea6f4f4f934a975b>

The URL provided in the supporting information leads to the final URL, which will be opened up to the general public after the manuscript has been accepted.

Until that time the data is under embargo for the general public, but not for the reviewers. Reviewers have access to all data using the link above.
