# Supplementary material for: Cu-Promoted ipso-Hydroxylation of sp2 Bonds with Concomitant Aromatic 1,2-Rearrangement Involving a Cu-oxyl-hydroxo Species
Source: Inorg Chem. 2024 Oct 18;63(43):20675–88. doi: 10.1021/acs.inorgchem.4c03304 (PMC11523237; doi:10.1021/acs.inorgchem.4c03304)
Supplement: Supplementary file 2 — ic4c03304_si_002.zip [file ic4c03304_si_002.zip › DFT Calculations/compdata-final.pdf]

long-FOMe-X-OMe

```
Please give the temperature (in Kelvin) at which the thermodynamics must be computed
298.15


System fwd_frq_ts_FOMe-gamma-AB-pA_1_2

Reading 1 outputfiles

       Pauli        Elstat        OrbInt       Disp.       Solv.         TOTAL          Erel   Symm.      <S2>
-----------------------------------------------------------------------------------------------------------------
-----
   28301.741     -6034.563    -29164.412     -25.329     -46.382     -6969.115         0.000   NOSYM     0.759
ADF(1)

Corresponding output files
 1 : fwd_frq_ts_FOMe-gamma-AB-pA_1_2.1282193.out

Coordinates (Angs)
  48
rev_frq_ts_FOMe-gamma-AB-pA_1_2.1282193.out        -6969.1151
C       0.000000    0.000000    5.239344
C      -1.001020    0.281857    4.308195
C      -0.961868   -0.204567    3.017277
C       0.142231   -1.121904    2.596288
C       1.151599   -1.392845    3.643411
C       1.089271   -0.832721    4.903361
C      -1.982831    0.088687    2.059644
N      -1.779284   -0.135622    0.778326
C      -2.930573   -0.163924   -0.127030
C      -1.802518    0.691912   -2.145949
N      -0.664500    1.039720   -1.521757
C       0.046152    2.101558   -1.931731
C      -0.372229    2.883507   -2.994410
C      -1.545775    2.541329   -3.655448
C      -2.264853    1.434178   -3.227821
Cu      0.000000    0.000000    0.000000
O       1.656048    0.000000   -0.804308
H      -3.796881   -1.244619    3.558095
O       0.881150   -0.670396    1.482104
H       0.965917    2.301998   -1.391657
H       0.220876    3.743259   -3.291394
H      -1.900002    3.133319   -4.496073
H      -3.186596    1.136597   -3.719618
H      -3.486265    0.779646   -0.093983
H      -1.933739   -1.374034   -1.615046
H       2.224750   -0.406796   -0.130676
H       1.221194   -0.880581    7.627881
H       3.005790   -0.916717    7.532314
H       2.122074    0.543557    7.002413
H      -0.348147   -2.095500    2.358221
H       1.991537   -2.023056    3.362729
H      -1.814627    0.934415    4.613919
H      -0.067998    0.447876    6.224857
O       2.099066   -1.127704    5.744058
H      -3.455371   -0.647410   -2.138922
C      -2.544512   -0.462659   -1.564859
H      -3.605242   -0.949274    0.234948
C       2.095915   -0.551352    7.054757
C      -3.288779    0.591828    2.549784
C      -3.685957    1.901420    2.263926
C      -4.886112    2.393671    2.756750
C      -5.682376    1.548943    3.515828
C      -5.322415    0.243413    3.809446
C      -4.108004   -0.228213    3.328631
H      -3.042371    2.550107    1.673559
H      -5.202779    3.415244    2.564646
F      -6.856491    2.021821    3.993977
H      -5.977071   -0.382283    4.409941

Frequencies (cm-1)
  100.00    100.00    100.00    100.00    100.00    100.00    100.00    100.00    100.00    104.34
  125.65    147.60    162.05    172.18    195.55    200.57    203.63    227.86    244.48    253.21
  267.08    288.12    307.13    327.05    330.52    337.07    355.57    372.26    383.43    401.55
  404.93    420.80    432.49    468.38    492.75    505.85    516.27    533.09    540.68    560.89
  570.98    577.87    605.12    610.63    640.94    650.21    694.13    714.54    732.71    741.94
  745.37    751.72    782.94    799.91    808.66    810.52    815.32    828.89    861.30    866.97
  893.37    919.29    929.59    937.45    941.11    946.37    952.05    956.62    992.87   1001.68
 1007.74   1018.07   1023.82   1032.59   1061.15   1070.83   1077.66   1094.18   1111.29   1119.91
 1140.59   1147.91   1150.70   1160.61   1165.25   1179.39   1197.04   1208.19   1220.64   1231.07
 1238.71   1267.07   1274.59   1293.38   1295.23   1322.67   1327.65   1342.93   1345.00   1357.84
 1385.81   1398.01   1407.59   1410.38   1412.88   1414.72   1428.18   1434.73   1438.14   1480.06
 1482.64   1502.31   1528.00   1565.32   1586.77   1599.40   1610.21   1614.84   2807.42   2976.87
 3006.00   3010.01   3067.82   3072.71   3104.44   3130.87   3144.83   3150.81   3154.90   3158.30
 3166.25   3171.32   3172.37   3173.24   3174.65   3185.13   3190.01   3712.45
Note: any frequencies below 100 cm-1 (including spurious imaginary ones) are upscaled to 100 cm-1
      for the calculation of thermodynamic properties.
      (Averkiev, Truhlar, Catal. Sci. Technol. 2011, 1, 1526)

Thermodynamics

Note: this script does not take into account the spin entropy
For more info, see eq. 3 of Inorg. Chem. 2002, 41, 6928-6935
(M. Reiher), https://doi.org/10.1021/ic025891l
```

```
Temperature is now: 298.150

Reporting max. of 0 frequencies (set by $GETFREQSMAX env. variable)


Reading 1 outputfiles

            ScnFrq(1)
--------------------
--------------------
(ZPVE)        228.986
(dH,0->T)      16.481
(-TS)         -49.329
(dGibbs)      196.138
====================

Corresponding output files
 1 : frq_fwd_frq_ts_FOMe-gamma-AB-pA_1_2.1284185.out


System fwd_frq_ts_FOMe-gamma-BC-pA_1_2

Reading 1 outputfiles

       Pauli        Elstat        OrbInt       Disp.       Solv.         TOTAL          Erel   Symm.      <S2>
-----------------------------------------------------------------------------------------------------------------
-----
   28448.142     -6037.870    -29348.288     -24.880     -50.401     -7013.474         0.000   NOSYM     0.752
ADF(1)

Corresponding output files
 1 : fwd_frq_ts_FOMe-gamma-BC-pA_1_2.1303891.out

Coordinates (Angs)
  48
fwd_frq_ts_FOMe-gamma-BC-pA_1_2.1303891.out        -7013.4716
C       0.000000   -0.000000    5.481069
C      -0.962110    0.191024    4.492497
C      -0.817228   -0.145349    3.140099
C       0.419260   -0.734478    2.739078
C       1.448609   -1.071325    3.780381
C       1.193865   -0.610135    5.159938
C      -1.917376    0.047228    2.209449
N      -1.773378    0.014388    0.914389
C      -2.973739    0.068987    0.072286
C      -1.902074    0.737440   -2.096017
N      -0.679989    1.010475   -1.604612
C       0.082589    1.951356   -2.178341
C      -0.348601    2.676249   -3.277433
C      -1.608691    2.407120   -3.798624
C      -2.391459    1.427732   -3.201718
Cu      0.000000    0.000000    0.000000
O       1.743731    0.000000   -0.720989
H      -3.495572   -1.841643    3.327851
O       0.735891   -1.028035    1.560472
H       1.063610    2.096387   -1.734540
H       0.298399    3.432514   -3.712533
H      -1.979236    2.954158   -4.662628
H      -3.380287    1.190371   -3.584797
H      -3.404123    1.077715    0.106763
H      -2.167440   -1.268048   -1.415769
H       2.283513   -0.478329   -0.074944
H      -3.344405    2.373619    2.412516
C       3.360102   -1.394720    5.810954
H      -6.844214    0.715101    4.253434
H       1.558199   -2.167703    3.784398
H       2.416426   -0.698969    3.419441
H      -1.894563    0.659041    4.800098
H      -0.184542    0.325355    6.500336
O       2.090161   -0.779807    6.108575
H      -3.654632   -0.446383   -1.881843
C      -2.695081   -0.305176   -1.377237
H      -3.731285   -0.613553    0.473343
H      -5.783348   -1.549182    4.239537
C      -3.273742    0.236502    2.803241
C      -3.860642    1.502267    2.807466
C      -5.132478    1.632930    3.339646
C      -5.845303    0.561453    3.853590
C      -5.244375   -0.692812    3.841439
C      -3.961011   -0.859033    3.329426
H       3.888328   -1.427116    6.762809
H       3.216351   -2.407985    5.424298
H       3.918022   -0.785847    5.092757
F      -5.701300    2.862962    3.353210

Frequencies (cm-1)
   100.00    100.00    100.00    100.00    100.00    100.00    100.00    100.00    100.00    100.78
   127.77    144.80    160.45    174.24    197.06    201.88    204.45    228.84    241.23    252.50
   259.88    289.96    309.07    317.55    325.79    332.05    357.74    373.66    385.90    402.00
   405.55    420.99    431.75    469.07    493.17    503.54    514.82    532.11    542.70    558.79
   570.38    577.57    600.97    612.87    639.72    650.06    695.50    708.52    728.57    741.39
   744.33    753.38    780.46    799.87    808.34    810.15    813.91    829.22    860.69    867.49
   891.18    918.42    926.77    937.59    939.56    945.37    951.54    955.56    992.97   1001.33
  1008.36   1020.67   1026.89   1034.63   1060.04   1068.65   1075.56   1093.63   1112.80   1119.79
```

```
 1139.12   1147.28   1152.00   1161.07   1167.48   1179.81   1189.70   1208.25   1219.64   1225.97
 1237.98   1262.82   1274.04   1292.89   1294.73   1321.53   1326.33   1342.01   1344.82   1357.36
 1382.90   1398.47   1407.88   1410.90   1414.85   1416.38   1427.34   1435.87   1439.09   1480.25
 1482.60   1502.24   1527.72   1565.98   1586.62   1598.53   1609.72   1615.59   2781.33   2976.35
 3004.23   3010.75   3070.79   3072.75   3105.54   3130.75   3143.26   3151.14   3156.57   3158.61
 3165.69   3171.35   3172.92   3173.58   3174.97   3184.85   3189.80   3715.20
Note: any frequencies below 100 cm-1 (including spurious imaginary ones) are upscaled to 100 cm-1
      for the calculation of thermodynamic properties.
      (Averkiev, Truhlar, Catal. Sci. Technol. 2011, 1, 1526)

Thermodynamics

Note: this script does not take into account the spin entropy
For more info, see eq. 3 of Inorg. Chem. 2002, 41, 6928-6935
(M. Reiher), https://doi.org/10.1021/ic025891l

Temperature is now: 298.150

Reporting max. of 0 frequencies (set by $GETFREQSMAX env. variable)


Reading 1 outputfiles

            ScnFrq(1)
---------------------
---------------------
(ZPVE)        228.864
(dH,0->T)      16.505
(-TS)         -49.370
(dGibbs)      195.999
=====================

Corresponding output files
 1 : frq_fwd_frq_ts_FOMe-gamma-BC-pA_1_2.1283706.out


System fwd_frq_ts_FOMe-gamma-BC-pA_1_2

Reading 1 outputfiles

       Pauli        Elstat        OrbInt       Disp.       Solv.         TOTAL          Erel   Symm.      <S2>
------------------------------------------------------------------------------------------------------------------------
-----
   28448.142     -6037.870    -29348.288     -24.880     -50.401     -7013.474         0.000   NOSYM     0.752
ADF(1)

Corresponding output files
 1 : fwd_frq_ts_FOMe-gamma-BC-pA_1_2.1303891.out

Coordinates (Angs)
  48
fwd_frq_ts_FOMe-gamma-BC-pA_1_2.1303891.out        -7013.4716
C       0.000000   -0.000000    5.481069
C      -0.962110    0.191024    4.492497
C      -0.817228   -0.145349    3.140099
C       0.419260   -0.734478    2.739078
C       1.448609   -1.071325    3.780381
C       1.193865   -0.610135    5.159938
C      -1.917376    0.047228    2.209449
N      -1.773378    0.014388    0.914389
C      -2.973739    0.068987    0.072286
C      -1.902074    0.737440   -2.096017
N      -0.679989    1.010475   -1.604612
C       0.082589    1.951356   -2.178341
C      -0.348601    2.676249   -3.277433
C      -1.608691    2.407120   -3.798624
C      -2.391459    1.427732   -3.201718
Cu      0.000000    0.000000    0.000000
O       1.743731    0.000000   -0.720989
H      -3.495572   -1.841643    3.327851
O       0.735891   -1.028035    1.560472
H       1.063610    2.096387   -1.734540
H       0.298399    3.432514   -3.712533
H      -1.979236    2.954158   -4.662628
H      -3.380287    1.190371   -3.584797
H      -3.404123    1.077715    0.106763
H      -2.167440   -1.268048   -1.415769
H       2.283513   -0.478329   -0.074944
H      -3.344405    2.373619    2.412516
C       3.360102   -1.394720    5.810954
H      -6.844214    0.715101    4.253434
H       1.558199   -2.167703    3.784398
H       2.416426   -0.698969    3.419441
H      -1.894563    0.659041    4.800098
H      -0.184542    0.325355    6.500336
O       2.090161   -0.779807    6.108575
H      -3.654632   -0.446383   -1.881843
C      -2.695081   -0.305176   -1.377237
H      -3.731285   -0.613553    0.473343
H      -5.783348   -1.549182    4.239537
C      -3.273742    0.236502    2.803241
C      -3.860642    1.502267    2.807466
C      -5.132478    1.632930    3.339646
C      -5.845303    0.561453    3.853590
C      -5.244375   -0.692812    3.841439
```

```
C      -3.961011   -0.859033    3.329426
H       3.888328   -1.427116    6.762809
H       3.216351   -2.407985    5.424298
H       3.918022   -0.785847    5.092757
F      -5.701300    2.862962    3.353210

Frequencies (cm-1)
  100.00    100.00    100.00    100.00    100.00    100.00    100.00    100.00    100.00    102.17
  117.02    137.15    139.25    175.37    183.99    190.83    192.86    221.24    222.51    224.96
  240.03    248.12    265.12    302.36    307.98    319.05    326.26    345.06    360.70    408.78
  413.54    419.07    445.74    471.92    487.75    488.89    495.95    510.63    523.66    539.79
  566.29    575.59    588.18    605.85    637.68    644.28    683.89    710.53    721.62    732.93
  743.99    757.48    776.78    783.27    815.64    828.68    832.96    860.17    872.44    876.07
  877.89    889.60    918.88    943.22    954.50    965.74    968.35    970.56    991.48    992.71
  994.82   1013.34   1023.01   1058.29   1070.31   1078.51   1093.89   1106.21   1108.05   1134.37
 1147.20   1149.05   1153.86   1165.42   1169.03   1187.71   1219.99   1234.40   1243.59   1252.83
 1261.08   1291.11   1293.85   1298.74   1324.03   1333.23   1336.66   1347.95   1367.68   1383.56
 1393.76   1409.63   1412.67   1414.25   1429.20   1430.77   1435.81   1456.29   1473.26   1477.99
 1519.73   1527.67   1566.09   1585.77   1590.92   1608.81   1614.88   1619.06   2990.86   2997.35
 2999.89   3005.38   3049.53   3063.07   3093.73   3102.41   3146.77   3149.55   3151.34   3152.23
 3159.48   3160.73   3162.70   3167.76   3171.39   3177.15   3178.12   3751.09
Note: any frequencies below 100 cm-1 (including spurious imaginary ones) are upscaled to 100 cm-1
      for the calculation of thermodynamic properties.
      (Averkiev, Truhlar, Catal. Sci. Technol. 2011, 1, 1526)

Thermodynamics

Note: this script does not take into account the spin entropy
For more info, see eq. 3 of Inorg. Chem. 2002, 41, 6928-6935
(M. Reiher), https://doi.org/10.1021/ic025891l

Temperature is now: 298.150

Reporting max. of 0 frequencies (set by $GETFREQSMAX env. variable)


Reading 1 outputfiles

            ScnFrq(1)
---------------------
---------------------
(ZPVE)        229.430
(dH,0->T)      16.785
(-TS)         -50.000
(dGibbs)      196.215
=====================

Corresponding output files
 1 : frq_fwd_frq_ts_FOMe-gamma-BC-pA_1_2.1304131.out


System fwd_frq_ts_FOMe-gamma-BC-pA_1_2

Reading 1 outputfiles

       Pauli        Elstat        OrbInt       Disp.       Solv.         TOTAL          Erel   Symm.      <S2>
-----------------------------------------------------------------------------------------------------------------
-----
   28448.142     -6037.870    -29348.288     -24.880     -50.401     -7013.474         0.000   NOSYM     0.752
ADF(1)

Corresponding output files
 1 : fwd_frq_ts_FOMe-gamma-BC-pA_1_2.1303891.out

Coordinates (Angs)
  48
fwd_frq_ts_FOMe-gamma-BC-pA_1_2.1303891.out        -7013.4716
C       0.000000   -0.000000    5.481069
C      -0.962110    0.191024    4.492497
C      -0.817228   -0.145349    3.140099
C       0.419260   -0.734478    2.739078
C       1.448609   -1.071325    3.780381
C       1.193865   -0.610135    5.159938
C      -1.917376    0.047228    2.209449
N      -1.773378    0.014388    0.914389
C      -2.973739    0.068987    0.072286
C      -1.902074    0.737440   -2.096017
N      -0.679989    1.010475   -1.604612
C       0.082589    1.951356   -2.178341
C      -0.348601    2.676249   -3.277433
C      -1.608691    2.407120   -3.798624
C      -2.391459    1.427732   -3.201718
Cu      0.000000    0.000000    0.000000
O       1.743731    0.000000   -0.720989
H      -3.495572   -1.841643    3.327851
O       0.735891   -1.028035    1.560472
H       1.063610    2.096387   -1.734540
H       0.298399    3.432514   -3.712533
H      -1.979236    2.954158   -4.662628
H      -3.380287    1.190371   -3.584797
H      -3.404123    1.077715    0.106763
H      -2.167440   -1.268048   -1.415769
H       2.283513   -0.478329   -0.074944
H      -3.344405    2.373619    2.412516
C       3.360102   -1.394720    5.810954
```

```
H      -6.844214    0.715101    4.253434
H       1.558199   -2.167703    3.784398
H       2.416426   -0.698969    3.419441
H      -1.894563    0.659041    4.800098
H      -0.184542    0.325355    6.500336
O       2.090161   -0.779807    6.108575
H      -3.654632   -0.446383   -1.881843
C      -2.695081   -0.305176   -1.377237
H      -3.731285   -0.613553    0.473343
H      -5.783348   -1.549182    4.239537
C      -3.273742    0.236502    2.803241
C      -3.860642    1.502267    2.807466
C      -5.132478    1.632930    3.339646
C      -5.845303    0.561453    3.853590
C      -5.244375   -0.692812    3.841439
C      -3.961011   -0.859033    3.329426
H       3.888328   -1.427116    6.762809
H       3.216351   -2.407985    5.424298
H       3.918022   -0.785847    5.092757
F      -5.701300    2.862962    3.353210

Frequencies (cm-1)
  100.00    100.00    100.00    100.00    100.00    100.00    100.00    100.00    100.00    102.17
  117.02    137.15    139.25    175.37    183.99    190.83    192.86    221.24    222.51    224.96
  240.03    248.12    265.12    302.36    307.98    319.05    326.26    345.06    360.70    408.78
  413.54    419.07    445.74    471.92    487.75    488.89    495.95    510.63    523.66    539.79
  566.29    575.59    588.18    605.85    637.68    644.28    683.89    710.53    721.62    732.93
  743.99    757.48    776.78    783.27    815.64    828.68    832.96    860.17    872.44    876.07
  877.89    889.60    918.88    943.22    954.50    965.74    968.35    970.56    991.48    992.71
  994.82   1013.34   1023.01   1058.29   1070.31   1078.51   1093.89   1106.21   1108.05   1134.37
 1147.20   1149.05   1153.86   1165.42   1169.03   1187.71   1219.99   1234.40   1243.59   1252.83
 1261.08   1291.11   1293.85   1298.74   1324.03   1333.23   1336.66   1347.95   1367.68   1383.56
 1393.76   1409.63   1412.67   1414.25   1429.20   1430.77   1435.81   1456.29   1473.26   1477.99
 1519.73   1527.67   1566.09   1585.77   1590.92   1608.81   1614.88   1619.06   2990.86   2997.35
 2999.89   3005.38   3049.53   3063.07   3093.73   3102.41   3146.77   3149.55   3151.34   3152.23
 3159.48   3160.73   3162.70   3167.76   3171.39   3177.15   3178.12   3751.09
Note: any frequencies below 100 cm-1 (including spurious imaginary ones) are upscaled to 100 cm-1
      for the calculation of thermodynamic properties.
      (Averkiev, Truhlar, Catal. Sci. Technol. 2011, 1, 1526)

Thermodynamics

Note: this script does not take into account the spin entropy
For more info, see eq. 3 of Inorg. Chem. 2002, 41, 6928-6935
(M. Reiher), https://doi.org/10.1021/ic025891l

Temperature is now: 298.150

Reporting max. of 0 frequencies (set by $GETFREQSMAX env. variable)


Reading 1 outputfiles

            ScnFrq(1)
--------------------
--------------------
(ZPVE)        229.430
(dH,0->T)      16.785
(-TS)         -50.000
(dGibbs)      196.215
====================

Corresponding output files
 1 : frq_fwd_frq_ts_FOMe-gamma-BC-pA_1_2.1304161.out


System fwd_frq_ts_FOMe-gamma-CD-pA_1_2

Reading 1 outputfiles

       Pauli        Elstat        OrbInt       Disp.       Solv.         TOTAL          Erel   Symm.      <S2>
---------------------------------------------------------------------------------------------------------------------
-----
   28617.813     -6064.853    -29516.195     -25.080     -44.612     -7033.101         0.000   NOSYM     0.752
ADF(1)

Corresponding output files
 1 : fwd_frq_ts_FOMe-gamma-CD-pA_1_2.1282194.out

Coordinates (Angs)
  48
fwd_frq_ts_FOMe-gamma-CD-pA_1_2.1282194.out        -7033.1078
C       0.000000    0.000000    4.659083
C       0.023453    0.058686    3.248106
C       0.938566   -0.818288    2.564836
C       1.760510   -1.661957    3.328959
C       1.693731   -1.690376    4.717053
C       0.796368   -0.844996    5.396114
C      -0.840745    0.994157    2.574133
H      -0.080430    3.388486    3.561382
O       1.067702   -0.875112    1.264396
Cu      0.000000    0.000000    0.000000
O       1.817183   -0.000000   -1.243866
C      -3.041794   -0.150283   -3.638754
C      -2.934491    1.302862   -0.152855
```

```
H      -2.305552    2.702966    1.336987
H      -1.245216    2.641400   -0.072354
N      -1.265268    0.105430   -1.520234
H      -4.405438    0.927324   -2.366337
C      -2.509610    0.616982   -1.416779
N      -0.943094    1.091111    1.274973
H      -3.713330    2.028602   -0.407164
H      -3.409989    0.562889    0.507379
C      -0.902328   -0.520198   -2.657938
H      -1.412136   -1.186388   -4.626145
C      -3.412119    0.499538   -2.473333
C      -1.756521   -0.671615   -3.734128
H      -0.683145    0.650647    5.195220
H       0.724696   -0.844514    6.477762
O       2.527037   -2.556944    5.331149
H       2.454136   -2.317660    2.808158
H       2.542717   -0.163072   -0.621057
H       2.036970    0.841291   -1.671352
H       1.524667   -2.947123    7.126404
H       3.252804   -3.388396    7.018499
H       2.787841   -1.673873    7.210483
C       2.508927   -2.633365    6.759346
C      -1.649473    1.918037    3.424306
C      -2.951142    1.580641    3.795321
C      -3.700744    2.441285    4.588831
C      -3.123614    3.634590    4.992578
C      -1.834833    3.999565    4.637898
C      -1.096447    3.126808    3.848097
H      -3.382727    0.636048    3.471399
H      -4.713941    2.194101    4.894155
F      -3.848336    4.478180    5.767925
H      -1.420945    4.945242    4.977523
H       0.112279   -0.906172   -2.690571
H      -3.744177   -0.242648   -4.463878
C      -1.839574    2.027033    0.615904
```

Frequencies (cm-1)

```
  100.00    100.00    100.00    100.00    100.00    100.00    100.00    100.00    100.00    100.00
  114.43    126.46    136.12    150.69    162.55    169.55    194.71    199.98    211.94    216.10
  223.02    240.34    260.36    274.43    293.13    308.68    339.65    354.38    398.78    399.56
  405.45    407.72    413.66    445.30    454.52    481.38    485.79    514.06    520.70    526.87
  576.56    587.21    591.49    610.45    625.71    638.83    645.83    673.84    682.63    731.27
  732.47    736.23    743.42    746.09    780.74    784.57    800.08    810.15    823.59    826.10
  857.56    880.75    902.88    932.97    933.67    948.21    955.42    960.34    982.59    996.40
 1002.85   1016.54   1026.25   1032.59   1067.26   1080.21   1093.15   1110.79   1120.45   1133.36
 1148.78   1151.50   1157.66   1169.87   1189.56   1201.83   1209.26   1212.86   1248.63   1260.75
 1274.55   1291.25   1299.98   1319.43   1326.36   1337.20   1349.74   1354.26   1389.33   1392.99
 1405.21   1412.07   1426.00   1426.87   1431.68   1434.60   1439.01   1479.21   1484.47   1492.37
 1516.02   1565.52   1569.08   1581.66   1602.54   1612.70   1615.12   1618.75   2977.29   2995.28
 3006.07   3072.96   3076.56   3085.17   3121.69   3145.23   3148.91   3150.74   3158.66   3164.57
 3168.72   3170.28   3170.70   3171.60   3180.91   3191.11   3711.22   3796.29
```

Note: any frequencies below 100 cm-1 (including spurious imaginary ones) are upscaled to 100 cm-1
for the calculation of thermodynamic properties.
(Averkiev, Truhlar, Catal. Sci. Technol. 2011, 1, 1526)

Thermodynamics

Note: this script does not take into account the spin entropy
For more info, see eq. 3 of Inorg. Chem. 2002, 41, 6928-6935
(M. Reiher), https://doi.org/10.1021/ic025891l

Temperature is now: 298.150

Reporting max. of 0 frequencies (set by $GETFREQSMAX env. variable)

Reading 1 outputfiles

```
            ScnFrq(1)
---------------------
---------------------
(ZPVE)        229.769
(dH,0->T)      17.100
(-TS)         -50.715
(dGibbs)      196.155
=====================
```

Corresponding output files
1 : frq_fwd_frq_ts_FOMe-gamma-CD-pA_1_2.1282788.out

System fwd_frq_ts_FOMe-gamma-mixOB-pA_1_2

Reading 1 outputfiles

```
       Pauli        Elstat        OrbInt       Disp.       Solv.         TOTAL          Erel   Symm.      <S2>
---------------------------------------------------------------------------------------------------------------
-----
   28350.794     -6043.542    -29202.890     -25.304     -49.343     -6970.453         0.000   NOSYM     0.757
ADF(1)
```

Corresponding output files
1 : fwd_frq_ts_FOMe-gamma-mixOB-pA_1_2.1286008.out

Coordinates (Angs)

```
  48
fwd_frq_ts_FOMe-ipsgamma-AF-pA_1_2.1286008.out        -6970.4616
C       0.000000    0.000000    5.139443
C       1.032752   -0.197231    4.235918
C       0.988922    0.256297    2.920803
C      -0.152507    1.125225    2.523947
C      -1.276913    1.181110    3.470900
C      -1.171579    0.687140    4.760646
C       2.041185   -0.074012    1.990063
N       1.844535    0.000000    0.699559
C       2.954407   -0.231286   -0.221069
C       1.604675   -1.488384   -1.897348
N       0.432131   -1.481767   -1.236907
C      -0.430552   -2.499173   -1.370142
C      -0.144378   -3.596184   -2.165499
C       1.067929   -3.629129   -2.844684
C       1.947057   -2.562429   -2.712666
Cu      0.000000    0.000000    0.000000
O      -1.762046    0.032844   -0.576345
H       3.016318   -2.605699    2.145839
O      -0.392938    1.413385    1.230963
H      -1.365224   -2.402612   -0.825070
H      -0.864053   -4.405771   -2.243208
H       1.327098   -4.477366   -3.473973
H       2.899443   -2.551725   -3.235577
H       3.940318    1.488001    3.099819
H       6.128522    0.765808    4.053309
H      -2.175335    0.703174   -0.010453
H      -1.415890    0.739033    7.480628
H      -3.185873    0.554049    7.307476
H      -2.105599   -0.786007    6.823442
H       0.303834    2.083367    2.976274
H      -2.161408    1.729651    3.155655
H       1.895848   -0.769270    4.564157
H       0.104881   -0.398001    6.142903
O      -2.232481    0.877649    5.560214
F       6.934618   -1.697193    4.157223
C       2.501272   -0.315755   -1.670439
H       5.192755   -3.319879    3.128558
C      -2.217489    0.303166    6.873573
C       3.346957   -0.511076    2.549663
C       4.216033    0.436068    3.093681
C       5.436076    0.043264    3.629946
C       5.752984   -1.305544    3.624708
C       4.907569   -2.271741    3.101425
C       3.698087   -1.863135    2.555031
H       3.505408   -1.140761    0.042862
H       3.652773    0.608567   -0.112107
H       3.388069   -0.396816   -2.303785
H       1.980581    0.608188   -1.955294

Frequencies (cm-1)
  100.00    100.00    100.00    100.00    100.00    100.00    100.00    100.00    100.00    124.23
  129.23    153.14    162.63    165.93    182.01    194.29    208.03    218.73    230.78    252.59
  255.77    294.59    300.92    314.01    331.98    337.58    362.99    373.24    378.95    398.23
  404.39    413.83    424.40    437.82    481.85    487.05    503.67    517.78    535.71    539.70
  566.77    573.18    581.48    606.38    612.08    647.48    673.34    690.27    702.87    736.90
  744.92    756.91    758.99    778.92    799.13    805.85    817.71    827.69    836.18    855.07
  860.53    876.09    886.24    922.11    934.95    948.37    949.61    952.69    961.06    996.02
  996.68   1002.26   1015.22   1019.33   1032.00   1057.72   1070.17   1092.94   1106.66   1116.58
 1120.23   1146.55   1149.08   1156.16   1165.52   1171.69   1197.79   1204.77   1218.44   1223.84
 1228.08   1274.40   1280.51   1289.25   1293.58   1320.93   1326.62   1342.55   1347.46   1354.99
 1361.16   1398.75   1407.03   1410.67   1416.53   1429.17   1435.37   1436.90   1447.36   1478.47
 1488.81   1515.35   1537.31   1575.75   1584.98   1600.97   1611.98   1615.67   2555.79   2980.68
 3000.20   3009.87   3062.63   3078.45   3102.65   3133.78   3144.55   3149.48   3152.79   3163.73
 3165.40   3165.71   3170.39   3172.73   3173.61   3179.89   3190.69   3730.45
Note: any frequencies below 100 cm-1 (including spurious imaginary ones) are upscaled to 100 cm-1
      for the calculation of thermodynamic properties.
      (Averkiev, Truhlar, Catal. Sci. Technol. 2011, 1, 1526)

Thermodynamics

Note: this script does not take into account the spin entropy
For more info, see eq. 3 of Inorg. Chem. 2002, 41, 6928-6935
(M. Reiher), https://doi.org/10.1021/ic025891l

Temperature is now: 298.150

Reporting max. of 0 frequencies (set by $GETFREQSMAX env. variable)


Reading 1 outputfiles

            ScnFrq(1)
---------------------
---------------------
(ZPVE)        227.636
(dH,0->T)      16.660
(-TS)         -49.561
(dGibbs)      194.735
=====================

Corresponding output files
 1 : frq_fwd_frq_ts_FOMe-gamma-mixOB-pA_1_2.1286296.out
```

```
System fwd_frq_ts_FOMe-ipso-AO-pA_1_2

Reading 1 outputfiles

       Pauli        Elstat        OrbInt       Disp.       Solv.         TOTAL          Erel   Symm.      <S2>
----------------------------------------------------------------------------------------------------------------------
-----
   28167.447     -5970.580    -29074.046     -27.275     -48.509     -6953.132         0.000   NOSYM     0.760
ADF(1)

Corresponding output files
 1 : fwd_frq_ts_FOMe-ipso-AO-pA_1_2.1282199.out

Coordinates (Angs)
  48
rev_frq_ts_FOMe-ipso-AF-pA_1_2.1282199.out        -6953.0980
C       0.000000    0.000000    3.874912
C       0.233673    0.838975    2.802502
C      -0.788779    1.633649    2.262379
C      -2.060293    1.607107    2.862556
C      -2.313003    0.752365    3.911957
C      -1.289910   -0.068374    4.423532
C      -0.480344    2.504171    1.115096
N       0.045374    1.933758    0.071693
C       0.670150    2.630290   -1.044007
C       2.520009    0.905440   -0.964557
N       1.931483    0.000000   -0.161327
C       2.654906   -0.811408    0.623044
C       4.035398   -0.729928    0.658979
C       4.667734    0.213950   -0.141992
C       3.904618    1.031152   -0.965093
Cu      0.000000    0.000000    0.000000
O      -0.053811   -1.815621   -0.059277
H      -1.486927    4.243924   -0.768648
O      -1.747636   -0.004257    0.207390
H       2.094549   -1.527064    1.218289
H       4.593507   -1.394647    1.311156
H       5.750514    0.310341   -0.128821
H       4.369698    1.765920   -1.615838
H      -0.121139    3.978505    3.315172
H      -0.549951    6.409340    3.602174
H      -0.949122   -2.022042    0.259895
H       0.176178   -1.153957    6.449151
H      -1.158395   -2.294835    6.787976
H      -0.250500   -2.425416    5.252977
H      -2.854318    2.238442    2.471670
H      -3.302516    0.693966    4.358488
H       1.241402    0.920345    2.403590
H       0.817368   -0.582917    4.285395
O      -1.633918   -0.868516    5.442139
F      -1.487173    7.951343    1.742390
C       1.609531    1.712282   -1.827662
H      -1.932449    6.654501   -0.455274
C      -0.642060   -1.734390    6.008181
C      -0.735464    3.942044    1.242004
C      -0.505234    4.561081    2.482260
C      -0.741062    5.914417    2.654081
C      -1.243444    6.635757    1.580458
C      -1.515506    6.055461    0.349445
C      -1.248002    4.707077    0.182976
H       1.213179    3.501057   -0.658253
H      -0.095019    2.982600   -1.741928
H       2.189116    2.328867   -2.518397
H       1.013228    1.021769   -2.438800

Frequencies (cm-1)
   100.00    100.00    100.00    100.00    100.00    100.00    100.00    100.00    100.00    100.00
   111.04    135.02    148.12    152.29    170.02    202.83    208.23    212.37    216.54    238.31
   257.52    268.54    285.03    302.49    319.55    338.61    367.28    393.17    396.57    401.18
   406.69    412.00    420.84    435.50    483.04    492.37    500.85    508.84    548.72    566.65
   593.47    594.41    609.62    612.73    622.82    656.70    678.01    698.84    731.04    744.71
   745.34    756.20    783.59    789.91    793.70    794.82    823.76    826.48    830.97    860.72
   876.07    891.94    919.75    944.73    947.70    951.93    955.88    958.54    977.53    995.46
   995.48   1000.25   1014.63   1024.75   1028.75   1053.91   1066.35   1102.75   1113.42   1114.87
  1119.31   1147.39   1151.64   1161.37   1167.56   1184.08   1192.33   1212.56   1221.61   1234.78
  1276.78   1279.84   1285.46   1294.38   1309.93   1322.84   1331.43   1349.58   1357.21   1368.83
  1411.15   1412.03   1419.50   1421.36   1428.25   1434.06   1439.26   1448.94   1480.92   1491.24
  1500.38   1550.37   1565.87   1584.64   1592.22   1606.67   1613.91   1616.73   2981.75   3008.78
  3010.15   3079.35   3080.76   3106.52   3133.97   3153.80   3156.86   3157.85   3160.40   3165.36
  3168.32   3169.23   3175.18   3177.20   3182.77   3184.56   3186.57   3693.52
Note: any frequencies below 100 cm-1 (including spurious imaginary ones) are upscaled to 100 cm-1
      for the calculation of thermodynamic properties.
      (Averkiev, Truhlar, Catal. Sci. Technol. 2011, 1, 1526)

Thermodynamics

Note: this script does not take into account the spin entropy
For more info, see eq. 3 of Inorg. Chem. 2002, 41, 6928-6935
(M. Reiher), https://doi.org/10.1021/ic025891l

Temperature is now: 298.150

Reporting max. of 0 frequencies (set by $GETFREQSMAX env. variable)
```

```
Reading 1 outputfiles

            ScnFrq(1)
---------------------
---------------------
(ZPVE)        229.449
(dH,0->T)      16.729
(-TS)         -49.922
(dGibbs)      196.256
=====================

Corresponding output files
 1 : frq_fwd_frq_ts_FOMe-ipso-AO-pA_1_2.1284186.out


System fwd_frq_ts_FOMe-ipso-FG-pA_1_2

Reading 1 outputfiles

       Pauli        Elstat        OrbInt       Disp.       Solv.         TOTAL          Erel   Symm.      <S2>
----------------------------------------------------------------------------------------------------------------------
-----
   28376.924     -6020.717    -29277.694     -26.521     -50.139     -6998.319         0.000   NOSYM     0.753
ADF(1)

Corresponding output files
 1 : fwd_frq_ts_FOMe-ipso-FG-pA_1_2.1282761.out

Coordinates (Angs)
  48
rev_frq_ts_FOMe-ipso-FG-pA_1_2.1282761.out        -6998.3223
C       0.000000    0.000000    4.483032
C      -0.049455   -0.175656    3.013038
C       0.630996   -1.410975    2.448186
C       1.070991   -2.417465    3.361365
C       1.001074   -2.200468    4.699530
C       0.490123   -0.979643    5.276015
C      -1.472665   -0.209042    2.445203
N      -1.611967   -0.040618    1.183464
C      -2.944664   -0.032687    0.573747
C      -2.161460    1.477856   -1.288902
N      -0.834264    1.501067   -1.063601
C      -0.112452    2.585842   -1.372824
C      -0.693866    3.721264   -1.914167
C      -2.065967    3.723612   -2.135895
C      -2.804719    2.589282   -1.825001
Cu      0.000000    0.000000    0.000000
O       1.567251    0.000000   -1.007704
H      -2.550715   -2.631010    2.748824
O       0.758394   -1.531306    1.211139
H       0.954769    2.517116   -1.177377
H      -0.076735    4.582914   -2.151475
H      -2.558041    4.600099   -2.551094
H      -3.877108    2.554688   -1.997536
H      -2.847441    1.429622    4.159965
H      -4.667656    0.744972    5.716271
H       2.130472   -0.701831   -0.649507
H      -1.028060    0.319175    7.051401
H       0.167613    0.011205    8.349117
H       0.588387    1.097494    6.989996
H       1.489899   -3.332698    2.951680
H       1.356457   -2.962248    5.391686
H       0.453866    0.681694    2.537944
H      -0.389147    0.928982    4.882316
O       0.564934   -0.958290    6.623207
F      -5.569626   -1.675809    5.939157
C      -2.879828    0.216717   -0.927814
H      -4.359927   -3.303056    4.320929
C       0.038028    0.194249    7.281951
C      -2.582857   -0.556873    3.356107
C      -3.174210    0.393144    4.190353
C      -4.190654    0.021334    5.060969
C      -4.586975   -1.306816    5.085076
C      -4.018796   -2.272625    4.269661
C      -3.011593   -1.886881    3.395085
H      -3.546648    0.742393    1.062856
H      -3.440541   -0.992424    0.755304
H      -3.903977    0.247958   -1.308797
H      -2.380546   -0.629981   -1.417387

Frequencies (cm-1)
   100.00    100.00    100.00    100.00    100.00    100.00    100.00    100.00    100.00    116.38
   123.86    131.94    144.89    169.74    175.41    186.18    191.02    203.59    217.62    228.46
   248.00    263.08    294.82    314.93    318.08    336.74    360.70    372.12    395.96    396.31
   404.30    405.17    418.68    463.31    485.65    490.97    509.68    511.11    522.54    532.61
   579.01    604.78    605.40    628.32    641.08    665.38    679.73    720.61    742.43    753.47
   759.85    769.36    776.28    785.23    798.78    803.46    821.17    825.63    827.11    845.81
   870.74    880.34    933.05    940.66    948.29    949.77    960.22    978.28    993.99    998.44
  1002.81   1013.45   1020.96   1048.09   1055.48   1065.36   1080.66   1095.05   1106.97   1115.09
  1125.13   1145.49   1148.67   1148.87   1168.02   1176.10   1207.97   1218.45   1234.67   1235.12
  1244.43   1270.37   1276.64   1289.53   1292.53   1323.03   1325.78   1331.30   1346.24   1354.07
  1402.83   1402.93   1406.96   1418.98   1424.73   1431.07   1435.70   1436.26   1475.94   1491.02
  1541.62   1584.68   1587.03   1599.41   1611.74   1613.93   1643.52   1654.87   2968.20   2979.23
  3008.24   3010.47   3059.13   3073.76   3097.74   3130.43   3143.75   3146.36   3151.52   3151.63
```

```
 3158.32   3167.96   3170.66   3172.83   3173.46   3177.83   3192.79   3751.35
Note: any frequencies below 100 cm-1 (including spurious imaginary ones) are upscaled to 100 cm-1
      for the calculation of thermodynamic properties.
      (Averkiev, Truhlar, Catal. Sci. Technol. 2011, 1, 1526)

Thermodynamics

Note: this script does not take into account the spin entropy
For more info, see eq. 3 of Inorg. Chem. 2002, 41, 6928-6935
(M. Reiher), https://doi.org/10.1021/ic025891l

Temperature is now: 298.150

Reporting max. of 0 frequencies (set by $GETFREQSMAX env. variable)


Reading 1 outputfiles

            ScnFrq(1)
---------------------
---------------------
(ZPVE)        229.354
(dH,0->T)      16.726
(-TS)         -49.852
(dGibbs)      196.229
=====================

Corresponding output files
 1 : frq_fwd_frq_ts_FOMe-ipso-FG-pA_1_2.1284188.out


System fwd_frq_ts_FOMe-ipso-GH-pA_1_2

Reading 1 outputfiles

       Pauli        Elstat        OrbInt       Disp.       Solv.         TOTAL          Erel   Symm.      <S2>
---------------------------------------------------------------------------------------------------------------------
-----
   28590.041     -6062.330    -29487.645     -25.393     -45.275     -7030.775         0.000   NOSYM     0.752
ADF(1)

Corresponding output files
 1 : fwd_frq_ts_FOMe-ipso-GH-pA_1_2.1282767.out

Coordinates (Angs)
  48
fwd_frq_ts_FOMe-ipso-GH-pA_1_2.1282767.out        -7030.7700
C       0.000000    0.000000    4.658200
C       0.020805    0.078222    3.242872
C      -1.211332    0.304539    2.552449
C      -2.381301    0.501485    3.330829
C      -2.367794    0.413003    4.700156
C      -1.163219    0.150100    5.381783
C       1.300927   -0.029416    2.579258
N       1.474236   -0.000000    1.281735
C       2.830832   -0.108586    0.737739
C       2.238555   -0.869505   -1.587610
N       0.907590   -1.000784   -1.428941
C       0.234311   -1.927762   -2.130111
C       0.867043   -2.779448   -3.019227
C       2.242601   -2.663820   -3.184691
C       2.931964   -1.697649   -2.464683
Cu      0.000000    0.000000    0.000000
O      -1.336981    0.929075   -1.429881
H       2.685416    1.972851    3.754847
O      -1.346601    0.340723    1.255358
H      -0.838919   -1.977490   -1.962423
H       0.286823   -3.518241   -3.564039
H       2.774278   -3.320411   -3.869476
H       4.006360   -1.578237   -2.574088
H       2.560992   -2.307969    3.357338
H       4.552008   -2.498555    4.846384
H      -0.922782    1.444609   -2.137768
H       3.233050   -1.110040    0.939124
H       3.482222    0.605288    1.253375
H       3.960321    0.245649   -1.033144
H      -3.308470    0.694546    2.796040
H      -3.283991    0.543329    5.273355
H      -1.911716    1.553621   -0.960887
H       0.932868   -0.177492    5.175799
H      -0.336761   -0.192428    8.516746
F       5.782247   -0.477825    5.911570
C       2.904962    0.177114   -0.756426
H       4.684745    1.773732    5.232997
H       2.452416    1.156083   -0.965882
C       2.504470   -0.153939    3.453826
C       3.028226   -1.410783    3.757537
C       4.135793   -1.528112    4.589469
C       4.703551   -0.370703    5.097177
C       4.210799    0.892124    4.809715
C       3.099290    0.992908    3.982115
O      -1.249618    0.077384    6.738956
H       0.390670   -1.140107    7.187626
C      -0.049374   -0.172508    7.464139
H       0.687689    0.625824    7.302686
```

```
Frequencies (cm-1)
  100.00    100.00    100.00    100.00    100.00    100.00    100.00    100.00    100.00    106.54
  110.59    112.74    131.21    144.52    154.88    165.83    184.23    188.42    199.40    239.06
  244.24    253.32    261.13    280.55    319.44    328.55    352.12    375.92    387.21    391.65
  398.35    403.06    408.75    432.20    449.09    454.11    490.99    499.23    525.83    530.21
  537.06    543.00    577.09    610.29    614.86    648.31    650.65    682.03    693.09    737.13
  742.99    750.48    757.88    783.21    787.16    801.48    813.11    815.23    827.79    834.57
  852.06    875.42    887.99    933.14    939.47    945.98    949.67    955.32    979.10    995.55
 1003.40   1021.57   1027.34   1038.96   1060.86   1074.77   1092.38   1106.17   1111.65   1125.43
 1143.33   1150.35   1155.04   1169.83   1171.86   1202.90   1221.60   1222.18   1238.92   1256.73
 1273.85   1274.97   1298.30   1325.02   1327.52   1332.49   1344.44   1353.52   1389.98   1401.64
 1405.12   1412.37   1414.77   1419.81   1426.28   1438.04   1441.46   1464.92   1483.12   1491.10
 1514.46   1563.79   1565.70   1585.16   1601.96   1611.99   1618.14   1620.53   2958.20   2996.19
 3005.35   3044.73   3062.46   3096.15   3118.53   3142.38   3144.99   3146.48   3150.67   3155.79
 3159.47   3170.32   3170.52   3171.17   3179.52   3211.79   3714.20   3801.35
Note: any frequencies below 100 cm-1 (including spurious imaginary ones) are upscaled to 100 cm-1
      for the calculation of thermodynamic properties.
      (Averkiev, Truhlar, Catal. Sci. Technol. 2011, 1, 1526)

Thermodynamics

Note: this script does not take into account the spin entropy
For more info, see eq. 3 of Inorg. Chem. 2002, 41, 6928-6935
(M. Reiher), https://doi.org/10.1021/ic025891l

Temperature is now: 298.150

Reporting max. of 0 frequencies (set by $GETFREQSMAX env. variable)


Reading 1 outputfiles

            ScnFrq(1)
---------------------
---------------------
(ZPVE)        229.593
(dH,0->T)      17.103
(-TS)         -50.685
(dGibbs)      196.011
=====================

Corresponding output files
 1 : frq_fwd_frq_ts_FOMe-ipso-GH-pA_1_2.1283714.out


System fwd_frq_ts_FOMe-ipso-OF-pA_1_2

Reading 1 outputfiles

       Pauli        Elstat        OrbInt       Disp.       Solv.         TOTAL          Erel   Symm.      <S2>
-----------------------------------------------------------------------------------------------------------------
-----
   28259.349     -5998.853    -29160.127     -25.686     -51.322     -6976.804         0.000   NOSYM     0.755
ADF(1)

Corresponding output files
 1 : fwd_frq_ts_FOMe-ipso-OF-pA_1_2.1286010.out

Coordinates (Angs)
  48
fwd_frq_ts_FOMe-ipso-preFF-pA_1_2.1286010.out        -6976.7771
C       0.000000    0.000000    4.671745
C      -0.368173    0.118681    3.375158
C      -0.635623   -1.049436    2.475925
C      -0.571411   -2.356487    3.188533
C      -0.206004   -2.464600    4.485368
C       0.080350   -1.296621    5.252696
C      -1.982772   -0.812471    1.769475
N      -1.866098   -0.215812    0.641542
C      -3.006791    0.209621   -0.158920
C      -1.706988    1.353484   -1.986971
N      -0.477341    1.313788   -1.436317
C       0.411302    2.280726   -1.714730
C       0.108831    3.345570   -2.546362
C      -1.159448    3.409920   -3.109273
C      -2.070797    2.401484   -2.828979
Cu      0.000000    0.000000    0.000000
O       1.803205    0.000000   -0.488777
H      -3.659280   -2.693662    0.823423
O       0.402299   -1.063693    1.526891
H       1.385498    2.168458   -1.247137
H       0.858632    4.107583   -2.738352
H      -1.437746    4.235664   -3.760004
H      -3.069338    2.416578   -3.257092
H      -3.056685    0.004522    4.130807
H      -5.107697   -0.885857    5.217308
H       2.241374   -0.503990    0.213198
H      -0.056678    0.252812    7.481525
H       1.038905   -0.892592    8.317300
H       1.665233    0.108759    6.970041
H      -0.759797   -3.243014    2.586775
H      -0.095129   -3.431654    4.969053
H      -0.402556    1.096242    2.897400
H       0.270420    0.879503    5.246114
```

```
O       0.446353   -1.518612    6.490785
F      -6.606297   -2.748208    4.213784
C      -2.663405    0.256902   -1.642432
H      -5.704734   -3.576120    1.926512
C       0.795326   -0.422419    7.365758
C      -3.228982   -1.272458    2.395832
C      -3.629966   -0.778440    3.641199
C      -4.774593   -1.267137    4.256005
C      -5.494281   -2.263748    3.615645
C      -5.117451   -2.787054    2.388364
C      -3.979063   -2.281634    1.777785
H      -3.591458    0.399892   -2.201737
H      -2.246948   -0.711474   -1.949329
H      -3.290501    1.210999    0.191960
H      -3.867035   -0.445438   -0.004075

Frequencies (cm-1)
  100.00    100.00    100.00    100.00    100.00    100.00    100.00    100.00    100.00    110.44
  136.34    141.10    149.82    167.71    177.51    187.44    191.21    206.25    217.31    231.93
  252.07    266.45    291.42    295.05    314.24    341.44    357.60    375.85    397.85    400.61
  404.68    409.95    420.63    449.85    468.69    487.15    503.06    508.73    519.13    548.81
  573.90    575.64    586.35    615.87    642.27    676.12    689.30    720.29    730.45    744.71
  755.40    758.58    765.38    782.63    799.50    813.13    816.22    825.27    847.72    852.89
  878.86    913.83    934.98    939.62    947.71    950.91    959.17    959.49    969.94    979.50
  995.66   1000.09   1005.74   1014.43   1026.38   1059.43   1062.98   1075.90   1096.89   1101.94
 1108.03   1111.20   1149.17   1149.57   1158.93   1166.04   1168.87   1211.12   1220.76   1235.67
 1241.27   1260.22   1280.15   1294.80   1304.45   1315.15   1318.52   1327.02   1343.72   1355.12
 1392.23   1409.83   1410.39   1413.83   1425.41   1429.47   1433.59   1434.65   1476.61   1478.19
 1491.65   1538.15   1583.98   1596.25   1611.15   1614.87   1625.77   1654.45   3004.50   3005.57
 3013.89   3092.39   3098.83   3115.70   3143.02   3147.83   3151.16   3151.18   3155.41   3155.78
 3162.38   3169.17   3169.67   3173.06   3174.95   3177.72   3184.58   3744.53
Note: any frequencies below 100 cm-1 (including spurious imaginary ones) are upscaled to 100 cm-1
      for the calculation of thermodynamic properties.
      (Averkiev, Truhlar, Catal. Sci. Technol. 2011, 1, 1526)

Thermodynamics

Note: this script does not take into account the spin entropy
For more info, see eq. 3 of Inorg. Chem. 2002, 41, 6928-6935
(M. Reiher), https://doi.org/10.1021/ic025891l

Temperature is now: 298.150

Reporting max. of 0 frequencies (set by $GETFREQSMAX env. variable)


Reading 1 outputfiles

            ScnFrq(1)
---------------------
---------------------
(ZPVE)        229.027
(dH,0->T)      16.759
(-TS)         -49.901
(dGibbs)      195.885
=====================

Corresponding output files
 1 : frq_fwd_frq_ts_FOMe-ipso-OF-pA_1_2.1286217.out


System rev_frq_ts_FOMe-gamma-AB-pA_1_2

Reading 1 outputfiles

       Pauli        Elstat        OrbInt       Disp.       Solv.         TOTAL          Erel   Symm.      <S2>
--------------------------------------------------------------------------------------------------------------------
-----
   28226.542     -5971.917    -29141.911     -25.940     -43.015     -6956.407         0.000   NOSYM     0.752
ADF(1)

Corresponding output files
 1 : rev_frq_ts_FOMe-gamma-AB-pA_1_2.1282192.out

Coordinates (Angs)
  48
fwd_frq_ts_FOMe-gamma-AB-pA_1_2.1282192.out        -6956.4096
C       0.000000    0.000000    4.620102
C      -0.990873    0.397579    3.738403
C      -1.252275   -0.325490    2.567655
C      -0.520462   -1.508480    2.330616
C       0.456511   -1.923046    3.210298
C       0.739558   -1.161953    4.357181
C      -2.226828    0.159150    1.584494
N      -1.928005   -0.006531    0.328714
C      -2.842696    0.291870   -0.761388
C      -1.243693    2.076743   -1.584900
N      -0.144424    1.814823   -0.846371
C       0.690171    2.804198   -0.501232
C       0.458970    4.121191   -0.863761
C      -0.681001    4.413927   -1.602746
C      -1.536136    3.381651   -1.968886
Cu      0.000000    0.000000    0.000000
O       2.673887    0.000000   -0.774011
H      -4.097450   -0.789826    3.292462
```

```
O       1.763719   -0.420263    0.223980
H       1.561743    2.516503    0.082084
H       1.160070    4.894583   -0.564431
H      -0.900915    5.437275   -1.897316
H      -2.428076    3.576776   -2.558199
H      -3.664980    0.938528   -0.445916
H      -1.461116    0.140806   -2.411855
H       3.344751    0.469145   -0.244775
H       1.195387   -0.875480    7.021593
H       2.862767   -1.481685    6.800031
H       2.395130    0.097338    6.104068
H      -0.768408   -2.133624    1.474083
H       1.004987   -2.846177    3.039580
H      -1.550140    1.306181    3.946579
H       0.195577    0.597512    5.504145
O       1.720925   -1.634269    5.145136
H      -2.838801    1.201123   -2.702525
C      -2.100921    0.905811   -1.952155
H      -3.280519   -0.660012   -1.089112
C       2.051678   -0.916950    6.338571
C      -3.422777    0.865065    2.085482
C      -3.646840    2.202018    1.733647
C      -4.710689    2.906958    2.277142
C      -5.552088    2.247760    3.160968
C      -5.362389    0.925188    3.531482
C      -4.278598    0.241333    2.998633
H      -2.964023    2.709100    1.054759
H      -4.885509    3.951031    2.032210
F      -6.593196    2.927850    3.692747
H      -6.043904    0.452644    4.233597

Frequencies (cm-1)
   100.00    100.00    100.00    100.00    100.00    100.00    100.00    100.00    100.00    100.00
   100.00    111.17    134.70    139.34    155.76    166.88    184.90    198.43    207.95    219.38
   239.82    255.97    259.06    290.25    292.81    315.50    345.78    366.70    396.49    405.16
   407.52    410.44    415.39    422.66    481.99    489.95    502.05    506.60    515.57    550.65
   583.93    594.47    613.92    621.58    641.93    675.24    695.39    731.63    742.06    747.94
   760.53    776.31    788.65    799.22    800.12    821.29    826.33    829.81    864.23    879.58
   899.91    921.85    941.19    942.71    950.96    953.38    956.87    960.84    993.10    994.83
  1003.03   1012.10   1021.40   1031.31   1055.85   1064.58   1096.10   1108.14   1115.03   1122.48
  1146.14   1149.26   1160.08   1165.75   1182.89   1190.38   1204.69   1210.78   1225.36   1273.74
  1276.20   1285.49   1294.61   1310.36   1316.98   1326.58   1340.24   1341.74   1359.72   1362.56
  1410.76   1414.62   1416.55   1426.94   1428.38   1433.58   1435.57   1444.06   1474.61   1490.64
  1502.75   1558.63   1575.64   1581.28   1597.96   1609.78   1613.02   1616.44   2979.24   3004.18
  3009.37   3075.44   3086.15   3100.91   3128.94   3131.57   3142.33   3150.81   3151.92   3155.52
  3157.41   3162.45   3168.33   3172.17   3173.92   3179.34   3185.78   3648.26
Note: any frequencies below 100 cm-1 (including spurious imaginary ones) are upscaled to 100 cm-1
      for the calculation of thermodynamic properties.
      (Averkiev, Truhlar, Catal. Sci. Technol. 2011, 1, 1526)

Thermodynamics

Note: this script does not take into account the spin entropy
For more info, see eq. 3 of Inorg. Chem. 2002, 41, 6928-6935
(M. Reiher), https://doi.org/10.1021/ic025891l

Temperature is now: 298.150

Reporting max. of 0 frequencies (set by $GETFREQSMAX env. variable)


Reading 1 outputfiles

            ScnFrq(1)
--------------------
--------------------
(ZPVE)        229.514
(dH,0->T)      16.992
(-TS)         -50.749
(dGibbs)      195.757
====================

Corresponding output files
 1 : frq_rev_frq_ts_FOMe-gamma-AB-pA_1_2.1282995.out


System rev_frq_ts_FOMe-gamma-BC-pA_1_2

Reading 1 outputfiles

       Pauli        Elstat        OrbInt       Disp.       Solv.         TOTAL          Erel   Symm.      <S2>
------------------------------------------------------------------------------------------------------------------
-----
   28325.143     -6041.827    -29182.536     -25.468     -46.248     -6971.101         0.000   NOSYM     0.758
ADF(1)

Corresponding output files
 1 : rev_frq_ts_FOMe-gamma-BC-pA_1_2.1304062.out

Coordinates (Angs)
  48
rev_frq_ts_FOMe-gamma-BC-pA_1_2.1304062.out        -6971.1123
C       0.000000    0.000000    5.053367
C      -1.063846    0.167243    4.188562
C      -1.029376   -0.292774    2.870851
```

```
C       0.099231   -1.189258    2.476978
C       1.278267   -1.171186    3.375797
C       1.198780   -0.637771    4.640417
C      -2.062233    0.056301    1.934934
N      -1.846834   -0.004861    0.643429
C      -2.926003    0.238452   -0.305849
C      -1.516431    1.432378   -1.978372
N      -0.388326    1.466121   -1.245057
C       0.481562    2.479100   -1.367422
C       0.249481    3.530545   -2.237437
C      -0.909704    3.517842   -3.004127
C      -1.797815    2.458414   -2.874356
Cu      0.000000    0.000000    0.000000
O       1.771960    0.000000   -0.505832
H      -3.997334   -1.490969    3.002549
O       0.337939   -1.446629    1.160159
H       1.376335    2.416106   -0.755169
H       0.971923    4.338361   -2.306195
H      -1.121613    4.327123   -3.698871
H      -2.710666    2.414502   -3.461779
H      -3.453757    1.170994   -0.074757
H      -1.914958   -0.664002   -1.985804
H       2.165972   -0.694994    0.045102
H      -3.029068    2.611957    2.069142
C       3.439055   -1.218719    5.178802
H      -6.806698    1.666575    3.865850
H      -0.373310   -2.149524    2.867605
H       2.152517   -1.701729    3.012417
H      -1.927786    0.730181    4.531238
H      -0.050587    0.396861    6.064309
O       2.187860   -0.631447    5.551986
H      -3.297512    0.346209   -2.406214
C      -2.431596    0.274718   -1.743949
H      -3.652004   -0.577349   -0.195522
H      -6.198630   -0.757829    3.880819
C      -3.378409    0.503018    2.469278
C      -3.711878    1.858644    2.454271
C      -4.942704    2.234722    2.965587
C      -5.851876    1.321820    3.477632
C      -5.502362   -0.024361    3.481520
C      -4.268791   -0.438368    2.987294
H       4.088972   -1.101681    6.046504
H       3.317959   -2.283778    4.947175
H       3.872652   -0.695880    4.317672
F      -5.264898    3.549311    2.965999

Frequencies (cm-1)
   100.00    100.00    100.00    100.00    100.00    100.00    100.00    100.00    116.09    124.01
   128.43    153.67    179.41    188.05    196.21    208.16    221.74    228.42    231.97    241.88
   258.23    266.73    296.59    320.52    326.82    363.72    369.74    384.01    400.97    413.20
   418.67    422.31    443.43    469.81    491.11    509.41    511.98    522.18    533.98    548.84
   558.86    568.47    589.49    599.08    640.58    651.50    680.34    692.91    696.88    723.14
   744.30    753.35    774.84    777.77    787.23    816.74    832.80    863.29    870.70    872.74
   876.28    889.57    892.24    927.07    938.07    954.25    954.50    963.79    967.38    991.27
   995.10   1016.96   1021.47   1032.54   1044.03   1058.48   1068.64   1079.24   1098.68   1110.01
  1123.77   1140.51   1149.39   1152.53   1156.20   1169.17   1176.75   1186.74   1218.96   1225.98
  1235.24   1253.68   1259.68   1282.97   1293.26   1319.68   1325.97   1340.64   1344.03   1357.09
  1367.48   1397.63   1409.95   1415.06   1428.02   1428.36   1436.06   1437.22   1448.60   1472.63
  1478.48   1502.55   1521.51   1571.40   1584.74   1590.47   1615.38   1617.86   2647.00   2974.87
  2999.39   3008.45   3060.23   3069.46   3103.94   3132.08   3148.32   3154.47   3157.64   3159.94
  3161.42   3168.41   3170.64   3172.45   3172.46   3182.28   3183.10   3721.41
Note: any frequencies below 100 cm-1 (including spurious imaginary ones) are upscaled to 100 cm-1
      for the calculation of thermodynamic properties.
      (Averkiev, Truhlar, Catal. Sci. Technol. 2011, 1, 1526)

Thermodynamics

Note: this script does not take into account the spin entropy
For more info, see eq. 3 of Inorg. Chem. 2002, 41, 6928-6935
(M. Reiher), https://doi.org/10.1021/ic025891l

Temperature is now: 298.150

Reporting max. of 0 frequencies (set by $GETFREQSMAX env. variable)


Reading 1 outputfiles

            ScnFrq(1)
---------------------
---------------------
(ZPVE)        228.229
(dH,0->T)      16.438
(-TS)         -49.002
(dGibbs)      195.666
=====================

Corresponding output files
 1 : frq_rev_frq_ts_FOMe-gamma-BC-pA_1_2.1304160.out


System rev_frq_ts_FOMe-gamma-CD-pA_1_2

Reading 1 outputfiles
```

```
       Pauli        Elstat        OrbInt       Disp.       Solv.         TOTAL          Erel   Symm.      <S2>
-----------------------------------------------------------------------------------------------------------------------
-----
   28462.344     -6033.717    -29369.234     -24.892     -48.679     -7014.354         0.000   NOSYM     0.752
ADF(1)

Corresponding output files
 1 : rev_frq_ts_FOMe-gamma-CD-pA_1_2.1282195.out

Coordinates (Angs)
  48
rev_frq_ts_FOMe-gamma-CD-pA_1_2.1282195.out        -7014.3502
C       0.000000    0.000000    4.618019
C      -0.114037    0.282901    3.256854
C       1.027873    0.842638    2.598257
C       2.218388    1.244661    3.424251
C       2.222599    0.845403    4.852316
C       1.127362    0.231559    5.411495
C      -1.380912    0.082164    2.570102
H      -2.613118    2.049797    3.953965
O       1.110768    1.058552    1.367542
Cu      0.000000    0.000000    0.000000
O       1.538940   -0.000000   -1.070277
C      -2.248135   -2.627701   -3.459097
C      -3.053394   -1.356215    0.001282
H      -3.591681    0.219269    1.354628
H      -2.818062    0.778694   -0.131767
N      -1.090478   -1.075023   -1.459449
H      -3.901898   -2.800932   -2.088132
C      -2.315480   -1.607056   -1.277020
N      -1.484843    0.054869    1.272966
H      -4.125984   -1.446288   -0.197110
H      -2.807468   -2.151764    0.718087
C      -0.452490   -1.298514   -2.622303
H      -0.429861   -2.207636   -4.560841
C      -2.911489   -2.389991   -2.266755
C      -0.991990   -2.063098   -3.642520
H      -0.855428   -0.453852    5.112121
H       1.107758   -0.061974    6.454384
O       3.343132    1.151083    5.468769
H       2.299818    2.341295    3.364889
H       3.124006    0.871712    2.928563
H       2.196484    0.505773   -0.571652
H       3.414693   -0.249836    7.015276
H       4.482390    1.184922    7.137628
H       2.726181    1.354648    7.454120
C       3.487742    0.831828    6.867830
C      -2.582222   -0.044859    3.442939
C      -3.185369   -1.288832    3.639787
C      -4.282852   -1.413529    4.482621
C      -4.765405   -0.274500    5.107240
C      -4.192812    0.975307    4.930498
C      -3.085541    1.080961    4.098979
H      -2.786650   -2.172524    3.147343
H      -4.757405   -2.375216    4.657639
F      -5.836759   -0.387925    5.927669
H      -4.604190    1.841968    5.440864
H       0.528633   -0.841188   -2.706092
H      -2.708362   -3.236128   -4.234554
C      -2.795269   -0.000770    0.639694

Frequencies (cm-1)
   100.00    100.00    100.00    100.00    100.00    100.00    100.00    100.00    100.00    108.57
   120.15    138.24    143.79    159.99    171.48    175.74    201.54    204.66    212.80    223.20
   246.46    252.79    286.59    296.28    300.51    327.76    349.20    356.18    397.53    402.54
   404.48    409.07    437.31    459.92    478.36    499.06    505.58    520.34    531.88    540.17
   575.14    585.04    601.73    618.09    622.82    631.96    673.83    715.66    730.45    736.50
   744.97    747.97    781.73    800.86    807.13    809.63    825.94    830.34    855.60    875.53
   878.78    887.93    919.71    935.34    949.50    969.43    970.51    976.32    992.85    995.18
   999.90   1002.52   1023.12   1064.87   1083.07   1093.02   1099.01   1111.91   1115.17   1146.41
  1149.61   1150.93   1158.00   1164.65   1189.64   1204.91   1210.77   1227.10   1251.86   1257.51
  1274.14   1295.85   1298.43   1304.07   1318.10   1329.46   1335.99   1349.08   1355.17   1383.42
  1385.95   1394.25   1406.80   1422.93   1426.96   1429.84   1438.86   1442.21   1477.64   1491.11
  1523.40   1542.91   1580.16   1582.24   1600.70   1611.17   1614.20   1618.26   2998.11   3000.20
  3003.75   3010.46   3056.81   3078.10   3086.24   3107.13   3146.41   3147.09   3149.59   3151.19
  3156.28   3160.21   3164.39   3171.92   3172.88   3175.29   3195.62   3756.28
Note: any frequencies below 100 cm-1 (including spurious imaginary ones) are upscaled to 100 cm-1
      for the calculation of thermodynamic properties.
      (Averkiev, Truhlar, Catal. Sci. Technol. 2011, 1, 1526)

Thermodynamics

Note: this script does not take into account the spin entropy
For more info, see eq. 3 of Inorg. Chem. 2002, 41, 6928-6935
(M. Reiher), https://doi.org/10.1021/ic025891l

Temperature is now: 298.150

Reporting max. of 0 frequencies (set by $GETFREQSMAX env. variable)


Reading 1 outputfiles

            ScnFrq(1)
---------------------
```

```
--------------------
(ZPVE)        229.638
(dH,0->T)      16.808
(-TS)         -50.190
(dGibbs)      196.255
====================

Corresponding output files
 1 : frq_rev_frq_ts_FOMe-gamma-CD-pA_1_2.1282786.out


System rev_frq_ts_FOMe-gamma-mixOB-pA_1_2

Reading 1 outputfiles

       Pauli        Elstat        OrbInt       Disp.       Solv.         TOTAL          Erel   Symm.      <S2>
------------------------------------------------------------------------------------------------------------------
-----
   28165.382     -5969.956    -29072.761     -27.237     -48.407     -6953.146         0.000   NOSYM     0.760
ADF(1)

Corresponding output files
 1 : rev_frq_ts_FOMe-gamma-mixOB-pA_1_2.1286009.out

Coordinates (Angs)
  48
rev_frq_ts_FOMe-ipsgamma-AF-pA_1_2.1286009.out        -6953.1589
C       0.000000    0.000000    3.890751
C       0.838838   -0.212123    2.813600
C       1.619354    0.825026    2.281485
C       1.578500    2.089893    2.894510
C       0.724215    2.321185    3.949499
C      -0.082156    1.282899    4.453193
C       2.488588    0.541976    1.128270
N       1.931760    0.000000    0.085483
C       2.646854   -0.608816   -1.027676
C       0.967579   -2.502050   -0.946177
N       0.046254   -1.931447   -0.148153
C      -0.753158   -2.669912    0.634406
C      -0.642345   -4.048169    0.674244
C       0.318721   -4.662099   -0.120734
C       1.122668   -3.883759   -0.942543
Cu      0.000000    0.000000    0.000000
O      -1.816155    0.011492   -0.076906
H       4.157287    1.650396   -0.753974
O      -0.046669    1.748439    0.184939
H      -1.483307   -2.123788    1.225229
H      -1.298207   -4.618521    1.324779
H       0.438430   -5.742485   -0.104253
H       1.869869   -4.334651   -1.589075
H       4.011835    0.177837    3.297593
H       6.432083    0.687114    3.555424
H      -2.049408    0.901403    0.238826
H      -1.142787   -0.217477    6.466477
H      -2.298258    1.099614    6.824085
H      -2.424027    0.206638    5.279974
H       2.198063    2.896229    2.510024
H       0.655906    3.305128    4.406875
H       0.932440   -1.215144    2.405665
H      -0.571527   -0.828943    4.293977
O      -0.883069    1.606753    5.478053
F       7.905820    1.728090    1.695804
C       1.757651   -1.577031   -1.809649
H       6.556467    2.179402   -0.468454
C      -1.734718    0.598468    6.036603
C       3.919820    0.840982    1.241911
C       4.566948    0.602591    2.465629
C       5.913869    0.883284    2.620914
C       6.597599    1.438857    1.548720
C       5.986897    1.720170    0.334675
C       4.646635    1.406649    0.183377
H       3.531266   -1.126167   -0.638053
H       2.979826    0.163102   -1.727679
H       2.393533   -2.146095   -2.491528
H       1.056880   -1.003303   -2.430702

Frequencies (cm-1)
  100.00    100.00    100.00    100.00    100.00    100.00    100.00    100.00    100.00    100.00
  109.83    133.84    148.37    151.37    169.90    202.16    207.20    211.83    215.24    237.43
  253.81    268.19    285.73    302.35    319.34    338.72    366.22    392.16    393.36    401.23
  407.35    411.69    420.96    435.53    483.08    492.63    501.36    508.78    548.35    566.02
  593.47    594.43    610.58    612.81    622.86    656.88    678.36    698.73    731.09    744.70
  745.41    756.05    783.14    790.00    794.10    795.35    823.81    826.26    831.20    860.16
  875.80    890.88    919.84    944.99    947.87    952.21    956.29    958.40    977.43    995.31
  995.56   1000.37   1014.66   1024.63   1028.79   1054.13   1066.45   1101.89   1113.52   1114.93
 1119.03   1146.88   1151.66   1161.35   1167.47   1184.58   1192.22   1212.63   1221.07   1234.67
 1276.41   1279.25   1285.45   1294.32   1310.22   1323.28   1331.42   1349.68   1357.61   1368.41
 1411.15   1411.89   1418.96   1421.06   1428.45   1433.95   1439.18   1448.46   1480.83   1491.02
 1500.60   1551.15   1564.31   1584.53   1592.50   1606.34   1613.66   1616.57   2981.68   3008.73
 3010.96   3079.22   3081.33   3106.84   3133.61   3153.82   3157.48   3158.00   3160.46   3165.53
 3168.26   3168.38   3175.33   3177.60   3181.73   3184.68   3186.55   3694.35
Note: any frequencies below 100 cm-1 (including spurious imaginary ones) are upscaled to 100 cm-1
      for the calculation of thermodynamic properties.
      (Averkiev, Truhlar, Catal. Sci. Technol. 2011, 1, 1526)
```

```
Thermodynamics

Note: this script does not take into account the spin entropy
For more info, see eq. 3 of Inorg. Chem. 2002, 41, 6928-6935
(M. Reiher), https://doi.org/10.1021/ic025891l

Temperature is now: 298.150

Reporting max. of 0 frequencies (set by $GETFREQSMAX env. variable)


Reading 1 outputfiles

            ScnFrq(1)
---------------------
---------------------
(ZPVE)        229.424
(dH,0->T)      16.742
(-TS)         -49.961
(dGibbs)      196.205
=====================

Corresponding output files
 1 : frq_rev_frq_ts_FOMe-gamma-mixOB-pA_1_2.1286208.out


System rev_frq_ts_FOMe-ipso-AO-pA_1_2

Reading 1 outputfiles

       Pauli        Elstat        OrbInt       Disp.       Solv.         TOTAL          Erel   Symm.      <S2>
-----------------------------------------------------------------------------------------------------------------------
-----
   28236.769     -5977.471    -29148.517     -25.633     -42.853     -6957.875         0.000   NOSYM     0.752
ADF(1)

Corresponding output files
 1 : rev_frq_ts_FOMe-ipso-AO-pA_1_2.1282198.out

Coordinates (Angs)
  48
fwd_frq_ts_FOMe-ipso-AF-pA_1_2.1282198.out        -6957.8225
C       0.000000    0.000000    3.465634
C       0.189959    0.922800    2.444640
C      -0.869444    1.708422    1.965130
C      -2.126362    1.587882    2.572816
C      -2.331702    0.653362    3.569141
C      -1.276780   -0.165719    4.008141
C      -0.648388    2.571856    0.784154
N      -0.215112    1.929079   -0.260757
C       0.285252    2.565122   -1.470822
C       2.229174    0.907299   -1.604086
N       1.888346    0.000000   -0.666276
C       2.836809   -0.687230   -0.013517
C       4.185954   -0.494020   -0.258308
C       4.556375    0.453166   -1.206020
C       3.569535    1.156349   -1.884891
Cu      0.000000    0.000000    0.000000
O      -0.518355   -2.691188   -0.340229
H      -1.640775    4.359214   -1.095571
O      -0.491701   -1.649679    0.614252
H       2.485421   -1.407129    0.722663
H       4.923163   -1.074627    0.288375
H       5.606288    0.639718   -1.419033
H       3.829054    1.894446   -2.639110
H      -0.254011    4.029573    2.974736
H      -0.602062    6.469359    3.277352
H      -0.164736   -3.437416    0.177886
H       0.251352   -1.447254    5.877745
H      -1.059756   -2.639359    6.109717
H      -0.192665   -2.568449    4.547307
H      -2.954922    2.203018    2.228883
H      -3.314672    0.525841    4.016804
H       1.196333    1.087842    2.062418
H       0.844967   -0.579801    3.820573
O      -1.590440   -1.070077    4.958141
F      -1.497679    8.054117    1.429935
C       1.106019    1.591039   -2.318822
H      -1.997674    6.778968   -0.769426
C      -0.574461   -1.978770    5.389910
C      -0.884546    4.014356    0.907396
C      -0.626064    4.626294    2.146384
C      -0.816453    5.986360    2.327892
C      -1.300399    6.729169    1.261684
C      -1.598872    6.162255    0.031347
C      -1.380563    4.804634   -0.141862
H       0.877407    3.451423   -1.208414
H      -0.551380    2.897292   -2.094385
H       1.497258    2.144050   -3.177118
H       0.432809    0.819296   -2.715995

Frequencies (cm-1)
Traceback (most recent call last):
  File "/users/marcel/msxc/bin/getALLfreqs", line 223, in <module>
    ProcessFile(MyArguments[i])
```

```
  File "/users/marcel/msxc/bin/getALLfreqs", line 42, in ProcessFile
    Text = open(FileName)
IOError: [Errno 2] No such file or directory: 'frq_rev_frq_ts_FOMe-ipso-AO-pA_1_2.1282993.out'

Note: any frequencies below 100 cm-1 (including spurious imaginary ones) are upscaled to 100 cm-1
      for the calculation of thermodynamic properties.
      (Averkiev, Truhlar, Catal. Sci. Technol. 2011, 1, 1526)

Thermodynamics

Note: this script does not take into account the spin entropy
For more info, see eq. 3 of Inorg. Chem. 2002, 41, 6928-6935
(M. Reiher), https://doi.org/10.1021/ic025891l

Temperature is now: 298.150

Reporting max. of 0 frequencies (set by $GETFREQSMAX env. variable)


Reading 1 outputfiles

Traceback (most recent call last):
  File "/users/marcel/msxc/bin/getfreqs", line 843, in <module>
    ProcessFile(Files[i])
  File "/users/marcel/msxc/bin/getfreqs", line 659, in ProcessFile
    Text = open(FileName)
IOError: [Errno 2] No such file or directory: 'frq_rev_frq_ts_FOMe-ipso-AO-pA_1_2.1282993.out'


System rev_frq_ts_FOMe-ipso-AO-pA_1_2

Reading 1 outputfiles

       Pauli        Elstat        OrbInt       Disp.       Solv.         TOTAL          Erel   Symm.      <S2>
-------------------------------------------------------------------------------------------------------------------
-----
   28236.769     -5977.471    -29148.517     -25.633     -42.853     -6957.875         0.000   NOSYM     0.752
ADF(1)

Corresponding output files
 1 : rev_frq_ts_FOMe-ipso-AO-pA_1_2.1282198.out

Coordinates (Angs)
  48
fwd_frq_ts_FOMe-ipso-AF-pA_1_2.1282198.out        -6957.8225
C       0.000000    0.000000    3.465634
C       0.189959    0.922800    2.444640
C      -0.869444    1.708422    1.965130
C      -2.126362    1.587882    2.572816
C      -2.331702    0.653362    3.569141
C      -1.276780   -0.165719    4.008141
C      -0.648388    2.571856    0.784154
N      -0.215112    1.929079   -0.260757
C       0.285252    2.565122   -1.470822
C       2.229174    0.907299   -1.604086
N       1.888346    0.000000   -0.666276
C       2.836809   -0.687230   -0.013517
C       4.185954   -0.494020   -0.258308
C       4.556375    0.453166   -1.206020
C       3.569535    1.156349   -1.884891
Cu      0.000000    0.000000    0.000000
O      -0.518355   -2.691188   -0.340229
H      -1.640775    4.359214   -1.095571
O      -0.491701   -1.649679    0.614252
H       2.485421   -1.407129    0.722663
H       4.923163   -1.074627    0.288375
H       5.606288    0.639718   -1.419033
H       3.829054    1.894446   -2.639110
H      -0.254011    4.029573    2.974736
H      -0.602062    6.469359    3.277352
H      -0.164736   -3.437416    0.177886
H       0.251352   -1.447254    5.877745
H      -1.059756   -2.639359    6.109717
H      -0.192665   -2.568449    4.547307
H      -2.954922    2.203018    2.228883
H      -3.314672    0.525841    4.016804
H       1.196333    1.087842    2.062418
H       0.844967   -0.579801    3.820573
O      -1.590440   -1.070077    4.958141
F      -1.497679    8.054117    1.429935
C       1.106019    1.591039   -2.318822
H      -1.997674    6.778968   -0.769426
C      -0.574461   -1.978770    5.389910
C      -0.884546    4.014356    0.907396
C      -0.626064    4.626294    2.146384
C      -0.816453    5.986360    2.327892
C      -1.300399    6.729169    1.261684
C      -1.598872    6.162255    0.031347
C      -1.380563    4.804634   -0.141862
H       0.877407    3.451423   -1.208414
H      -0.551380    2.897292   -2.094385
H       1.497258    2.144050   -3.177118
H       0.432809    0.819296   -2.715995

Frequencies (cm-1)
```

```
  100.00    100.00    100.00    100.00    100.00    100.00    100.00    100.00    100.00    100.00
  105.39    114.36    119.22    132.92    146.06    166.85    179.34    189.83    214.94    222.53
  235.18    254.99    276.64    289.92    298.30    320.40    326.26    357.31    393.15    397.77
  400.81    412.74    416.26    422.23    480.37    491.86    499.54    509.17    513.46    550.99
  585.43    593.72    615.09    624.57    641.23    678.37    694.62    732.60    741.55    746.02
  760.43    781.28    789.10    793.73    798.70    821.63    825.66    830.38    861.78    880.17
  906.81    919.60    934.90    942.86    950.04    950.38    955.54    974.63    994.80    997.30
 1000.82   1013.33   1023.50   1031.01   1054.17   1062.90   1102.93   1106.58   1109.46   1123.37
 1148.11   1148.89   1161.04   1167.74   1181.07   1189.58   1212.34   1219.35   1232.58   1266.74
 1278.97   1282.51   1295.62   1310.98   1322.33   1327.92   1345.30   1348.85   1357.53   1370.27
 1408.29   1414.51   1417.65   1417.75   1425.95   1428.68   1435.12   1440.39   1476.61   1492.32
 1503.88   1566.79   1573.08   1582.21   1594.75   1608.60   1613.71   1616.42   2973.82   2996.54
 3005.52   3065.22   3067.47   3095.81   3127.27   3131.74   3145.27   3147.95   3154.03   3158.81
 3163.11   3168.78   3169.29   3174.48   3178.02   3186.61   3188.10   3648.13
Note: any frequencies below 100 cm-1 (including spurious imaginary ones) are upscaled to 100 cm-1
      for the calculation of thermodynamic properties.
      (Averkiev, Truhlar, Catal. Sci. Technol. 2011, 1, 1526)

Thermodynamics

Note: this script does not take into account the spin entropy
For more info, see eq. 3 of Inorg. Chem. 2002, 41, 6928-6935
(M. Reiher), https://doi.org/10.1021/ic025891l

Temperature is now: 298.150

Reporting max. of 0 frequencies (set by $GETFREQSMAX env. variable)


Reading 1 outputfiles

            ScnFrq(1)
---------------------
---------------------
(ZPVE)        229.475
(dH,0->T)      17.031
(-TS)         -50.887
(dGibbs)      195.619
=====================

Corresponding output files
 1 : frq_rev_frq_ts_FOMe-ipso-AO-pA_1_2.1282996.out


System rev_frq_ts_FOMe-ipso-FG-pA_1_2

Reading 1 outputfiles

       Pauli        Elstat        OrbInt       Disp.       Solv.         TOTAL          Erel   Symm.      <S2>
-------------------------------------------------------------------------------------------------------------------------
-----
   28264.785     -6000.365    -29165.516     -25.658     -50.574     -6977.494         0.000   NOSYM     0.755
ADF(1)

Corresponding output files
 1 : rev_frq_ts_FOMe-ipso-FG-pA_1_2.1282760.out

Coordinates (Angs)
  48
fwd_frq_ts_FOMe-ipso-FG-pA_1_2.1282760.out        -6977.5146
C       0.000000    0.000000    4.757753
C      -0.360221    0.160527    3.462395
C      -0.623558   -0.978062    2.526789
C      -0.562142   -2.308070    3.197900
C      -0.207855   -2.457028    4.493267
C       0.071134   -1.313295    5.299593
C      -1.967506   -0.730358    1.819852
N      -1.846756   -0.144347    0.685713
C      -2.976682    0.291754   -0.119307
C      -1.727335    0.985559   -2.195387
N      -0.500091    1.051310   -1.640279
C       0.411794    1.900206   -2.140526
C       0.142137    2.730241   -3.215173
C      -1.118251    2.677222   -3.796006
C      -2.056867    1.795448   -3.279608
Cu      0.000000    0.000000    0.000000
O       1.808698    0.000000   -0.470756
H      -3.684262   -2.552843    0.841618
O       0.423787   -0.956106    1.588911
H       1.380822    1.885815   -1.650091
H       0.911999    3.403969   -3.580030
H      -1.370419    3.315864   -4.639371
H      -3.053133    1.727346   -3.707696
H      -3.010735    0.047078    4.213144
H      -5.063337   -0.841176    5.295478
H       2.248610   -0.437868    0.273327
H      -0.092453    0.161683    7.577479
H       0.992856   -1.011634    8.387673
H       1.635417    0.033874    7.082408
H      -0.744920   -3.174797    2.566201
H      -0.100528   -3.438859    4.947217
H      -0.387247    1.151716    3.013650
H       0.270114    0.860490    5.360405
O       0.421837   -1.575967    6.534721
F      -6.598973   -2.654151    4.258233
```

```
C      -2.728115    0.044061   -1.602391
H      -5.731799   -3.434793    1.941263
C       0.760695   -0.509982    7.449414
C      -3.215892   -1.181886    2.447258
C      -3.598569   -0.715292    3.709028
C      -4.744714   -1.202450    4.321573
C      -5.485409   -2.171167    3.662482
C      -5.128226   -2.667231    2.418041
C      -3.988362   -2.162447    1.810086
H      -3.096584    1.368373    0.061306
H      -3.902489   -0.198306    0.190025
H      -3.677773    0.155240   -2.131786
H      -2.400071   -0.993860   -1.749067

Frequencies (cm-1)
  100.00    100.00    100.00    100.00    100.00    100.00    100.00    100.00    100.00    116.38
  123.86    131.94    144.89    169.74    175.41    186.18    191.02    203.59    217.62    228.46
  248.00    263.08    294.82    314.93    318.08    336.74    360.70    372.12    395.96    396.31
  404.30    405.17    418.68    463.31    485.65    490.97    509.68    511.11    522.54    532.61
  579.01    604.78    605.40    628.32    641.08    665.38    679.73    720.61    742.43    753.47
  759.85    769.36    776.28    785.23    798.78    803.46    821.17    825.63    827.11    845.81
  870.74    880.34    933.05    940.66    948.29    949.77    960.22    978.28    993.99    998.44
 1002.81   1013.45   1020.96   1048.09   1055.48   1065.36   1080.66   1095.05   1106.97   1115.09
 1125.13   1145.49   1148.67   1148.87   1168.02   1176.10   1207.97   1218.45   1234.67   1235.12
 1244.43   1270.37   1276.64   1289.53   1292.53   1323.03   1325.78   1331.30   1346.24   1354.07
 1402.83   1402.93   1406.96   1418.98   1424.73   1431.07   1435.70   1436.26   1475.94   1491.02
 1541.62   1584.68   1587.03   1599.41   1611.74   1613.93   1643.52   1654.87   2968.20   2979.23
 3008.24   3010.47   3059.13   3073.76   3097.74   3130.43   3143.75   3146.36   3151.52   3151.63
 3158.32   3167.96   3170.66   3172.83   3173.46   3177.83   3192.79   3751.35
Note: any frequencies below 100 cm-1 (including spurious imaginary ones) are upscaled to 100 cm-1
      for the calculation of thermodynamic properties.
      (Averkiev, Truhlar, Catal. Sci. Technol. 2011, 1, 1526)

Thermodynamics

Note: this script does not take into account the spin entropy
For more info, see eq. 3 of Inorg. Chem. 2002, 41, 6928-6935
(M. Reiher), https://doi.org/10.1021/ic025891l

Temperature is now: 298.150

Reporting max. of 0 frequencies (set by $GETFREQSMAX env. variable)


Reading 1 outputfiles

            ScnFrq(1)
---------------------
---------------------
(ZPVE)        229.354
(dH,0->T)      16.726
(-TS)         -49.852
(dGibbs)      196.229
=====================

Corresponding output files
 1 : frq_rev_frq_ts_FOMe-ipso-FG-pA_1_2.1283701.out


System rev_frq_ts_FOMe-ipso-FG-pA_1_2

Reading 1 outputfiles

       Pauli        Elstat        OrbInt       Disp.       Solv.         TOTAL          Erel   Symm.      <S2>
-----------------------------------------------------------------------------------------------------------------
-----
   28264.785     -6000.365    -29165.516     -25.658     -50.574     -6977.494         0.000   NOSYM     0.755
ADF(1)

Corresponding output files
 1 : rev_frq_ts_FOMe-ipso-FG-pA_1_2.1282760.out

Coordinates (Angs)
  48
fwd_frq_ts_FOMe-ipso-FG-pA_1_2.1282760.out        -6977.5146
C       0.000000    0.000000    4.757753
C      -0.360221    0.160527    3.462395
C      -0.623558   -0.978062    2.526789
C      -0.562142   -2.308070    3.197900
C      -0.207855   -2.457028    4.493267
C       0.071134   -1.313295    5.299593
C      -1.967506   -0.730358    1.819852
N      -1.846756   -0.144347    0.685713
C      -2.976682    0.291754   -0.119307
C      -1.727335    0.985559   -2.195387
N      -0.500091    1.051310   -1.640279
C       0.411794    1.900206   -2.140526
C       0.142137    2.730241   -3.215173
C      -1.118251    2.677222   -3.796006
C      -2.056867    1.795448   -3.279608
Cu      0.000000    0.000000    0.000000
O       1.808698    0.000000   -0.470756
H      -3.684262   -2.552843    0.841618
O       0.423787   -0.956106    1.588911
H       1.380822    1.885815   -1.650091
```

```
H       0.911999    3.403969   -3.580030
H      -1.370419    3.315864   -4.639371
H      -3.053133    1.727346   -3.707696
H      -3.010735    0.047078    4.213144
H      -5.063337   -0.841176    5.295478
H       2.248610   -0.437868    0.273327
H      -0.092453    0.161683    7.577479
H       0.992856   -1.011634    8.387673
H       1.635417    0.033874    7.082408
H      -0.744920   -3.174797    2.566201
H      -0.100528   -3.438859    4.947217
H      -0.387247    1.151716    3.013650
H       0.270114    0.860490    5.360405
O       0.421837   -1.575967    6.534721
F      -6.598973   -2.654151    4.258233
C      -2.728115    0.044061   -1.602391
H      -5.731799   -3.434793    1.941263
C       0.760695   -0.509982    7.449414
C      -3.215892   -1.181886    2.447258
C      -3.598569   -0.715292    3.709028
C      -4.744714   -1.202450    4.321573
C      -5.485409   -2.171167    3.662482
C      -5.128226   -2.667231    2.418041
C      -3.988362   -2.162447    1.810086
H      -3.096584    1.368373    0.061306
H      -3.902489   -0.198306    0.190025
H      -3.677773    0.155240   -2.131786
H      -2.400071   -0.993860   -1.749067

Frequencies (cm-1)
   100.00    100.00    100.00    100.00    100.00    100.00    100.00    100.00    100.00    111.49
   139.54    142.91    151.91    169.12    180.28    192.14    192.83    213.33    218.01    231.72
   252.45    268.00    297.82    304.47    310.35    340.94    356.97    384.02    399.52    403.01
   408.03    410.19    423.56    456.04    469.12    486.46    502.89    507.50    520.65    549.52
   574.19    575.95    587.72    616.25    640.53    674.81    689.03    717.69    730.61    743.47
   753.41    757.79    764.96    783.60    799.67    813.67    815.75    825.64    848.25    853.09
   878.34    916.01    935.50    939.59    946.90    950.94    958.79    961.75    971.91    980.73
   995.04   1000.45   1008.00   1013.68   1030.16   1061.35   1065.61   1078.59   1096.29   1101.44
  1110.46   1110.85   1149.41   1151.23   1158.86   1167.95   1168.30   1212.11   1219.21   1235.35
  1241.99   1261.08   1280.47   1296.34   1303.43   1314.24   1317.42   1325.99   1344.09   1355.18
  1391.99   1408.38   1411.47   1422.30   1425.23   1429.42   1431.96   1435.12   1476.30   1478.51
  1491.94   1536.10   1584.14   1595.83   1611.22   1615.59   1625.15   1652.74   3003.72   3005.19
  3011.14   3091.79   3096.71   3114.86   3146.17   3148.12   3150.35   3151.59   3155.74   3157.66
  3163.56   3168.93   3169.19   3173.16   3175.05   3178.09   3184.60   3744.77
Note: any frequencies below 100 cm-1 (including spurious imaginary ones) are upscaled to 100 cm-1
      for the calculation of thermodynamic properties.
      (Averkiev, Truhlar, Catal. Sci. Technol. 2011, 1, 1526)

Thermodynamics

Note: this script does not take into account the spin entropy
For more info, see eq. 3 of Inorg. Chem. 2002, 41, 6928-6935
(M. Reiher), https://doi.org/10.1021/ic025891l

Temperature is now: 298.150

Reporting max. of 0 frequencies (set by $GETFREQSMAX env. variable)


Reading 1 outputfiles

            ScnFrq(1)
---------------------
---------------------
(ZPVE)        229.146
(dH,0->T)      16.706
(-TS)         -49.770
(dGibbs)      196.081
=====================

Corresponding output files
 1 : frq_rev_frq_ts_FOMe-ipso-FG-pA_1_2.1283710.out


System rev_frq_ts_FOMe-ipso-GH-pA_1_2

Reading 1 outputfiles

       Pauli        Elstat        OrbInt       Disp.       Solv.         TOTAL          Erel   Symm.      <S2>
-----------------------------------------------------------------------------------------------------------------
-----
   28384.899     -6023.776    -29284.840     -26.543     -49.103     -6999.536         0.000   NOSYM     0.753
ADF(1)

Corresponding output files
 1 : rev_frq_ts_FOMe-ipso-GH-pA_1_2.1282768.out

Coordinates (Angs)
  48
rev_frq_ts_FOMe-ipso-GH-pA_1_2.1282768.out        -6999.5425
C       0.000000   -0.000000    4.489870
C      -0.062892   -0.173701    3.019698
C       0.586134   -1.419916    2.447310
C       1.090421   -2.400743    3.355286
C       1.075722   -2.165033    4.691996
```

```
C       0.546532   -0.955715    5.274690
C      -1.481388   -0.087329    2.452604
N      -1.612050    0.116346    1.192611
C      -2.944805    0.185889    0.587182
C      -2.144643    1.214118   -1.560958
N      -0.821948    1.280515   -1.319932
C      -0.085630    2.268311   -1.844293
C      -0.646694    3.258958   -2.634109
C      -2.013545    3.216359   -2.882244
C      -2.767645    2.182169   -2.343350
Cu      0.000000    0.000000    0.000000
O       1.645855    0.000000   -0.885193
H      -2.072571   -2.389521    3.693558
O       0.658098   -1.561059    1.207803
H       0.976639    2.237162   -1.616183
H      -0.017880    4.045437   -3.041060
H      -2.489497    3.980468   -3.492524
H      -3.837085    2.115321   -2.524765
H      -3.354864    1.707557    3.309808
H      -5.183434    1.275386    4.944287
H       2.184031   -0.697918   -0.483555
H      -3.403360    1.141152    0.867621
H      -3.578654   -0.612172    0.990287
H      -3.916172    0.059022   -1.304944
H       1.506009   -3.315292    2.940756
H       1.479983   -2.907436    5.378365
H       0.489366    0.661898    2.555760
H      -0.424931    0.912043    4.893429
H       0.247355    0.030632    8.352068
F      -5.626301   -1.016933    6.071724
C      -2.889219    0.080504   -0.930746
H      -3.924863   -2.813920    5.294760
H      -2.422208   -0.871195   -1.217600
C      -2.602281   -0.314677    3.386347
C      -3.479296    0.714267    3.733762
C      -4.499154    0.484897    4.648707
C      -4.630833   -0.785952    5.185744
C      -3.782081   -1.831192    4.853613
C      -2.752847   -1.582813    3.957792
O       0.649394   -0.926303    6.620501
H       0.594282    1.129853    6.982301
C       0.088576    0.205080    7.287437
H      -0.986460    0.285223    7.078953

Frequencies (cm-1)
   100.00    100.00    100.00    100.00    100.00    100.00    100.00    100.00    100.00    117.45
   125.34    139.11    147.20    165.32    178.91    180.43    202.27    208.97    219.50    231.71
   250.42    264.40    310.72    318.14    321.04    329.63    358.19    365.83    395.30    397.86
   403.50    407.92    423.31    463.36    487.22    490.34    506.56    510.21    521.68    532.17
   578.68    603.07    607.76    628.90    641.53    651.03    681.57    727.23    743.54    747.67
   757.80    772.18    778.72    786.77    800.04    804.70    823.55    825.76    828.00    841.92
   873.41    880.04    933.11    941.29    950.21    955.36    964.26    978.07    994.70    999.87
  1001.05   1014.69   1021.03   1056.38   1059.40   1068.91   1082.36   1097.40   1106.80   1113.06
  1125.69   1146.08   1148.44   1149.42   1167.78   1169.37   1211.12   1220.14   1229.17   1239.19
  1245.43   1269.79   1279.75   1284.32   1294.70   1323.55   1327.27   1331.89   1345.46   1357.66
  1403.67   1404.91   1409.98   1417.31   1425.15   1431.60   1435.78   1436.29   1477.10   1492.33
  1542.78   1584.76   1587.70   1599.47   1611.45   1614.00   1635.57   1657.01   2959.68   2966.27
  3007.47   3008.84   3057.21   3071.26   3097.53   3131.49   3145.67   3146.02   3150.73   3152.59
  3159.93   3167.68   3170.41   3172.01   3174.66   3177.98   3188.04   3750.67
Note: any frequencies below 100 cm-1 (including spurious imaginary ones) are upscaled to 100 cm-1
      for the calculation of thermodynamic properties.
      (Averkiev, Truhlar, Catal. Sci. Technol. 2011, 1, 1526)

Thermodynamics

Note: this script does not take into account the spin entropy
For more info, see eq. 3 of Inorg. Chem. 2002, 41, 6928-6935
(M. Reiher), https://doi.org/10.1021/ic025891l

Temperature is now: 298.150

Reporting max. of 0 frequencies (set by $GETFREQSMAX env. variable)


Reading 1 outputfiles

            ScnFrq(1)
---------------------
---------------------
(ZPVE)        229.425
(dH,0->T)      16.685
(-TS)         -49.746
(dGibbs)      196.364
=====================

Corresponding output files
 1 : frq_rev_frq_ts_FOMe-ipso-GH-pA_1_2.1283703.out


System rev_frq_ts_FOMe-ipso-OF-pA_1_2

Reading 1 outputfiles

       Pauli        Elstat        OrbInt       Disp.       Solv.         TOTAL          Erel   Symm.      <S2>
```

```
--------------------------------------------------------------------------------------------------------------------
-----
   28164.876     -5969.745    -29072.472     -27.234     -48.411     -6953.154         0.000   NOSYM     0.760
ADF(1)

Corresponding output files
 1 : rev_frq_ts_FOMe-ipso-OF-pA_1_2.1286011.out

Coordinates (Angs)
  48
rev_frq_ts_FOMe-ipso-preFF-pA_1_2.1286011.out        -6953.1635
C       0.000000    0.000000    3.891485
C      -0.838825    0.204046    2.812972
C      -1.610071   -0.840323    2.280626
C      -1.558008   -2.104801    2.893828
C      -0.702753   -2.328068    3.949442
C       0.092703   -1.281976    4.454736
C      -2.483441   -0.563510    1.129348
N      -1.933270   -0.013065    0.087679
C      -2.656681    0.596110   -1.020487
C      -0.984078    2.493317   -0.951360
N      -0.058497    1.930604   -0.152644
C       0.735592    2.676010    0.628710
C       0.614006    4.053392    0.667224
C      -0.351198    4.659182   -0.129046
C      -1.149123    3.873862   -0.950086
Cu      0.000000    0.000000    0.000000
O       1.815922   -0.000000   -0.080074
H      -4.134530   -1.698597   -0.750153
O       0.057941   -1.747402    0.192751
H       1.470188    2.136136    1.219801
H       1.264832    4.629377    1.317851
H      -0.478760    5.738658   -0.114174
H      -1.899769    4.318310   -1.597104
H      -4.013453   -0.209448    3.296001
H      -6.427155   -0.750854    3.553514
H       2.056072   -0.888022    0.235670
H       1.128225    0.227391    6.474800
H       2.297071   -1.077145    6.834814
H       2.418906   -0.180310    5.292628
H      -2.169890   -2.916691    2.508669
H      -0.625478   -3.311164    4.407180
H      -0.940530    1.205798    2.403821
H       0.562855    0.834361    4.295801
O       0.892827   -1.597853    5.482466
F      -7.884867   -1.818047    1.696158
C      -1.770498    1.560621   -1.809951
H      -6.527624   -2.258425   -0.465310
C       1.731077   -0.581054    6.045916
C      -3.910983   -0.878991    1.242960
C      -4.562127   -0.644874    2.465233
C      -5.905417   -0.943061    2.620137
C      -6.580534   -1.511428    1.549233
C      -5.964976   -1.788813    0.336705
C      -4.628746   -1.458291    0.185722
H      -2.407783    2.123282   -2.495809
H      -1.068419    0.983659   -2.426403
H      -3.536029    1.116611   -0.623530
H      -2.998000   -0.175001   -1.717193

Frequencies (cm-1)
   100.00    100.00    100.00    100.00    100.00    100.00    100.00    100.00    100.00    100.00
   109.73    133.45    148.21    150.94    169.85    201.91    206.67    211.48    215.06    237.73
   254.80    268.29    285.64    302.46    319.21    338.98    366.13    392.59    393.20    401.40
   407.25    411.43    420.70    435.21    482.90    492.77    501.52    508.98    548.31    566.08
   593.17    593.82    610.44    612.70    622.79    656.92    678.48    698.55    731.16    744.64
   745.38    756.06    782.73    789.94    794.13    795.61    823.86    826.15    831.21    860.09
   875.81    891.01    919.80    945.28    947.96    952.58    956.34    958.37    977.15    995.24
   995.42   1000.35   1014.57   1024.68   1028.76   1053.55   1066.11   1101.63   1113.51   1114.99
  1119.11   1146.73   1151.57   1161.31   1167.44   1184.27   1192.37   1212.07   1220.92   1234.41
  1276.58   1279.26   1285.49   1294.36   1310.33   1323.42   1331.10   1349.38   1357.44   1368.31
  1410.64   1411.99   1418.76   1420.98   1428.44   1433.95   1439.20   1449.00   1480.81   1490.99
  1500.56   1550.68   1564.81   1584.49   1592.61   1606.44   1613.76   1616.66   2981.71   3008.97
  3011.70   3079.28   3082.52   3106.61   3133.78   3153.74   3157.25   3158.17   3160.15   3165.41
  3167.63   3168.25   3175.30   3177.50   3180.83   3184.71   3186.38   3694.88
Note: any frequencies below 100 cm-1 (including spurious imaginary ones) are upscaled to 100 cm-1
      for the calculation of thermodynamic properties.
      (Averkiev, Truhlar, Catal. Sci. Technol. 2011, 1, 1526)

Thermodynamics

Note: this script does not take into account the spin entropy
For more info, see eq. 3 of Inorg. Chem. 2002, 41, 6928-6935
(M. Reiher), https://doi.org/10.1021/ic025891l

Temperature is now: 298.150

Reporting max. of 0 frequencies (set by $GETFREQSMAX env. variable)


Reading 1 outputfiles

            ScnFrq(1)
---------------------
---------------------
```

```
(ZPVE)        229.417
(dH,0->T)      16.744
(-TS)         -49.967
(dGibbs)      196.193
=====================

Corresponding output files
 1 : frq_rev_frq_ts_FOMe-ipso-OF-pA_1_2.1286210.out


System ts_FOMe-gamma-AB-pA_1_2

Reading 1 outputfiles

       Pauli        Elstat        OrbInt       Disp.       Solv.         TOTAL          Erel   Symm.      <S2>
-------------------------------------------------------------------------------------------------------------------
-----
   28023.964     -5931.334    -28962.612     -25.499     -44.787     -6940.436         0.000   NOSYM     0.762
ADF(1)

Corresponding output files
 1 : ts_FOMe-gamma-AB-pA_1_2.1281136.out

Coordinates (Angs)
  48
ts_FOMe-gamma-AB-pA_1_2.1281136.out        -6940.4481
C      -3.654732   -3.000516    1.722626
C      -2.483673   -2.520176    2.290779
C      -2.084988   -1.195576    2.113061
C      -2.931544   -0.320974    1.392573
C      -4.131143   -0.787737    0.863253
C      -4.484683   -2.135653    0.994351
C      -0.821634   -0.731551    2.684480
N       0.000000   -0.000000    1.989617
C       1.143876    0.634966    2.661082
C       2.707111    0.713446    0.716030
N       1.952638    0.000000   -0.140098
C       2.529522   -0.769872   -1.076157
C       3.904896   -0.877282   -1.186752
C       4.700108   -0.157120   -0.302562
C       4.095073    0.648047    0.653604
Cu      0.000000    0.000000    0.000000
O      -0.518219    0.034071   -1.839739
H      -2.309014   -0.071707    4.811517
O      -1.746284   -0.217604   -0.459866
H       1.858610   -1.303786   -1.744800
H       4.335618   -1.516577   -1.951635
H       5.784300   -0.221590   -0.357186
H       4.688819    1.226201    1.356079
H       1.803368   -0.120281    3.099298
H       1.314233    2.245873    1.230672
H      -0.831334    0.935785   -2.032123
H      -6.231296   -4.135770    1.581879
H      -7.007155   -3.926928   -0.014805
H      -5.333497   -4.547128    0.081868
H      -2.728205    0.745355    1.400415
H      -4.800801   -0.110722    0.338909
H      -1.853907   -3.202242    2.856847
H      -3.916252   -4.045136    1.854184
O      -5.642332   -2.504508    0.411963
H       2.673607    2.084873    2.342074
C       1.965202    1.517557    1.732988
H       0.754080    1.243068    3.485800
C      -6.065299   -3.865864    0.532115
C      -0.562614   -1.113515    4.091757
C       0.518693   -1.929508    4.439605
C       0.713621   -2.312795    5.758998
C      -0.172226   -1.846119    6.718889
C      -1.253914   -1.036511    6.409635
C      -1.455086   -0.688702    5.081245
H       1.197095   -2.296845    3.672989
H       1.534782   -2.964165    6.045454
F       0.022188   -2.204777    8.008532
H      -1.926999   -0.699507    7.193300

Frequencies (cm-1)
 -421.83    -35.01    -27.83    -22.16    100.00    100.00    100.00    100.00    100.00    100.00
  112.18    117.77    125.05    144.53    159.80    167.64    177.03    187.18    207.11    218.53
  222.41    242.00    264.82    301.09    324.79    331.90    350.11    378.76    399.19    403.44
  408.39    409.34    430.46    467.12    473.80    492.42    507.86    510.45    520.30    551.10
  565.12    576.28    589.28    611.91    616.39    650.10    679.96    691.08    730.83    741.98
  744.39    756.57    779.99    786.38    798.32    804.30    820.26    826.55    827.21    863.72
  872.85    878.83    915.45    935.10    937.55    945.48    948.45    952.17    961.70    993.81
  994.17   1001.17   1020.62   1025.45   1031.97   1060.36   1071.47   1095.01   1106.41   1110.44
 1122.02   1144.99   1152.01   1159.10   1165.98   1169.34   1184.21   1209.15   1221.08   1238.41
 1264.91   1274.95   1284.15   1297.69   1312.71   1324.96   1337.41   1344.97   1359.20   1367.34
 1406.96   1409.35   1413.01   1414.10   1419.94   1427.79   1436.97   1438.77   1484.64   1489.09
 1499.80   1547.35   1563.26   1586.52   1598.09   1601.90   1609.36   1618.96   2975.99   3007.24
 3013.93   3070.35   3079.85   3098.38   3127.89   3147.82   3151.27   3152.62   3153.79   3157.35
 3157.64   3171.48   3171.54   3173.31   3174.23   3180.98   3184.92   3700.06
Note: any frequencies below 100 cm-1 (including spurious imaginary ones) are upscaled to 100 cm-1
      for the calculation of thermodynamic properties.
      (Averkiev, Truhlar, Catal. Sci. Technol. 2011, 1, 1526)

Thermodynamics
```

```
Note: this script does not take into account the spin entropy
For more info, see eq. 3 of Inorg. Chem. 2002, 41, 6928-6935
(M. Reiher), https://doi.org/10.1021/ic025891l

Temperature is now: 298.150

Reporting max. of 0 frequencies (set by $GETFREQSMAX env. variable)


Reading 1 outputfiles

            ScnFrq(1)
---------------------
---------------------
(ZPVE)        228.555
(dH,0->T)      17.040
(-TS)         -50.820
(dGibbs)      194.775
=====================

Corresponding output files
 1 : frq_ts_FOMe-gamma-AB-pA_1_2.1281281.out


System ts_FOMe-gamma-BC-pA_1_2

Reading 1 outputfiles

       Pauli        Elstat        OrbInt       Disp.       Solv.         TOTAL          Erel   Symm.      <S2>
-----------------------------------------------------------------------------------------------------------------------
-----
   28379.008     -6050.459    -29223.627     -25.154     -50.453     -6970.855         0.000   NOSYM     0.755
ADF(1)

Corresponding output files
 1 : ts_FOMe-gamma-BC-pA_1_2.1304844.out

Coordinates (Angs)
  48
ts_FOMe-gamma-BC-pA_1_2.1304844.out        -6970.8553
C      -3.343125   -3.446293    1.882082
C      -2.369624   -2.704977    2.526103
C      -1.849825   -1.519555    2.002057
C      -2.452039   -0.986366    0.768836
C      -3.440574   -1.836700    0.090094
C      -3.887784   -3.025283    0.654182
C      -0.756259   -0.827970    2.659593
N       0.000000    0.000000    1.993677
C       1.033401    0.762027    2.691197
C       2.672331    0.781543    0.805047
N       1.985048   -0.000000   -0.048204
C       2.631078   -0.794817   -0.912422
C       4.014206   -0.856589   -0.953246
C       4.741785   -0.062172   -0.074793
C       4.063561    0.766206    0.809778
Cu      0.000000    0.000000    0.000000
O      -0.105057   -0.086300   -1.862897
H      -2.232719    0.076698    4.726479
O      -1.938031    0.035939    0.102490
H       2.001772   -1.374877   -1.581881
H       4.503106   -1.516816   -1.663599
H       5.829024   -0.085345   -0.078580
H       4.601372    1.404131    1.505808
H       1.696963    0.096949    3.255244
H       1.204866    2.285331    1.172539
H      -1.056800   -0.129586   -2.041046
H       1.214889   -2.327173    3.793867
C      -5.335403   -3.468988   -1.185632
F       0.136162   -1.696818    8.113121
H      -3.445104   -0.627673    1.297178
H      -3.851304   -1.421308   -0.824439
H      -1.969560   -3.081377    3.463664
H      -3.708173   -4.370038    2.323511
O      -4.797234   -3.830549    0.094487
H       2.524132    2.248098    2.341839
C       1.862520    1.616339    1.743806
H       0.537461    1.410144    3.425067
H      -1.817349   -0.283230    7.158264
C      -0.527502   -1.089988    4.104852
C       0.550114   -1.873564    4.525822
C       0.774977   -2.090023    5.878993
C      -0.085718   -1.500622    6.792056
C      -1.165760   -0.722328    6.407564
C      -1.387744   -0.526847    5.049949
H      -6.024205   -4.272612   -1.446318
H      -5.878392   -2.519042   -1.125793
H      -4.538511   -3.405085   -1.935453
H       1.602433   -2.702938    6.226205

Frequencies (cm-1)
 -617.66    -31.77    -22.50    -15.81    100.00    100.00    100.00    100.00    100.00    111.82
  123.72    124.71    152.21    170.70    185.12    196.20    202.01    218.88    221.58    226.61
  238.17    249.37    256.21    291.48    322.59    327.78    342.00    357.14    372.45    408.07
  411.71    416.56    421.89    443.10    462.13    488.88    507.41    510.82    519.89    537.31
```

```
  560.88    569.23    579.81    591.63    621.74    640.37    647.52    684.25    703.19    725.42
  736.86    744.87    757.33    776.00    777.28    818.25    829.11    834.17    839.72    864.30
  872.49    877.15    890.19    919.20    938.10    956.88    962.14    964.73    968.19    988.49
  991.59    996.25   1014.76   1018.57   1029.97   1057.65   1068.22   1079.35   1101.66   1108.72
 1121.00   1141.31   1149.26   1153.15   1161.30   1169.97   1195.47   1207.66   1219.60   1232.42
 1239.66   1259.62   1285.93   1294.82   1309.14   1322.32   1329.44   1343.74   1354.15   1368.61
 1373.76   1395.16   1409.82   1415.53   1428.80   1430.15   1436.96   1437.02   1467.91   1473.28
 1479.08   1521.03   1547.44   1574.94   1585.56   1591.61   1616.30   1618.84   2395.73   2980.93
 3001.08   3008.59   3061.22   3079.63   3099.85   3137.48   3147.78   3152.48   3159.62   3159.79
 3161.14   3163.39   3169.67   3172.42   3172.76   3178.48   3186.46   3736.95
Note: any frequencies below 100 cm-1 (including spurious imaginary ones) are upscaled to 100 cm-1
      for the calculation of thermodynamic properties.
      (Averkiev, Truhlar, Catal. Sci. Technol. 2011, 1, 1526)

Thermodynamics

Note: this script does not take into account the spin entropy
For more info, see eq. 3 of Inorg. Chem. 2002, 41, 6928-6935
(M. Reiher), https://doi.org/10.1021/ic025891l

Temperature is now: 298.150

Reporting max. of 0 frequencies (set by $GETFREQSMAX env. variable)


Reading 1 outputfiles

            ScnFrq(1)
---------------------
---------------------
(ZPVE)        227.033
(dH,0->T)      16.925
(-TS)         -50.204
(dGibbs)      193.755
=====================

Corresponding output files
 1 : frq_ts_FOMe-gamma-BC-pA_1_2.1303893.out


System ts_FOMe-gamma-CD-pA_1_2

Reading 1 outputfiles

       Pauli        Elstat        OrbInt       Disp.       Solv.         TOTAL          Erel   Symm.      <S2>
-------------------------------------------------------------------------------------------------------------------
-----
   28518.003     -6069.859    -29378.232     -26.058     -43.534     -6999.854         0.000   NOSYM     0.753
ADF(1)

Corresponding output files
 1 : ts_FOMe-gamma-CD-pA_1_2.1281140.out

Coordinates (Angs)
  48
ts_FOMe-gamma-CD-pA_1_2.1281140.out        -6999.8536
C       2.227719   -1.783750    4.902499
C       1.607816   -0.695868    4.294975
C       2.430464    0.338154    3.742931
C       3.849664    0.063816    3.562769
C       4.426182   -1.068270    4.258010
C       3.609171   -1.980756    4.910464
C       0.161618   -0.668514    4.064757
H       0.000000   -0.000000    6.654461
O       1.943637    1.386871    3.198445
Cu      1.110326    0.360213    1.614399
O       2.957445   -0.000000    1.003801
C      -1.417042   -0.222847   -2.374001
C      -1.915904   -0.874452    1.313525
H      -2.326811   -0.308288    3.348802
H      -1.816190    1.034320    2.313928
N       0.000000    0.000000    0.000000
H      -3.027750   -0.906781   -1.119227
C      -1.276032   -0.442952    0.024582
N      -0.257378   -0.148477    2.951925
H      -2.998485   -0.903216    1.160127
H      -1.606271   -1.909750    1.515457
C       0.568387    0.318891   -1.177915
H       0.406675    0.492072   -3.304812
C      -2.000924   -0.553695   -1.161801
C      -0.101004    0.222271   -2.383579
H       1.602454   -2.545605    5.364656
H       4.017538   -2.850184    5.412175
O       5.750237   -1.132690    4.197139
H       4.491520    0.944508    3.483485
H       3.631417   -0.135583    2.335318
H       3.385208    0.785542    0.630968
H       6.121158   -3.180856    4.394340
H       7.488306   -2.054344    4.654267
H       6.227856   -2.224654    5.913881
C       6.427931   -2.227839    4.837460
C      -0.722600   -1.283231    5.078167
C      -1.572772   -2.346700    4.757749
C      -2.365368   -2.935814    5.733254
C      -2.305027   -2.431084    7.022777
```

```
C      -1.474837   -1.378355    7.376915
C      -0.669358   -0.817223    6.396520
H      -1.596613   -2.742708    3.745785
H      -3.016630   -3.775018    5.504956
F      -3.080486   -2.995153    7.977019
H      -1.457652   -1.017307    8.401689
H       1.600930    0.652827   -1.133238
H      -1.982490   -0.312880   -3.298574
C      -1.636561   -0.023254    2.548578

Frequencies (cm-1)
 -732.48    -37.06    -24.79     -9.76    100.00    100.00    100.00    100.00    100.00    110.49
  117.72    146.24    147.82    166.39    175.11    189.63    202.90    209.35    226.10    241.55
  243.28    281.66    282.51    302.76    338.81    342.23    382.51    391.17    400.72    402.27
  408.07    410.88    423.51    448.07    465.41    478.54    507.68    525.47    538.13    562.33
  581.51    584.81    615.60    627.87    640.91    641.80    670.70    681.90    701.29    730.97
  737.95    741.56    746.84    776.07    778.09    798.06    800.81    815.12    825.46    860.17
  874.97    878.79    902.52    936.43    948.00    949.86    950.64    956.57    981.34    994.39
 1001.03   1009.89   1013.22   1023.42   1067.63   1083.82   1095.27   1108.44   1112.02   1117.08
 1146.59   1151.87   1154.34   1160.40   1184.90   1207.31   1212.76   1216.33   1245.23   1261.86
 1274.65   1297.69   1299.76   1312.36   1321.24   1328.17   1341.69   1346.06   1352.98   1362.28
 1392.91   1397.33   1409.94   1423.92   1427.10   1431.20   1432.17   1438.67   1475.55   1479.11
 1492.03   1524.12   1557.17   1573.26   1581.71   1593.49   1600.64   1611.82   1616.38   2991.48
 2994.44   3001.86   3070.01   3083.28   3094.74   3099.69   3138.04   3142.72   3151.04   3152.82
 3155.00   3164.71   3167.64   3172.05   3173.65   3180.83   3194.08   3747.63
Note: any frequencies below 100 cm-1 (including spurious imaginary ones) are upscaled to 100 cm-1
      for the calculation of thermodynamic properties.
      (Averkiev, Truhlar, Catal. Sci. Technol. 2011, 1, 1526)

Thermodynamics

Note: this script does not take into account the spin entropy
For more info, see eq. 3 of Inorg. Chem. 2002, 41, 6928-6935
(M. Reiher), https://doi.org/10.1021/ic025891l

Temperature is now: 298.150

Reporting max. of 0 frequencies (set by $GETFREQSMAX env. variable)


Reading 1 outputfiles

            ScnFrq(1)
--------------------
--------------------
(ZPVE)        227.080
(dH,0->T)      16.696
(-TS)         -49.808
(dGibbs)      193.968
====================

Corresponding output files
 1 : frq_ts_FOMe-gamma-CD-pA_1_2.1281282.out


System ts_FOMe-gamma-mixOB-pA_1_2

Reading 1 outputfiles

       Pauli        Elstat        OrbInt       Disp.       Solv.         TOTAL          Erel   Symm.      <S2>
-----------------------------------------------------------------------------------------------------------------
-----
   28146.695     -5979.038    -29038.072     -27.239     -48.759     -6946.580         0.000   NOSYM     0.763
ADF(1)

Corresponding output files
 1 : ts_FOMe-gamma-mixOB-pA_1_2.1285463.out

Coordinates (Angs)
  48
ts_FOMe-ipsgamma-AF-pA_1_2.1285463.out        -6946.5838
C      -0.518069   -3.799519   -0.055469
C      -0.166032   -2.982460    1.019677
C      -1.034949   -2.015882    1.491525
C      -2.295448   -1.814101    0.842557
C      -2.658309   -2.679725   -0.209915
C      -1.764865   -3.635878   -0.685029
C      -0.588927   -1.015817    2.496470
N       0.000000   -0.000000    1.947288
C       0.700409    1.073428    2.631761
C       2.569270    0.793367    0.963443
N       1.941312   -0.000000    0.076299
C       2.617753   -0.889571   -0.663243
C       3.986606   -1.042348   -0.527832
C       4.657334   -0.246751    0.393568
C       3.944014    0.682613    1.139216
Cu      0.000000    0.000000    0.000000
O       0.012354   -0.025652   -1.809435
H      -0.855321    0.815589    4.582626
O      -1.796871   -0.140707   -0.066972
H       2.029775   -1.469132   -1.369544
H       4.506170   -1.777109   -1.135355
H       5.731507   -0.346855    0.528657
H       4.441426    1.328649    1.856898
H      -1.006546   -3.391858    3.609546
```

```
H      -1.468988   -3.906785    5.985804
H      -0.922435   -0.215834   -2.002721
H      -1.076328   -6.137180   -1.523520
H      -1.827589   -5.806350   -3.110669
H      -0.359167   -4.912623   -2.625046
H      -3.074708   -1.267459    1.366474
H      -3.631602   -2.575161   -0.681601
H       0.813329   -3.102015    1.477767
H       0.187348   -4.549026   -0.397544
O      -2.176005   -4.368992   -1.740329
F      -1.656007   -2.118482    7.854336
C       1.708671    1.759097    1.708144
H      -1.321919    0.278115    6.932062
C      -1.293764   -5.362980   -2.269021
C      -0.843983   -1.254123    3.912388
C      -1.055506   -2.582268    4.331239
C      -1.316810   -2.883196    5.655298
C      -1.393503   -1.837599    6.564123
C      -1.226355   -0.511208    6.191727
C      -0.946263   -0.225090    4.867297
H       1.209185    0.664461    3.512917
H      -0.020168    1.829004    2.963469
H       2.324228    2.430015    2.311616
H       1.171286    2.382091    0.981176

Frequencies (cm-1)
 -320.50    -45.22    -11.96    100.00    100.00    100.00    100.00    100.00    100.00    100.00
  124.54    135.97    147.11    158.45    174.69    208.27    213.97    215.33    230.69    239.43
  274.25    280.15    286.76    307.55    320.76    333.17    368.96    379.05    396.51    400.41
  415.18    419.73    433.72    461.00    473.12    488.97    497.95    510.37    547.12    553.75
  579.01    589.98    596.15    606.12    622.61    653.57    675.62    682.83    722.49    742.97
  744.68    756.67    781.18    787.40    790.43    797.96    824.80    828.69    830.12    862.99
  876.12    909.89    917.83    928.57    942.12    945.89    951.29    956.19    972.60    990.47
  995.49    999.35   1019.07   1025.24   1029.42   1048.13   1064.32   1092.95   1110.29   1113.04
 1121.75   1151.10   1151.35   1154.15   1163.28   1178.70   1183.34   1209.29   1226.19   1232.57
 1255.10   1277.73   1283.52   1294.17   1305.56   1319.76   1327.64   1344.99   1347.44   1373.64
 1391.80   1410.67   1414.10   1421.23   1425.06   1427.98   1436.67   1438.85   1473.78   1480.57
 1496.46   1529.50   1578.51   1580.63   1585.48   1592.79   1615.63   1619.82   2974.64   3000.05
 3010.83   3068.90   3070.43   3105.47   3127.58   3147.09   3148.82   3157.07   3165.90   3166.88
 3168.57   3173.38   3174.36   3179.81   3183.81   3186.37   3205.76   3675.31
Note: any frequencies below 100 cm-1 (including spurious imaginary ones) are upscaled to 100 cm-1
      for the calculation of thermodynamic properties.
      (Averkiev, Truhlar, Catal. Sci. Technol. 2011, 1, 1526)

Thermodynamics

Note: this script does not take into account the spin entropy
For more info, see eq. 3 of Inorg. Chem. 2002, 41, 6928-6935
(M. Reiher), https://doi.org/10.1021/ic025891l

Temperature is now: 298.150

Reporting max. of 0 frequencies (set by $GETFREQSMAX env. variable)


Reading 1 outputfiles

            ScnFrq(1)
--------------------
--------------------
(ZPVE)        229.122
(dH,0->T)      16.668
(-TS)         -49.687
(dGibbs)      196.103
====================

Corresponding output files
 1 : frq_ts_FOMe-gamma-mixOB-pA_1_2.1285894.out


System ts_FOMe-ipso-AO-pA_1_2

Reading 1 outputfiles

       Pauli        Elstat        OrbInt       Disp.       Solv.         TOTAL          Erel   Symm.      <S2>
------------------------------------------------------------------------------------------------------------------
-----
   28088.418     -5946.482    -29010.419     -26.183     -47.200     -6942.035         0.000   NOSYM     0.760
ADF(1)

Corresponding output files
 1 : ts_FOMe-ipso-AO-pA_1_2.1281142.out

Coordinates (Angs)
  48
ts_FOMe-ipso-AF-pA_1_2.1281142.out        -6942.0538
C      -0.035469   -4.076633    0.654000
C       0.144973   -2.824628    1.217414
C      -0.832395   -2.237169    2.032651
C      -2.011241   -2.959237    2.285814
C      -2.215959   -4.195587    1.710036
C      -1.228819   -4.770346    0.889850
C      -0.575499   -0.938330    2.653215
N       0.000000   -0.000000    1.956509
C       0.665628    1.150616    2.563131
```

```
C       2.564525    0.796448    0.898716
N       1.950586   -0.000000    0.000656
C       2.649319   -0.887313   -0.722093
C       4.015729   -1.038639   -0.562994
C       4.664760   -0.244282    0.375585
C       3.934388    0.684244    1.106761
Cu      0.000000    0.000000    0.000000
O      -0.627778    0.080799   -1.786053
H      -1.975062    1.117219    3.741503
O      -1.757136    0.014373   -0.411343
H       2.084528   -1.485937   -1.433746
H       4.550280   -1.770346   -1.161190
H       5.735581   -0.344925    0.534661
H       4.417472    1.326817    1.837332
H      -0.049599   -2.612289    4.707415
H      -0.671306   -2.300983    7.101771
H      -0.810504    1.015503   -1.995804
H       0.394534   -6.816186    0.142028
H      -0.976644   -7.601719   -0.692911
H      -0.321405   -6.062010   -1.322446
H      -2.780092   -2.530906    2.924658
H      -3.135592   -4.747909    1.888538
H       1.099758   -2.324546    1.082335
H       0.760895   -4.514310    0.061203
O      -1.509924   -5.991055    0.398283
F      -2.001404   -0.271238    8.008480
C       1.688081    1.778743    1.607856
H      -2.619189    1.397110    6.124878
C      -0.532411   -6.644144   -0.417768
C      -0.939757   -0.752271    4.068829
C      -0.600055   -1.727146    5.016015
C      -0.942166   -1.564677    6.349994
C      -1.655771   -0.431987    6.712748
C      -2.038652    0.538594    5.798135
C      -1.665331    0.376250    4.473715
H       1.158688    0.826787    3.486875
H      -0.057865    1.929251    2.824266
H       2.297223    2.482031    2.181116
H       1.157183    2.368750    0.848266

Frequencies (cm-1)
 -636.54    -29.04    -15.52     -8.16    100.00    100.00    100.00    100.00    100.00    100.00
  100.00    101.84    116.65    135.08    151.09    164.09    187.17    198.73    208.34    212.68
  220.83    243.65    284.28    298.79    318.57    339.17    359.10    383.82    403.32    405.83
  413.29    417.22    431.80    477.20    492.61    500.69    505.24    513.92    545.36    563.74
  589.49    595.52    613.07    614.26    626.23    654.63    679.58    697.29    735.48    743.69
  749.07    759.36    779.95    789.90    798.53    799.69    823.90    826.15    832.53    858.01
  878.83    919.16    927.36    945.38    946.24    953.35    957.07    957.77    975.29    996.13
  997.49   1002.20   1019.22   1023.16   1027.35   1049.93   1063.46   1098.49   1110.29   1115.71
 1122.19   1146.03   1150.87   1163.63   1169.21   1181.85   1190.30   1211.21   1215.92   1232.82
 1273.31   1276.97   1286.69   1296.45   1310.64   1325.66   1332.55   1347.11   1361.26   1364.92
 1414.03   1415.05   1416.04   1423.30   1428.21   1433.03   1438.70   1443.40   1481.66   1490.94
 1506.81   1552.54   1570.81   1583.82   1595.84   1607.65   1618.42   1622.04   2977.23   3004.28
 3009.31   3072.29   3076.49   3100.58   3127.93   3145.38   3149.25   3154.91   3156.54   3157.93
 3164.07   3165.26   3173.57   3173.58   3174.24   3181.74   3184.01   3680.41
Note: any frequencies below 100 cm-1 (including spurious imaginary ones) are upscaled to 100 cm-1
      for the calculation of thermodynamic properties.
      (Averkiev, Truhlar, Catal. Sci. Technol. 2011, 1, 1526)

Thermodynamics

Note: this script does not take into account the spin entropy
For more info, see eq. 3 of Inorg. Chem. 2002, 41, 6928-6935
(M. Reiher), https://doi.org/10.1021/ic025891l

Temperature is now: 298.150

Reporting max. of 0 frequencies (set by $GETFREQSMAX env. variable)


Reading 1 outputfiles

            ScnFrq(1)
--------------------
--------------------
(ZPVE)        229.096
(dH,0->T)      16.969
(-TS)         -50.801
(dGibbs)      195.265
====================

Corresponding output files
 1 : frq_ts_FOMe-ipso-AO-pA_1_2.1281284.out


System ts_FOMe-ipso-FG-pA_1_2

Reading 1 outputfiles

       Pauli        Elstat        OrbInt       Disp.       Solv.         TOTAL          Erel   Symm.      <S2>
-------------------------------------------------------------------------------------------------------------
-----
   28271.633     -5985.009    -29178.385     -27.636     -53.907     -6973.478         0.000   NOSYM     0.753
ADF(1)
```

```
Corresponding output files
 1 : ts_FOMe-ipso-FG-pA_1_2.1281144.out

Coordinates (Angs)
   48
ts_FOMe-ipso-FG-pA_1_2.1281144.out        -6973.4613
C      -2.341291   -3.204456    2.302184
C      -1.744310   -2.295887    1.469281
C      -2.240954   -0.933460    1.309882
C      -3.498460   -0.641414    1.978302
C      -4.097483   -1.550151    2.790766
C      -3.511678   -2.831829    2.994296
C      -0.978407   -0.513100    2.660242
N       0.000000    0.000000    2.061898
C       1.117814    0.593940    2.809465
C       2.747216    0.582320    0.893619
N       2.003404    0.000000   -0.068133
C       2.596701   -0.752866   -1.007546
C       3.963362   -0.977628   -1.020499
C       4.741060   -0.399609   -0.024763
C       4.125520    0.390982    0.936383
Cu      0.000000    0.000000    0.000000
O      -0.163786    0.023742   -1.875977
H      -2.333516    1.198135    4.196407
O      -1.918406   -0.209324    0.261992
H       1.932610   -1.168584   -1.760660
H       4.400232   -1.597451   -1.798124
H       5.816500   -0.559235    0.002751
H       4.705263    0.866085    1.723189
H      -0.233944   -2.566748    4.260522
H      -0.731268   -2.790645    6.693497
H      -1.108445   -0.117156   -2.037766
H      -2.618957   -4.847184    4.561685
H      -4.312111   -5.331698    4.890461
H      -3.633646   -5.541452    3.246811
H      -3.935669    0.339022    1.804992
H      -5.029460   -1.325952    3.303584
H      -0.854116   -2.568331    0.905189
H      -1.911124   -4.192567    2.420966
O      -4.142899   -3.606230    3.855221
F      -2.047174   -1.041518    8.080154
C       2.028442    1.412796    1.908792
H      -2.785774    0.978808    6.634685
C      -3.629930   -4.920638    4.147453
C      -1.251328   -0.669468    4.072011
C      -0.798923   -1.794396    4.774476
C      -1.067636   -1.924568    6.130109
C      -1.780504   -0.918076    6.762330
C      -2.233318    0.213436    6.096664
C      -1.973943    0.329104    4.741621
H       1.677726   -0.224841    3.278635
H       0.717921    1.223778    3.611017
H       2.755853    1.925319    2.544161
H       1.438191    2.187381    1.402436

Frequencies (cm-1)
  -205.59    -30.60    -14.94    100.00    100.00    100.00    100.00    100.00    100.00    102.93
   122.81    132.73    138.76    158.64    166.88    171.82    178.88    196.77    200.74    221.81
   232.10    249.31    263.21    292.63    314.08    329.99    343.28    378.07    387.12    397.43
   402.13    406.30    417.57    420.00    442.34    471.85    488.40    495.74    515.42    529.06
   541.05    570.92    587.21    613.69    625.42    638.18    647.21    684.96    726.41    739.80
   744.37    758.18    765.15    779.56    780.32    797.82    812.94    816.56    825.44    827.83
   832.98    879.04    885.22    936.69    944.63    948.32    954.18    963.63    967.36    972.61
   990.36    995.41    995.79   1003.49   1016.61   1056.28   1066.31   1093.51   1096.96   1105.62
  1115.43   1147.25   1149.47   1152.54   1162.79   1165.70   1204.06   1217.79   1219.85   1238.14
  1256.32   1273.68   1276.28   1296.34   1321.46   1322.78   1326.83   1332.00   1342.46   1350.88
  1400.04   1405.06   1411.96   1412.41   1429.65   1434.74   1434.98   1469.47   1478.57   1483.93
  1498.19   1506.85   1583.99   1588.73   1604.36   1615.44   1617.20   1706.47   2996.65   3006.91
  3014.46   3075.83   3096.79   3102.52   3141.65   3150.16   3150.98   3153.71   3155.42   3160.51
  3160.73   3167.72   3170.46   3175.08   3175.47   3177.25   3188.84   3748.99
Note: any frequencies below 100 cm-1 (including spurious imaginary ones) are upscaled to 100 cm-1
      for the calculation of thermodynamic properties.
      (Averkiev, Truhlar, Catal. Sci. Technol. 2011, 1, 1526)

Thermodynamics

Note: this script does not take into account the spin entropy
For more info, see eq. 3 of Inorg. Chem. 2002, 41, 6928-6935
(M. Reiher), https://doi.org/10.1021/ic025891l

Temperature is now: 298.150

Reporting max. of 0 frequencies (set by $GETFREQSMAX env. variable)


Reading 1 outputfiles

            ScnFrq(1)
---------------------
---------------------
(ZPVE)        228.133
(dH,0->T)      17.125
(-TS)         -50.659
(dGibbs)      194.599
=====================
```

```
Corresponding output files
 1 : frq_ts_FOMe-ipso-FG-pA_1_2.1282184.out


System ts_FOMe-ipso-GH-pA_1_2

Reading 1 outputfiles

       Pauli        Elstat        OrbInt       Disp.       Solv.         TOTAL          Erel   Symm.      <S2>
-----------------------------------------------------------------------------------------------------------------
-----
   28453.956     -6059.411    -29312.928     -26.791     -43.548     -6988.892         0.000   NOSYM     0.752
ADF(1)

Corresponding output files
 1 : ts_FOMe-ipso-GH-pA_1_2.1281148.out

Coordinates (Angs)
  48
ts_FOMe-ipso-GH-pA_1_2.1281148.out        -6988.8966
C      -3.003313   -2.100804    2.209345
C      -2.078710   -1.152986    1.597977
C      -2.652027   -0.092532    0.729278
C      -4.064401    0.004943    0.618733
C      -4.864658   -0.927288    1.212840
C      -4.342724   -2.013796    1.983481
C      -1.003496   -0.609726    2.517674
N       0.000000    0.000000    2.001591
C       1.066595    0.651189    2.740867
C       2.658638    0.674648    0.774696
N       1.930051   -0.000000   -0.142363
C       2.538053   -0.759250   -1.067822
C       3.915977   -0.873051   -1.133866
C       4.683119   -0.189371   -0.198964
C       4.047258    0.587371    0.760676
Cu      0.000000    0.000000    0.000000
O      -0.562264   -1.853704   -0.573653
H      -3.081916    0.326991    3.927479
O      -1.869405    0.629651    0.034793
H       1.882213   -1.288592   -1.753824
H       4.368642   -1.494780   -1.900521
H       5.768021   -0.262590   -0.212146
H       4.620516    1.132419    1.505391
H       0.590692   -1.888474    4.335483
H       0.183657   -2.094326    6.781412
H      -1.237848   -1.822992   -1.267487
H       1.693402   -0.105658    3.226515
H       0.646893    1.287765    3.529597
H       2.651415    2.048309    2.396654
H      -4.481466    0.808988    0.017735
H      -5.945695   -0.866066    1.098557
H      -1.450205   -1.710325    0.703178
H      -2.577959   -2.872110    2.842093
H      -5.735873   -4.477702    3.580371
F      -1.860143   -1.082361    8.014400
C       1.926279    1.490482    1.798426
H      -3.451046    0.154819    6.382257
H       1.288802    2.228532    1.293214
C      -1.209408   -0.750888    3.973790
C      -0.289360   -1.428158    4.777073
C      -0.510027   -1.554741    6.142517
C      -1.645709   -0.973138    6.683307
C      -2.575223   -0.287460    5.915245
C      -2.358123   -0.195282    4.548959
O      -5.286753   -2.860166    2.460149
H      -4.147374   -4.584975    2.763136
C      -4.839334   -3.923254    3.300013
H      -4.349650   -3.532022    4.201623

Frequencies (cm-1)
 -377.04    -26.06    -14.19    100.00    100.00    100.00    100.00    100.00    103.28    110.65
  122.11    140.52    156.03    166.01    179.18    182.79    196.29    222.23    222.70    246.84
  266.80    288.00    306.63    322.42    342.54    355.39    363.19    385.50    400.11    402.46
  408.49    413.67    428.70    463.31    489.12    497.08    509.34    513.86    526.98    542.34
  577.19    603.81    614.54    620.82    629.69    650.81    660.71    691.64    729.26    736.30
  743.49    754.32    774.85    780.48    796.47    803.21    810.46    824.01    834.96    836.09
  875.91    884.07    901.16    936.06    946.92    950.04    958.78    964.18    985.50    995.38
 1001.70   1015.51   1024.58   1027.67   1058.10   1064.86   1081.49   1098.77   1109.31   1121.83
 1126.04   1147.86   1150.74   1165.79   1167.78   1200.81   1209.84   1217.35   1227.06   1240.73
 1259.12   1277.71   1283.27   1297.00   1316.74   1319.95   1322.56   1335.69   1338.82   1361.38
 1403.55   1407.67   1410.34   1414.13   1425.81   1430.00   1435.39   1439.58   1476.25   1477.29
 1492.06   1540.64   1580.44   1582.08   1599.56   1609.06   1615.28   1633.84   1647.13   2963.46
 3001.78   3007.77   3052.80   3065.24   3098.11   3126.50   3145.25   3148.50   3153.30   3154.75
 3161.93   3167.04   3170.16   3172.67   3174.16   3180.55   3181.56   3749.82
Note: any frequencies below 100 cm-1 (including spurious imaginary ones) are upscaled to 100 cm-1
      for the calculation of thermodynamic properties.
      (Averkiev, Truhlar, Catal. Sci. Technol. 2011, 1, 1526)

Thermodynamics

Note: this script does not take into account the spin entropy
For more info, see eq. 3 of Inorg. Chem. 2002, 41, 6928-6935
(M. Reiher), https://doi.org/10.1021/ic025891l
```

```
Temperature is now: 298.150

Reporting max. of 0 frequencies (set by $GETFREQSMAX env. variable)


Reading 1 outputfiles

            ScnFrq(1)
---------------------
---------------------
(ZPVE)        227.318
(dH,0->T)      16.587
(-TS)         -49.497
(dGibbs)      194.409
=====================

Corresponding output files
 1 : frq_ts_FOMe-ipso-GH-pA_1_2.1282186.out


System ts_FOMe-ipso-OF-pA_1_2

Reading 1 outputfiles

       Pauli        Elstat        OrbInt       Disp.       Solv.         TOTAL          Erel   Symm.      <S2>
----------------------------------------------------------------------------------------------------------------
-----
   28144.407     -5973.112    -29050.052     -26.162     -47.600     -6952.687         0.000   NOSYM     0.759
ADF(1)

Corresponding output files
 1 : ts_FOMe-ipso-OF-pA_1_2.1285645.out

Coordinates (Angs)
  48
ts_FOMe-ipso-preFF-pA_1_2.1285645.out        -6952.6872
C      -1.683916   -3.673327    0.505174
C      -1.052917   -2.594588    1.073367
C      -1.785733   -1.527750    1.669141
C      -3.195355   -1.681765    1.794601
C      -3.831858   -2.739040    1.204253
C      -3.088236   -3.740279    0.533974
C      -1.023812   -0.570587    2.499667
N       0.000000    0.000000    1.945446
C       1.031989    0.717654    2.688840
C       2.679176    0.691519    0.801919
N       1.947312    0.000000   -0.090791
C       2.538826   -0.792754   -0.997416
C       3.913313   -0.946025   -1.038720
C       4.688929   -0.249765   -0.119836
C       4.065727    0.579160    0.802638
Cu      0.000000    0.000000    0.000000
O      -0.136784    0.035610   -1.811250
H      -1.557845    1.789110    3.737662
O      -1.765601   -0.193964    0.081646
H       1.875543   -1.295684   -1.695083
H       4.356119   -1.604256   -1.780059
H       5.771554   -0.350365   -0.121463
H       4.642724    1.144250    1.528969
H      -1.510062   -2.479195    4.336920
H      -2.269493   -2.147944    6.684718
H      -1.074062   -0.180037   -1.954243
H      -2.480092   -6.335934   -0.051164
H      -3.922096   -6.413516   -1.107583
H      -2.548259   -5.359219   -1.559324
H      -3.767883   -0.919506    2.316184
H      -4.913878   -2.835847    1.243378
H       0.034091   -2.560626    1.091521
H      -1.092971   -4.464282    0.056623
O      -3.804674   -4.719265   -0.016261
F      -2.743301    0.167153    7.749945
C       1.932545    1.538881    1.777606
H      -2.345314    2.100821    6.072134
C      -3.129669   -5.769348   -0.726735
C      -1.441467   -0.366009    3.892644
C      -1.676549   -1.474074    4.716570
C      -2.100490   -1.301070    6.025467
C      -2.319462   -0.009519    6.480729
C      -2.133499    1.109453    5.681200
C      -1.682616    0.924181    4.384294
H       2.638937    2.089353    2.403427
H       1.331003    2.281930    1.237257
H       1.628645   -0.029422    3.229275
H       0.589470    1.384447    3.430293

Frequencies (cm-1)
 -218.43    -34.18    -12.65    100.00    100.00    100.00    100.00    100.00    100.00    100.00
  121.52    137.55    156.45    158.90    174.86    192.59    200.87    208.41    212.00    239.20
  264.38    270.53    285.55    310.20    320.76    343.35    353.58    385.88    402.01    405.91
  409.08    412.51    418.90    436.10    467.56    483.11    502.59    513.91    539.49    569.64
  578.85    590.30    602.86    609.26    619.73    654.50    678.35    689.50    710.04    741.10
  745.18    753.69    775.93    782.34    786.71    797.36    815.63    821.39    828.01    861.43
  874.05    908.70    917.77    941.47    943.25    953.48    955.45    957.19    970.87    981.79
  996.14   1000.05   1005.59   1023.28   1029.03   1062.60   1073.13   1098.25   1111.51   1116.13
 1116.68   1145.92   1151.38   1162.71   1165.57   1170.95   1186.54   1213.42   1221.16   1235.97
```

```
 1276.22   1281.78   1294.56   1296.18   1308.00   1324.74   1328.95   1343.00   1346.75   1363.58
 1408.37   1410.50   1415.03   1415.75   1428.57   1435.53   1438.45   1466.46   1481.82   1483.55
 1491.90   1520.92   1576.37   1586.21   1594.85   1608.03   1615.99   1619.18   2989.42   3003.04
 3011.98   3090.27   3103.29   3106.39   3141.09   3145.25   3152.00   3153.34   3155.82   3157.89
 3167.54   3172.08   3173.84   3174.84   3176.47   3183.06   3188.80   3691.89
Note: any frequencies below 100 cm-1 (including spurious imaginary ones) are upscaled to 100 cm-1
      for the calculation of thermodynamic properties.
      (Averkiev, Truhlar, Catal. Sci. Technol. 2011, 1, 1526)

Thermodynamics

Note: this script does not take into account the spin entropy
For more info, see eq. 3 of Inorg. Chem. 2002, 41, 6928-6935
(M. Reiher), https://doi.org/10.1021/ic025891l

Temperature is now: 298.150

Reporting max. of 0 frequencies (set by $GETFREQSMAX env. variable)


Reading 1 outputfiles

            ScnFrq(1)
---------------------
---------------------
(ZPVE)        229.202
(dH,0->T)      16.747
(-TS)         -49.931
(dGibbs)      196.018
=====================

Corresponding output files
 1 : frq_ts_FOMe-ipso-OF-pA_1_2.1285895.out
```

long-FOMe-X-F

```
Please give the temperature (in Kelvin) at which the thermodynamics must be computed
298.15


System fwd_frq_ts_FOMe-gamma-AB-pA_1_2

Reading 1 outputfiles

       Pauli        Elstat        OrbInt       Disp.       Solv.         TOTAL          Erel   Symm.      <S2>
------------------------------------------------------------------------------------------------------------------
-----
   28312.068     -6042.318    -29163.566     -26.737     -46.741     -6967.467         0.000   NOSYM     0.760
ADF(1)

Corresponding output files
 1 : fwd_frq_ts_FOMe-gamma-AB-pA_1_2.1293354.out

Coordinates (Angs)
  48
fwd_frq_ts_FOMe-gamBa-AB-pA_1_2.1293354.out        -6967.4818
C       0.000000    0.000000    4.946679
C       0.889638   -0.526287    4.009852
C       1.083492    0.081123    2.784103
C       0.343336    1.343604    2.418632
C      -0.542472    1.865958    3.491252
C      -0.687880    1.191946    4.660073
C       2.015943   -0.410648    1.802566
N       1.835214    0.000000    0.560781
C       2.877102    0.003222   -0.455154
C       1.543051   -1.520786   -1.883909
N       0.417302   -1.520923   -1.146912
C      -0.358395   -2.612084   -1.068416
C      -0.013821   -3.785529   -1.717586
C       1.152969   -3.814853   -2.472163
C       1.933055   -2.669541   -2.562495
Cu      0.000000    0.000000    0.000000
O      -1.748499   -0.056978   -0.533666
H       3.847995   -0.144253    3.821039
O      -0.490010    1.234985    1.276191
H      -1.260792   -2.516764   -0.472247
H      -0.654208   -4.657006   -1.621936
H       1.454236   -4.723390   -2.988007
H       2.846267   -2.654060   -3.150576
H       3.671976   -0.709204   -0.222496
H       1.689593    0.575807   -2.169334
H      -2.197686    0.509787    0.114311
H       2.593757   -2.756461    0.638859
H       4.335587   -4.358612    1.240807
H       7.195234   -5.644938    3.066103
H       1.132275    2.103668    2.228729
H      -1.107326    2.769760    3.282010
H       1.416429   -1.446520    4.247721
H      -0.176833   -0.501582    5.893954
F      -1.531970    1.661281    5.604155
H       3.168712   -0.307723   -2.555371
C       2.331635   -0.257862   -1.855842
H       3.312939    1.011024   -0.438742
H       5.635248   -1.747952    4.415229
C       3.100223   -1.327610    2.175817
C       3.268308   -2.519846    1.459490
C       4.252391   -3.431983    1.798907
C       5.117159   -3.148746    2.865115
C       4.958908   -1.956049    3.589539
C       3.958354   -1.066161    3.254673
O       6.116489   -3.953916    3.270798
H       5.473280   -5.838139    2.628134
C       6.346046   -5.177526    2.565766
H       6.596777   -4.985036    1.515976

Frequencies (cm-1)
  100.00    100.00    100.00    100.00    100.00    100.00    100.00    100.00    100.00    100.00
  115.18    135.43    148.41    159.95    164.50    176.22    191.89    209.01    218.49    238.39
  249.18    272.51    292.35    304.72    322.53    329.74    335.41    372.88    392.07    403.93
  411.46    421.87    433.94    449.85    473.76    496.56    507.86    509.65    553.56    562.38
  592.74    611.77    613.23    617.17    624.04    658.01    671.05    691.24    726.99    742.14
  746.76    751.97    785.10    792.29    795.54    798.90    817.54    825.08    827.04    846.33
  876.58    913.42    923.07    938.51    943.07    951.04    952.52    954.63    963.53    995.48
  996.62   1001.52   1013.81   1019.51   1026.94   1050.72   1062.64   1101.27   1113.13   1116.75
 1121.43   1147.28   1151.91   1163.49   1168.00   1178.37   1185.42   1215.88   1221.42   1225.59
 1276.68   1277.52   1280.60   1287.47   1292.57   1315.12   1320.80   1343.72   1359.82   1367.45
 1414.73   1416.42   1418.06   1428.81   1431.46   1434.56   1439.09   1445.15   1475.55   1491.50
 1502.28   1552.31   1564.77   1582.45   1591.47   1605.79   1613.77   1620.88   2979.15   3014.25
 3033.43   3075.72   3100.19   3114.51   3129.98   3151.08   3153.59   3156.06   3158.15   3165.73
 3166.11   3167.85   3172.10   3174.16   3175.44   3185.70   3187.38   3705.24
Note: any frequencies below 100 cm-1 (including spurious imaginary ones) are upscaled to 100 cm-1
      for the calculation of thermodynamic properties.
      (Averkiev, Truhlar, Catal. Sci. Technol. 2011, 1, 1526)

Thermodynamics

Note: this script does not take into account the spin entropy
For more info, see eq. 3 of Inorg. Chem. 2002, 41, 6928-6935
(M. Reiher), https://doi.org/10.1021/ic025891l
```

```
Temperature is now: 298.150

Reporting max. of 0 frequencies (set by $GETFREQSMAX env. variable)


Reading 1 outputfiles

            ScnFrq(1)
---------------------
---------------------
(ZPVE)        229.302
(dH,0->T)      16.778
(-TS)         -50.006
(dGibbs)      196.074
=====================

Corresponding output files
 1 : frq_fwd_frq_ts_FOMe-gamma-AB-pA_1_2.1283958.out


System fwd_frq_ts_FOMe-gamma-BC-pA_1_2

Reading 1 outputfiles

       Pauli        Elstat        OrbInt       Disp.       Solv.         TOTAL          Erel   Symm.      <S2>
-----------------------------------------------------------------------------------------------------------------
-----
   28427.879     -6034.929    -29326.361     -24.657     -49.104     -7007.348         0.000   NOSYM     0.753
ADF(1)

Corresponding output files
 1 : fwd_frq_ts_FOMe-gamma-BC-pA_1_2.1304158.out

Coordinates (Angs)
   48
fwd_frq_ts_FOMe-gamBa-BC-pA_1_2.1304158.out        -7007.3444
C       0.000000    0.000000    5.501923
C      -0.979824    0.121974    4.490297
C      -0.800362   -0.215353    3.159061
C       0.487321   -0.721649    2.759505
C       1.503948   -1.036224    3.821260
C       1.182339   -0.563009    5.171842
C      -1.920781   -0.108345    2.224899
N      -1.768697   -0.052113    0.935932
C      -2.973942   -0.041109    0.097821
C      -1.942244    0.657141   -2.088382
N      -0.723541    0.969430   -1.613010
C       0.012236    1.916025   -2.211770
C      -0.445720    2.609164   -3.320512
C      -1.705133    2.303238   -3.822589
C      -2.459213    1.317504   -3.199889
Cu      0.000000    0.000000    0.000000
O       1.717899   -0.000000   -0.773912
H      -3.116566   -2.276256    3.242069
O       0.824404   -0.953320    1.580991
H       0.994459    2.091500   -1.782264
H       0.180767    3.369634   -3.777806
H      -2.096569    2.825640   -4.692611
H      -3.445977    1.050752   -3.568802
H      -3.428432    0.956617    0.144000
H      -2.143078   -1.351039   -1.400154
H       2.287836   -0.462824   -0.142587
H      -3.701725    1.949466    2.609886
H      -7.586466    3.116109    4.350019
H      -6.753574   -0.398608    4.536769
H       1.622053   -2.132446    3.850056
H       2.483618   -0.656411    3.502044
H      -1.941948    0.530741    4.788754
H      -0.203436    0.328769    6.515663
F       2.138752   -0.738066    6.077429
H      -3.657819   -0.573263   -1.847708
C      -2.696913   -0.403243   -1.354588
H      -3.708278   -0.744832    0.504318
H      -5.363735   -2.408571    4.286903
C      -3.275728   -0.157197    2.848122
C      -4.055521    0.985913    2.970317
C      -5.309938    0.911937    3.592439
C      -5.781918   -0.318134    4.060466
C      -4.991436   -1.454787    3.919514
C      -3.735443   -1.387161    3.332538
O      -5.983604    2.082352    3.694461
C      -7.245702    2.079466    4.364183
H      -7.973521    1.447797    3.840061
H      -7.141551    1.741888    5.402814

Frequencies (cm-1)
   100.00    100.00    100.00    100.00    100.00    100.00    100.00    100.00    100.00    115.64
   134.67    143.87    162.36    169.60    201.25    209.55    219.40    233.09    251.56    261.77
   271.59    292.53    313.87    317.79    334.76    349.19    350.80    374.20    378.08    404.94
   408.27    422.58    435.37    469.70    497.49    503.93    516.36    537.34    548.24    556.92
   570.08    577.28    610.38    615.90    648.02    653.11    692.17    720.69    736.68    742.68
   747.90    759.53    783.19    794.56    800.13    805.29    815.09    822.77    858.89    865.26
   898.14    928.34    935.12    938.46    943.94    950.21    955.85    959.23    994.02    995.87
  1003.24   1020.45   1023.05   1026.63   1060.31   1064.39   1076.51   1111.16   1112.11   1122.42
```

```
 1123.31   1149.63   1161.03   1163.69   1166.28   1167.62   1181.07   1219.96   1226.89   1231.95
 1263.32   1268.37   1285.44   1291.07   1294.05   1321.20   1324.58   1334.65   1344.60   1356.23
 1372.73   1399.93   1409.53   1412.48   1413.87   1427.42   1430.13   1437.80   1441.00   1479.97
 1488.04   1507.87   1512.46   1570.02   1574.78   1586.46   1613.93   1614.79   2862.25   2975.51
 3008.28   3009.53   3070.10   3071.91   3103.20   3127.57   3144.50   3150.59   3154.89   3158.52
 3161.12   3172.02   3176.45   3177.76   3179.92   3185.89   3188.38   3705.40
Note: any frequencies below 100 cm-1 (including spurious imaginary ones) are upscaled to 100 cm-1
      for the calculation of thermodynamic properties.
      (Averkiev, Truhlar, Catal. Sci. Technol. 2011, 1, 1526)

Thermodynamics

Note: this script does not take into account the spin entropy
For more info, see eq. 3 of Inorg. Chem. 2002, 41, 6928-6935
(M. Reiher), https://doi.org/10.1021/ic025891l

Temperature is now: 298.150

Reporting max. of 0 frequencies (set by $GETFREQSMAX env. variable)


Reading 1 outputfiles

            ScnFrq(1)
---------------------
---------------------
(ZPVE)        229.271
(dH,0->T)      16.383
(-TS)         -49.087
(dGibbs)      196.568
=====================

Corresponding output files
 1 : frq_fwd_frq_ts_FOMe-gamma-BC-pA_1_2.1285640.out


System fwd_frq_ts_FOMe-gamma-CD-pA_1_2

Reading 1 outputfiles

       Pauli        Elstat        OrbInt       Disp.       Solv.         TOTAL          Erel   Symm.      <S2>
-----------------------------------------------------------------------------------------------------------------
-----
   28627.262     -6067.258    -29521.949     -25.225     -45.613     -7032.958         0.000   NOSYM     0.752
ADF(1)

Corresponding output files
 1 : fwd_frq_ts_FOMe-gamma-CD-pA_1_2.1285048.out

Coordinates (Angs)
  48
fwd_frq_ts_FOMe-gamBa-CD-pA_1_2.1285048.out        -7032.9610
C       0.000000   -0.000000    4.650155
C       0.052366    0.116587    3.243287
C       1.140406   -0.517944    2.549877
C       2.120077   -1.194151    3.310246
C       1.999360   -1.264107    4.675678
C       0.947820   -0.680925    5.381188
C      -0.970393    0.888872    2.571077
H      -0.684462    3.288854    3.843020
O       1.303954   -0.512361    1.256148
Cu      0.000000    0.000000    0.000000
O       1.659286    0.000000   -1.396953
C      -3.307458   -1.143292   -3.206121
C      -3.117762    0.968469   -0.085230
H      -2.522659    2.495851    1.286508
H      -1.512354    2.402405   -0.157862
N      -1.407010   -0.278154   -1.375048
H      -4.684623    0.012953   -2.021367
C      -2.692249    0.105922   -1.237393
N      -1.059517    0.976185    1.272405
H      -3.897296    1.648569   -0.445166
H      -3.601858    0.325467    0.663414
C      -1.064709   -1.075939   -2.408059
H      -1.647792   -2.175949   -4.147050
C      -3.657256   -0.316801   -2.152521
C      -1.978766   -1.531470   -3.338177
H      -0.826190    0.464589    5.178830
H       0.891735   -0.766396    6.462055
F       2.945682   -1.937645    5.372959
H       2.950272   -1.671557    2.797398
H       2.460754    0.011928   -0.850521
H       1.737776    0.767900   -1.982459
O      -4.749186    3.699724    5.674596
H      -6.607532    3.047817    4.969303
C      -6.058207    3.180837    5.909635
H      -6.015821    2.228264    6.452242
C      -1.970776    1.612171    3.405272
C      -3.235787    1.063186    3.601644
C      -4.195395    1.724205    4.360353
C      -3.891775    2.965667    4.926596
C      -2.619745    3.522829    4.731729
C      -1.669607    2.848939    3.984735
H      -3.479324    0.095172    3.168301
H      -5.167781    1.264129    4.501917
```

```
H      -6.562999    3.928264    6.524002
H      -2.392713    4.488562    5.177958
H      -0.016255   -1.350785   -2.474536
H      -4.059411   -1.477216   -3.917197
C      -2.043924    1.801513    0.590791

Frequencies (cm-1)
  100.00    100.00    100.00    100.00    100.00    100.00    100.00    100.00    100.00    100.00
  116.50    129.95    143.80    148.72    158.30    170.42    191.92    200.55    215.60    226.39
  235.71    254.12    264.42    277.49    291.61    313.59    333.23    362.10    400.91    401.87
  406.93    411.98    425.68    446.11    452.13    458.73    504.94    517.25    536.88    548.09
  567.60    586.09    590.91    611.70    621.97    629.66    644.31    670.73    682.20    727.05
  730.06    737.52    742.88    749.33    777.11    790.02    797.73    801.52    819.67    824.21
  854.92    880.42    903.89    932.41    935.65    948.47    958.63    978.64    984.31    995.74
  998.55   1014.57   1021.90   1029.08   1067.94   1084.41   1106.30   1108.99   1112.74   1122.26
 1139.76   1153.13   1162.72   1168.06   1173.88   1203.49   1206.21   1239.36   1251.01   1259.22
 1275.04   1286.14   1299.04   1322.50   1326.07   1329.62   1351.16   1352.76   1380.83   1389.50
 1413.83   1419.49   1421.21   1429.39   1430.72   1435.65   1440.38   1470.16   1485.79   1500.98
 1531.46   1566.38   1577.84   1579.35   1582.17   1613.66   1618.91   1619.78   2970.59   2999.54
 3005.96   3062.34   3070.42   3080.30   3118.50   3139.26   3140.60   3150.99   3157.49   3163.76
 3168.60   3168.65   3173.45   3180.98   3181.45   3181.95   3710.08   3795.95
Note: any frequencies below 100 cm-1 (including spurious imaginary ones) are upscaled to 100 cm-1
      for the calculation of thermodynamic properties.
      (Averkiev, Truhlar, Catal. Sci. Technol. 2011, 1, 1526)

Thermodynamics

Note: this script does not take into account the spin entropy
For more info, see eq. 3 of Inorg. Chem. 2002, 41, 6928-6935
(M. Reiher), https://doi.org/10.1021/ic025891l

Temperature is now: 298.150

Reporting max. of 0 frequencies (set by $GETFREQSMAX env. variable)


Reading 1 outputfiles

            ScnFrq(1)
---------------------
---------------------
(ZPVE)        229.838
(dH,0->T)      17.023
(-TS)         -50.512
(dGibbs)      196.350
=====================

Corresponding output files
 1 : frq_fwd_frq_ts_FOMe-gamma-CD-pA_1_2.1285642.out


System fwd_frq_ts_FOMe-ipso-AO-pA_1_2

Reading 1 outputfiles

       Pauli        Elstat        OrbInt       Disp.       Solv.         TOTAL          Erel   Symm.      <S2>
-----------------------------------------------------------------------------------------------------------------
-----
   28159.158     -5967.566    -29070.890     -26.905     -47.048     -6953.418         0.000   NOSYM     0.760
ADF(1)

Corresponding output files
 1 : fwd_frq_ts_FOMe-ipso-AO-pA_1_2.1285280.out

Coordinates (Angs)
  48
rev_frq_ts_FOMe-ipsoBa-AF-pA_1_2.1285280.out        -6953.4114
C       0.000000   -0.000000    4.028677
C      -0.221636   -0.813771    2.928491
C       0.796962   -1.635962    2.429416
C       2.041642   -1.656802    3.069207
C       2.286272   -0.830187    4.154007
C       1.259709   -0.014088    4.608651
C       0.556640   -2.474601    1.243168
N       0.017882   -1.915254    0.191297
C      -0.570491   -2.641055   -0.928090
C      -2.497408   -0.984770   -0.838979
N      -1.928176   -0.051641   -0.052349
C      -2.665021    0.769257    0.707502
C      -4.044814    0.670055    0.735372
C      -4.658578   -0.302905   -0.045252
C      -3.879732   -1.131110   -0.842141
Cu      0.000000    0.000000    0.000000
O       0.013054    1.816875   -0.142929
H       1.774330   -3.991373   -0.675768
O       1.748448   -0.000000   -0.072386
H      -2.118774    1.508396    1.287108
H      -4.617109    1.345047    1.364135
H      -5.740213   -0.411861   -0.039088
H      -4.330873   -1.886768   -1.478672
H       0.223016   -4.124200    3.345713
H       0.886087   -6.498129    3.504464
H       0.923257    2.100362    0.046696
O       2.034376   -7.853946    1.702872
H       2.858698   -9.570460    1.036499
```

```
C       2.688846   -8.566468    0.646036
H       2.831197   -2.303024    2.694600
H       3.253916   -0.810513    4.647762
H      -1.211714   -0.849365    2.482707
H      -0.784959    0.627617    4.441622
F       1.492009    0.787005    5.669230
H       3.648432   -8.099645    0.396253
C      -1.573553   -1.780942   -1.699888
H       2.461779   -6.314043   -0.506455
H       2.050905   -8.617614   -0.243496
C       0.913241   -3.885354    1.306856
C       0.699467   -4.609754    2.497852
C       1.066624   -5.932734    2.593457
C       1.697693   -6.570904    1.509873
C       1.945685   -5.854161    0.329390
C       1.540584   -4.536798    0.233260
H      -1.059579   -3.538819   -0.533094
H       0.202180   -2.954193   -1.635591
H      -2.145575   -2.439402   -2.357571
H      -1.027871   -1.083846   -2.350410

Frequencies (cm-1)
  100.00    100.00    100.00    100.00    100.00    100.00    100.00    100.00    100.00    100.00
  105.48    127.63    141.59    153.78    155.76    171.10    195.71    207.98    219.56    221.26
  247.82    261.79    289.27    299.06    307.07    314.53    336.75    359.19    393.40    403.40
  409.69    416.74    419.76    435.26    468.06    497.85    502.58    511.99    554.41    561.41
  593.38    596.54    613.67    617.94    623.62    659.47    675.29    701.72    730.28    743.67
  747.08    753.66    781.51    790.64    799.47    801.86    819.31    827.33    828.73    858.96
  875.25    916.70    925.37    942.24    949.16    954.99    956.49    958.06    977.85    994.26
  994.97   1002.65   1016.53   1023.15   1029.33   1052.70   1065.01   1099.26   1113.28   1118.92
 1119.62   1146.55   1150.92   1162.95   1167.83   1181.83   1195.26   1212.25   1217.86   1232.23
 1275.42   1279.59   1287.17   1293.00   1311.27   1327.06   1331.65   1349.92   1362.20   1366.72
 1410.75   1413.35   1416.40   1420.83   1429.17   1435.07   1439.04   1448.87   1479.29   1492.35
 1502.96   1534.49   1559.58   1583.96   1595.89   1607.65   1615.06   1618.44   2980.79   3006.42
 3013.25   3078.85   3084.01   3105.43   3131.30   3154.15   3157.35   3157.74   3162.60   3165.44
 3168.01   3168.77   3174.56   3175.08   3176.03   3185.42   3188.43   3708.88
Note: any frequencies below 100 cm-1 (including spurious imaginary ones) are upscaled to 100 cm-1
      for the calculation of thermodynamic properties.
      (Averkiev, Truhlar, Catal. Sci. Technol. 2011, 1, 1526)

Thermodynamics

Note: this script does not take into account the spin entropy
For more info, see eq. 3 of Inorg. Chem. 2002, 41, 6928-6935
(M. Reiher), https://doi.org/10.1021/ic025891l

Temperature is now: 298.150

Reporting max. of 0 frequencies (set by $GETFREQSMAX env. variable)


Reading 1 outputfiles

            ScnFrq(1)
---------------------
---------------------
(ZPVE)        229.214
(dH,0->T)      16.900
(-TS)         -50.404
(dGibbs)      195.709
=====================

Corresponding output files
 1 : frq_fwd_frq_ts_FOMe-ipso-AO-pA_1_2.1285785.out


System fwd_frq_ts_FOMe-ipso-FG-pA_1_2

Reading 1 outputfiles

       Pauli        Elstat        OrbInt       Disp.       Solv.         TOTAL          Erel   Symm.      <S2>
-----------------------------------------------------------------------------------------------------------------
-----
   28374.819     -6020.101    -29277.630     -25.852     -48.860     -6997.801         0.000   NOSYM     0.753
ADF(1)

Corresponding output files
 1 : fwd_frq_ts_FOMe-ipso-FG-pA_1_2.1285284.out

Coordinates (Angs)
  48
rev_frq_ts_FOMe-ipsoBa-FG-pA_1_2.1285284.out        -6997.7782
C       0.000000   -0.000000    4.514452
C      -0.094239   -0.148973    3.041412
C       0.487281   -1.426831    2.460046
C       0.955553   -2.446045    3.348858
C       1.000914   -2.218283    4.686174
C       0.547315   -0.980702    5.245044
C      -1.496383    0.096134    2.462844
N      -1.602869    0.194943    1.184030
C      -2.924701    0.307006    0.558336
C      -2.117742    1.092096   -1.677022
N      -0.801993    1.195146   -1.412805
C      -0.063714    2.139951   -2.009105
C      -0.615859    3.045575   -2.900829
```

```
C      -1.974206    2.958359   -3.182177
C      -2.730537    1.969838   -2.566350
Cu      0.000000    0.000000    0.000000
O       1.682933    0.000000   -0.815698
H      -2.364942   -1.799840    4.209283
O       0.561038   -1.565421    1.221513
H       0.992623    2.144498   -1.753382
H       0.013163    3.801947   -3.361167
H      -2.442274    3.653790   -3.875100
H      -3.794154    1.870997   -2.766487
H      -3.138523    2.211434    2.842006
H      -5.017635    2.392926    4.388613
H       2.211616   -0.683293   -0.378193
O      -5.817372    0.365413    6.037181
H      -7.391821    1.287201    6.896044
C      -6.638823    1.530612    6.145016
H       1.306547   -3.377093    2.912899
H       1.389616   -2.972745    5.366972
H       0.530881    0.652717    2.606319
H      -0.362680    0.913231    4.976168
F       0.675984   -0.855398    6.582746
H      -7.130304    1.756114    5.190932
C      -2.872214    0.033226   -0.938974
H      -4.293389   -1.634978    5.740415
H      -6.053974    2.396558    6.477785
C      -2.627891    0.190004    3.394282
C      -3.392505    1.356562    3.463996
C      -4.457591    1.464768    4.346037
C      -4.790937    0.378730    5.162980
C      -4.025091   -0.796851    5.101604
C      -2.946855   -0.881086    4.243482
H      -3.322439    1.313991    0.721574
H      -3.612381   -0.397106    1.040772
H      -3.899471   -0.013681   -1.310140
H      -2.418095   -0.949649   -1.122997

Frequencies (cm-1)
  100.00    100.00    100.00    100.00    100.00    100.00    100.00    100.00    100.17    113.59
  119.55    137.81    150.56    159.59    175.74    191.96    204.01    207.22    222.75    231.80
  242.56    278.38    297.73    306.58    312.69    319.45    353.42    364.42    389.78    403.60
  407.52    415.90    434.76    455.01    471.90    495.24    504.11    511.57    524.23    530.01
  577.22    600.39    608.77    630.92    641.68    643.17    681.62    731.58    743.61    750.34
  758.95    770.83    779.27    785.27    796.02    811.90    816.29    822.26    824.56    846.80
  874.54    878.64    935.06    941.04    950.71    955.22    963.88    966.59    994.40    995.27
 1000.64   1014.79   1023.48   1051.04   1058.08   1067.76   1076.43   1096.51   1107.09   1116.51
 1122.59   1133.85   1148.60   1163.54   1165.11   1170.36   1181.02   1220.02   1223.17   1238.80
 1267.25   1271.26   1276.27   1291.26   1295.08   1308.29   1323.33   1332.42   1344.27   1356.65
 1407.08   1414.69   1415.39   1416.29   1427.64   1428.07   1436.14   1440.83   1477.51   1500.83
 1545.63   1572.69   1584.88   1586.27   1606.85   1614.63   1625.78   1671.70   2946.22   2974.14
 3007.99   3011.29   3068.24   3075.72   3096.94   3126.06   3142.11   3149.93   3150.58   3155.91
 3160.79   3162.20   3167.50   3175.90   3177.28   3177.68   3185.05   3752.35
Note: any frequencies below 100 cm-1 (including spurious imaginary ones) are upscaled to 100 cm-1
      for the calculation of thermodynamic properties.
     (Averkiev, Truhlar, Catal. Sci. Technol. 2011, 1, 1526)

Thermodynamics

Note: this script does not take into account the spin entropy
For more info, see eq. 3 of Inorg. Chem. 2002, 41, 6928-6935
(M. Reiher), https://doi.org/10.1021/ic025891l

Temperature is now: 298.150

Reporting max. of 0 frequencies (set by $GETFREQSMAX env. variable)


Reading 1 outputfiles

            ScnFrq(1)
--------------------
--------------------
(ZPVE)        229.293
(dH,0->T)      16.722
(-TS)         -49.890
(dGibbs)      196.126
====================

Corresponding output files
 1 : frq_fwd_frq_ts_FOMe-ipso-FG-pA_1_2.1285856.out


System fwd_frq_ts_FOMe-ipso-GH-pA_1_2

Reading 1 outputfiles

       Pauli        Elstat        OrbInt       Disp.       Solv.         TOTAL          Erel   Symm.      <S2>
-------------------------------------------------------------------------------------------------------------------
-----
   28584.440     -6060.287    -29484.371     -25.024     -45.847     -7031.262         0.000   NOSYM     0.752
ADF(1)

Corresponding output files
 1 : fwd_frq_ts_FOMe-ipso-GH-pA_1_2.1285287.out

Coordinates (Angs)
```

```
  48
fwd_frq_ts_FOMe-ipsoBa-GH-pA_1_2.1285287.out        -7031.2543
C       0.000000    0.000000    4.645045
C       0.039227    0.117923    3.235560
C      -1.180073    0.417791    2.545105
C      -2.354786    0.628634    3.308157
C      -2.363348    0.508441    4.679532
C      -1.171989    0.186435    5.327153
C       1.318743   -0.028932    2.573350
N       1.480468    0.000000    1.274954
C       2.834941   -0.063127    0.714585
C       2.235617   -0.881746   -1.577088
N       0.912198   -1.048590   -1.392879
C       0.259473   -2.029773   -2.037010
C       0.908448   -2.901391   -2.894588
C       2.275749   -2.744565   -3.091762
C       2.943242   -1.723301   -2.429326
Cu      0.000000    0.000000    0.000000
O      -1.419524    0.726200   -1.453771
H       2.758128    1.880338    3.891199
O      -1.295225    0.512549    1.247960
H      -0.808446   -2.106887   -1.848561
H       0.346766   -3.686574   -3.391777
H       2.818748   -3.413709   -3.755183
H       4.010905   -1.572615   -2.562717
H       2.569658   -2.350127    3.155872
H       4.598401   -2.690074    4.485439
H      -1.058832    1.206746   -2.213814
H       3.280294   -1.044620    0.918181
H       3.460661    0.681774    1.218221
H       3.924917    0.309610   -1.078183
H      -3.266674    0.867622    2.766619
H      -3.275478    0.654679    5.253620
H      -2.011584    1.353342   -1.009825
H       0.901621   -0.239108    5.199158
O       5.993528   -0.655439    5.665117
H       6.878235   -2.391804    4.905469
C       2.877102    0.207929   -0.784008
H       4.824907    1.548574    5.215949
H       2.385889    1.166004   -1.002542
C       2.527542   -0.214391    3.424022
C       3.061264   -1.488489    3.603579
C       4.214038   -1.683612    4.355903
C       4.858230   -0.583732    4.930346
C       4.321472    0.699972    4.757975
C       3.166594    0.879460    4.016690
F      -1.179836    0.059866    6.683938
H       7.489158   -1.750645    6.457803
C       6.593783   -1.935730    5.861922
H       5.922697   -2.609429    6.409125

Frequencies (cm-1)
  100.00    100.00    100.00    100.00    100.00    100.00    100.00    100.00    100.00    100.80
  110.99    126.00    132.31    141.77    156.25    170.28    186.27    194.20    224.79    226.85
  236.19    259.41    270.02    282.00    296.06    324.49    346.67    375.14    386.08    399.41
  401.34    408.57    415.64    431.35    449.88    461.78    489.30    499.84    527.07    530.77
  540.68    550.60    577.23    607.98    616.60    646.68    649.49    688.02    688.52    738.86
  742.66    751.78    758.91    780.20    785.25    796.37    807.86    811.68    820.70    847.53
  858.28    875.02    888.19    930.09    931.45    945.69    948.26    955.81    979.51    995.20
  999.52   1020.98   1026.15   1029.60   1060.35   1072.26   1102.84   1107.95   1109.94   1123.89
 1139.16   1150.55   1162.54   1167.56   1168.08   1184.45   1221.74   1232.23   1237.74   1258.93
 1269.21   1285.79   1297.71   1324.66   1329.77   1332.86   1344.08   1351.92   1386.69   1405.69
 1413.69   1415.08   1417.65   1419.78   1427.89   1438.52   1439.23   1448.05   1483.23   1500.02
 1519.02   1562.43   1570.89   1578.03   1585.69   1618.35   1619.40   1625.31   2969.58   3002.12
 3006.47   3061.23   3065.88   3097.04   3120.23   3138.87   3140.05   3152.39   3152.65   3157.52
 3158.15   3166.89   3171.15   3180.13   3180.58   3180.63   3710.49   3798.59
Note: any frequencies below 100 cm-1 (including spurious imaginary ones) are upscaled to 100 cm-1
      for the calculation of thermodynamic properties.
      (Averkiev, Truhlar, Catal. Sci. Technol. 2011, 1, 1526)

Thermodynamics

Note: this script does not take into account the spin entropy
For more info, see eq. 3 of Inorg. Chem. 2002, 41, 6928-6935
(M. Reiher), https://doi.org/10.1021/ic025891l

Temperature is now: 298.150

Reporting max. of 0 frequencies (set by $GETFREQSMAX env. variable)


Reading 1 outputfiles

            ScnFrq(1)
---------------------
---------------------
(ZPVE)        229.660
(dH,0->T)      17.055
(-TS)         -50.574
(dGibbs)      196.141
=====================

Corresponding output files
 1 : frq_fwd_frq_ts_FOMe-ipso-GH-pA_1_2.1286219.out
```

```
System fwd_frq_ts_FOMe-ipso-OF-pA_1_2

Reading 1 outputfiles

       Pauli        Elstat        OrbInt       Disp.       Solv.         TOTAL          Erel   Symm.      <S2>
--------------------------------------------------------------------------------------------------------------------
-----
   28277.260     -6015.532    -29159.822     -25.505     -46.878     -6970.646         0.000   NOSYM     0.758
ADF(1)

Corresponding output files
 1 : fwd_frq_ts_FOMe-ipso-OF-pA_1_2.1286012.out

Coordinates (Angs)
  48
fwd_frq_ts_FOMe-ipsoBa-preFF-pA_1_2.1286012.out        -6970.6407
C       0.000000    0.000000    4.942568
C      -0.286307    0.325524    3.653685
C      -0.617362   -0.698466    2.615616
C      -0.670402   -2.094442    3.148402
C      -0.388645   -2.398350    4.443595
C      -0.077835   -1.349876    5.325807
C      -1.943590   -0.374484    1.920563
N      -1.819977   -0.049057    0.685779
C      -2.942350    0.270298   -0.183231
C      -1.692431    0.505405   -2.353608
N      -0.485451    0.726467   -1.793957
C       0.428326    1.465606   -2.442818
C       0.182848    2.017224   -3.688159
C      -1.049184    1.788460   -4.286470
C      -1.990934    1.027527   -3.609823
Cu      0.000000    0.000000    0.000000
O       1.798031    0.000000   -0.438791
H      -3.893224   -2.236848    1.569344
O       0.444912   -0.673944    1.687402
H       1.377823    1.592920   -1.932140
H       0.951741    2.613653   -4.170318
H      -1.278148    2.202007   -5.265859
H      -2.967788    0.832540   -4.043289
H      -2.797370    1.168327    3.970856
H      -4.805658    0.882177    5.321719
H       2.243969   -0.286961    0.373165
O      -6.631289   -1.109528    4.917482
H      -7.884340   -0.669352    6.434973
C      -6.952886   -0.271078    6.029907
H      -0.880161   -2.875933    2.421577
H      -0.367459   -3.422436    4.805907
H      -0.200735    1.350483    3.299841
H       0.314655    0.742278    5.670846
F       0.204348   -1.657495    6.585917
H      -7.105085    0.767230    5.711210
C      -2.706611   -0.280430   -1.584339
H      -5.922526   -2.534079    2.944389
H      -6.169921   -0.315087    6.796559
C      -3.194916   -0.510366    2.673727
C      -3.471079    0.347282    3.740483
C      -4.615639    0.189075    4.509129
C      -5.501230   -0.855289    4.225499
C      -5.231013   -1.721312    3.154156
C      -4.097155   -1.546718    2.385632
H      -3.654638   -0.264036   -2.127421
H      -2.397123   -1.332126   -1.516061
H      -3.031384    1.363634   -0.217776
H      -3.875360   -0.127177    0.221792

Frequencies (cm-1)
   100.00    100.00    100.00    100.00    100.00    100.00    100.00    100.00    100.00    121.45
   140.01    150.79    157.45    166.56    185.72    196.93    216.42    226.41    227.79    254.84
   259.85    278.49    294.98    305.23    308.54    335.71    355.70    388.20    393.31    403.76
   410.92    414.05    416.78    459.27    479.60    488.68    500.42    519.25    528.59    560.02
   574.63    581.07    588.24    615.15    643.44    677.34    684.43    711.27    729.83    741.86
   749.76    755.42    775.93    783.23    793.34    801.85    803.08    819.27    848.60    873.39
   882.19    914.48    929.65    932.93    943.71    946.19    949.96    959.32    970.18    995.34
   996.06   1006.77   1014.98   1023.55   1038.51   1062.94   1066.05   1069.55   1089.71   1110.87
  1116.12   1124.02   1141.17   1150.31   1165.03   1167.03   1173.11   1217.85   1226.59   1239.32
  1246.23   1262.71   1272.03   1278.55   1290.56   1297.20   1316.71   1323.95   1343.81   1352.86
  1405.59   1414.35   1416.79   1418.61   1424.24   1427.98   1428.84   1434.60   1441.09   1478.05
  1500.61   1505.82   1571.46   1583.27   1596.50   1614.56   1617.03   1649.28   2973.31   3007.40
  3014.15   3067.21   3096.72   3099.09   3125.09   3140.06   3151.77   3151.81   3154.28   3155.21
  3161.05   3166.78   3170.40   3174.25   3174.53   3180.16   3185.22   3734.05
Note: any frequencies below 100 cm-1 (including spurious imaginary ones) are upscaled to 100 cm-1
      for the calculation of thermodynamic properties.
      (Averkiev, Truhlar, Catal. Sci. Technol. 2011, 1, 1526)

Thermodynamics

Note: this script does not take into account the spin entropy
For more info, see eq. 3 of Inorg. Chem. 2002, 41, 6928-6935
(M. Reiher), https://doi.org/10.1021/ic025891l

Temperature is now: 298.150

Reporting max. of 0 frequencies (set by $GETFREQSMAX env. variable)
```

```
Reading 1 outputfiles

            ScnFrq(1)
--------------------
--------------------
(ZPVE)        229.040
(dH,0->T)      16.582
(-TS)         -49.425
(dGibbs)      196.198
====================

Corresponding output files
 1 : frq_fwd_frq_ts_FOMe-ipso-OF-pA_1_2.1286221.out


System rev_frq_ts_FOMe-gamma-AB-pA_1_2

Reading 1 outputfiles

       Pauli        Elstat        OrbInt       Disp.       Solv.         TOTAL          Erel   Symm.      <S2>
-------------------------------------------------------------------------------------------------------------------
-----
   28229.704     -5974.602    -29139.720     -26.665     -43.372     -6954.823         0.000   NOSYM     0.752
ADF(1)

Corresponding output files
 1 : rev_frq_ts_FOMe-gamma-AB-pA_1_2.1283655.out

Coordinates (Angs)
  48
rev_frq_ts_FOMe-gamBa-AB-pA_1_2.1283655.out        -6954.8349
C       0.000000    0.000000    4.505255
C       0.539740    0.918453    3.618412
C      -0.184791    1.330478    2.491477
C      -1.488501    0.838336    2.295110
C      -2.044157   -0.071114    3.180621
C      -1.279391   -0.481612    4.262997
C       0.421620    2.223175    1.483153
N       0.137690    1.937338    0.242856
C       0.485398    2.781320   -0.886277
C       2.155394    1.042115   -1.634611
N       1.831534   -0.000000   -0.839661
C       2.779664   -0.849642   -0.421928
C       4.112547   -0.692080   -0.765665
C       4.466670    0.384539   -1.570132
C       3.477834    1.254453   -2.011760
Cu      0.000000    0.000000    0.000000
O      -0.086916   -2.746302   -0.501153
H       0.052946    4.087000    3.438145
O      -0.512283   -1.714085    0.362971
H       2.446693   -1.671299    0.207502
H       4.850854   -1.400461   -0.402134
H       5.503938    0.545905   -1.853731
H       3.720474    2.101301   -2.648101
H       1.181159    3.578553   -0.614073
H       0.227329    1.330207   -2.462866
H       0.335872   -3.364990    0.122772
H       2.945206    2.606685    0.615485
H       4.612596    4.158974    1.477815
H       5.493345    6.272954    1.943802
H      -2.081563    1.204498    1.459941
H      -3.053817   -0.450190    3.048979
H       1.544477    1.298226    3.783559
H       0.556212   -0.349609    5.370699
F      -1.806325   -1.376316    5.124327
H       1.367411    2.622898   -2.837100
C       1.036304    1.944917   -2.046435
H      -0.444689    3.255080   -1.226510
H       1.728167    5.697593    4.286200
C       1.362083    3.254351    1.934375
C       2.656614    3.300569    1.401636
C       3.611215    4.175117    1.894919
C       3.272925    5.050810    2.934006
C       1.977105    5.017585    3.474766
C       1.045764    4.118632    2.994674
O       4.119341    5.942032    3.486542
H       5.953197    6.773962    3.596985
C       5.464789    5.998631    3.004994
H       5.977874    5.041577    3.157926

Frequencies (cm-1)

Note: any frequencies below 100 cm-1 (including spurious imaginary ones) are upscaled to 100 cm-1
      for the calculation of thermodynamic properties.
      (Averkiev, Truhlar, Catal. Sci. Technol. 2011, 1, 1526)

Thermodynamics

Note: this script does not take into account the spin entropy
For more info, see eq. 3 of Inorg. Chem. 2002, 41, 6928-6935
(M. Reiher), https://doi.org/10.1021/ic025891l

Temperature is now: 298.150
```

```
Reporting max. of 0 frequencies (set by $GETFREQSMAX env. variable)


Reading 1 outputfiles

Unknown type of output file frq_rev_frq_ts_FOMe-gamma-AB-pA_1_2.1283958.out

Corresponding output files


System rev_frq_ts_FOMe-gamma-AB-pA_1_2

Reading 1 outputfiles

       Pauli        Elstat        OrbInt       Disp.       Solv.         TOTAL          Erel   Symm.      <S2>
------------------------------------------------------------------------------------------------------------------
-----
   28229.704     -5974.602    -29139.720     -26.665     -43.372     -6954.823         0.000   NOSYM     0.752
ADF(1)

Corresponding output files
 1 : rev_frq_ts_FOMe-gamma-AB-pA_1_2.1283655.out

Coordinates (Angs)
  48
rev_frq_ts_FOMe-gamBa-AB-pA_1_2.1283655.out        -6954.8349
C       0.000000    0.000000    4.505255
C       0.539740    0.918453    3.618412
C      -0.184791    1.330478    2.491477
C      -1.488501    0.838336    2.295110
C      -2.044157   -0.071114    3.180621
C      -1.279391   -0.481612    4.262997
C       0.421620    2.223175    1.483153
N       0.137690    1.937338    0.242856
C       0.485398    2.781320   -0.886277
C       2.155394    1.042115   -1.634611
N       1.831534   -0.000000   -0.839661
C       2.779664   -0.849642   -0.421928
C       4.112547   -0.692080   -0.765665
C       4.466670    0.384539   -1.570132
C       3.477834    1.254453   -2.011760
Cu      0.000000    0.000000    0.000000
O      -0.086916   -2.746302   -0.501153
H       0.052946    4.087000    3.438145
O      -0.512283   -1.714085    0.362971
H       2.446693   -1.671299    0.207502
H       4.850854   -1.400461   -0.402134
H       5.503938    0.545905   -1.853731
H       3.720474    2.101301   -2.648101
H       1.181159    3.578553   -0.614073
H       0.227329    1.330207   -2.462866
H       0.335872   -3.364990    0.122772
H       2.945206    2.606685    0.615485
H       4.612596    4.158974    1.477815
H       5.493345    6.272954    1.943802
H      -2.081563    1.204498    1.459941
H      -3.053817   -0.450190    3.048979
H       1.544477    1.298226    3.783559
H       0.556212   -0.349609    5.370699
F      -1.806325   -1.376316    5.124327
H       1.367411    2.622898   -2.837100
C       1.036304    1.944917   -2.046435
H      -0.444689    3.255080   -1.226510
H       1.728167    5.697593    4.286200
C       1.362083    3.254351    1.934375
C       2.656614    3.300569    1.401636
C       3.611215    4.175117    1.894919
C       3.272925    5.050810    2.934006
C       1.977105    5.017585    3.474766
C       1.045764    4.118632    2.994674
O       4.119341    5.942032    3.486542
H       5.953197    6.773962    3.596985
C       5.464789    5.998631    3.004994
H       5.977874    5.041577    3.157926

Frequencies (cm-1)
   100.00    100.00    100.00    100.00    100.00    100.00    100.00    100.00    100.00    100.00
   100.00    106.76    125.90    144.57    152.48    172.66    183.75    197.17    212.59    217.86
   249.78    257.23    278.81    301.67    303.28    316.80    330.56    365.79    396.77    403.18
   407.58    412.00    418.96    435.62    469.45    496.05    502.69    507.42    517.29    555.84
   580.67    593.03    614.40    624.04    640.87    662.51    696.84    727.21    741.60    749.48
   761.92    778.07    795.04    797.22    800.29    816.17    823.55    830.87    864.13    877.70
   905.87    923.74    942.54    943.81    949.35    952.97    954.32    959.21    994.82    997.31
  1000.24   1011.03   1022.75   1027.79   1055.96   1064.34   1099.15   1108.16   1113.78   1122.92
  1144.14   1148.98   1162.43   1168.62   1177.23   1190.14   1202.66   1216.02   1225.32   1270.09
  1274.09   1286.78   1293.08   1308.42   1316.69   1321.77   1338.19   1341.29   1358.11   1365.19
  1414.64   1416.04   1418.01   1427.81   1428.67   1431.72   1435.91   1441.54   1473.85   1489.59
  1501.43   1559.42   1571.15   1580.73   1590.62   1605.19   1613.04   1619.51   2975.14   3004.00
  3010.15   3069.58   3085.99   3101.69   3125.83   3145.78   3146.47   3151.81   3152.87   3156.43
  3159.04   3162.22   3168.12   3173.88   3175.07   3180.63   3184.18   3644.29
Note: any frequencies below 100 cm-1 (including spurious imaginary ones) are upscaled to 100 cm-1
      for the calculation of thermodynamic properties.
      (Averkiev, Truhlar, Catal. Sci. Technol. 2011, 1, 1526)

Thermodynamics
```

```
Note: this script does not take into account the spin entropy
For more info, see eq. 3 of Inorg. Chem. 2002, 41, 6928-6935
(M. Reiher), https://doi.org/10.1021/ic025891l

Temperature is now: 298.150

Reporting max. of 0 frequencies (set by $GETFREQSMAX env. variable)


Reading 1 outputfiles

            ScnFrq(1)
---------------------
---------------------
(ZPVE)        229.543
(dH,0->T)      16.960
(-TS)         -50.673
(dGibbs)      195.830
=====================

Corresponding output files
 1 : frq_rev_frq_ts_FOMe-gamma-AB-pA_1_2.1284183.out


System rev_frq_ts_FOMe-gamma-BC-pA_1_2

Reading 1 outputfiles

       Pauli        Elstat        OrbInt       Disp.       Solv.         TOTAL          Erel   Symm.      <S2>
-----------------------------------------------------------------------------------------------------------------
-----
   28333.872     -6043.989    -29186.702     -25.416     -45.803     -6968.210         0.000   NOSYM     0.760
ADF(1)

Corresponding output files
 1 : rev_frq_ts_FOMe-gamma-BC-pA_1_2.1304159.out

Coordinates (Angs)
  48
rev_frq_ts_FOMe-gamBa-BC-pA_1_2.1304159.out        -6968.2167
C       0.000000   -0.000000    4.993635
C      -1.094398    0.076107    4.142120
C      -1.042290   -0.406107    2.839771
C       0.129358   -1.247895    2.428062
C       1.331266   -1.105004    3.291763
C       1.204363   -0.558201    4.529128
C      -2.101509   -0.118315    1.903769
N      -1.849648   -0.048954    0.624219
C      -2.922043    0.216172   -0.329810
C      -1.495981    1.480422   -1.944742
N      -0.372114    1.490562   -1.205091
C       0.506440    2.498560   -1.299134
C       0.285922    3.572018   -2.144492
C      -0.871045    3.587641   -2.914114
C      -1.766797    2.531733   -2.814190
Cu      0.000000    0.000000    0.000000
O       1.764259   -0.000000   -0.500645
H      -3.705670   -2.090064    2.679190
O       0.342868   -1.478270    1.100352
H       1.398903    2.413776   -0.686331
H       1.015614    4.374729   -2.192360
H      -1.074221    4.415110   -3.589774
H      -2.676808    2.508813   -3.407082
H      -3.452757    1.136840   -0.060810
H      -1.895588   -0.612047   -2.037745
H       2.149508   -0.723187    0.019611
H      -3.525033    2.208016    2.343021
H      -7.298164    3.905072    3.885752
H      -7.053020    0.300047    3.908101
H      -0.249729   -2.244197    2.815634
H       2.263270   -1.545257    2.948513
H      -1.985993    0.595882    4.482200
H      -0.043820    0.413324    5.997127
F       2.272921   -0.504021    5.350180
H      -3.273993    0.415183   -2.424411
C      -2.413264    0.315797   -1.759009
H      -3.645029   -0.605879   -0.259135
H      -6.004714   -1.899622    3.591747
C      -3.479497    0.046865    2.441938
C      -4.060189    1.298239    2.606621
C      -5.351310    1.400161    3.144673
C      -6.051655    0.241884    3.494255
C      -5.455235   -1.002444    3.315368
C      -4.170331   -1.115123    2.802378
O      -5.829032    2.658846    3.289046
C      -7.129899    2.827332    3.855891
H      -7.899157    2.353785    3.233532
H      -7.174495    2.421081    4.873975

Frequencies (cm-1)
  100.00    100.00    100.00    100.00    100.00    100.00    100.00    100.00    104.35    115.77
  126.14    155.09    161.89    179.37    189.95    212.85    219.01    230.88    242.56    264.39
  269.84    299.77    312.04    321.27    337.26    350.81    370.85    387.70    401.22    408.42
  415.86    422.62    434.16    465.63    496.32    502.68    516.98    523.86    532.90    545.12
```

```
  556.88    574.29    581.98    612.73    644.04    653.13    686.09    690.39    712.10    738.17
  743.00    751.10    777.91    791.94    798.02    802.15    806.92    820.64    857.99    870.22
  878.34    896.33    933.94    935.81    941.69    947.26    954.03    957.91    968.86    994.91
  996.70   1021.66   1024.03   1031.78   1054.77   1059.57   1071.33   1100.28   1111.16   1114.58
 1122.25   1131.70   1145.17   1149.41   1162.62   1166.80   1171.84   1206.91   1210.02   1218.77
 1226.42   1262.64   1268.56   1288.01   1293.29   1314.04   1318.40   1331.59   1335.64   1344.78
 1362.78   1396.57   1413.83   1415.05   1427.13   1427.52   1429.33   1437.53   1440.92   1478.42
 1493.90   1501.16   1529.85   1573.55   1577.92   1585.80   1614.90   1616.63   2683.37   2974.37
 3007.29   3009.53   3068.30   3073.87   3102.16   3126.58   3146.73   3148.67   3154.45   3159.62
 3162.70   3169.28   3172.56   3176.63   3179.22   3183.53   3184.34   3720.16
Note: any frequencies below 100 cm-1 (including spurious imaginary ones) are upscaled to 100 cm-1
      for the calculation of thermodynamic properties.
      (Averkiev, Truhlar, Catal. Sci. Technol. 2011, 1, 1526)

Thermodynamics

Note: this script does not take into account the spin entropy
For more info, see eq. 3 of Inorg. Chem. 2002, 41, 6928-6935
(M. Reiher), https://doi.org/10.1021/ic025891l

Temperature is now: 298.150

Reporting max. of 0 frequencies (set by $GETFREQSMAX env. variable)


Reading 1 outputfiles

            ScnFrq(1)
---------------------
---------------------
(ZPVE)        228.413
(dH,0->T)      16.411
(-TS)         -49.069
(dGibbs)      195.755
=====================

Corresponding output files
 1 : frq_rev_frq_ts_FOMe-gamma-BC-pA_1_2.1304206.out


System rev_frq_ts_FOMe-gamma-CD-pA_1_2

Reading 1 outputfiles

       Pauli        Elstat        OrbInt       Disp.       Solv.         TOTAL          Erel   Symm.      <S2>
------------------------------------------------------------------------------------------------------------------
-----
   28461.750     -6036.166    -29359.017     -24.920     -48.693     -7007.225         0.000   NOSYM     0.752
ADF(1)

Corresponding output files
 1 : rev_frq_ts_FOMe-gamma-CD-pA_1_2.1285049.out

Coordinates (Angs)
  48
rev_frq_ts_FOMe-gamBa-CD-pA_1_2.1285049.out        -7007.2247
C       0.000000    0.000000    4.588618
C      -0.136350    0.302181    3.244447
C       0.973747    0.941531    2.579478
C       2.141240    1.410090    3.403872
C       2.141794    0.987718    4.808893
C       1.129720    0.302400    5.383093
C      -1.399222    0.039784    2.556792
H      -2.755665    1.818486    4.132369
O       1.038500    1.154606    1.352534
Cu      0.000000    0.000000    0.000000
O       1.562318   -0.000000   -1.029995
C      -2.149743   -2.766589   -3.404812
C      -3.088349   -1.324770   -0.051017
H      -3.593615    0.237653    1.323269
H      -2.792929    0.800303   -0.150434
N      -1.045022   -1.148835   -1.426615
H      -3.875315   -2.817993   -2.115747
C      -2.293198   -1.635668   -1.280885
N      -1.492621    0.044646    1.258620
H      -4.150295   -1.345087   -0.316163
H      -2.943393   -2.135815    0.674593
C      -0.355478   -1.453174   -2.541412
H      -0.261497   -2.464711   -4.425370
C      -2.863879   -2.447556   -2.262831
C      -0.866165   -2.253595   -3.547972
H      -0.820738   -0.516218    5.080244
H       1.169992    0.009985    6.427165
F       3.221281    1.331839    5.503610
H       2.165440    2.511103    3.358507
H       3.069791    1.089867    2.911070
H       2.196207    0.546441   -0.543688
O      -5.936808   -0.868484    5.772010
H      -6.890867   -2.498221    4.872913
C      -6.545109   -2.158536    5.856948
H      -5.853281   -2.895374    6.282991
C      -2.588402   -0.210206    3.410046
C      -3.134549   -1.492043    3.477287
C      -4.246715   -1.759950    4.264516
C      -4.842823   -0.724853    4.991457
```

```
C      -4.293335    0.564967    4.936834
C      -3.172651    0.814030    4.165872
H      -2.672758   -2.306654    2.924453
H      -4.636131   -2.771763    4.303668
H      -7.401236   -2.034706    6.521962
H      -4.761877    1.362259    5.509372
H       0.642245   -1.028858   -2.598009
H      -2.590482   -3.398700   -4.172535
C      -2.796788    0.009828    0.610576

Frequencies (cm-1)
  100.00    100.00    100.00    100.00    100.00    100.00    100.00    100.00    100.00    100.00
  131.21    137.81    141.52    161.73    163.86    199.20    209.74    211.69    219.56    228.52
  247.65    275.26    286.09    293.74    299.73    325.58    338.65    357.68    371.94    401.58
  403.59    426.61    447.54    453.58    462.93    493.59    512.51    516.51    523.17    539.32
  571.02    584.52    594.85    608.51    621.15    632.43    672.04    712.68    726.60    731.49
  745.41    749.76    779.64    797.19    802.37    818.52    830.32    835.97    853.41    870.28
  879.92    891.07    932.70    934.49    949.39    965.15    971.09    982.80    995.34    996.56
 1000.89   1019.28   1026.01   1064.51   1077.82   1095.40   1110.19   1112.89   1122.87   1127.23
 1143.38   1150.01   1162.37   1169.07   1180.32   1195.96   1209.68   1234.74   1255.75   1263.86
 1276.60   1285.15   1295.95   1299.48   1312.41   1319.89   1331.31   1350.66   1353.63   1376.13
 1390.98   1394.02   1415.22   1423.19   1427.52   1429.48   1435.15   1439.73   1478.66   1500.62
 1530.70   1551.54   1575.71   1582.21   1590.60   1615.05   1618.61   1643.00   2972.66   2989.03
 3003.74   3013.69   3044.52   3065.58   3077.67   3082.71   3121.00   3141.81   3146.88   3148.41
 3159.24   3159.86   3160.23   3164.52   3175.59   3184.15   3186.91   3755.99
Note: any frequencies below 100 cm-1 (including spurious imaginary ones) are upscaled to 100 cm-1
      for the calculation of thermodynamic properties.
      (Averkiev, Truhlar, Catal. Sci. Technol. 2011, 1, 1526)

Thermodynamics

Note: this script does not take into account the spin entropy
For more info, see eq. 3 of Inorg. Chem. 2002, 41, 6928-6935
(M. Reiher), https://doi.org/10.1021/ic025891l

Temperature is now: 298.150

Reporting max. of 0 frequencies (set by $GETFREQSMAX env. variable)


Reading 1 outputfiles

            ScnFrq(1)
--------------------
--------------------
(ZPVE)        229.501
(dH,0->T)      16.780
(-TS)         -50.099
(dGibbs)      196.182
====================

Corresponding output files
 1 : frq_rev_frq_ts_FOMe-gamma-CD-pA_1_2.1285638.out


System rev_frq_ts_FOMe-ipso-AO-pA_1_2

Reading 1 outputfiles

       Pauli        Elstat        OrbInt       Disp.       Solv.         TOTAL          Erel   Symm.      <S2>
------------------------------------------------------------------------------------------------------------------
-----
   28254.528     -5968.883    -29175.729     -24.731     -42.423     -6957.413         0.000   NOSYM     0.752
ADF(1)

Corresponding output files
 1 : rev_frq_ts_FOMe-ipso-AO-pA_1_2.1286302.out

Coordinates (Angs)
  48
fwd_frq_ts_FOMe-ipsoBa-AF-pA_1_2.1286302.out        -6957.4933
C       0.000000    0.000000    4.148843
C       0.717403   -0.276041    2.993979
C       1.836619    0.491418    2.644098
C       2.245449    1.527848    3.493924
C       1.528157    1.829492    4.641787
C       0.414789    1.059068    4.942893
C       2.580646    0.250392    1.392986
N       1.907887   -0.000000    0.309457
C       2.533560   -0.338608   -0.964353
C       1.156910    1.388401   -2.355713
N       0.046672    1.151927   -1.633723
C      -1.113901    1.743866   -1.972603
C      -1.221175    2.610913   -3.042710
C      -0.084495    2.861951   -3.805097
C       1.103297    2.241517   -3.460552
Cu      0.000000    0.000000    0.000000
O      -2.719003    0.070898    0.528660
H       4.334043    1.785973   -0.075992
O      -1.643832   -0.778354    0.192625
H      -1.973645    1.507200   -1.349535
H      -2.177147    3.073362   -3.269618
H      -0.124904    3.534059   -4.658992
H       2.009676    2.415893   -4.034858
H       4.145024   -1.099948    3.106919
```

```
H       6.612379   -1.111680    3.222186
H      -3.465184   -0.372988    0.083776
O       8.196041    0.232702    1.768749
H      10.048827    0.751797    1.164111
C       9.022474    0.997917    0.887992
H       3.121434    2.119527    3.240734
H       1.820997    2.644967    5.297637
H       0.426700   -1.129060    2.384554
H      -0.858406   -0.597985    4.442694
F      -0.285536    1.341961    6.063971
H       8.854234    2.073320    1.021294
C       2.485650    0.801195   -1.972244
H       6.760965    1.800684    0.050257
H       8.845950    0.719375   -0.158045
C       4.051615    0.303845    1.465072
C       4.719064   -0.475589    2.425667
C       6.097650   -0.486004    2.496509
C       6.856959    0.315708    1.629301
C       6.204374    1.139432    0.706027
C       4.819857    1.117750    0.627919
H       1.990099   -1.203005   -1.367790
H       3.576134   -0.643723   -0.830955
H       2.980053    0.453786   -2.886581
H       3.099000    1.635486   -1.609444

Frequencies (cm-1)
   100.00    100.00    100.00    100.00    100.00    100.00    100.00    100.00    100.00    100.00
   100.00    122.74    130.02    140.81    154.17    167.39    184.03    201.29    205.19    215.18
   225.90    245.28    282.29    293.33    311.46    332.77    338.54    354.90    400.29    404.10
   407.44    413.06    427.47    447.89    455.58    495.04    500.91    510.00    519.80    556.71
   583.43    597.29    616.96    625.25    640.14    671.50    683.79    717.69    732.26    741.36
   750.02    774.24    794.29    796.63    801.71    813.07    823.48    828.40    854.53    879.52
   912.67    919.92    933.73    940.79    951.63    953.44    960.96    989.42    993.59    997.71
  1005.44   1009.48   1015.09   1025.40   1064.88   1084.51   1099.72   1111.29   1114.18   1123.62
  1145.39   1153.98   1163.43   1166.53   1178.62   1201.80   1211.64   1223.01   1256.03   1268.59
  1274.39   1284.82   1298.61   1312.55   1320.86   1331.58   1352.13   1355.13   1360.19   1367.02
  1387.89   1411.92   1415.53   1427.89   1429.36   1429.94   1440.94   1442.26   1485.05   1492.98
  1502.74   1572.55   1577.07   1582.20   1597.32   1609.09   1615.67   1622.21   2972.99   2997.53
  3015.78   3067.11   3068.74   3072.89   3124.39   3142.88   3144.71   3147.54   3152.35   3156.12
  3161.07   3164.79   3169.46   3171.41   3173.47   3179.30   3185.30   3639.73
Note: any frequencies below 100 cm-1 (including spurious imaginary ones) are upscaled to 100 cm-1
      for the calculation of thermodynamic properties.
      (Averkiev, Truhlar, Catal. Sci. Technol. 2011, 1, 1526)

Thermodynamics

Note: this script does not take into account the spin entropy
For more info, see eq. 3 of Inorg. Chem. 2002, 41, 6928-6935
(M. Reiher), https://doi.org/10.1021/ic025891l

Temperature is now: 298.150

Reporting max. of 0 frequencies (set by $GETFREQSMAX env. variable)


Reading 1 outputfiles

            ScnFrq(1)
---------------------
---------------------
(ZPVE)        229.665
(dH,0->T)      16.961
(-TS)         -50.768
(dGibbs)      195.858
=====================

Corresponding output files
 1 : frq_rev_frq_ts_FOMe-ipso-AO-pA_1_2.1287546.out


System rev_frq_ts_FOMe-ipso-FG-pA_1_2

Reading 1 outputfiles

       Pauli        Elstat        OrbInt       Disp.       Solv.         TOTAL          Erel   Symm.      <S2>
------------------------------------------------------------------------------------------------------------------
-----
   28285.400     -6021.066    -29163.922     -25.617     -46.702     -6972.075         0.000   NOSYM     0.758
ADF(1)

Corresponding output files
 1 : rev_frq_ts_FOMe-ipso-FG-pA_1_2.1285283.out

Coordinates (Angs)
  48
fwd_frq_ts_FOMe-ipsoBa-FG-pA_1_2.1285283.out        -6972.0755
C       0.000000    0.000000    5.051337
C      -0.431410    0.466057    3.846280
C      -0.630958   -0.423905    2.668268
C      -0.470523   -1.879451    2.982736
C      -0.042749   -2.324837    4.194240
C       0.182651   -1.381786    5.212408
C      -1.956442   -0.185714    1.933843
N      -1.817743   -0.157911    0.648333
C      -2.930662   -0.171481   -0.286761
```

```
C      -1.637658   -0.081914   -2.426560
N      -0.492204    0.376950   -1.884991
C       0.328135    1.157161   -2.606358
C       0.037913    1.521847   -3.909714
C      -1.135622    1.054173   -4.486990
C      -1.975983    0.244883   -3.736756
Cu      0.000000    0.000000    0.000000
O       1.798216    0.000000   -0.425212
H      -2.810431   -1.538119    4.150877
O       0.449726   -0.098268    1.804546
H       1.237047    1.477357   -2.106385
H       0.726321    2.163042   -4.452189
H      -1.397734    1.319723   -5.508415
H      -2.904002   -0.137179   -4.152804
H      -3.881291    1.637552    1.435700
H      -5.897200    2.073025    2.715078
H       2.243751   -0.147485    0.423536
O      -6.611603    0.654681    4.936656
H      -8.347461    1.598253    5.344686
C      -7.564374    1.664819    4.588305
H      -0.621664   -2.567198    2.153517
H       0.159375   -3.375091    4.385899
H      -0.559460    1.531573    3.671996
H       0.219712    0.665306    5.881703
F       0.620287   -1.824074    6.387239
H      -7.991742    1.475499    3.596645
C      -2.543568   -0.901978   -1.566119
H      -4.858987   -1.102737    5.438589
H      -7.108281    2.661261    4.616589
C      -3.195668   -0.000364    2.669674
C      -4.096800    1.007165    2.292865
C      -5.239895    1.268472    3.027174
C      -5.523695    0.498879    4.162309
C      -4.629062   -0.511229    4.555691
C      -3.480353   -0.744254    3.831053
H      -3.226769    0.855425   -0.531040
H      -3.789670   -0.665467    0.179520
H      -3.454560   -1.134247   -2.122936
H      -2.066589   -1.858036   -1.312998

Frequencies (cm-1)
  100.00    100.00    100.00    100.00    100.00    100.00    100.00    100.00    100.15    126.41
  134.08    156.70    170.48    172.27    186.35    199.98    209.40    218.95    227.43    251.76
  259.29    280.03    305.53    309.68    315.04    333.73    346.76    384.00    391.40    402.99
  404.62    414.12    416.00    457.22    478.26    495.14    503.62    518.04    530.22    566.38
  578.12    582.39    587.38    619.45    644.26    671.32    686.68    703.57    730.66    743.32
  747.03    751.87    776.83    785.80    789.54    797.33    807.70    820.05    853.39    873.92
  883.54    916.30    927.77    935.66    939.65    945.15    950.56    957.34    969.33    982.70
  994.52    995.03   1017.01   1020.14   1038.49   1060.99   1065.88   1074.26   1096.60   1110.88
 1122.07   1122.69   1141.79   1150.90   1163.74   1164.56   1172.61   1219.14   1232.62   1240.53
 1244.91   1275.00   1280.31   1282.17   1295.48   1298.09   1321.24   1329.16   1343.34   1359.18
 1406.47   1411.88   1414.27   1416.91   1424.25   1429.76   1433.94   1435.97   1445.71   1478.42
 1503.19   1504.69   1565.98   1584.22   1587.71   1601.07   1615.63   1628.18   2977.25   3005.26
 3013.54   3072.90   3073.19   3100.87   3129.40   3149.32   3153.12   3153.56   3157.72   3166.75
 3167.68   3168.14   3171.59   3171.99   3174.77   3181.76   3187.62   3734.50
Note: any frequencies below 100 cm-1 (including spurious imaginary ones) are upscaled to 100 cm-1
      for the calculation of thermodynamic properties.
      (Averkiev, Truhlar, Catal. Sci. Technol. 2011, 1, 1526)

Thermodynamics

Note: this script does not take into account the spin entropy
For more info, see eq. 3 of Inorg. Chem. 2002, 41, 6928-6935
(M. Reiher), https://doi.org/10.1021/ic025891l

Temperature is now: 298.150

Reporting max. of 0 frequencies (set by $GETFREQSMAX env. variable)


Reading 1 outputfiles

            ScnFrq(1)
---------------------
---------------------
(ZPVE)        229.099
(dH,0->T)      16.564
(-TS)         -49.382
(dGibbs)      196.281
=====================

Corresponding output files
 1 : frq_rev_frq_ts_FOMe-ipso-FG-pA_1_2.1285861.out


System rev_frq_ts_FOMe-ipso-GH-pA_1_2

Reading 1 outputfiles

       Pauli        Elstat        OrbInt       Disp.       Solv.         TOTAL          Erel   Symm.      <S2>
----------------------------------------------------------------------------------------------------------------
-----
   28376.268     -6020.673    -29278.890     -25.854     -48.631     -6997.955         0.000   NOSYM     0.753
ADF(1)
```

```
Corresponding output files
 1 : rev_frq_ts_FOMe-ipso-GH-pA_1_2.1285288.out

Coordinates (Angs)
  48
rev_frq_ts_FOMe-ipsoBa-GH-pA_1_2.1285288.out        -6997.9547
C       0.000000   -0.000000    4.508572
C      -0.101194   -0.153568    3.036666
C       0.461398   -1.441072    2.458101
C       0.922168   -2.462044    3.349073
C       0.977385   -2.228683    4.685071
C       0.540363   -0.983468    5.240669
C      -1.500186    0.111232    2.458567
N      -1.601279    0.222720    1.179699
C      -2.916526    0.363335    0.548623
C      -2.109929    1.024349   -1.727019
N      -0.794724    1.134175   -1.462198
C      -0.048543    2.041170   -2.105993
C      -0.591049    2.899428   -3.048900
C      -1.948231    2.803516   -3.333034
C      -2.712866    1.854550   -2.667678
Cu      0.000000    0.000000    0.000000
O       1.701676   -0.000000   -0.774747
H      -2.358284   -1.771256    4.222485
O       0.527411   -1.584604    1.219808
H       1.006531    2.054504   -1.845605
H       0.044676    3.626227   -3.546167
H      -2.408986    3.461879   -4.066007
H      -3.776021    1.750515   -2.867544
H      -3.157144    2.221675    2.816096
H      -5.039581    2.404323    4.357838
H       2.219389   -0.687063   -0.330232
H      -3.261794    1.396767    0.661102
H      -3.639449   -0.279658    1.063147
H      -3.907894   -0.029592   -1.297546
H       1.260294   -3.398947    2.915460
H       1.361226   -2.984279    5.367340
H       0.534166    0.637417    2.596712
H      -0.350397    0.919201    4.967989
O      -5.827957    0.387021    6.023599
H      -7.146883    1.764968    5.165414
C      -2.877812    0.015810   -0.934046
H      -4.289986   -1.605706    5.748137
H      -2.443857   -0.983558   -1.071997
C      -2.631865    0.209381    3.388407
C      -3.405714    1.370931    3.445544
C      -4.472554    1.480012    4.324988
C      -4.799992    0.399387    5.151552
C      -4.025951   -0.771479    5.102498
C      -2.945490   -0.855670    4.247567
F       0.677031   -0.853872    6.577245
H      -7.408740    1.306385    6.873290
C      -6.655869    1.548723    6.121814
H      -6.075816    2.419735    6.449656

Frequencies (cm-1)
   100.00    100.00    100.00    100.00    100.00    100.00    100.00    100.00    101.52    114.10
   119.87    137.70    151.33    159.85    175.87    190.88    205.30    209.39    223.21    231.56
   242.24    279.21    297.09    305.68    312.88    319.25    351.37    364.34    389.43    404.11
   407.41    415.86    434.75    456.26    472.06    495.23    504.22    511.91    524.25    530.23
   577.59    600.36    609.11    630.74    641.96    643.25    680.96    731.60    743.55    750.04
   758.79    771.17    780.13    785.91    795.96    812.11    816.40    822.07    823.89    846.91
   874.60    878.55    935.30    941.26    950.80    956.62    964.92    966.73    994.51    995.31
  1001.32   1015.00   1023.11   1052.27   1058.58   1067.96   1077.32   1097.15   1107.42   1117.03
  1122.62   1133.61   1148.88   1163.38   1164.74   1170.62   1180.90   1219.89   1222.83   1239.82
  1267.83   1271.74   1276.42   1291.86   1296.12   1308.14   1323.10   1332.47   1344.32   1357.01
  1408.37   1414.50   1415.15   1415.88   1428.03   1428.77   1436.26   1441.04   1477.88   1501.12
  1545.82   1572.49   1584.75   1586.02   1605.42   1614.61   1625.20   1671.51   2945.76   2974.78
  3008.08   3010.28   3068.83   3073.46   3096.67   3125.90   3142.73   3150.08   3152.24   3156.10
  3161.13   3162.45   3167.38   3175.80   3176.84   3177.51   3185.04   3752.96
Note: any frequencies below 100 cm-1 (including spurious imaginary ones) are upscaled to 100 cm-1
      for the calculation of thermodynamic properties.
      (Averkiev, Truhlar, Catal. Sci. Technol. 2011, 1, 1526)

Thermodynamics

Note: this script does not take into account the spin entropy
For more info, see eq. 3 of Inorg. Chem. 2002, 41, 6928-6935
(M. Reiher), https://doi.org/10.1021/ic025891l

Temperature is now: 298.150

Reporting max. of 0 frequencies (set by $GETFREQSMAX env. variable)


Reading 1 outputfiles

            ScnFrq(1)
---------------------
---------------------
(ZPVE)        229.317
(dH,0->T)      16.716
(-TS)         -49.871
(dGibbs)      196.163
=====================
```

```
Corresponding output files
 1 : frq_rev_frq_ts_FOMe-ipso-GH-pA_1_2.1285786.out


System rev_frq_ts_FOMe-ipso-OF-pA_1_2

Reading 1 outputfiles

       Pauli        Elstat        OrbInt       Disp.       Solv.         TOTAL          Erel   Symm.      <S2>
-----------------------------------------------------------------------------------------------------------------
-----
   28166.240     -5969.552    -29075.639     -27.035     -47.324     -6953.478         0.000   NOSYM     0.760
ADF(1)

Corresponding output files
 1 : rev_frq_ts_FOMe-ipso-OF-pA_1_2.1286013.out

Coordinates (Angs)
  48
rev_frq_ts_FOMe-ipsoBa-preFF-pA_1_2.1286013.out        -6953.4751
C       0.000000   -0.000000    3.995820
C      -0.830885    0.262024    2.916056
C      -1.622187   -0.751415    2.365457
C      -1.590730   -2.035137    2.918447
C      -0.743870   -2.318762    3.978253
C       0.034879   -1.293005    4.495101
C      -2.496643   -0.463547    1.208557
N      -1.923154   -0.010959    0.127637
C      -2.611912    0.547600   -1.025677
C      -0.969073    2.470798   -0.930298
N      -0.071979    1.928426   -0.085024
C       0.689724    2.690518    0.711171
C       0.561874    4.068311    0.716236
C      -0.372166    4.654326   -0.130335
C      -1.137240    3.850149   -0.964393
Cu      0.000000    0.000000    0.000000
O       1.821038   -0.000000   -0.066115
H      -4.374687   -1.367725   -0.635499
O       0.046144   -1.749493   -0.046766
H       1.403311    2.166890    1.341363
H       1.184635    4.660919    1.379214
H      -0.501533    5.733672   -0.144561
H      -1.862076    4.278584   -1.650477
H      -3.858490   -0.111638    3.461469
H      -6.227464   -0.480680    3.884504
H       2.095203   -0.890263    0.212266
O      -7.963430   -1.404381    1.991963
H      -9.629771   -1.490003    3.125828
C      -8.586463   -1.205654    3.267086
H      -2.215637   -2.820074    2.500234
H      -0.683804   -3.315393    4.406698
H      -0.900105    1.274321    2.526950
H       0.604104    0.779320    4.452093
F       0.849859   -1.562263    5.537363
H      -8.526880   -0.154801    3.571891
C      -1.717343    1.517918   -1.801968
H      -6.750259   -1.771515   -0.196634
H      -8.128237   -1.846151    4.029211
C      -3.926234   -0.678212    1.376579
C      -4.487562   -0.465443    2.649559
C      -5.830855   -0.673321    2.893501
C      -6.655118   -1.143143    1.861242
C      -6.106291   -1.381846    0.587918
C      -4.772638   -1.139589    0.347811
H      -2.343148    2.065122   -2.510500
H      -0.990987    0.947835   -2.397182
H      -3.517841    1.058255   -0.679267
H      -2.905049   -0.254356   -1.710538

Frequencies (cm-1)
  100.00    100.00    100.00    100.00    100.00    100.00    100.00    100.00    100.00    100.00
  114.78    135.57    145.15    155.30    166.04    171.47    202.14    207.89    217.40    225.93
  249.71    261.78    280.01    304.37    323.91    327.46    335.90    363.17    394.97    401.42
  407.75    413.39    422.70    435.42    471.62    497.18    501.29    513.67    552.12    564.09
  590.48    597.41    613.78    617.64    620.08    659.29    679.92    703.63    731.09    744.17
  746.48    755.01    785.49    788.21    798.37    800.95    818.84    826.14    827.17    861.74
  875.63    910.53    922.22    939.80    947.74    953.20    954.17    957.28    975.95    994.14
  995.74   1003.33   1016.94   1024.87   1029.10   1052.30   1064.04   1097.29   1113.65   1120.21
 1122.26   1146.08   1151.10   1164.13   1170.45   1179.79   1196.09   1210.91   1216.74   1229.87
 1274.90   1281.21   1289.98   1292.47   1308.79   1319.86   1329.19   1348.75   1363.18   1368.61
 1411.50   1413.40   1414.79   1422.09   1428.65   1435.47   1439.37   1450.86   1478.79   1493.00
 1505.24   1545.31   1562.34   1584.10   1597.10   1608.54   1614.93   1619.99   2981.23   3005.98
 3007.51   3077.63   3079.42   3104.95   3131.18   3156.62   3158.07   3159.01   3159.50   3163.54
 3167.00   3174.79   3174.89   3175.21   3181.69   3186.04   3187.37   3705.62
Note: any frequencies below 100 cm-1 (including spurious imaginary ones) are upscaled to 100 cm-1
      for the calculation of thermodynamic properties.
      (Averkiev, Truhlar, Catal. Sci. Technol. 2011, 1, 1526)

Thermodynamics

Note: this script does not take into account the spin entropy
For more info, see eq. 3 of Inorg. Chem. 2002, 41, 6928-6935
(M. Reiher), https://doi.org/10.1021/ic025891l
```

```
Temperature is now: 298.150

Reporting max. of 0 frequencies (set by $GETFREQSMAX env. variable)


Reading 1 outputfiles

            ScnFrq(1)
---------------------
---------------------
(ZPVE)        229.310
(dH,0->T)      16.833
(-TS)         -50.188
(dGibbs)      195.955
=====================

Corresponding output files
 1 : frq_rev_frq_ts_FOMe-ipso-OF-pA_1_2.1286216.out


System ts_FOMe-gamma-AB-pA_1_2

Reading 1 outputfiles

       Pauli        Elstat        OrbInt       Disp.       Solv.         TOTAL          Erel   Symm.      <S2>
---------------------------------------------------------------------------------------------------------------------
-----
   28075.582     -5943.836    -28997.084     -27.207     -46.366     -6939.082         0.000   NOSYM     0.759
ADF(1)

Corresponding output files
 1 : ts_FOMe-gamma-AB-pA_1_2.1282285.out

Coordinates (Angs)
  48
ts_FOMe-gamBa-AB-pA_1_2.1282285.out        -6939.0999
C      -0.211790   -4.747388    1.996034
C       0.228568   -3.534854    2.501688
C      -0.447995   -2.342667    2.200303
C      -1.606547   -2.395027    1.410581
C      -2.068484   -3.603000    0.912956
C      -1.353967   -4.755138    1.208547
C       0.073264   -1.067679    2.713654
N       0.000000    0.000000    1.972813
C       0.389393    1.328031    2.426319
C       2.453505    0.968695    0.985452
N       1.948406   -0.000000    0.192440
C       2.739755   -0.953698   -0.317039
C       4.098040   -0.987891   -0.047221
C       4.639237    0.000497    0.766332
C       3.810463    0.989267    1.283366
Cu      0.000000    0.000000    0.000000
O      -0.427989    0.108141   -1.839458
H      -1.030199   -1.884683    5.055959
O      -1.700041    0.146155   -0.579479
H       2.260852   -1.698907   -0.947760
H       4.710981   -1.778914   -0.468252
H       5.700605    0.000171    1.001567
H       4.204595    1.774336    1.922556
H       0.738825    1.305157    3.459505
H       0.974378    2.439149    0.650555
H      -0.519090    1.051685   -2.069082
H       2.613065   -0.357853    3.353318
H       3.690823   -0.428590    5.537344
H       3.892763    0.274359    7.764292
H      -2.161997   -1.485539    1.201627
H      -2.972767   -3.662674    0.313297
H       1.120753   -3.509005    3.121723
H       0.312601   -5.675675    2.205621
F      -1.795500   -5.934022    0.720295
H       1.968429    2.752366    2.062637
C       1.461863    1.958251    1.509456
H      -0.507453    1.956963    2.385944
H       0.040966   -1.909482    7.286270
C       0.702178   -1.078773    4.047063
C       2.031675   -0.679397    4.213236
C       2.650274   -0.723512    5.452506
C       1.923913   -1.151001    6.570267
C       0.592190   -1.568351    6.412971
C      -0.001253   -1.549473    5.166205
O       2.415348   -1.204769    7.824248
H       3.925605   -0.887151    9.122808
C       3.760969   -0.776104    8.050102
H       4.472828   -1.404665    7.501749

Frequencies (cm-1)
 -628.76    -35.63    -15.60    100.00    100.00    100.00    100.00    100.00    100.00    100.00
  100.00    100.96    124.49    143.71    159.15    159.45    184.47    207.74    217.45    222.89
  227.45    256.14    285.76    303.28    317.57    331.76    369.55    390.45    404.41    412.00
  414.55    424.51    447.08    468.66    490.70    499.43    507.71    517.18    549.22    556.52
  583.83    603.37    610.72    615.97    624.44    653.67    669.63    693.58    731.15    745.16
  748.80    758.46    782.48    795.17    797.32    798.85    817.02    824.27    830.96    851.94
  880.50    920.72    924.68    941.00    943.88    951.71    953.63    955.56    961.51    996.51
  998.03   1002.40   1017.81   1022.60   1025.21   1048.82   1061.43   1102.36   1110.86   1114.12
 1122.92   1147.80   1151.22   1163.17   1169.49   1176.90   1184.17   1212.25   1216.94   1225.46
```

```
 1269.15   1279.10   1287.67   1293.41   1295.70   1314.84   1321.70   1341.44   1357.54   1368.64
 1414.80   1417.40   1419.83   1428.85   1429.39   1433.95   1439.92   1441.69   1478.15   1493.06
 1502.55   1560.33   1571.42   1583.33   1594.96   1608.98   1617.81   1620.64   2974.82   3004.38
 3020.78   3069.14   3096.40   3109.04   3125.66   3146.12   3152.23   3157.85   3157.98   3160.19
 3162.06   3163.74   3172.00   3175.22   3175.70   3183.11   3184.54   3679.79
Note: any frequencies below 100 cm-1 (including spurious imaginary ones) are upscaled to 100 cm-1
      for the calculation of thermodynamic properties.
      (Averkiev, Truhlar, Catal. Sci. Technol. 2011, 1, 1526)

Thermodynamics

Note: this script does not take into account the spin entropy
For more info, see eq. 3 of Inorg. Chem. 2002, 41, 6928-6935
(M. Reiher), https://doi.org/10.1021/ic025891l

Temperature is now: 298.150

Reporting max. of 0 frequencies (set by $GETFREQSMAX env. variable)


Reading 1 outputfiles

            ScnFrq(1)
--------------------
--------------------
(ZPVE)        229.148
(dH,0->T)      16.894
(-TS)         -50.547
(dGibbs)      195.496
====================

Corresponding output files
 1 : frq_ts_FOMe-gamma-AB-pA_1_2.1282751.out


System ts_FOMe-gamma-AB-pA_1_2

Reading 1 outputfiles

       Pauli        Elstat        OrbInt       Disp.       Solv.         TOTAL          Erel   Symm.      <S2>
------------------------------------------------------------------------------------------------------------------
-----
   28075.582     -5943.836    -28997.084     -27.207     -46.366     -6939.082         0.000   NOSYM     0.759
ADF(1)

Corresponding output files
 1 : ts_FOMe-gamma-AB-pA_1_2.1282285.out

Coordinates (Angs)
  48
ts_FOMe-gamBa-AB-pA_1_2.1282285.out        -6939.0999
C      -0.211790   -4.747388    1.996034
C       0.228568   -3.534854    2.501688
C      -0.447995   -2.342667    2.200303
C      -1.606547   -2.395027    1.410581
C      -2.068484   -3.603000    0.912956
C      -1.353967   -4.755138    1.208547
C       0.073264   -1.067679    2.713654
N       0.000000    0.000000    1.972813
C       0.389393    1.328031    2.426319
C       2.453505    0.968695    0.985452
N       1.948406   -0.000000    0.192440
C       2.739755   -0.953698   -0.317039
C       4.098040   -0.987891   -0.047221
C       4.639237    0.000497    0.766332
C       3.810463    0.989267    1.283366
Cu      0.000000    0.000000    0.000000
O      -0.427989    0.108141   -1.839458
H      -1.030199   -1.884683    5.055959
O      -1.700041    0.146155   -0.579479
H       2.260852   -1.698907   -0.947760
H       4.710981   -1.778914   -0.468252
H       5.700605    0.000171    1.001567
H       4.204595    1.774336    1.922556
H       0.738825    1.305157    3.459505
H       0.974378    2.439149    0.650555
H      -0.519090    1.051685   -2.069082
H       2.613065   -0.357853    3.353318
H       3.690823   -0.428590    5.537344
H       3.892763    0.274359    7.764292
H      -2.161997   -1.485539    1.201627
H      -2.972767   -3.662674    0.313297
H       1.120753   -3.509005    3.121723
H       0.312601   -5.675675    2.205621
F      -1.795500   -5.934022    0.720295
H       1.968429    2.752366    2.062637
C       1.461863    1.958251    1.509456
H      -0.507453    1.956963    2.385944
H       0.040966   -1.909482    7.286270
C       0.702178   -1.078773    4.047063
C       2.031675   -0.679397    4.213236
C       2.650274   -0.723512    5.452506
C       1.923913   -1.151001    6.570267
C       0.592190   -1.568351    6.412971
C      -0.001253   -1.549473    5.166205
```

```
O       2.415348   -1.204769    7.824248
H       3.925605   -0.887151    9.122808
C       3.760969   -0.776104    8.050102
H       4.472828   -1.404665    7.501749

Frequencies (cm-1)
 -164.64    -24.10     -5.05    100.00    100.00    100.00    100.00    100.00    100.00    100.00
  103.84    140.48    149.00    166.23    171.44    201.78    206.37    213.36    239.29    241.95
  253.83    262.36    293.31    309.87    320.54    327.48    364.55    369.03    393.86    399.86
  408.36    421.18    428.51    434.91    470.97    500.40    509.10    512.34    553.11    559.31
  583.58    590.08    600.27    608.09    621.60    651.68    654.65    697.84    719.83    745.23
  747.55    759.12    778.78    791.27    795.90    804.70    814.38    824.63    827.38    860.88
  875.58    903.94    918.07    924.99    945.92    946.55    951.60    955.34    959.77    994.80
  995.86    996.80   1018.19   1023.07   1027.89   1051.80   1063.75   1090.08   1111.50   1117.12
 1120.87   1139.40   1150.30   1162.52   1167.64   1172.55   1191.57   1205.82   1207.29   1227.75
 1269.61   1277.48   1287.55   1291.12   1306.71   1323.91   1325.60   1343.79   1359.49   1362.57
 1398.17   1414.12   1419.32   1425.77   1429.71   1434.20   1439.34   1446.81   1478.79   1486.58
 1501.14   1530.95   1552.05   1563.46   1584.02   1589.55   1615.91   1617.40   2979.12   3006.36
 3013.83   3076.00   3093.88   3110.58   3130.47   3150.04   3156.92   3158.48   3158.64   3165.26
 3169.16   3169.62   3173.69   3176.23   3181.01   3185.14   3186.34
Note: any frequencies below 100 cm-1 (including spurious imaginary ones) are upscaled to 100 cm-1
      for the calculation of thermodynamic properties.
      (Averkiev, Truhlar, Catal. Sci. Technol. 2011, 1, 1526)

Thermodynamics

Note: this script does not take into account the spin entropy
For more info, see eq. 3 of Inorg. Chem. 2002, 41, 6928-6935
(M. Reiher), https://doi.org/10.1021/ic025891l

Temperature is now: 298.150

Reporting max. of 0 frequencies (set by $GETFREQSMAX env. variable)


Reading 1 outputfiles

            ScnFrq(1)
---------------------
---------------------
(ZPVE)        229.067
(dH,0->T)      16.732
(-TS)         -49.869
(dGibbs)      195.930
=====================

Corresponding output files
 1 : frq_ts_FOMe-gamma-AB-pA_1_2.1292706.out


System ts_FOMe-gamma-BC-pA_1_2

Reading 1 outputfiles

       Pauli        Elstat        OrbInt       Disp.       Solv.         TOTAL          Erel   Symm.      <S2>
-----------------------------------------------------------------------------------------------------------------
-----
   28367.546     -6045.627    -29213.372     -24.961     -50.518     -6967.106         0.000   NOSYM     0.755
ADF(1)

Corresponding output files
 1 : ts_FOMe-gamma-BC-pA_1_2.1303653.out

Coordinates (Angs)
  48
ts_FOMe-gamBa-BC-pA_1_2.1303653.out        -6967.1009
C      -3.481906   -3.296212    2.027053
C      -2.479573   -2.548025    2.633606
C      -1.939081   -1.408091    2.050223
C      -2.508676   -0.911206    0.781262
C      -3.531016   -1.760859    0.152517
C      -3.996345   -2.881210    0.803994
C      -0.843093   -0.699838    2.701101
N       0.000000    0.000000    2.003457
C       1.073701    0.711419    2.697804
C       2.700020    0.748592    0.780026
N       1.989989   -0.000000   -0.083598
C       2.613753   -0.748312   -1.004044
C       3.995054   -0.794431   -1.095371
C       4.746053   -0.036015   -0.205200
C       4.091121    0.743505    0.739035
Cu      0.000000    0.000000    0.000000
O      -0.116293   -0.007520   -1.864942
H      -2.396396    0.643403    4.393445
O      -1.949366    0.056324    0.080425
H       1.967539   -1.300797   -1.680434
H       4.464240   -1.413397   -1.854641
H       5.832743   -0.047325   -0.247548
H       4.647477    1.355084    1.444141
H       1.712348   -0.006727    3.226183
H       1.280449    2.273057    1.226833
H      -1.069157   -0.035445   -2.040388
H       0.815576   -2.236427    4.286659
H       1.801819   -3.406972    8.426677
H      -0.784806   -0.906937    8.056495
```

```
H      -3.486203   -0.486229    1.294397
H      -3.956018   -1.421676   -0.788225
H      -2.086645   -2.885196    3.588470
H      -3.880101   -4.186792    2.504243
F      -4.960517   -3.608729    0.222386
H       2.609491    2.150206    2.376401
C       1.921444    1.561145    1.764393
H       0.623016    1.355283    3.463007
H      -2.355460    0.563569    6.871681
C      -0.781696   -0.780269    4.186472
C       0.118269   -1.611957    4.841204
C       0.117977   -1.672576    6.242248
C      -0.772943   -0.879666    6.971875
C      -1.662735   -0.048552    6.298277
C      -1.687554    0.002324    4.911537
O       1.010194   -2.530175    6.791933
C       1.031067   -2.664212    8.214335
H       1.292994   -1.716125    8.699986
H       0.064933   -3.020510    8.592794

Frequencies (cm-1)
 -597.74    -32.99    -24.64    -18.00    100.00    100.00    100.00    100.00    100.00    100.00
  119.28    128.40    158.20    167.74    175.50    194.44    200.20    217.49    226.51    240.93
  250.10    253.76    262.84    290.95    304.66    322.86    338.17    352.97    365.86    393.77
  407.67    409.26    422.24    442.51    446.46    488.60    506.02    516.30    537.73    545.24
  556.44    566.87    573.42    596.34    618.81    641.16    645.55    687.80    708.01    723.56
  739.27    743.41    754.87    767.96    776.64    822.74    826.47    837.79    841.42    855.60
  863.47    875.45    876.26    915.18    935.97    951.50    958.42    961.29    963.04    982.79
  983.84    994.91   1015.79   1024.05   1043.69   1059.01   1072.56   1094.39   1102.82   1109.36
 1123.70   1137.26   1150.02   1156.42   1160.43   1169.32   1172.06   1204.38   1219.86   1237.98
 1250.32   1274.00   1280.79   1295.42   1297.06   1324.27   1328.31   1342.69   1346.71   1371.59
 1377.41   1392.80   1409.26   1413.52   1427.89   1436.12   1437.05   1444.49   1457.77   1476.30
 1479.45   1522.29   1567.40   1581.19   1585.56   1586.03   1614.72   1616.11   2373.23   2970.76
 3001.54   3008.39   3061.26   3063.21   3096.70   3121.71   3143.92   3148.25   3152.24   3160.05
 3164.14   3165.24   3168.87   3178.90   3179.01   3182.06   3182.89   3737.76
Note: any frequencies below 100 cm-1 (including spurious imaginary ones) are upscaled to 100 cm-1
      for the calculation of thermodynamic properties.
      (Averkiev, Truhlar, Catal. Sci. Technol. 2011, 1, 1526)

Thermodynamics

Note: this script does not take into account the spin entropy
For more info, see eq. 3 of Inorg. Chem. 2002, 41, 6928-6935
(M. Reiher), https://doi.org/10.1021/ic025891l

Temperature is now: 298.150

Reporting max. of 0 frequencies (set by $GETFREQSMAX env. variable)


Reading 1 outputfiles

            ScnFrq(1)
---------------------
---------------------
(ZPVE)        226.848
(dH,0->T)      16.962
(-TS)         -50.326
(dGibbs)      193.485
=====================

Corresponding output files
 1 : frq_ts_FOMe-gamma-BC-pA_1_2.1304130.out


System ts_FOMe-gamma-CD-pA_1_2

Reading 1 outputfiles

       Pauli        Elstat        OrbInt       Disp.       Solv.         TOTAL          Erel   Symm.      <S2>
----------------------------------------------------------------------------------------------------------------
-----
   28517.591     -6069.346    -29373.883     -26.043     -43.597     -6995.454         0.000   NOSYM     0.753
ADF(1)

Corresponding output files
 1 : ts_FOMe-gamma-CD-pA_1_2.1284213.out

Coordinates (Angs)
  48
ts_FOMe-gamBa-CD-pA_1_2.1284213.out        -6995.4580
C       1.932789   -1.776945    4.931001
C       1.419322   -0.642463    4.326290
C       2.330234    0.345193    3.807563
C       3.739308   -0.029577    3.667002
C       4.168389   -1.210511    4.363035
C       3.308387   -2.072112    4.979244
C      -0.024107   -0.485665    4.069755
H       0.000000    0.000000    6.720632
O       1.939075    1.425374    3.266741
Cu      1.092935    0.385406    1.630629
O       2.920714   -0.000000    1.056187
C      -1.326432   -0.357815   -2.409874
C      -2.067893   -0.500579    1.287460
H      -2.417501    0.252973    3.272929
```

```
H      -1.670877    1.421964    2.173848
N       0.000000    0.000000    0.000000
H      -3.059618   -0.698481   -1.178771
C      -1.318029   -0.293674    0.002070
N      -0.342974    0.050495    2.929525
H      -3.131673   -0.341631    1.087710
H      -1.963687   -1.559416    1.563752
C       0.653673    0.107596   -1.172547
H       0.608053    0.031872   -3.310018
C      -1.997806   -0.468601   -1.203725
C       0.031508   -0.064039   -2.394794
H       1.240329   -2.500131    5.357267
H       3.674204   -2.969082    5.468557
F       5.482695   -1.456595    4.371157
H       4.450532    0.799924    3.650801
H       3.581039   -0.206971    2.454244
H       3.374383    0.793175    0.733116
O      -3.675331   -2.212436    7.978389
H      -5.462120   -2.513080    6.934306
C      -4.809022   -3.021445    7.653741
H      -4.498960   -3.994297    7.254006
C      -0.989101   -0.974083    5.063339
C      -2.062071   -1.798488    4.707643
C      -2.973340   -2.247252    5.650607
C      -2.834062   -1.855885    6.987004
C      -1.750434   -1.044965    7.361435
C      -0.835396   -0.626766    6.416319
H      -2.174462   -2.133702    3.680449
H      -3.780832   -2.900760    5.338155
H      -5.342724   -3.166099    8.594186
H      -1.645141   -0.754421    8.404164
H       1.715046    0.326194   -1.104679
H      -1.856689   -0.500277   -3.348532
C      -1.675358    0.366257    2.476718

Frequencies (cm-1)
 -512.07    -38.35    -20.49     -4.53    100.00    100.00    100.00    100.00    100.00    108.33
  122.82    143.06    155.22    158.88    165.24    198.68    210.88    213.72    225.29    233.64
  255.02    273.59    285.88    310.89    328.64    342.33    355.28    396.41    400.57    406.20
  419.25    425.47    445.03    451.61    471.91    502.63    508.07    512.23    536.80    558.30
  584.07    590.21    608.82    624.48    635.98    641.50    671.90    691.14    715.14    730.49
  738.17    741.42    748.70    776.90    794.15    796.72    805.84    815.65    827.20    860.29
  877.31    879.65    910.20    937.05    951.69    954.54    957.12    981.63    984.93    994.92
  996.00   1011.73   1020.00   1025.88   1067.79   1080.72   1091.48   1111.60   1114.72   1121.69
 1129.26   1151.89   1162.62   1166.96   1173.92   1195.58   1211.89   1229.57   1247.59   1269.61
 1281.81   1292.09   1299.76   1304.96   1313.82   1321.73   1323.34   1348.09   1356.50   1363.14
 1393.51   1401.14   1415.15   1428.80   1429.23   1429.91   1434.99   1441.95   1479.43   1485.47
 1502.15   1523.53   1571.92   1579.47   1581.77   1602.97   1615.61   1621.51   1652.97   2975.35
 2992.41   3000.60   3069.67   3070.52   3081.94   3099.79   3123.19   3145.79   3148.22   3151.61
 3160.70   3162.39   3165.65   3169.59   3181.44   3182.98   3185.64   3745.28
Note: any frequencies below 100 cm-1 (including spurious imaginary ones) are upscaled to 100 cm-1
      for the calculation of thermodynamic properties.
      (Averkiev, Truhlar, Catal. Sci. Technol. 2011, 1, 1526)

Thermodynamics

Note: this script does not take into account the spin entropy
For more info, see eq. 3 of Inorg. Chem. 2002, 41, 6928-6935
(M. Reiher), https://doi.org/10.1021/ic025891l

Temperature is now: 298.150

Reporting max. of 0 frequencies (set by $GETFREQSMAX env. variable)


Reading 1 outputfiles

            ScnFrq(1)
---------------------
---------------------
(ZPVE)        227.359
(dH,0->T)      16.649
(-TS)         -49.733
(dGibbs)      194.275
=====================

Corresponding output files
 1 : frq_ts_FOMe-gamma-CD-pA_1_2.1284597.out


System ts_FOMe-ipso-AO-pA_1_2

Reading 1 outputfiles

       Pauli        Elstat        OrbInt       Disp.       Solv.         TOTAL          Erel   Symm.      <S2>
---------------------------------------------------------------------------------------------------------------------
-----
   28091.797     -5946.890    -29014.492     -25.957     -46.388     -6942.100         0.000   NOSYM     0.760
ADF(1)

Corresponding output files
 1 : ts_FOMe-ipso-AO-pA_1_2.1284215.out

Coordinates (Angs)
  48
```

```
ts_FOMe-ipsoBa-AF-pA_1_2.1284215.out        -6942.1129
C      -0.315912   -4.033539    0.600447
C      -0.029000   -2.820248    1.205908
C      -0.977340   -2.176156    2.015022
C      -2.219133   -2.787685    2.230163
C      -2.528542   -3.991869    1.617473
C      -1.569590   -4.588783    0.811013
C      -0.650470   -0.891248    2.650297
N       0.000000   -0.000000    1.956219
C       0.707269    1.122848    2.569475
C       2.573368    0.813987    0.855863
N       1.948886   -0.000000   -0.018685
C       2.645936   -0.862855   -0.772101
C       4.022846   -0.964049   -0.675261
C       4.684678   -0.147162    0.234526
C       3.954260    0.750266    1.003154
Cu      0.000000    0.000000    0.000000
O      -0.635873    0.091955   -1.784724
H      -1.923854    1.273738    3.628809
O      -1.759003    0.028176   -0.405889
H       2.072824   -1.478336   -1.462364
H       4.556484   -1.672670   -1.301378
H       5.765096   -0.205278    0.341828
H       4.445758    1.409560    1.712912
H      -0.407757   -2.591055    4.768104
H      -1.192508   -2.198907    7.081198
H      -0.812633    1.028708   -1.990107
O      -2.389926   -0.134849    7.931767
H      -3.278431    0.927076    9.398982
C      -3.061875    1.061471    8.338318
H      -2.957119   -2.304045    2.864692
H      -3.493908   -4.470452    1.757610
H       0.965162   -2.394394    1.097273
H       0.417241   -4.555423   -0.008670
F      -1.861686   -5.767870    0.222082
H      -3.999275    1.193752    7.785310
C       1.700361    1.771687    1.600727
H      -2.732470    1.648439    5.894416
H      -2.419258    1.939030    8.200972
C      -1.069857   -0.666233    4.035545
C      -0.901994   -1.655591    5.020488
C      -1.338119   -1.443738    6.312222
C      -1.991281   -0.246738    6.651118
C      -2.198897    0.731183    5.669588
C      -1.727383    0.519502    4.386191
H       1.228586    0.756277    3.461830
H       0.012646    1.905057    2.887594
H       2.314793    2.472487    2.171522
H       1.145889    2.369864    0.864225

Frequencies (cm-1)
 -639.52    -31.04    -25.78    100.00    100.00    100.00    100.00    100.00    100.00    100.00
  100.00    102.37    112.12    132.17    150.09    163.08    186.47    191.73    215.20    221.35
  222.95    245.55    284.40    301.38    309.13    339.95    355.55    382.71    405.59    407.08
  413.93    415.90    431.54    466.13    491.18    500.46    503.69    516.33    555.14    563.02
  585.77    593.29    612.65    615.75    625.33    655.44    674.66    700.37    732.68    743.71
  749.53    757.45    778.27    795.34    799.01    801.79    817.79    825.70    830.68    858.65
  879.23    918.68    925.98    943.66    949.30    952.90    955.62    957.79    974.86    996.31
  997.11   1001.78   1019.06   1023.19   1028.13   1052.01   1064.03   1099.52   1110.27   1114.62
 1121.71   1145.86   1150.57   1162.97   1168.09   1180.83   1191.25   1212.31   1216.12   1232.55
 1272.68   1274.83   1286.76   1296.29   1311.50   1325.31   1330.52   1347.05   1360.26   1366.34
 1411.46   1414.42   1416.00   1420.89   1428.98   1432.64   1438.73   1443.39   1481.68   1492.45
 1503.18   1549.85   1568.82   1583.97   1595.20   1607.88   1618.41   1620.78   2976.54   3001.18
 3010.97   3072.07   3081.36   3098.29   3127.26   3146.57   3147.90   3154.59   3155.94   3156.73
 3158.57   3162.91   3172.53   3172.86   3174.09   3182.17   3185.82   3681.89
Note: any frequencies below 100 cm-1 (including spurious imaginary ones) are upscaled to 100 cm-1
      for the calculation of thermodynamic properties.
      (Averkiev, Truhlar, Catal. Sci. Technol. 2011, 1, 1526)

Thermodynamics

Note: this script does not take into account the spin entropy
For more info, see eq. 3 of Inorg. Chem. 2002, 41, 6928-6935
(M. Reiher), https://doi.org/10.1021/ic025891l

Temperature is now: 298.150

Reporting max. of 0 frequencies (set by $GETFREQSMAX env. variable)


Reading 1 outputfiles

            ScnFrq(1)
--------------------
--------------------
(ZPVE)        229.027
(dH,0->T)      16.981
(-TS)         -50.854
(dGibbs)      195.154
====================

Corresponding output files
 1 : frq_ts_FOMe-ipso-AO-pA_1_2.1284599.out
```

```
System ts_FOMe-ipso-FG-pA_1_2

Reading 1 outputfiles

       Pauli        Elstat        OrbInt       Disp.       Solv.         TOTAL          Erel   Symm.      <S2>
----------------------------------------------------------------------------------------------------------------------
-----
   28259.898     -5995.299    -29153.270     -26.607     -51.567     -6967.016         0.000   NOSYM     0.754
ADF(1)

Corresponding output files
 1 : ts_FOMe-ipso-FG-pA_1_2.1284217.out

Coordinates (Angs)
  48
ts_FOMe-ipsoBa-FG-pA_1_2.1284217.out        -6967.0027
C      -3.225795   -2.707678    2.265386
C      -2.336927   -2.040382    1.475086
C      -2.317420   -0.574106    1.406623
C      -3.432820    0.110488    2.050786
C      -4.341319   -0.565294    2.816981
C      -4.200091   -1.953242    2.938319
C      -1.039723   -0.382292    2.621218
N       0.000000    0.000000    2.012881
C       1.202801    0.411141    2.744665
C       2.768350    0.600976    0.797420
N       2.000522    0.000000   -0.133777
C       2.578348   -0.695727   -1.125613
C       3.952772   -0.832173   -1.229776
C       4.756996   -0.220263   -0.276410
C       4.156001    0.504303    0.743211
Cu      0.000000    0.000000    0.000000
O      -0.202388   -0.088780   -1.866403
H      -2.471767    0.933109    4.579122
O      -1.897536   -0.007243    0.266068
H       1.896026   -1.138272   -1.845740
H       4.375677   -1.410923   -2.045740
H       5.840179   -0.306486   -0.323224
H       4.752704    0.997091    1.505976
H       0.096088   -2.443123    3.816973
H      -0.002294   -3.070392    6.174976
H      -1.157603   -0.188018   -1.994474
O      -1.398301   -1.660630    8.052748
H      -0.942654   -2.786574    9.662656
C      -0.707943   -2.789178    8.597398
H      -3.500434    1.184667    1.898042
H      -5.169625   -0.067540    3.313237
H      -1.580322   -2.569548    0.899628
H      -3.229757   -3.789931    2.357696
F      -5.060579   -2.600306    3.709751
H       0.375262   -2.691559    8.458318
C       2.074899    1.330111    1.902927
H      -2.547791    0.317939    6.967793
H      -1.061273   -3.723222    8.144834
C      -1.178351   -0.712124    4.024629
C      -0.485069   -1.832528    4.504502
C      -0.540809   -2.190192    5.840220
C      -1.286170   -1.410387    6.734578
C      -1.977979   -0.283148    6.263307
C      -1.936288    0.053384    4.925243
H       1.761085   -0.491589    3.021764
H       0.902934    0.913190    3.670090
H       2.819651    1.788071    2.559011
H       1.460630    2.143367    1.494747

Frequencies (cm-1)
 -278.14    -37.21    -21.61     -8.01    100.00    100.00    100.00    100.00    100.00    100.00
  114.99    133.62    145.05    161.87    165.83    178.40    182.07    208.50    210.49    223.40
  241.89    260.07    282.51    310.90    316.63    328.77    343.35    354.31    379.70    390.35
  401.90    409.75    414.70    427.82    452.32    487.62    492.43    497.49    510.05    520.48
  563.07    572.74    600.22    604.49    631.26    643.39    657.97    667.52    725.58    740.84
  743.85    757.20    773.76    779.82    785.48    792.72    807.41    820.89    826.72    834.24
  835.92    878.00    894.53    936.92    949.76    951.43    954.95    958.37    968.56    974.14
  992.48    995.49   1006.87   1016.98   1022.84   1059.64   1069.11   1073.58   1107.33   1114.77
 1122.72   1124.09   1150.38   1161.59   1163.22   1168.67   1196.76   1217.50   1223.55   1238.10
 1241.08   1257.67   1272.94   1287.66   1294.06   1305.34   1320.97   1327.07   1341.70   1349.67
 1404.40   1410.29   1414.34   1427.83   1430.29   1435.81   1436.85   1441.21   1465.83   1477.90
 1495.93   1510.56   1563.89   1584.73   1597.32   1609.47   1616.07   1677.24   2976.27   3008.28
 3013.28   3071.45   3077.11   3097.04   3128.31   3145.44   3151.36   3152.71   3153.36   3161.70
 3163.86   3168.18   3168.42   3176.39   3176.91   3179.31   3185.98   3745.63
Note: any frequencies below 100 cm-1 (including spurious imaginary ones) are upscaled to 100 cm-1
      for the calculation of thermodynamic properties.
      (Averkiev, Truhlar, Catal. Sci. Technol. 2011, 1, 1526)

Thermodynamics

Note: this script does not take into account the spin entropy
For more info, see eq. 3 of Inorg. Chem. 2002, 41, 6928-6935
(M. Reiher), https://doi.org/10.1021/ic025891l

Temperature is now: 298.150

Reporting max. of 0 frequencies (set by $GETFREQSMAX env. variable)
```

```
Reading 1 outputfiles

            ScnFrq(1)
---------------------
---------------------
(ZPVE)        228.022
(dH,0->T)      17.039
(-TS)         -50.511
(dGibbs)      194.550
=====================

Corresponding output files
 1 : frq_ts_FOMe-ipso-FG-pA_1_2.1284603.out


System ts_FOMe-ipso-GH-pA_1_2

Reading 1 outputfiles

       Pauli        Elstat        OrbInt       Disp.       Solv.         TOTAL          Erel   Symm.      <S2>
-----------------------------------------------------------------------------------------------------------------
-----
   28434.053     -6052.703    -29299.469     -26.243     -43.073     -6987.607         0.000   NOSYM     0.752
ADF(1)

Corresponding output files
 1 : ts_FOMe-ipso-GH-pA_1_2.1284219.out

Coordinates (Angs)
  48
ts_FOMe-ipsoBa-GH-pA_1_2.1284219.out        -6987.6093
C      -2.987101   -2.155325    2.195094
C      -2.049854   -1.204988    1.601441
C      -2.637563   -0.106607    0.777510
C      -4.050332    0.033359    0.707088
C      -4.860221   -0.906813    1.275832
C      -4.310616   -2.011086    1.978177
C      -0.923526   -0.729898    2.510328
N       0.000000    0.000000    1.995353
C       1.069192    0.654646    2.731263
C       2.654588    0.695437    0.771644
N       1.933639    0.000000   -0.135902
C       2.549847   -0.773972   -1.043421
C       3.928919   -0.880582   -1.100562
C       4.688245   -0.171552   -0.178029
C       4.043719    0.619503    0.763811
Cu      0.000000    0.000000    0.000000
O      -0.548261   -1.819565   -0.642408
H      -2.841110   -0.193709    4.375585
O      -1.869748    0.619530    0.076791
H       1.898591   -1.321118   -1.719872
H       4.389141   -1.516266   -1.851066
H       5.773695   -0.237027   -0.185931
H       4.609571    1.183004    1.500461
H       0.946296   -2.165661    3.818641
H       0.988313   -2.789653    6.178128
H      -1.234785   -1.765674   -1.323766
H       1.711912   -0.081925    3.224250
H       0.633219    1.285971    3.515445
H       2.625313    2.083281    2.381796
H      -4.456951    0.871225    0.147282
H      -5.942323   -0.834549    1.189739
H      -1.452840   -1.745124    0.691764
H      -2.607766   -2.977007    2.795534
O      -0.957095   -2.085260    7.961668
H       1.066508   -2.260715    8.459488
C       1.910075    1.509664    1.786664
H      -2.781606   -0.786999    6.773586
H       1.258910    2.232841    1.277504
C      -0.934861   -1.116297    3.930300
C       0.131077   -1.844559    4.462884
C       0.155314   -2.205249    5.801996
C      -0.892426   -1.811498    6.642012
C      -1.970798   -1.084673    6.112724
C      -1.998132   -0.756411    4.771138
F      -5.188215   -2.916579    2.470934
H      -0.138711   -2.894612    9.617673
C       0.121115   -2.807228    8.561649
H       0.219495   -3.807817    8.123664

Frequencies (cm-1)
 -302.38    -29.20    -22.37     -5.81    100.00    100.00    100.00    100.00    100.00    111.68
  112.11    137.21    160.82    168.20    172.53    185.87    205.10    220.77    234.13    242.34
  271.02    285.98    297.33    311.84    328.23    355.74    365.42    387.55    401.32    405.64
  407.20    421.65    446.64    454.09    485.03    493.95    504.18    515.48    525.02    541.33
  574.46    599.70    615.94    622.57    638.93    650.56    666.58    684.13    732.07    741.45
  743.58    755.11    773.08    776.55    793.72    797.52    814.09    818.42    829.49    847.61
  876.32    883.79    924.29    937.96    947.08    949.85    955.93    959.96    995.09    995.36
 1006.55   1016.79   1023.65   1025.32   1054.91   1062.85   1074.61   1109.27   1113.23   1115.37
 1123.36   1150.85   1162.43   1164.89   1169.01   1169.57   1202.13   1217.65   1225.06   1248.95
 1265.25   1275.08   1288.43   1296.37   1304.06   1316.26   1319.01   1324.11   1337.63   1356.59
 1403.27   1414.78   1415.40   1423.48   1426.56   1429.40   1435.76   1440.55   1476.57   1480.07
 1500.69   1547.26   1573.11   1580.79   1602.54   1615.71   1621.27   1644.11   1700.12   2973.71
 3007.06   3007.87   3067.28   3073.16   3098.31   3124.66   3143.08   3148.18   3153.18   3155.44
 3161.52   3162.07   3169.82   3171.45   3172.71   3180.78   3183.98   3752.00
```

```
Note: any frequencies below 100 cm-1 (including spurious imaginary ones) are upscaled to 100 cm-1
      for the calculation of thermodynamic properties.
      (Averkiev, Truhlar, Catal. Sci. Technol. 2011, 1, 1526)

Thermodynamics

Note: this script does not take into account the spin entropy
For more info, see eq. 3 of Inorg. Chem. 2002, 41, 6928-6935
(M. Reiher), https://doi.org/10.1021/ic025891l

Temperature is now: 298.150

Reporting max. of 0 frequencies (set by $GETFREQSMAX env. variable)


Reading 1 outputfiles

            ScnFrq(1)
---------------------
---------------------
(ZPVE)        227.464
(dH,0->T)      16.600
(-TS)         -49.593
(dGibbs)      194.471
=====================

Corresponding output files
 1 : frq_ts_FOMe-ipso-GH-pA_1_2.1284607.out


System ts_FOMe-ipso-OF-pA_1_2

Reading 1 outputfiles

       Pauli        Elstat        OrbInt       Disp.       Solv.         TOTAL          Erel   Symm.      <S2>
-----------------------------------------------------------------------------------------------------------------
-----
   28142.620     -5973.528    -29047.234     -25.980     -46.229     -6950.521         0.000   NOSYM     0.761
ADF(1)

Corresponding output files
 1 : ts_FOMe-ipso-OF-pA_1_2.1285646.out

Coordinates (Angs)
  48
ts_FOMe-ipsoBa-preFF-pA_1_2.1285646.out        -6950.5273
C      -2.238437   -3.486193    0.545534
C      -1.406514   -2.524375    1.076117
C      -1.940321   -1.359102    1.692792
C      -3.347616   -1.258653    1.856490
C      -4.182710   -2.197464    1.298552
C      -3.612562   -3.294359    0.649170
C      -1.033013   -0.525807    2.522901
N       0.000000    0.000000    1.941905
C       1.088295    0.650444    2.666884
C       2.677189    0.738333    0.743532
N       1.946605   -0.000000   -0.112217
C       2.542687   -0.772699   -1.033362
C       3.920593   -0.849123   -1.132638
C       4.695203   -0.092559   -0.262007
C       4.066428    0.708425    0.680585
Cu      0.000000    0.000000    0.000000
O      -0.130264   -0.019418   -1.812085
H      -1.270293    1.741142    4.078033
O      -1.772627   -0.080461    0.065963
H       1.881847   -1.321687   -1.697133
H       4.367317   -1.493793   -1.883494
H       5.780782   -0.126338   -0.314489
H       4.640164    1.315505    1.374932
H      -1.650895   -2.552426    4.143091
H      -2.315746   -2.472360    6.488692
H      -1.072941   -0.210282   -1.955849
O      -2.559635   -0.150517    7.905411
H      -3.178879   -0.936495    9.656380
C      -2.907857   -1.310163    8.668094
H      -3.759026   -0.406585    2.390021
H      -5.263581   -2.114559    1.367833
H      -0.328042   -2.660424    1.042323
H      -1.847330   -4.379981    0.068287
F      -4.429846   -4.219607    0.126891
H      -2.056582   -1.995518    8.753590
C       1.932734    1.535529    1.761508
H      -1.982601    1.825432    6.430875
H      -3.764317   -1.829709    8.222540
C      -1.367216   -0.421508    3.943694
C      -1.704854   -1.588015    4.642914
C      -2.083770   -1.546294    5.973866
C      -2.177422   -0.310733    6.625797
C      -1.873922    0.869138    5.924848
C      -1.462316    0.812517    4.609996
H       2.640944    2.087437    2.383757
H       1.292225    2.277458    1.265982
H       1.712435   -0.139040    3.106708
H       0.701317    1.258375    3.484214
```

```
Frequencies (cm-1)
 -214.36    -38.29    -21.89    -12.26    100.00    100.00    100.00    100.00    100.00    100.00
  113.55    129.16    154.48    156.72    171.72    195.10    200.68    217.90    218.78    248.96
  260.57    277.26    284.82    309.27    316.38    336.10    352.98    387.24    393.96    404.87
  409.38    411.01    421.76    438.94    457.48    482.52    502.36    516.25    549.13    569.70
  575.58    587.98    602.55    609.19    617.43    655.00    676.23    691.24    712.12    740.17
  744.81    750.82    775.93    780.75    791.07    799.76    811.97    814.09    822.78    862.13
  871.66    908.18    919.88    938.30    939.19    945.39    953.69    957.12    969.20    988.95
  995.07    996.34   1019.76   1024.88   1032.07   1062.82   1072.81   1099.42   1112.23   1116.70
 1121.95   1142.11   1151.06   1162.99   1165.77   1169.79   1185.36   1222.20   1231.34   1233.56
 1272.35   1273.97   1288.62   1295.55   1303.70   1320.19   1327.30   1342.53   1347.03   1362.60
 1410.33   1413.03   1414.31   1428.32   1431.90   1432.91   1438.47   1443.54   1466.56   1480.92
 1501.95   1544.04   1565.47   1579.75   1585.76   1598.23   1616.40   1621.54   2977.49   3003.44
 3012.78   3073.52   3104.41   3118.18   3127.89   3146.95   3148.81   3150.25   3155.79   3164.16
 3165.14   3170.79   3173.94   3178.51   3178.98   3184.38   3187.13   3692.05
Note: any frequencies below 100 cm-1 (including spurious imaginary ones) are upscaled to 100 cm-1
      for the calculation of thermodynamic properties.
      (Averkiev, Truhlar, Catal. Sci. Technol. 2011, 1, 1526)

Thermodynamics

Note: this script does not take into account the spin entropy
For more info, see eq. 3 of Inorg. Chem. 2002, 41, 6928-6935
(M. Reiher), https://doi.org/10.1021/ic025891l

Temperature is now: 298.150

Reporting max. of 0 frequencies (set by $GETFREQSMAX env. variable)


Reading 1 outputfiles

            ScnFrq(1)
---------------------
---------------------
(ZPVE)        229.078
(dH,0->T)      16.763
(-TS)         -50.004
(dGibbs)      195.836
=====================

Corresponding output files
 1 : frq_ts_FOMe-ipso-OF-pA_1_2.1285896.out
```
