# Supplementary figures and images for: Cu-Promoted ipso-Hydroxylation of sp2 Bonds with Concomitant Aromatic 1,2-Rearrangement Involving a Cu-oxyl-hydroxo Species
Source: Inorg Chem. 2024 Oct 18;63(43):20675–88. doi: 10.1021/acs.inorgchem.4c03304 (PMC11523237; doi:10.1021/acs.inorgchem.4c03304)

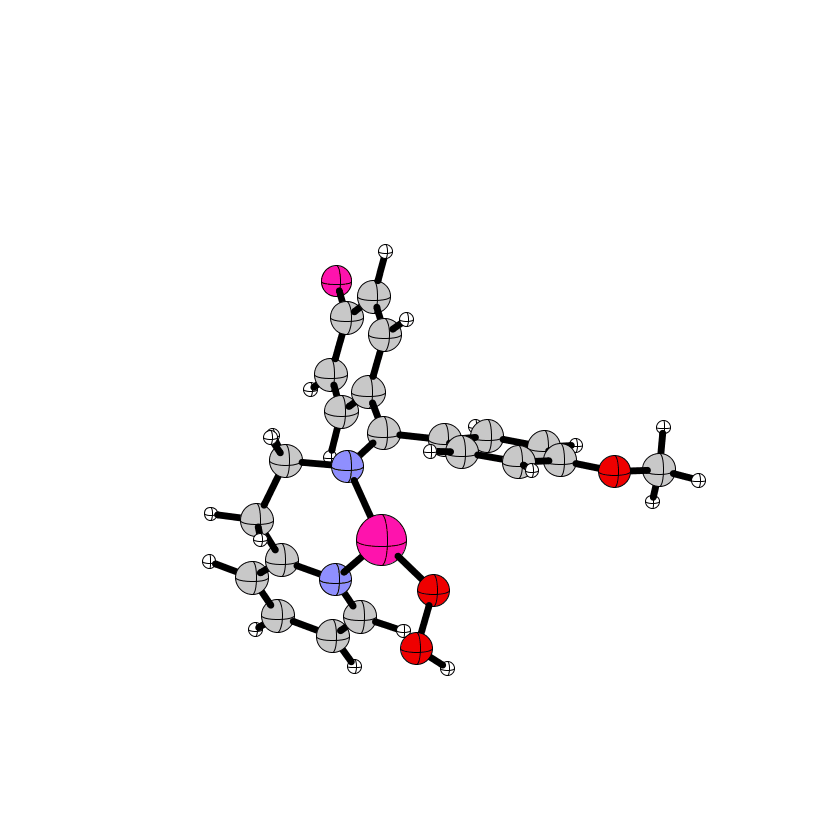

Supplement: Supplementary file 2 — ic4c03304_si_002.zip [file ic4c03304_si_002.zip › DFT Calculations/movie_gamma_pathway.gif]

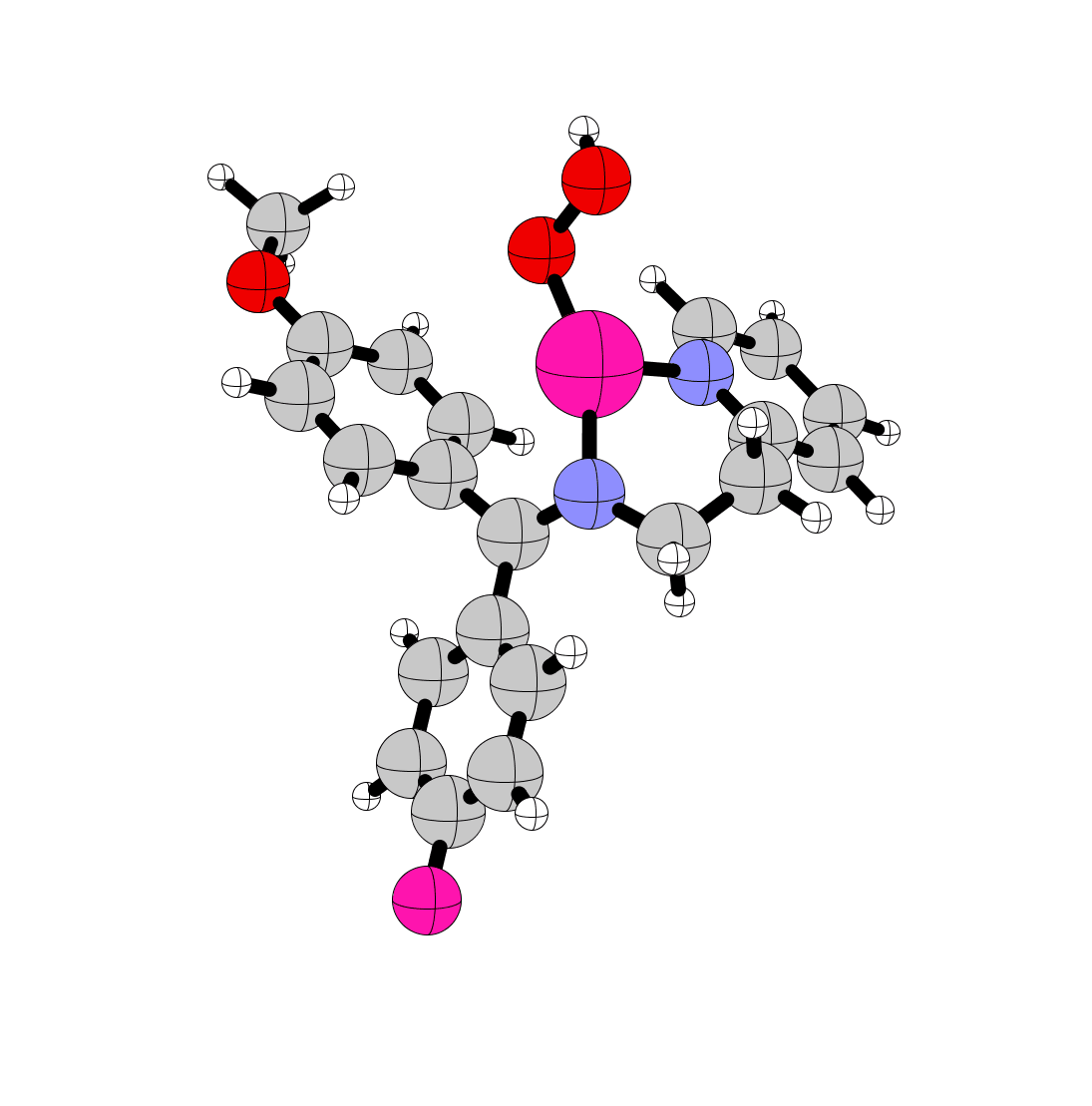

Supplement: Supplementary file 2 — ic4c03304_si_002.zip [file ic4c03304_si_002.zip › DFT Calculations/movie_beta_ipso_pathway.gif]
